# Genomic and Transcriptome Analysis to Identify the Role of the mTOR Pathway in Kidney Renal Clear Cell Carcinoma and Its Potential Therapeutic Significance

**DOI:** 10.1155/2021/6613151

**Published:** 2021-06-07

**Authors:** Xiangyu Che, Xiaochen Qi, Yingkun Xu, Qifei Wang, Guangzhen Wu

**Affiliations:** ^1^Department of Urology, The First Affiliated Hospital of Dalian Medical University, Dalian, Liaoning 116011, China; ^2^Department of Urology, Shandong Provincial Hospital, Cheeloo College of Medicine, Shandong University, Jinan, Shandong 250021, China

## Abstract

The mTOR pathway, a major signaling pathway, regulates cell growth and protein synthesis by activating itself in response to upstream signals. Overactivation of the mTOR pathway may affect the occurrence and development of cancer, but no specific treatment has been proposed for targeting the mTOR pathway. In this study, we explored the expression of mTOR pathway genes in a variety of cancers and the potential compounds that target the mTOR pathway and focused on an abnormal type of cancer, kidney renal clear cell carcinoma (KIRC). Based on the mRNA expression of the mTOR pathway gene, we divided KIRC patient samples into three clusters. We explored possible therapeutic targets of the mTOR pathway in KIRC. We predicted the IC50 of some classical targeted drugs to analyze their correlation with the mTOR pathway. Subsequently, we investigated the correlation of the mTOR pathway with histone modification and immune infiltration, as well as the response to anti-PD-1 and anti-CTLA-4 therapy. Finally, we used a LASSO regression analysis to construct a model to predict the survival of patients with KIRC. This study shows that mTOR scores can be used as tools to study various treatments targeting the mTOR pathway and that we can predict the recovery of KIRC patients through the expression of mTOR pathway genes. These research results can provide a reference for future research on KIRC patient treatment strategies.

## 1. Introduction

Renal cell carcinoma (RCC) is the eighth most common malignant tumor in the United States [1]. The estimated incidence of kidney cancer in 2020 was 74,000. The typical symptoms of kidney cancer patients, such as pain, lumps, and hematuria, account for only 10% of cases [2]. Due to the kidney's ability to compensate when there is damage, it is usually impossible to detect the loss of kidney function early. Therefore, RCC is clinically insidious in terms of the development of the disease. Approximately one-third of patients have metastatic disease at the time of diagnosis, and patients with locally advanced kidney cancer have a 40% risk of recurrence after tumor resection [3, 4]. The development of targeted therapy and immunotherapy in the past decade has filled the gap in the treatment of advanced kidney cancer. Although the tumor response rate to these drugs is relatively high, most patients eventually experience cancer progression. For current treatments, the emergence of drug resistance is a major challenge, forcing us to reconsider the treatment of RCC [5].

Current studies have shown that the emergence of drug resistance is related to the existence of tumor stem cells and the activation of other pathways [6]. The mechanisms include the activation of the WNT-*β*-catenin, TP53, c-Met, and VEGF/angiogenesis signaling pathways [7–9]. In addition, although the current tumor research has made unprecedented progress in cancer genetics, we have not yet reached a unified view of genetics, for example, combining gene mutations, copy number variations, driving pathways, and other aspects to overcome tumors [10]. Here, we analyzed gene mutations, copy number changes, gene expression, and gene prognosis correlation results of 33 tumors from The Cancer Genome Atlas (TCGA). We combined this analysis with functional research to dissect the components that identify specific temporal events that reflect the complexity of the mTOR signaling pathway.

The mTOR pathway senses and integrates multiple intracellular and environmental signals through two protein complexes with different structures and functions: mTOR complex 1 (mTORC1) and mTOR complex 2 (mTORC2) [11, 12]. mTOR signaling is usually involved in regulating cell survival, cell growth, cell metabolism, protein synthesis, autophagy, and homeostasis [13]. In addition, mTOR negatively regulates autophagy in different ways. The pathological relevance of mTOR signal dysregulation has been explained in many human diseases, especially in various human cancers. The mTOR signaling pathway has been reported to be overactivated in more than 70% of cancers [14]. It has been widely demonstrated in animal models and clinical cancer patients in the past few years [15, 16]. The regulation of the mTOR pathway is also affected by the positive and negative regulators that cross-talk with it, such as phosphoinositide 3-kinase (PI3K)/AKT, mitogen-activated protein kinase (MAPK), vascular endothelial growth factor (VEGF), nuclear factor *κ*B (NF*κ*B) and p53, which form a more complex signal cascade [17].

Therefore, this study used the mutation, expression, and clinical data from the TCGA database to analyze the CNV, SNV, and gene expression status of mTOR signaling pathway genes in 33 tumors and the relationship between each tumor and patient prognosis. Surprisingly, most of the genes in the mTOR pathway in clear cell renal cell carcinoma are protective for patient prognosis. To further explain this phenomenon, we used bioinformatics methods to analyze the mTOR pathway-related genes in kidney renal clear cell carcinoma (KIRC). This study is aimed at systematically evaluating mTOR pathway-related genes and the KIRC prognoses associated with them. Through the expression pattern of mTOR pathway-related genes in KIRC, the prognostic value and impact on immune correlation can improve prognostic risk stratification and promote treatment decisions in KIRC patients.

## 2. Materials and Methods

### 2.1. Acquisition of Gene Data and Patient Clinical Information Data

The mTOR pathway genes were identified using the REACTOME dataset in the gene set enrichment analysis (GSEA) website. We obtained 32 types of cancer and 40 mTOR pathway genes. The CNV, SNV, and gene expression data were downloaded from The Cancer Genome Atlas (https://portal.gdc.cancer.gov) database [18, 19]. We used the Perl language to analyze the data and TBtools to visualize the results. The RNA-seq KIRC cohort included 72 normal samples and 539 cancer samples.

### 2.2. Connectivity Map Analysis (CMap) and Mechanism of Action (MoA)

To determine which target drugs are useful for mTOR pathway therapy, we used the Broad Institute's Connectivity Map Build02 (CM), which allows users to predict compounds that can activate or inhibit tumors based on the gene expression characteristics of different tumors [20]. To further study the mechanism of action (MoA) and drug targets related to the mTOR pathway, we conducted a specific analysis using the Connectivity Map tool [10, 21]. We obtained 16 differential expression characteristics of mRNA by performing differential expression analyses on mTOR pathway gene expression samples. CMap is a method similar to GSEA; it is based on the Kolmogorov-Smirnov test's pattern-matching strategy, which is used to find similarities between differentially expressed genes (DEGs). Then, we compared the DEG rankings to determine the positive or negative regulatory relationship of the genes, thereby generating an enrichment score (ES) from -1 to 1, and finally sorted the above scores based on all of the case data in the database. For each cancer type, we obtained two tables that applied the connection diagram findings to the expression characteristics of the mTOR pathway. A *p* value < 0.05 was used as an inclusion criterion to determine the average meaningful compound of each tumor type. These compounds may inhibit or activate the mTOR pathway in tumors. We use the “GEOquery” package in R to get data from the Gene Expression Omnibus (GEO) database, the “xlsx,” “tidyverse,” “plyr,” and “circlize” packages to process and analyze the data, and the “pheatmap” package to plot the heat map.

### 2.3. Cluster Analysis Based on mTOR Scores

Because the gene expression profile in the previously obtained data set had a large variation, we constructed an mTOR-score model based on mRNA expression to show the differential expression between the samples. According to the expression of mRNA in normal tissues, the expression statuses of the mRNA in the tumor tissues were classified into three categories: mTOR active (cluster1), normal (cluster2), and mTOR inactive (cluster3). To further illustrate the relationship between gene expression levels among these three clusters, a violin plot was used to depict the enrichment score levels of the three clusters. Statistical significance was set at *p* < 0.05. The “gplots” package was used in RStudio for cluster analysis. We used the “survival” package in RStudio to plot the survival curve of the three clusters. A heat map was drawn by “pheatmap” in RStudio to describe the relationship between the three groups of clusters and the clinicopathological characteristics of the KIRC patients. Statistical significance was set at *p* < 0.05.

### 2.4. GDSC Database and pRRophetic Algorithm

The Genomics of Drug Sensitivity in Cancer (GDSC) is the largest public pharmacogenomics database (https://www.cancerrxgene.org/). We used the GDSC database to predict the chemotherapeutic response. We selected several classic and novel targeted drugs to treat KIRC. We used the “pRRophetic” package in R to perform the prediction process; a ridge regression was used to estimate the half-maximal inhibitory concentration (IC50) of the sample [22, 23]. We also used a 10-fold cross-validation based on the GDSC training set to estimate the precision of the prediction. Except for “combat” and “allSoldTumours” tissue types, we set all parameters to default. The repetitive expression of genes was summarized as an average value. Statistical significance was set at *p* < 0.05.

### 2.5. Classical Cancer-Related Genes and Histone Modification

For the mTOR pathway, the differential expression of known classical oncogenes and histone modification-related genes leads to activation or inhibition of the mTOR pathway. To explore the potential regulatory mechanism of the mTOR pathway in KIRC, we examined the expression levels of various oncogenes in three groups of clusters in the form of a heat map to explore the influence of differentially expressed oncogenes on the mTOR pathway. Using the same approach, we also demonstrated the relationship between the three clusters of mTOR pathways and two gene types, SIRT and HDAC, which are involved in histone modification. Statistical significance was set at *p* < 0.05.

### 2.6. Immune Cell Infiltration and Immunotherapy

**Figure 1:**
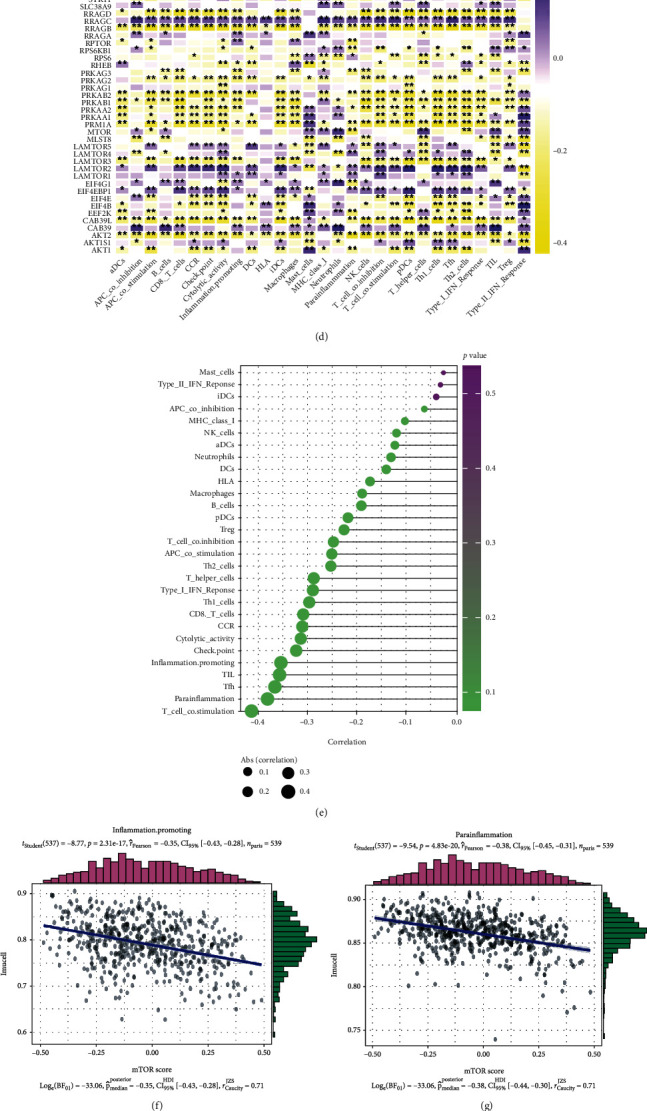
(a–c) Heat map showing that mTOR scores were associated with other signaling pathways in KIRC. (a) The correlation with potentially targetable classical genes. (b) The correlation with sirtuin family genes. (c) The correlation with HDAC family genes. (d) Heat map showing the correlation between mTOR pathway genes and substances related to immune filtering. Purple means positive and yellow means negative. ^∗^*p* < 0.05 and ^∗∗^*p* < 0.01. (e) Plot showing the degree of correlation; the area of the sphere represents the abs(correlation), and the color indicates the *p* value. (f–j) Scatter diagram showing the specific relationship between the five immune-infiltration-related substances and the mTOR scores. It can be seen from the diagram that they are all negatively correlated. (k) The plot shows the correlation analysis between PD-1, CTLA-4, and the mTOR scores. In the figure, a scatter plot and color block are, respectively, used to represent their specific correlation and correlation coefficients. (l) Submap analysis indicating that the mTOR-inactive cluster could be more sensitive to PD-1 (the programmed cell death protein 1) inhibitors (nominal *p* value = 0.029).

We used a single-sample gene set enrichment analysis combined with the expression of related genes in the TCGA database to quantify immune cells [24]. Then, a heat map expressing the correlation between the two was drawn using the “ggplot2” and “dplyr” packages in R. An ssGSEA analysis can be applied to gene signals expressed by immune cell populations in a single sample. Twenty-nine types of immune cells and regulators used in this study involved innate and adaptive immunity. Based on the results of ssGSEA, we showed the correlation between mTOR scores and the immune substances in Figure 1(e), where the area of a sphere represents the degree of correlation. The color represents the *p* value. R software packages “data.table,” “dplyr,” “tidyr,” “ggplot2,” and “ggstatsplot” were used to analyze and plot the figure. We then selected six classical immune regulators: inflammation, promotion, parainflammation, T cell costimulation, Tfh, and TIL. We used the “ggscatterstats” package to draw the scatter diagram to represent their specific correlations to the mTOR scores separately. PD-1 and CTLA-4 are two types of immune regulatory factors related to T cell-killing tumor cells [25–27]. The correlation between CTLA-4, PD-1, and mTOR scores was demonstrated through a visual correlation matrix analysis. The three graphs in the upper right of Figure 1(k) correspond to correlation coefficients, while the three graphs in the lower left show a specific correlation. Based on the correlation between PD-1, CTLA-4, and mTOR scores, we hypothesized that immunotherapy based on PD-1 and CTLA-4 would respond to the mTOR pathway. TIDE and subclass mapping are two algorithms used to predict a single sample's response possibility or a subtype of immunotherapy [26–28]. Their source sites are http://tide.dfci.harvard.edu/ and https://cloud.genepattern.org/gp. TIDE was used to predict single-sample immune checkpoint inhibitor response, and a submap was used to predict the immunotherapy responses of the subtypes. We used a Bonferroni correction to correct the *p* value of the test level. Finally, the heat map was plotted using the “pheatmap” package. Statistical significance was set at *p* < 0.05.

### 2.7. Construction of the Prediction Model with a LASSO Regression Analysis

We used “pheatmap” to describe the expression levels of mTOR pathway genes in normal and KIRC tissues. We used “corrplot” to describe the coexpression relationship between any two of the mTOR pathway genes. A hazard ratio analysis was performed to analyze the relationship between the pathway and progression of KIRC. A LASSO regression curve using the “glmnet” package was used to establish a risk model. Risk score = ∑*ni* = 1 (Expi∗Coei); *N*, Coei, and Expi represent the gene number, the regression correlation coefficient obtained by the LASSO regression analysis, and the gene expression level, respectively. We determined the cut-off value of each risk score in the tumor group using the “survminer” package. We divided the samples into high-risk and low-risk groups based on the best cut-off values. We acquired the survival curve of the two groups with the “survival” package in RStudio. Then, we used the “survival-ROC” package to plot the ROC curve and get the AUC value. We used a heat map to show the correlation of clinicopathological features between the low-risk and high-risk groups. Statistical significance was set at *p* < 0.05.

### 2.8. Validation of the Prediction Model and Nomogram

We use the Sankey diagram plotted by the “ggalluvial” package to show the multiple attributes of protective and risky genes with statistical significance in an HR analysis. We obtained protein-related information from the HPA website (https://www.proteinatlas.org/) [29]. Univariate and multivariate Cox regression analyses were used to show the correlations between age, stage, grade, T (tumor), M (metastasis), and risk score in the model. N (node) was not included in the analysis because the sample quantity was not large enough to support the study. All statistical analyses were performed using RStudio. The nomogram was drawn by the “rms” package in R. Finally, in order to make our conclusion more convincing, we used KIRC clinical specimens to conduct immunohistochemical experiments on the two key molecules involved in the model, PRKAA2 and EIF4EBP1. A *p* value < 0.05 was considered statistically significant.

## 3. Results

### 3.1. Widespread Genetic Mutations of mTOR Pathway Genes in 32 Types of Cancer

**Figure 2:**
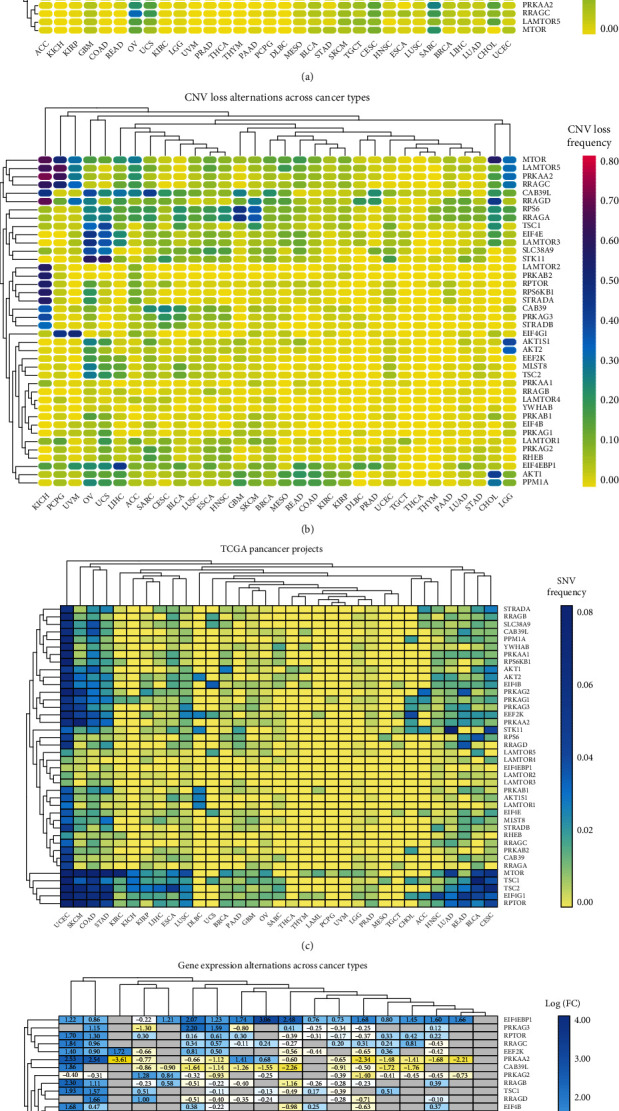
(a, b) The CNV frequencies of the 40 mTOR pathway genes are shown for the 32 tumor types. The color code bar refers to differential gain or loss of copy numbers on the right side; purple indicates a CNV gain and yellow indicates a CNV loss. (c) The SNV frequencies of the 40 mTOR pathway genes are shown for the 32 tumor types. The color code bar refers to the degree of SNV on the right side, with blue representing a high frequency and yellow representing a low frequency. (d) There were changes in the expression of 40 mTOR pathway genes among the 32 different types of cancer. The color code bar shows the corresponding value of log2(FC) on the right side.

Through the TCGA Pan-cancer Project, copy number variation (CNV), single-nucleotide variation (SNV), and gene expression levels (Figure 2) of 40 types of mTOR pathway-related genes and in 32 cancer types were studied. We downloaded the data from The Cancer Genome Atlas (TCGA) database and analyzed them using R [30]. We found that only a few types of cancer, such as THCA, THYM, and PRAD, had almost no CNV gains or CNV losses in mTOR pathway genes (Table S1, S2). In addition, CNV of mTOR pathway genes are present in most cancers. We also found that changes in SNV, the mTOR pathway gene, were also predominant in most cancers (Table S3). A large frequency of SNVs occurred in UCEC, SKCM, and COAD. To further study the expression of mTOR pathway genes in different cancers, we used log2(FC) of the gene expression level between normal and cancer tissues. We found that most of the mTOR pathway-related genes had a high expression level in most cancers, except for a few genes such as CAB39L and PRKAA2, which had a lower expression in most cancers compared to normal tissues. This was also consistent with the conclusion that mTOR is an oncogene-activated pathway (Table S4) [31, 32]. We also examined gene mutations in KIRC, as shown by the heat map. No large CNV or SNV values were noted; the CNV frequency was lower than 0.4, and most of the CNVs fluctuated around 0.2. For SNVs, the frequencies of all other genes were lower than 0.02, except for mTOR, which reached 0.06–0.08.

### 3.2. Connectivity Map (CMap) Analysis Identifying Potential Compounds/Inhibitors That Can Target the mTOR Pathway

**Figure 3:**
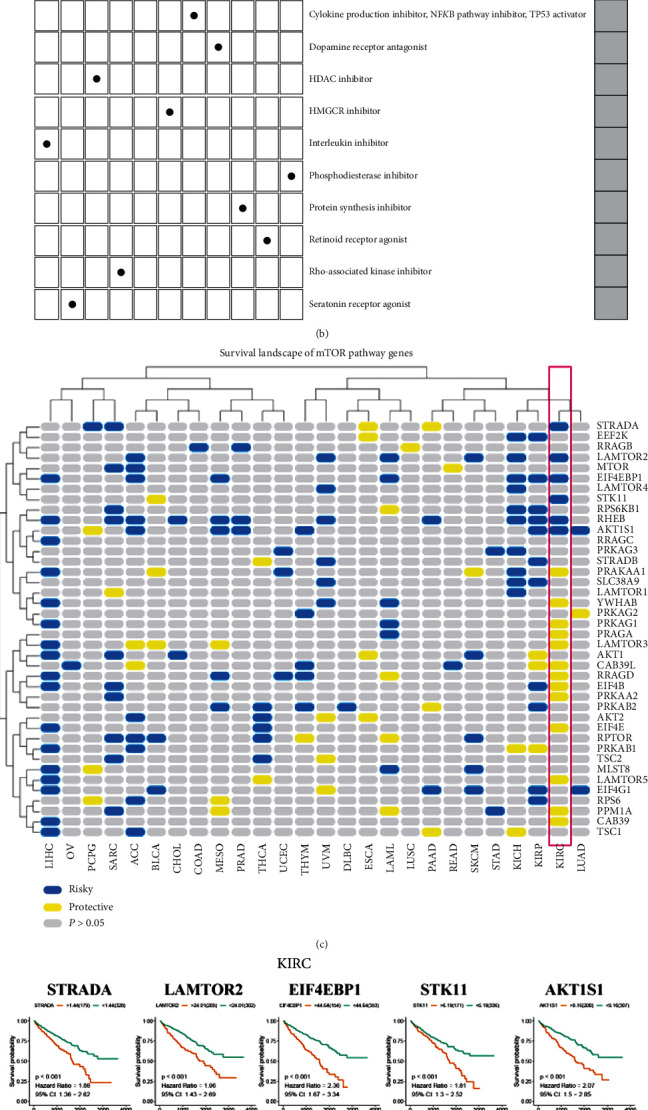
(a) Heat map shows each compound's enrichment score for each cancer type from the CMap. These are ordered from right to left in descending order of significant enrichment based on cancer type. The color bar refers to different enrichment scores: blue means positive and red means negative. (b) Heat map showing the mechanisms (column) shared by each compound (row) from the CMap. (c) Heat map showing the survival landscape of the mTOR pathway genes, with blue representing risky genes and yellow representing protective genes. The grey bar represents no statistical significance. (d) The survival curve of the statistically significant mTOR pathway genes in KIRC.

We used Connectivity Map (CMap) [33], a systematic approach that is driven by data, to discover links between genes, chemicals, and biological situations to search for compounds and inhibitors that might target mTOR-related pathways (Figure 3(a)). According to the results and the actual situation, most of these candidate compounds have been reported to be used against cancer. Some of the candidate compounds have been reported to directly or indirectly affect the mTOR pathway. bergenin has been reported to have anticancer effects in cervical cancer and bladder cancer [34, 35] and to have a relationship with the mTOR pathway [36]. There have also been reports indicating that mepacrine has anticancer effects [37] and that it is correlated with the mTOR pathway [38].

The CMap mode-of-action (MoA) analysis of the 11 compounds revealed their action mechanisms (Figure 3(b)). It is convenient to explore their common internal mechanisms. Interestingly, each of these 11 compounds has a separate anticancer mechanism of action: indoprofen (cyclooxygenase inhibitor and prostanoid receptor antagonist); mepacrine (cytokine production inhibitor, NF*κ*B pathway inhibitor, and TP53 activator), molindone (dopamine receptor antagonist), depudecin (HDAC inhibitor), lovastatin (HMGCR inhibitor), bergenin (interleukin inhibitor), zardaverine (phosphodiesterase inhibitor), rifabutin (protein synthesis inhibitor), TTNPB (retinoid receptor agonist), fasudil (Rho-associated kinase inhibitor), and buspirone (serotonin receptor agonist). The corresponding action mechanisms are shown in parentheses.

### 3.3. The Role of mTOR Genes in Cancer

The mammalian or mechanistic target of rapamycin (mTOR) pathway plays a vital role in cancer and regulates cell survival, metabolism, growth, and protein synthesis [16]. The mTOR signaling pathway has been reported to be overactivated in most human cancers [39]. It has been reported that excessive activation of the mTOR pathway and abnormal cell metabolism jointly lead to cancer occurrence [40]. We determined whether the genes involved exists as risky genes or as protective genes based on the relationship between patient survival rates and the mTOR pathway gene expression levels reported in the TCGA database. A high expression of a gene that led to an increased survival rate indicated that it was a protective gene, while a high expression of a gene that caused a decreased survival rate led it to be judged as a risky gene. We used this method to analyze the survival landscape of the mTOR pathway genes. As shown in the resulting figure, since the mTOR pathway itself is a cancer pathway, related genes are present as risky genes in most cancers (Figure 3(c)). However, we found an interesting result indicating that most mTOR-related genes exist as protective genes in KIRC, which contradicts previous studies' results. Therefore, we focused on the relationship between mTOR-related pathway genes and the survival rate of patients with KIRC. We plotted the Kaplan-Meier curve (K-M curve) for each of the statistically significant gene pathways based on patient survival data, according to *p* < 0.05. The results obtained are consistent with the conclusion of the survival landscape of mTOR pathway genes (Figure 3(d)). This suggests that there are still some underexplained roles of the mTOR pathway in KIRC.

### 3.4. Cluster Analysis Based on mTOR Scores

**Figure 4:**
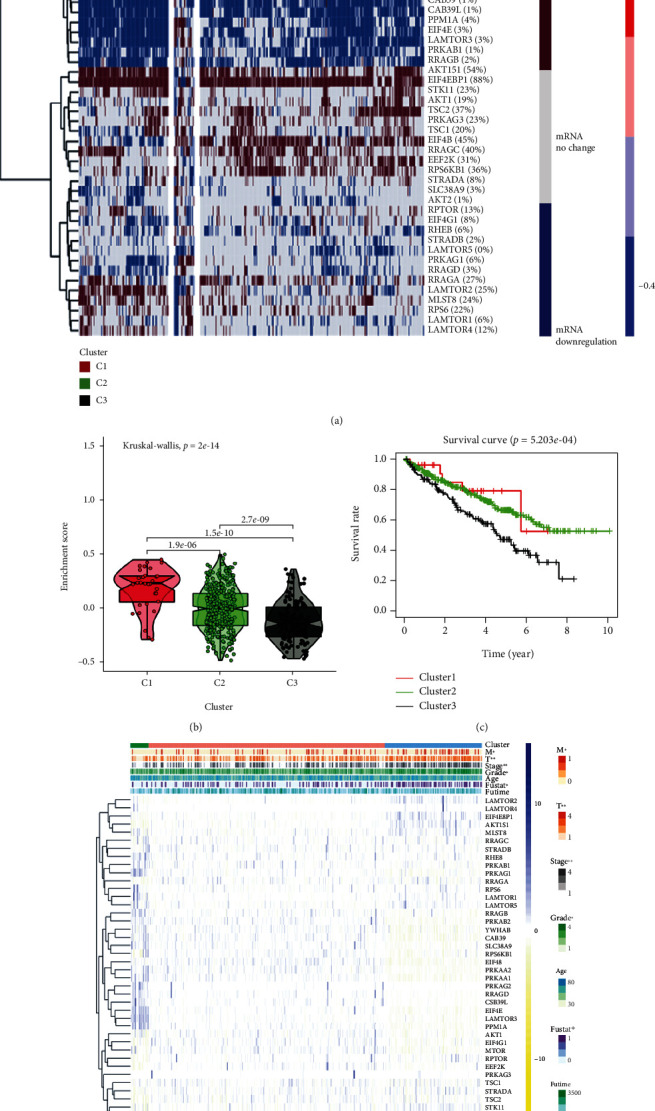
(a) Clustering of gene data from the TCGA database reveals three clusters. Upregulation of mTOR pathway genes was demonstrated in cluster1, and downregulation of mTOR pathway genes was demonstrated in cluster3. In cluster2, mTOR pathway genes were affected the least. The percentage of patients whose genes were altered is provided, showing the high frequency of changes in mTOR pathway genes. (b) The Violin plot shows the enrichment score of the three clusters. The plot shows the enrichment scores of the three clusters, from high to low, as cluster1, then cluster2, followed by cluster3. (c) The survival curves of the three clusters are shown in the plot. Survival in cluster1 is higher than that in cluster2, and survival in cluster2 is higher than that in cluster3. (d) Heat map showing the correlation between mTOR scores and the clinicopathological characteristics of KIRC patients. ^∗^*p* < 0.05, ^∗∗^*p* < 0.01, and ^∗∗∗^*p* < 0.001.

To explore the specific relationship between mTOR and KIRC patients, we constructed an mTOR-score model based on the mRNA expression of the 40 genes. According to the final mTOR-score results, the patient samples were divided into three clusters. cluster1: mTOR-active cluster; cluster2: normal cluster; cluster3: mTOR-inactive cluster (Figure 4(a), Table S6). We can also see the gene enrichment scores of the three clusters through the violin plot: cluster1>cluster2>cluster3 (Figure 4(b)). It is worth mentioning that the quantity of samples in cluster1 was relatively small compared to the other two groups, which will influence the subsequent experiments. After plotting the survival curves of the three clusters, we found that the mTOR-inactive cluster had the lowest survival rate, the second cluster was the normal group, and the mTOR-active cluster had the highest survival rate (Figure 4(c)). This result supports the previously discovered abnormal phenomenon that mTOR pathway-related genes are mostly protective genes in KIRC. We then analyzed the relationship between these three clusters and the clinicopathological characteristics of KIRC patients, and the results showed that T (tumor), M (metastasis), stage, grade, and fustat, were related to the mTOR pathway (Figure 4(d)). The mTOR pathway scores were generally protective. The higher the mTOR score, the lower the grade and stage, and the better the prognosis.

### 3.5. The Relationship between Classic Anticancer Drugs and mTOR

To further explore the drug sensitivity between mTOR clusters, we also conducted a GDSC drug sensitivity analysis. Our research is mainly focused on drugs to treat tumors, especially drugs for targeted therapy of kidney cancer and classical drugs for tumor research, such as metformin. There have been reports that cancer can be targeted with inhibitors of the mTOR pathway [41, 42]. At present, there are many kinds of targeted anticancer drugs, but their mechanisms of action are quite different. Pazopanib, sorafenib, and sunitinib are three drugs that are multitarget kinase inhibitors [43]. Gefitinib inhibits epidermal growth factor receptor (EGFR) [44], bosutinib is a tyrosine kinase inhibitor (TKI) [45], and axitinib inhibits the VEGF pathway [46]. Studies have also found a connection between the mTOR pathway and temsirolimus and metformin [47, 48]. Metformin, an antidiabetic drug, can also be used to prevent cancer alone and in combination with other drugs, mainly by reducing glycemia to cut-off the PI3K/MAPK pathway, which is involved in cell growth, or by activating the AMPK pathway, targeting tumor metabolism angiogenesis, cancer stem cells, and other pathways [49]. Therefore, it is necessary to explore the correlation and mechanism between these targeted drugs and the mTOR pathway.

**Figure 5:**
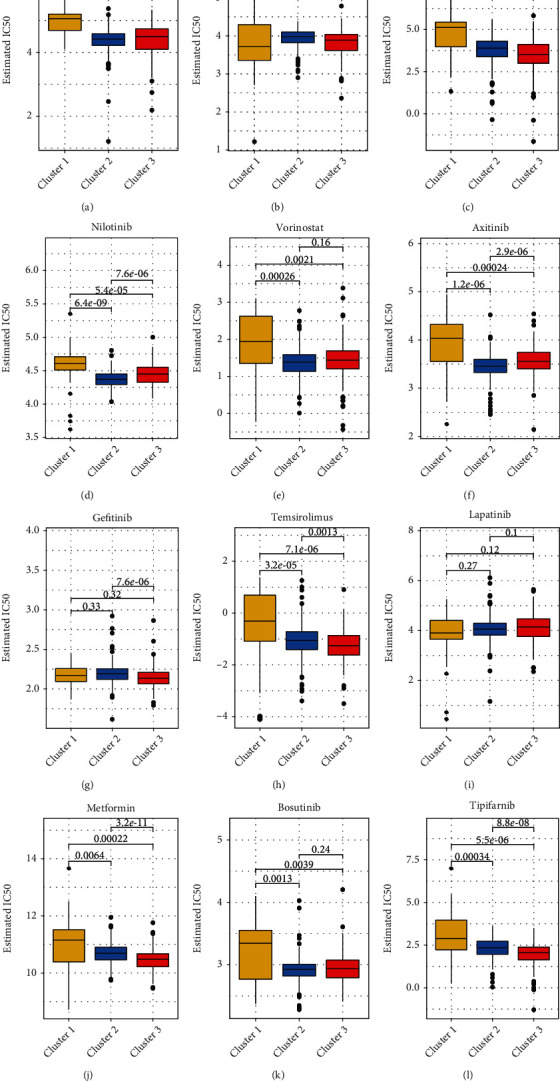
(a–l) The estimated IC50 for 12 types of common chemotherapeutic agents are shown in the plot for cluster1, cluster2, and cluster3. The 12 types of chemotherapeutic agents are pazopanib, sorafenib, sunitinib, nilotinib, vorinostat, axitinib, gefitinib, temsirolimus, lapatinib, metformin, bosutinib, and tipifarnib.

We constructed a ridge regression model to predict the IC50 of drugs (contained in the GDSC) against cancer cells through the cell expression profile of the Genomics of Drug Sensitivity in Cancer (GDSC) database; three clusters were obtained through a cluster analysis. Using this method, we can infer the relationship between these drugs and the mTOR pathway genes (Figure 5). Considering *p* < 0.05 to indicate statistical significance, the cluster-wise significance for each drug was as follows: pazopanib—C1>C3; sorafenib—C2>C3; sunitinib: C1>C3; nilotinib—C1>C3>C2; vorinostat—C1>C3>C2; axitinib—C1>C3>C2; gefitinib—C2>C3; temsirolimus—C1>C2>C3; lapatinib—no significance; metformin—C1>C2>C3; bosutinib—C1>C3; tipipifarnib—C1>C2>C3. C1, C2, and C3 represent cluster1, cluster2, and cluster3. The lower the IC50, the better the drug efficacy. We can understand the therapeutic effects of the drugs in the three clusters through this analysis method to assist in the development of precise cancer treatments in the future.

### 3.6. The mTOR Pathway's Destruction Is Related to the Dysregulation of Several Potential Target Oncogenes and Tumor Suppressor Genes

To further explore the potential regulatory mechanism of the mTOR pathway in KIRC, we studied the relationship between various well-known oncogenes in KIRC and tumor suppressor genes in the three mTOR pathway clusters. We found that the expression of the HRAS, MYC, and VEGFA oncogenes in the inactive group was significantly higher than that in the active group. In comparison, the expression of tumor suppressor genes VHL and PTEN in the inactive group was significantly lower than that in the active group. The above results indicate that the poor prognosis of the inactive group may be related to the abnormal expression of these genes. The expression of oncogenes BRAF, AKT1, KRASM, TOR, and PIK3CA in the active group was significantly higher than that in the inactive group.

In comparison, the expression of the tumor suppressor gene TP53 in the active group was significantly lower than that in the inactive group, indicating that the activation of the mTOR pathway may be closely related to the participation of these genes. Interestingly, we found that the expression of EGFR, MYC, CCND1, CTNNB1, and STAT3 in the normal group was significantly higher than that in the inactive and active groups (Figure 1(a)). This special phenomenon once again illustrates that mTOR plays different roles in different stages of tumor development through the degree of activation. Proper activation of the mTOR pathway increases the expression of oncogenes, and pathway inhibition may cause downregulation of certain oncogenes' expression levels, and at the same time activate other oncogenes through the cross-talk pathway to cause tumor progression. Although the biological mechanisms of these associations may be complicated, the oncogenes mentioned above or tumor suppressor genes may be potential targets for mTOR signaling interruption in KIRC.

Recently, an increasing number of studies have found that sirtuins are involved in various biological processes related to tumorigenesis, such as changes in cancer-related metabolic pathways, uncontrolled proliferation, genome instability, and tumor microenvironment. In human cancers, sirtuins are thought to play complex roles. Depending on the type of cancer and the experimental conditions, they act as both oncogenes and tumor suppressors [50, 51]. The analysis of the transcriptomes of TCGA KIRC patients showed that there is a strong correlation between abnormal sirtuin and HDAC expression levels and the mTOR pathway. A recent study found that the ethanol extract of *Patrinia scabiosaefolia* induces the death of human renal cell carcinoma 786-O cells via SIRT1 and mTOR signaling-mediated metabolic disruptions [52]. SIRT5-mediated SDHA desuccinylation promotes clear cell renal cell carcinoma tumorigenesis [53]. In addition, the SIRT family shows a differentially expressed organization in RCC. Among the seven SIRTs, SIRT1, SIRT3, and SIRT6 can be used as tumor suppressors in KIRC [54]. In our study, the expression of SIRT2, SIRT3, SIRT6, and SIRT7 in the inactive group was significantly higher than that in the active group. In comparison, the expression of SIRT1, SIRT4, and SIRT5 in the inactive group was significantly lower than that in the active group (Figure 1(b)). In summary, these results indicate that sirtuins and mTOR signaling pathways may act synergistically to promote or inhibit multiple processes in the progression of KIRC. Histone deacetylases (HDACs) catalyze the removal of acetyl groups from lysine residues on histones and nonhistone proteins and play a vital role in regulating gene transcription [55]. Deacetylation of histone tails induces chromatin condensation and allows DNA to bind more tightly to the histone core, preventing the transcription mechanism from reaching the promoter region, thereby inhibiting transcription [56]. In this study, we found that the expression of HDAC8, HDAC9, and HDAC11 in the active group was significantly higher than that in the inactive group. In comparison, the expression of HDAC1, HDAC6, HDAC7, and HDAC10 in the active group was significantly lower than that in the inactive group (Figure 1(c)). At present, SIRT and HDAC inhibitors provide new prospects for tumor treatment, and our research results can further offer new directions for future tumor precision treatment. For example, HDAC10 was almost not expressed in the active group, but was abnormally high in the inactive group. Therefore, the use of HDAC10 inhibitors may be more beneficial to patients with inactivation of the mTOR pathway (Figure 1(c)).

### 3.7. mTOR Pathway Implication in Immune Cell Infiltration and in Immune Checkpoints Targeting Cancer Therapy

The tumor microenvironment (TME) is a mixture of fluid, stromal cells, immune cells, extracellular matrix molecules, and various cytokines and chemokines. The cells and molecules in the TME are dynamic in promoting tumor immune escape, tumor growth, and metastasis [57, 58]. As a major regulator of metabolism, mTOR signaling controls immune cell biology in a cell type-specific manner. In addition, mTOR activity needs to be adjusted to maintain proper immune function [59]. To further study the relationship between the mTOR pathway in KIRC and patient immunity, we first performed a correlation analysis between the mTOR pathway and immune cell infiltration. We found that many genes related to the mTOR signaling pathway are associated with the infiltration of multiple immune cells, especially RRAGC, LAmTOR2, EIF4EBP1, PRKAB1, PRKAB2, and other genes, among which RRAGC, LAmTOR2, and EIF4EBP1 were positively correlated with immune cell infiltration.

In contrast, PRKAB1 and PRKAB2 were negatively correlated with immune cell infiltration (Figure 1(d)). We further used the “ggstatsplot package” in R to analyze the relationship between the mTOR pathway score and immune cell infiltration. The results showed that the mTOR score was negatively correlated with various immune cell infiltrations, such as T cell costimulation, parainflammation, Tfh, TIL, and inflammation promotion (Figures 1(e)–1(j)). Immune checkpoint blocking antibodies, including anti-CTLA-4 and anti-PD-1, can induce tumor responses in a variety of tumor types, including melanoma, non-small-cell lung cancer (NSCLC), and kidney renal clear cell carcinoma (KIRC) [60]. In addition, the therapeutic effect of immune checkpoints may be related to the expression of CTLA-4 and PD-1. In the correlation analysis, we found that the mTOR pathway score is negatively correlated with CTLA-4 and PD-1 (Figure 1(k)), so it is potentially inferred that patients with mTOR pathway inactivation may have higher expression of CTLA-4 and PD-1 than patients with mTOR pathway activation. In addition to the TIDE prediction, we also used subclass mapping to compare the expression profiles of the two subtypes (cluster1+cluster2 and cluster3) that responded to immunotherapies [23]. We were delighted to see that the mTOR-inactive cluster was more responsive to anti-PD-1 therapy (Figure 1(l)). Unfortunately, following a Bonferroni correction of the results, the difference between the two groups was not statistically significant (Figure 1(l)).

### 3.8. LASSO Regression to Establish the Prediction Model

**Figure 6:**
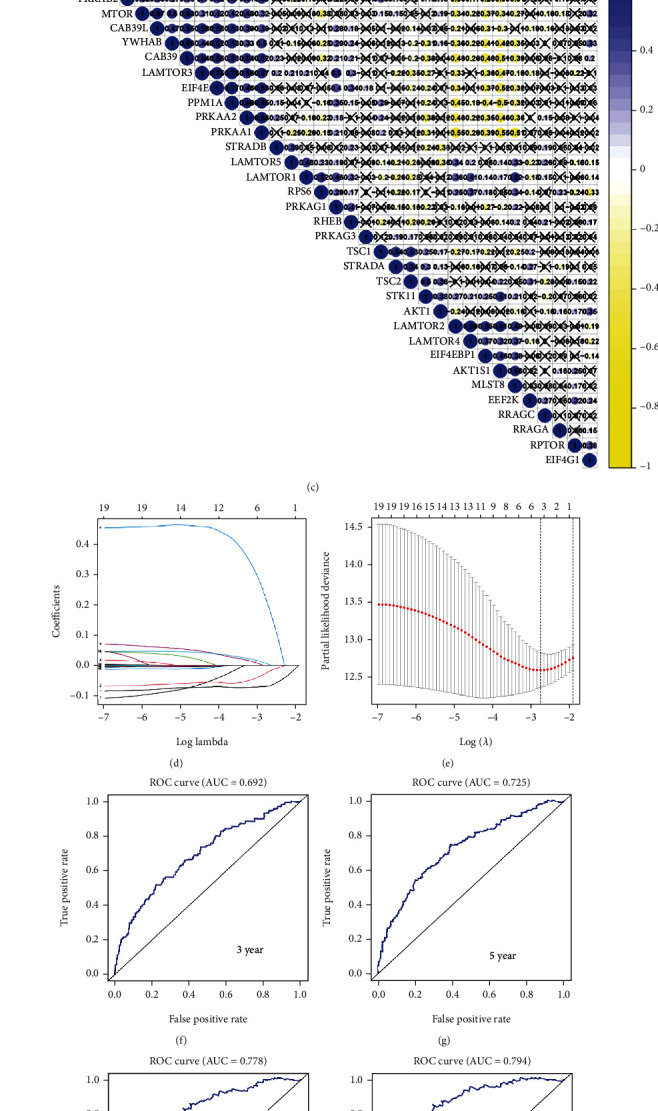
(a) The expression of 40 mTOR pathway genes in KIRC patients. In the color bar on the right side, blue represents upregulation and yellow represents downregulation. N (green) is the normal sample, T (red) is the tumor sample. ^∗^*p* < 0.05, ^∗∗^*p* < 0.01, ^∗∗∗^*p* < 0.001. (b) The plot shows the hazard ratio (HR) analysis with 95% confidence intervals (CI) and *p* values for the mTOR pathway genes. (c) The plot shows the result of the coexpression analysis of 40 mTOR pathway genes. Many of them were correlated in KIRC tissues. (d) The LASSO coefficient profiles of mTOR pathway genes in KIRC. (e) Five genes were selected by LASSO Cox regression analysis. (j) The survival curve was obtained based on this model. Blue and yellow correspond, respectively, to the high-risk group and the low-risk group. (f–i) ROC curves of 3, 5, 7, and 10 years; the AUCs of the curves are 0.692, 0.725, 0.778, and 0.794, respectively. (k) The correlation of five selected genes and the clinicopathological characteristics in two groups. The color bar shows the expression of the genes. Blue represents upregulation, and yellow represents downregulation. ^∗^*p* < 0.05, ^∗∗^*p* < 0.01, ^∗∗∗^*p* < 0.001.

We found that 36 of 40 mTOR pathway genes were differentially expressed by analyzing gene expression in 72 normal tissue samples and 539 KIRC cancer tissues (Figure 6(a), Table S7). We performed a hazard ratio analysis to show the relationship between these gene pathways and the progression of KIRC (Figure 6(b), Table S5). A coexpression analysis was used to analyze the relationship between gene pathways, and the results showed that there were coexpression relationships among these genes (Figure 6(c)). To explore the possibility of using the mTOR pathway genes to build a model to predict KIRC patient prognosis, we conducted a LASSO regression analysis (Figures 6(d) and 6(e)) to establish the model. We selected five genes as risk factors: PRKAA2, AmTOR3, STRADA, EIF4EBP1, and RHEB. We used this model to divide the sample into two groups: high-risk and low-risk groups based on the best cut-off values of the risk scores. We plotted the survival curves of the two groups, which showed that the low-risk group predicted better survival than the high-risk group (Figure 6(j)). A receiver operating characteristic (ROC) curve analysis was then performed to analyze the predictive prognostic performance of the new survival model in KIRC patients. The 3-year survival had an area under the curve (AUC) of 0.692; 5-year survival, AUC = 0.725; 7-year survival, AUC = 0.778; and 10-year survival, AUC = 0.794 (Figures 6(f)–6(i)). Generally, an AUC value greater than 0.7 is considered predictive. We then used heat maps to demonstrate the correlation between the model and the pathological features of renal cell carcinoma. The results show that M, T, stage, grade, and fustat were related to the model we established (Figure 6(k)).

### 3.9. Validation of the Model and Representation Using a Nomogram

**Figure 7:**
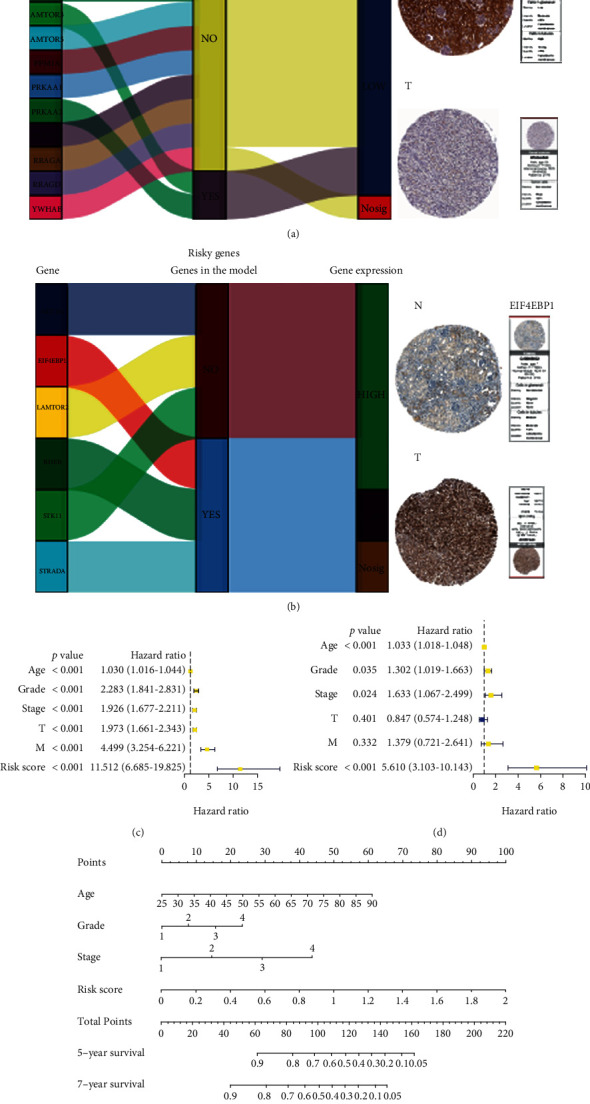
(a, b) Sankey diagrams were plotted for two types of genes, risky and protective. Immunohistochemical images were obtained from the HPA website for PRKAA2 and EIF4EBP1, which are representative of the two gene groups. (c) Univariate Cox analysis. (d) Multivariate Cox analysis. (e) Nomogram of the model.

We selected genes that were statistically significant in the previous HR analyses. They were divided into two groups according to risky genes and protective genes. After the mulberry diagram was drawn to classify them, we found that both AmTOR3 and PRKAA2, which are protective genes, were in the model and showed low expression levels in KIRC tissues, while EIF4EBP1, a risky gene, was highly expressed in KIRC tissues. In addition, we obtained the immunohistochemical information of PRKAA2 and EIF4EBP1 from the human protein atlas (HPA) website [61] and verified the results of their gene expression at the protein level (Figures 7(a) and 7(b)). In addition, the results of immunohistochemistry experiments on the two molecules PRKAA2 and EIF4EBP1 on our KIRC clinical specimens are also consistent with the above results, and the corresponding results are shown in Supplementary Materials Figure S1. We then performed univariate and multivariate Cox regression analyses and found that the risk model was a risk factor in both regression analyses and was prominent in the multivariate Cox regression model (Table S8 and Table S9) (Figures 7(c) and 7(d)). Finally, we used a nomogram to predict the risk and prognosis of patients with KIRC. The nomogram generated a total of nine rows (Figure 7(e)). The second to the ninth rows were age, grade, stage, risk score, total points, 5-year survival, 7-year survival, and 10-year survival. From the second to the fifth lines, the patient scores were found and added together to obtain the total scores, corresponding to 5-, 7-, and 10-year survival.

## 4. Discussion

Rapamycin was first identified by Sehgel in 1964 [62], and its two important target genes TOR1 and TOR2 were identified in 1991 [63]. The mTOR protein was identified three years later as a direct target of the rapamycin complex [64]. In recent years, the mechanism of the mTOR pathway in the human body has been gradually explored. It regulates various cellular processes, including protein synthesis, growth, metabolism, senescence, regeneration, and autophagy. At present, the mTOR gene has been found to play a role in a variety of diseases [65], such as neurological diseases [66–70], tumors [39, 71–74], and diabetes [75, 76].

mTOR contains two complexes, mTORC1 and mTORC2. mTORC1 promotes protein synthesis by phosphorylation of two key effectors, S6K1 and 4EBP, and inhibits protein decomposition by blocking AMPK activation of ULK1, thereby controlling cell growth and division. The most important role of mTORC2 may be to activate AKT to promote cell survival [39]. Both play important roles in the occurrence and development of cancer. mTORC1 regulates mutations in many oncogenic pathways, such as the PI3K/AKT pathway and the Ras/Raf/Mek/Erk (MAPK) pathway. Simultaneously, mTORC2 also affects cancer by activating AKT, which promotes proliferation and suppresses apoptosis [15]. Drugs targeting mTOR have been developed based on the above mechanism. First-generation rapamycin and rapalog mainly downregulate the activation of mTORC1 to S6K1 by inhibiting mTORC1 to reduce the growth and proliferation of cancer cells. However, mTORC1 achieves a carcinogenic effect by inhibiting 4EBP, so the effects from rapalogs are not ideal. Following this, second-generation mTOR kinase inhibitors that simultaneously act on mTORC1 and mTORC2 have also been developed. These can compete with mTOR catalytic sites for ATP and selectively inhibit mTORC1 and mTORC2 [16, 39]. At present, there have been third-generation mTOR inhibitors targeting more mTOR molecule binding sites, aiming at reducing tumor resistance through stronger binding with mTOR molecules [77].

In cancer, the mechanism of the mTOR pathway is more complicated. The conclusions are as follows: (1) The upstream signaling pathway overactivates the mTOR pathway [78]. (2) The expression of the mTOR gene is modified or regulated by miRNAs [79]. (3) The mTOR pathway gene regulates the human immune system and causes the immune escape of tumor cells [80, 81]. Based on the current research results, we first chose to study the expression level of mTOR pathway genes in cancer and their differential expression levels. We then focused on some potential compounds that may target the mTOR pathway, laying a foundation for future studies. We then compared the mTOR pathway genes in normal and cancer tissues to determine whether they exist as risky or protective genes. In our results, we found an interesting phenomenon: compared with other cancer types, the mTOR pathway gene in KIRC mostly exists as a protective gene, which is inconsistent with previous research results that mTOR pathway overexpression can lead to the occurrence of cancer. Therefore, we turned our attention to KIRC.

KIRC samples were divided into three clusters according to their mTOR scores, which were based on their mRNA expression levels. The three clusters represented three different gene expression states in the mTOR pathway for the convenience of subsequent experiments. After plotting the survival curves for the three clusters, we found that the mTOR pathway gene expresses inactive clustering survival rates, confirming our previous findings that mTOR is protective in KIRC. In fact, we also found relevant research reports pertaining to this abnormal mTOR action. Zhong et al. reported in the literature in October 2020 that mTOR pathway-related genes were enriched in their low-risk group of KIRC samples [82]. However, the reason for this phenomenon remains unclear. Based on the mechanism of mTOR and previous studies on the mechanism of mTOR in KIRC [83], we found that the activation of the mTOR pathway in KIRC is still the cause of cancer development; however, our results contradict the conclusion that mutations in the mTOR pathway promote cancer progression. We studied the mTOR pathway gene survival landscape in patients with KIRC and found that more than half of the statistically significant mTOR pathway genes have a protective effect in KIRC.

There is a well-established system for targeted therapies for KIRCs. The efficacy of targeted mTOR pathway therapies for cancer is currently well established. The first of the aforementioned three generations of mTOR inhibitors, everolimus, is still widely used in the treatment of patients with advanced renal cell carcinoma after the failure of sunitinib or sorafenib. Therefore, we used CMap to look for potential drugs to treat KIRC and performed a GDSC analysis to confirm the effects of some of the most common targeted mTOR pathway gene drugs in KIRC therapy. We hope that these analyses will help in the clinical treatment of KIRC. The results showed that most targeted drug therapies for KIRC are related to the impact of mTOR pathway gene expression levels. These results could provide new insights into the development of targeted drugs to treat KIRC in the future, especially those that target the mTOR pathway.

Currently, research on immunotherapy for cancer is very popular. The treatment of histone acetylation [84, 85] and the enhancement of T cell-killing effects are relatively accepted concrete means. By observing the expression of some classical protooncogenes, tumor suppressor genes (KRAS, VHL, and so on), and immune-related genes, especially histone acetylation, in the three clusters, we found the effects of these genes in the mTOR pathway, and most of the genes were positively or negatively correlated with the mTOR pathway. However, the expression of some genes in cluster1 (mTOR active) and cluster3 (mTOR inactive) was consistent and contrary to that in cluster2 (normal). Among them, we found an interesting phenomenon: for example, mTOR pathway upregulation or downregulation were both related to low expression levels of MYC and EGFR. Meanwhile, HDAC8 exhibited the opposite: mTOR pathway upregulation or downregulation were both related to its high expression. For this nonlinear relationship, we speculate that there are still undiscovered intermediate pathways between the expression of the mTOR pathway and the expression of such genes, which need to be further elucidated.

We observed a correlation between many immune-infiltration-related factors and mTOR pathway genes. The results showed that almost all immune-infiltration-related factors were negatively correlated with mTOR. This indicates that the activation of the mTOR pathway suppresses immune infiltration of the body. Therefore, from the perspective of immunity, the mTOR pathway is still a cancer pathway, which cannot support the previous conclusions obtained by studying the mTOR-score-related survival curve in KIRC. These results indicate that the mechanism of the mTOR pathway is not fully understood.

Immunotherapy for T cells has been the main treatment method for KIRC, with PD-1 and CTLA-4 as the research focus [25]. Inhibition of PD-1 and CTLA-4 increases T cell killing. In our study of three mTOR-score clusters' responsiveness to PD-1 and CTLA-4 inhibitor targets, we combined cluster1 and cluster2 as mTOR-active clusters because of the small size of cluster1, and we defined cluster3 as an mTOR-inactive cluster. We were delighted to see that the mTOR-inactive cluster was more likely to be responsive to anti-PD-1 therapy. Unfortunately, following a Bonferroni correction, the results were not statistically significant.

We then analyzed the differential expression of these 40 mTOR pathway genes in cancer and normal tissues and performed an HR analysis and coexpression analysis of these genes. Subsequently, five genes in the mTOR pathway genes were screened using a LASSO regression to construct a model to predict the survival rate of KIRC patients. We hope that this prediction model will provide some help for future clinical studies.

## Data Availability

The data used to support the findings of this study are available from the corresponding authors upon request.

## Abbreviations

mTOR: Mammalian target of rapamycin
KIRC: Kidney renal clear cell carcinoma
RCC: Renal cell carcinoma
THCA: Thyroid carcinoma
THYM: Thymoma
PRAD: Prostate adenocarcinoma
UCEC: Uterine corpus endometrial carcinoma
SKCM: Skin cutaneous melanoma
COAD: Colon adenocarcinoma
TCGA: The Cancer Genome Atlas
GDSC: Genomics of Drug Sensitivity in Cancer
HPA: The Human Protein Atlas
LASSO: Least absolute shrinkage and selection operator
GSEA: Gene set enrichment analysis
CMap: Connectivity map
CNV: Copy number variation
SNV: Single-nucleotide variation
WNT-*β*-catenin: Wingless/integrated-*β*-catenin
TP53: Tumor protein 53
VEGF: Vascular endothelial growth factor
CAB39L: Calcium-binding protein 39-like
PRKAA2: AMPK alpha 2 subunit alpha 2
PRKAB1: AMPK alpha 2 subunit beta 1
PRKAB2: AMPK alpha 2 subunit beta 2
TME: The tumor microenvironment
LAmTOR2: Late endosomal and lysosomal adaptor and MAPK (mitogen-activated protein kinase) and mTOR (mechanistic target of rapamycin) activator complex
EIF4EBP1: 4E binding protein 1
CTLA-4: Cytotoxic T-lymphocyte-associated protein 4
PD-1: Programmed cell death protein 1
HMGCR: Recombinant 3-hydroxy-3-methylglutaryl coenzyme a reductase
TTNPB: 4-[(1E)-2-(5,5,8,8-Tetramethyl-5,6,7,8-tetrahydro-2-naphthalenyl)-1-propen-1-yl] benzoic acid
HRAS: HRas protooncogene
MYC: Myelocytomatosis oncogene
VEGFA: Vascular endothelial growth factor A
VHL: Von Hippel-Lindau
PTEN: Phosphatase and tensin homolog
BRAF: Mutant serine/threonine protein kinase B-Raf
KRASM: KRAS-mutant
TOR: Target of rapamycin
EGFR: Epidermal growth factor receptor
CCND1: CyclinD1
CTNNB1: Catenin beta1
STAT3: Signal transducer and activator of transcription 3
HDAC: Zinc-dependent histone deacetylases
SIRT: Sirtuins
SDHA: Succinate dehydrogenase subunit A.

## Supplementary Materials

Supplementary Materials Figure S1: the results of immunohistochemistry experiments on the two molecules PRKAA2 and EIF4EBP1; Table S1: CNV deletion frequency of mTOR pathway genes across 33 cancer types; Table S2: CNV amplification frequency of mTOR pathway genes across 33 cancer types; Table S3: SNV frequency of mTOR pathway genes in 33 cancer types; Table S4: logFCs of mTOR pathway genes across cancer types; Table S5: HRs of mTOR pathway genes across cancer types; Table S6: cluster and mTOR_score information; Table S7: expression of mTOR gene in KIRC; Table S8: uniCox analysis in KIRC; Table S9: multi-Cox analysis in KIRC.
