# Supplementary material for: Genomic and Transcriptome Analysis to Identify the Role of the mTOR Pathway in Kidney Renal Clear Cell Carcinoma and Its Potential Therapeutic Significance
Source: Oxid Med Cell Longev. 2021 Jun 7;2021:6613151. doi: 10.1155/2021/6613151 (PMC8203410; doi:10.1155/2021/6613151)
Supplement: Supplementary Materials — Figure S1: the results of immunohistochemistry experiments on the two molecules PRKAA2 and EIF4EBP1; Table S1: CNV deletion frequency of mTOR pathway genes across 33 cancer types; Table S2: CNV amplification frequency of mTOR pathway genes across 33 cancer types; Table S3: SNV frequency of mTOR pathway genes in 33 cancer types; Table S4: logFCs of mTOR pathway genes across cancer types; Table S5: HRs of mTOR pathway genes across cancer types; Table S6: cluster and mTOR_score information; Table S7: expression of mTOR gene in KIRC; Table S8: uniCox analysis in KIRC; Table S9: multi-Cox analysis in KIRC. [file 6613151.f1.pdf]

Supplementary Materials Figures and Legends

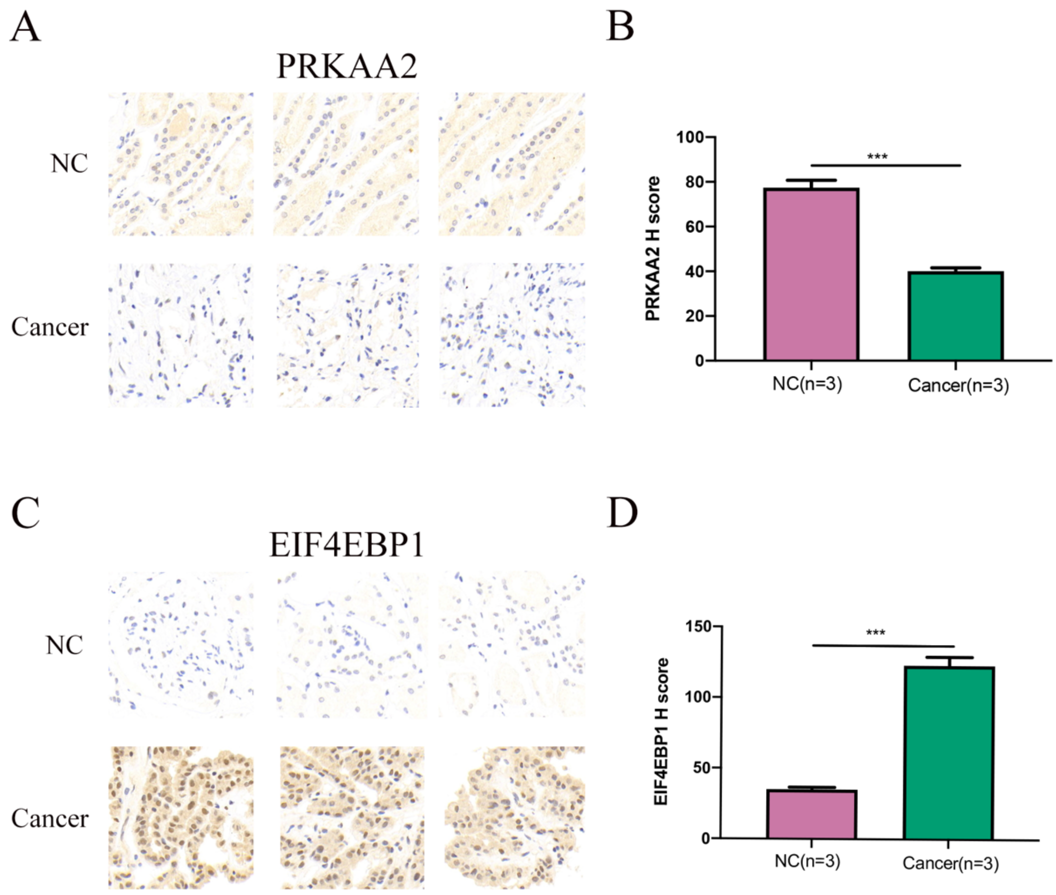

Figure S1: The results of immunohistochemistry experiments on the two molecules PRKAA2 and EIF4EBP1. (A-B) Typical immunohistochemical images of PRKAA2 in kidney cancer tissue and normal kidney tissue, and the corresponding semi-quantitative histogram. (C-D) Typical immunohistochemical images of EIF4EBP1 in kidney cancer tissue and normal kidney tissue, and the corresponding semi-quantitative histogram. ***p <0.001.

Table S1: CNV deletion frequency of mTOR pathway genes across 33 cancer types

	ACC	BLCA	BRCA	CESC	CHOL	COAD	DLBC	ESCA
AKT1	0.06667	0.07971	0.07498	0.05306	0.42424	0.19238	0	0.0625
AKT1S1	0.02222	0.00966	0.02343	0.0449	0.0303	0.01403	0	0.04167
AKT2	0.03333	0.01208	0.02999	0.01633	0	0.01202	0	0.03125
CAB39	0.06667	0.21498	0.05904	0.26531	0	0.02405	0	0.125
CAB39L	0.27778	0.16184	0.23524	0.22041	0.21212	0.02204	0.06897	0.08333
EEF2K	0.01111	0.05072	0.01125	0.02857	0	0.00802	0	0.05208
EIF4B	0.01111	0.02899	0.02812	0.00408	0	0	0	0.02083
EIF4E	0.03333	0.04348	0.06186	0.10204	0.18182	0.05812	0.06897	0.11458
EIF4EBP1	0.11111	0.157	0.10965	0.11837	0.06061	0.1483	0.17241	0.14583
EIF4G1	0.1	0	0.01312	0	0.06061	0.00601	0	0
LAMTOR1	0.14444	0.03623	0.0956	0.10612	0.0303	0.03407	0	0.08333
LAMTOR2	0.1	0	0.00187	0	0	0	0	0
LAMTOR3	0.03333	0.04348	0.06092	0.10204	0.18182	0.06212	0.06897	0.11458
LAMTOR4	0.05556	0.00966	0.04311	0.05306	0	0.00601	0	0.01042
LAMTOR5	0.22222	0.00725	0.08997	0.0449	0.12121	0.08417	0.06897	0.15625
MLST8	0.02222	0.1401	0.01312	0.0449	0.0303	0.01804	0	0.05208
MTOR	0.28889	0.01691	0.14714	0.03673	0.57576	0.12224	0.03448	0.13542
PPM1A	0.05556	0.07005	0.0806	0.01633	0.30303	0.18036	0	0.05208
PRKAA1	0.01111	0.00483	0.02437	0.00408	0	0.02004	0	0.02083
PRKAA2	0.23333	0.01208	0.07123	0.02041	0.18182	0.08417	0.03448	0.09375
PRKAB1	0	0.02174	0.04592	0.00816	0.09091	0.01002	0	0.01042
PRKAB2	0.1	0	0.00094	0.00408	0	0.002	0	0
PRKAG1	0	0.0314	0.02718	0.00408	0	0	0.03448	0.03125
PRKAG2	0.06667	0.02899	0.06373	0.11837	0.0303	0.01403	0.03448	0.10417
PRKAG3	0.05556	0.17633	0.04217	0.22449	0	0.00601	0.03448	0.09375
RHEB	0.05556	0.02174	0.05998	0.1102	0.0303	0.00601	0.03448	0.09375
RPS6	0.18889	0.23188	0.10309	0.06939	0.24242	0.03808	0.03448	0.19792
RPS6KB1	0.12222	0.00242	0.0253	0.00816	0	0.03206	0	0.01042
RPTOR	0.12222	0.01449	0.04686	0.0898	0	0.04609	0	0.02083
RRAGA	0.18889	0.22705	0.1059	0.06531	0.24242	0.03808	0.03448	0.19792
RRAGB	0.01111	0.01691	0.0075	0.02857	0	0.01403	0	0.07292
RRAGC	0.18889	0.01208	0.05436	0.00408	0.15152	0.05812	0.03448	0.09375
RRAGD	0.08889	0.09903	0.16214	0.12245	0.45455	0.03607	0.2069	0.04167
SLC38A9	0	0.12319	0.11621	0.1102	0	0.05411	0	0.19792
STK11	0.02222	0.0942	0.1059	0.21224	0	0.04208	0	0.10417
STRADA	0.13333	0.00725	0.02156	0.01224	0	0.02806	0	0.03125
STRADB	0.05556	0.11353	0.03187	0.15918	0	0.00601	0.03448	0.04167
TSC1	0.01111	0.18116	0.06842	0.02041	0.24242	0.02004	0	0.0625
TSC2	0.02222	0.13285	0.01312	0.04898	0.0303	0.01804	0	0.05208
YWHAB	0	0	0.01593	0.02857	0.0303	0.00401	0	0

GBM	HNSC	KICH	KIRC	KIRP	LGG	LIHC	LUAD	LUSC
0.16242	0.07759	0.04545	0.12733	0.10526	0.11069	0.17414	0.05019	0.03817
0.07803	0.05172	0	0.0034	0.01645	0.4015	0.03166	0.05212	0.05916
0.0414	0.05172	0.01515	0.0034	0.01316	0.3546	0.01847	0.02124	0.03817
0.00478	0.09483	0.36364	0.04244	0.04276	0.0469	0.02902	0.01158	0.06679
0.2707	0.09483	0.4697	0.02547	0.03618	0.13133	0.30607	0.08494	0.12595
0.05573	0.00862	0.01515	0	0	0.00938	0.11609	0.00772	0.03626
0.04777	0	0	0	0	0.0394	0.00792	0.00965	0.00382
0.02548	0.09483	0	0.01528	0.04276	0.06191	0.24538	0.02896	0.07252
0.03662	0.06897	0.16667	0.08829	0.05263	0.01313	0.44855	0.12162	0.05916
0.00478	0	0.04545	0.03226	0.00329	0.01313	0.00792	0.02896	0
0.04299	0.09483	0.09091	0.00679	0.01645	0.0075	0.03694	0.00965	0.03435
0	0.00862	0.57576	0.02037	0.03289	0	0	0	0
0.02548	0.09483	0	0.01698	0.04276	0.06191	0.24802	0.0251	0.07252
0.00159	0.00862	0	0	0	0.00375	0.02639	0.00965	0.00954
0.01592	0.09483	0.63636	0.04244	0.07895	0.32458	0.08443	0.06371	0.08588
0.02229	0.03448	0.01515	0	0.00329	0	0.14512	0.01351	0.06107
0.07643	0.09483	0.66667	0.06282	0.11513	0.32458	0.22955	0.03089	0.09351
0.19108	0.05172	0.01515	0.13413	0.09211	0.10882	0.15831	0.03282	0.05153
0.01115	0	0.09091	0.0017	0.00658	0.02064	0	0.00772	0.00954
0.01274	0.05172	0.71212	0.05263	0.07566	0.32458	0.09763	0.01931	0.04771
0.0207	0.00862	0	0.0017	0	0.02627	0.0343	0.01544	0.0229
0.00159	0	0.56061	0.02037	0.03618	0.0075	0.00264	0	0
0.06529	0.00862	0	0	0	0.04503	0.00792	0.00579	0.00382
0.00478	0.10345	0	0.0034	0.00329	0	0.04485	0.02124	0.04389
0	0.11207	0.39394	0.03396	0.02961	0.01501	0.02639	0.01158	0.0687
0.00159	0.10345	0	0.0034	0.00329	0	0.04222	0.00965	0.03435
0.47293	0.24138	0.07576	0.05263	0.07895	0.17261	0.14248	0.12934	0.15267
0.00478	0	0.57576	0.01019	0.00329	0.00375	0	0.00579	0.00954
0.01752	0.02586	0.56061	0.01528	0.00329	0.00938	0.00792	0.00965	0.01718
0.4793	0.24138	0.07576	0.05263	0.08224	0.16698	0.14248	0.12741	0.15076
0.00318	0.03448	0	0.00509	0.01316	0.00188	0.05277	0.02317	0.0229
0.00637	0.0431	0.60606	0.04924	0.09211	0.31707	0.13456	0.00579	0.06107
0.12261	0.02586	0.68182	0.08149	0.04934	0.05816	0.18997	0.14286	0.02099
0.0207	0.16379	0.09091	0.0017	0.01645	0.01876	0.01055	0.05019	0.14122
0.01274	0.09483	0	0.00849	0.01316	0.00563	0.09763	0.11969	0.07443
0.00318	0	0.57576	0.01019	0.00329	0.00375	0	0.00772	0.00763
0	0.0431	0.33333	0.01528	0.01645	0.0075	0.01055	0.00193	0.02672
0.07643	0.01724	0.06061	0.05433	0.07895	0.02251	0.09763	0.0444	0.05916
0.02229	0.03448	0.01515	0	0.00329	0	0.14512	0.01351	0.06107
0.00478	0.03448	0	0	0	0	0.01055	0.02896	0.01718

MESO	OV	PAAD	PCPG	PRAD	READ	SARC	SKCM	STAD
0.17442	0.16852	0.02013	0.07333	0.01014	0.21687	0.03333	0.12076	0.04299
0.04651	0.27027	0	0.00667	0.00609	0.08434	0.14167	0.02331	0.02036
0.01163	0.17647	0	0.00667	0.01014	0.03614	0.05833	0.02754	0.02489
0.03488	0.11288	0	0.02	0.01014	0	0.24167	0.0678	0.00905
0.12791	0.39905	0.02013	0.02667	0.24138	0.01205	0.43333	0.06568	0.01131
0.01163	0.1876	0	0.02	0.00203	0	0.09167	0.02754	0.02715
0.02326	0.062	0.01342	0.01333	0.00406	0.01205	0.075	0.07415	0.02262
0.12791	0.46582	0.01342	0	0.01014	0.13253	0.1	0.06992	0.04072
0.02326	0.23688	0.04698	0.10667	0.18053	0.19277	0.10833	0.11864	0.07014
0.04651	0.00795	0.01342	0.46667	0.01826	0	0.05	0.05508	0
0	0.03975	0	0.15333	0	0.03614	0.04167	0.11441	0.01584
0	0.00318	0	0.02667	0.01014	0	0	0	0.00226
0.11628	0.47695	0.01342	0	0.01014	0.13253	0.09167	0.0678	0.03846
0	0.03657	0.00671	0.05333	0.00811	0	0.04167	0.01907	0
0.19767	0.08267	0.05369	0.66667	0.0284	0.13253	0.125	0.07415	0.03394
0	0.2814	0.02013	0.03333	0.00203	0.01205	0.08333	0.03178	0.05204
0.15116	0.17647	0.06711	0.52	0.02434	0.19277	0.08333	0.0911	0.03394
0.13953	0.24801	0.00671	0.06667	0.01014	0.18072	0.05	0.11653	0.02262
0	0.03816	0	0.01333	0.00406	0.03614	0.01667	0.0572	0.00905
0.04651	0.07154	0.04698	0.63333	0.03651	0.09639	0.05833	0.0339	0.01131
0.01163	0.16216	0.01342	0.03333	0.01014	0.0241	0.01667	0.06992	0.02941
0.01163	0.00318	0	0.04	0	0	0.00833	0	0
0.02326	0.06995	0.02013	0.04	0.00406	0	0.05	0.05932	0.01584
0.01163	0.04452	0.02685	0.06667	0.03043	0.01205	0.175	0.02119	0.05204
0.04651	0.09221	0.00671	0.01333	0.01014	0	0.11667	0.03602	0.00679
0.01163	0.04134	0.01342	0.06	0.02231	0	0.175	0.02119	0.05204
0.11628	0.26232	0.12081	0.02667	0.01217	0.09639	0.2	0.36864	0.11312
0.01163	0.2194	0.06711	0.04	0.01826	0.03614	0.05833	0.02542	0.00679
0.03488	0.15421	0.0604	0.04	0.0142	0.04819	0.03333	0.02966	0.02036
0.11628	0.2655	0.12081	0.02667	0.02028	0.07229	0.21667	0.37076	0.11312
0	0.0461	0.01342	0	0.00609	0.0241	0	0.01059	0.01357
0.06977	0.04134	0.04698	0.52667	0.00406	0.10843	0.09167	0.04025	0.01584
0.16279	0.27345	0.12752	0.13333	0.21907	0.07229	0.125	0.27754	0.0543
0.02326	0.3911	0.02685	0.02667	0.1217	0.13253	0.03333	0.09746	0.08597
0.04651	0.58506	0.01342	0	0.00811	0.07229	0.11667	0.06568	0.07466
0.01163	0.21145	0.0604	0.04	0.00811	0.0241	0.03333	0.01907	0.00905
0	0.05087	0	0.01333	0.00811	0	0.08333	0.03178	0.01357
0.0814	0.33545	0.02685	0.03333	0.00406	0.04819	0.125	0.2161	0.01357
0.01163	0.28458	0.02013	0.03333	0.00203	0.01205	0.09167	0.03178	0.04977
0	0.03021	0	0.01333	0.01217	0	0.03333	0.00636	0

TGCT	THCA	THYM	UCEC	UCS	UVM
0	0.00196	0	0.03461	0.14286	0.05
0.01282	0	0	0.04007	0.17857	0.0125
0.01282	0	0	0.03279	0.07143	0.0125
0.00641	0.00587	0	0.02004	0.01786	0.05
0.04487	0.03718	0.03333	0.09654	0.25	0.0375
0.00641	0.00196	0	0.04736	0.125	0
0	0	0	0.00546	0.08929	0.05
0.0641	0	0	0.07286	0.375	0.0625
0.00641	0	0	0.08561	0.25	0.225
0	0.00587	0	0.00546	0.03571	0.5125
0.08974	0.00978	0	0.03279	0.19643	0.0125
0	0	0	0	0	0
0.0641	0	0	0.06922	0.375	0.0625
0	0.00196	0	0.02004	0.01786	0
0.01923	0.00391	0	0.02186	0.01786	0.2875
0.00641	0.00196	0	0.07104	0.16071	0
0.00641	0.00391	0.03333	0.04918	0.14286	0.3125
0.00641	0	0.03333	0.05829	0.19643	0.025
0.02564	0	0	0.01821	0.01786	0.0125
0.00641	0.00391	0	0.00911	0.03571	0.2875
0	0	0	0.03097	0.10714	0
0	0	0	0	0	0
0	0	0	0.00546	0.16071	0.05
0.00641	0	0	0.04736	0.125	0.0125
0	0.00587	0	0.02004	0	0.0125
0.00641	0	0	0.04918	0.10714	0.0125
0.03205	0.0137	0.1	0.06011	0.26786	0.0375
0	0	0	0.05829	0.03571	0
0	0.00587	0.03333	0.04554	0.05357	0
0.03205	0.0137	0.1	0.06011	0.26786	0.0375
0	0.00196	0	0.01093	0.01786	0
0.00641	0.00391	0	0.00911	0.01786	0.3375
0.00641	0.00587	0.03333	0.01275	0.05357	0.325
0.03846	0	0	0.10747	0.32143	0.05
0.01282	0.01566	0.03333	0.18761	0.60714	0.025
0	0	0	0.06011	0.03571	0
0	0.00391	0	0.00729	0	0.0125
0.00641	0.03914	0.03333	0.12022	0.41071	0.0375
0.00641	0.00196	0	0.07286	0.21429	0
0	0	0	0.01093	0.03571	0

Table S2: CNV amplification frequency of mTOR pathway genes across 33 cancer types

	ACC	BLCA	BRCA	CESC	CHOL	COAD	DLBC	ESCA
AKT1	0.166667	0.050725	0.062793	0.110204	0	0.012024	0.034483	0.135417
AKT1S1	0.455556	0.142512	0.061856	0.155102	0.060606	0.066132	0.034483	0.052083
AKT2	0.433333	0.18599	0.075914	0.183673	0.090909	0.076152	0	0.03125
CAB39	0.066667	0	0.009372	0.008163	0.090909	0.026052	0	0
CAB39L	0.044444	0.048309	0.015933	0.008163	0	0.442886	0.034483	0.020833
EEF2K	0.411111	0.036232	0.292409	0.061224	0.060606	0.09018	0.034483	0.03125
EIF4B	0.611111	0.041063	0.044986	0.040816	0.090909	0.094188	0.034483	0.020833
EIF4E	0.266667	0.016908	0.033739	0.028571	0	0.008016	0	0.03125
EIF4EBP1	0.288889	0.166667	0.276476	0.061224	0.030303	0.196393	0.034483	0.25
EIF4G1	0.111111	0.219807	0.143393	0.677551	0.090909	0.072144	0.137931	0.708333
LAMTOR1	0.044444	0.128019	0.109653	0.069388	0	0.018036	0.034483	0.354167
LAMTOR2	0.077778	0.246377	0.56045	0.326531	0.484848	0.104208	0.206897	0.197917
LAMTOR3	0.266667	0.016908	0.032802	0.028571	0	0.006012	0	0.03125
LAMTOR4	0.355556	0.086957	0.088097	0.081633	0.060606	0.306613	0.137931	0.229167
LAMTOR5	0.011111	0.031401	0.039363	0.089796	0.151515	0.002004	0	0.020833
MLST8	0.4	0.012077	0.302718	0.130612	0.060606	0.082164	0.068966	0.020833
MTOR	0.011111	0.057971	0.012184	0.114286	0	0.006012	0	0.020833
PPM1A	0.133333	0.038647	0.041237	0.102041	0.030303	0.014028	0.068966	0.125
PRKAA1	0.577778	0.280193	0.132146	0.35102	0.121212	0.096192	0.068966	0.4375
PRKAA2	0.011111	0.060386	0.044049	0.146939	0.151515	0.002004	0	0
PRKAB1	0.577778	0.055556	0.043112	0.040816	0.060606	0.084168	0.034483	0.0625
PRKAB2	0.1	0.268116	0.448922	0.269388	0.424242	0.104208	0.137931	0.197917
PRKAG1	0.6	0.031401	0.04686	0.044898	0.090909	0.098196	0.034483	0.03125
PRKAG2	0.4	0.082126	0.079663	0.04898	0.030303	0.270541	0.034483	0.083333
PRKAG3	0.077778	0	0.018744	0.012245	0.060606	0.028056	0	0
RHEB	0.4	0.074879	0.079663	0.04898	0.030303	0.272545	0.034483	0.083333
RPS6	0.122222	0.070048	0.067479	0.102041	0	0.094188	0.103448	0.041667
RPS6KB1	0.066667	0.161836	0.266167	0.097959	0.151515	0.088176	0.034483	0.104167
RPTOR	0.066667	0.15942	0.223055	0.089796	0.121212	0.104208	0.034483	0.104167
RRAGA	0.133333	0.067633	0.066542	0.102041	0	0.094188	0.103448	0.041667
RRAGB	0.044444	0.045894	0.056232	0.036735	0.060606	0.018036	0.034483	0.041667
RRAGC	0	0.055556	0.053421	0.134694	0.090909	0.006012	0	0.072917
RRAGD	0.111111	0.002415	0.056232	0.032653	0.030303	0.018036	0	0.052083
SLC38A9	0.566667	0.031401	0.044986	0.04898	0.060606	0.02004	0.068966	0.020833
STK11	0.444444	0.021739	0.014058	0.028571	0.030303	0.028056	0.068966	0.03125
STRADA	0.1	0.181159	0.247423	0.114286	0.151515	0.102204	0.034483	0.09375
STRADB	0.044444	0.014493	0.028116	0.040816	0.060606	0.028056	0	0.083333
TSC1	0.244444	0.043478	0.04686	0.15102	0.030303	0.0501	0.068966	0.197917
TSC2	0.388889	0.014493	0.30553	0.122449	0.060606	0.08016	0.068966	0.020833
YWHAB	0.444444	0.258454	0.204311	0.22449	0.090909	0.659319	0	0.239583

GBM	HNSC	KICH	KIRC	KIRP	LGG	LIHC	LUAD	LUSC
0.030255	0.146552	0.454545	0.013582	0.009868	0.009381	0.015831	0.079151	0.104962
0.173567	0.043103	0.318182	0.027165	0.013158	0.026266	0.068602	0.023166	0.074427
0.203822	0.051724	0.30303	0.028862	0.013158	0.035647	0.073879	0.094595	0.187023
0.012739	0.008621	0.015152	0.035654	0.046053	0.005629	0.026385	0.032819	0.009542
0.001592	0.025862	0.045455	0.013582	0.065789	0.005629	0	0.021236	0.007634
0.009554	0.043103	0.333333	0.059423	0.411184	0.005629	0.039578	0.069498	0.022901
0.046178	0.008621	0.469697	0.04584	0.282895	0.005629	0.026385	0.090734	0.049618
0.017516	0	0.424242	0.001698	0.013158	0.005629	0.013193	0.003861	0.01145
0.022293	0.189655	0.227273	0.011885	0.0625	0.033771	0.063325	0.102317	0.244275
0.084395	0.663793	0.333333	0.054329	0.220395	0.026266	0.073879	0.119691	0.765267
0.012739	0.137931	0.424242	0.008489	0.029605	0.046904	0.029024	0.094595	0.093511
0.08758	0.12069	0	0.056027	0.036184	0.045028	0.60686	0.413127	0.230916
0.019108	0	0.439394	0.001698	0.016447	0.005629	0.007916	0.003861	0.015267
0.699045	0.215517	0.333333	0.149406	0.480263	0.236398	0.171504	0.144788	0.187023
0.050955	0	0	0.003396	0.006579	0.015009	0.050132	0.019305	0.01145
0.020701	0.025862	0.454545	0.057725	0.384868	0.013133	0.036939	0.07722	0.020992
0.049363	0.025862	0	0.001698	0.003289	0.013133	0.029024	0.03861	0.017176
0.006369	0.077586	0.424242	0.006791	0	0.005629	0.015831	0.071429	0.053435
0.036624	0.206897	0.257576	0.140917	0.069079	0.015009	0.174142	0.372587	0.486641
0.05414	0.025862	0	0.003396	0.006579	0.016886	0.050132	0.03668	0.028626
0.035032	0.025862	0.318182	0.04584	0.292763	0.020638	0.044855	0.046332	0.028626
0.070064	0.094828	0.015152	0.050934	0.019737	0.0394	0.448549	0.351351	0.208015
0.046178	0	0.454545	0.04584	0.282895	0.005629	0.034301	0.083012	0.03626
0.716561	0.094828	0.484848	0.16129	0.542763	0.260788	0.187335	0.158301	0.114504
0.015924	0	0.015152	0.035654	0.042763	0.007505	0.029024	0.019305	0.01145
0.711783	0.094828	0.515152	0.157895	0.542763	0.260788	0.17942	0.15251	0.116412
0.012739	0.094828	0.242424	0.013582	0.019737	0.013133	0.023747	0.028958	0.040076
0.035032	0.051724	0	0.027165	0.526316	0.045028	0.21372	0.167954	0.135496
0.042994	0.068966	0.030303	0.027165	0.526316	0.041276	0.240106	0.175676	0.158397
0.014331	0.086207	0.227273	0.01528	0.013158	0.013133	0.029024	0.025097	0.040076
0.014331	0.051724	0	0.011885	0.013158	0.050657	0.013193	0.034749	0.032443
0.052548	0.034483	0	0.005093	0.006579	0.015009	0.029024	0.052124	0.030534
0.011146	0.025862	0.030303	0.001698	0.009868	0	0.036939	0.005792	0.055344
0.022293	0	0.363636	0.123939	0.065789	0.009381	0.113456	0.100386	0.017176
0.283439	0.008621	0.318182	0.011885	0.009868	0.061914	0.023747	0.015444	0.026718
0.031847	0.068966	0	0.028862	0.532895	0.045028	0.200528	0.146718	0.152672
0.012739	0.051724	0.030303	0.047538	0.049342	0.005629	0.042216	0.034749	0.053435
0.065287	0.12069	0.257576	0.013582	0.003289	0.033771	0.031662	0.013514	0.053435
0.020701	0.025862	0.454545	0.057725	0.388158	0.013133	0.044855	0.07722	0.026718
0.292994	0.051724	0.424242	0.056027	0.276316	0.045028	0.137203	0.111969	0.160305

MESO	OV	PAAD	PCPG	PRAD	READ	SARC	SKCM	STAD
0.011628	0.17806	0.020134	0.006667	0.016227	0.012048	0.2	0.033898	0.011312
0.011628	0.058824	0.040268	0.033333	0.006085	0.096386	0.133333	0.040254	0.074661
0.046512	0.222576	0.127517	0.033333	0.006085	0.120482	0.291667	0.040254	0.063348
0.011628	0.144674	0.040268	0.006667	0.006085	0.012048	0.025	0.012712	0.013575
0	0.057234	0.04698	0.04	0	0.650602	0	0.091102	0.128959
0.069767	0.065183	0	0.013333	0.018256	0.108434	0.091667	0.038136	0.040724
0.023256	0.192369	0	0.04	0.010142	0.072289	0.025	0.014831	0.040724
0	0.015898	0	0.02	0.004057	0.012048	0.125	0.029661	0.006787
0.093023	0.147854	0.033557	0.033333	0.073022	0.108434	0.208333	0.108051	0.128959
0.046512	0.6407	0.026846	0	0.032454	0.096386	0.116667	0.048729	0.156109
0.05814	0.259141	0.033557	0.02	0.026369	0.036145	0.125	0.076271	0.090498
0.139535	0.486486	0.187919	0.133333	0.024341	0.204819	0.308333	0.326271	0.135747
0	0.017488	0	0.02	0.004057	0.012048	0.116667	0.033898	0.004525
0.116279	0.306836	0.067114	0.046667	0.068966	0.337349	0.166667	0.307203	0.167421
0.023256	0.162162	0	0	0.004057	0	0.091667	0.084746	0.013575
0.081395	0.082671	0.006711	0.013333	0.032454	0.108434	0.1	0.052966	0.031674
0.011628	0.138315	0.020134	0	0.002028	0	0.225	0.059322	0.056561
0.011628	0.049285	0.020134	0.02	0.006085	0.012048	0.175	0.033898	0.022624
0.197674	0.27345	0.026846	0.06	0.026369	0.168675	0.333333	0.122881	0.178733
0.023256	0.230525	0	0	0.002028	0.024096	0.266667	0.067797	0.00905
0.034884	0.152623	0	0.046667	0.018256	0.060241	0.091667	0.012712	0.022624
0.139535	0.437202	0.167785	0.146667	0.032454	0.204819	0.3	0.286017	0.115385
0.023256	0.158983	0	0.02	0.002028	0.072289	0.05	0.038136	0.027149
0.081395	0.424483	0.020134	0.053333	0.056795	0.337349	0.125	0.389831	0.079186
0.011628	0.158983	0.033557	0.013333	0.006085	0.048193	0.016667	0.021186	0.027149
0.069767	0.424483	0.020134	0.053333	0.054767	0.337349	0.125	0.389831	0.076923
0	0.162162	0.006711	0.006667	0.01217	0.108434	0.141667	0.016949	0.040724
0.139535	0.139905	0.026846	0.04	0.008114	0.120482	0.108333	0.135593	0.067873
0.104651	0.230525	0.020134	0.04	0.01217	0.072289	0.125	0.146186	0.065611
0	0.158983	0.006711	0.006667	0.010142	0.108434	0.125	0.010593	0.033937
0	0.138315	0	0.013333	0	0.036145	0.308333	0.025424	0.020362
0.034884	0.329094	0.006711	0.013333	0	0	0.191667	0.074153	0.033937
0	0.130366	0	0.013333	0	0.072289	0.05	0.023305	0.022624
0.069767	0.046105	0	0.026667	0.008114	0.048193	0.141667	0.048729	0.015837
0.069767	0.011129	0	0.073333	0.004057	0.048193	0.133333	0.03178	0.011312
0.139535	0.139905	0.04698	0.04	0.014199	0.120482	0.133333	0.148305	0.076923
0.011628	0.201908	0.040268	0.006667	0.004057	0.072289	0.05	0.025424	0.038462
0.011628	0.054054	0	0	0.046653	0.036145	0.05	0.019068	0.065611
0.081395	0.081081	0.013423	0.013333	0.032454	0.13253	0.1	0.050847	0.029412
0.034884	0.45787	0.053691	0.02	0.020284	0.879518	0.325	0.258475	0.375566

TGCT	THCA	THYM	UCEC	UCS	UVM
0.089744	0.005871	0	0.065574	0.089286	0.0375
0	0.003914	0	0.047359	0.142857	0
0	0.003914	0	0.087432	0.357143	0
0.012821	0.001957	0	0.052823	0.160714	0.025
0.00641	0	0	0.023679	0.089286	0.0375
0.019231	0.009785	0	0.029144	0.071429	0.0125
0.160256	0.009785	0	0.07286	0.196429	0
0	0.001957	0	0.010929	0	0.05
0.314103	0	0	0.153005	0.357143	0.2
0.121795	0.001957	0	0.220401	0.464286	0
0	0	0	0.038251	0.107143	0.0875
0.147436	0.054795	0.066667	0.415301	0.571429	0.0875
0	0.001957	0	0.01275	0	0.05
0.307692	0.035225	0.033333	0.116576	0.267857	0.075
0.089744	0	0	0.023679	0.107143	0
0.00641	0.013699	0	0.040073	0.125	0.0125
0.064103	0	0	0.056466	0.071429	0
0.096154	0.001957	0	0.029144	0.035714	0.0625
0	0.027397	0.033333	0.092896	0.321429	0
0.076923	0	0	0.029144	0.160714	0
0.083333	0.009785	0	0.034608	0.107143	0.025
0.121795	0.052838	0.066667	0.313297	0.446429	0.0625
0.153846	0.009785	0	0.03643	0.107143	0
0.24359	0.035225	0.033333	0.091075	0.196429	0.075
0.044872	0.001957	0	0.061931	0.214286	0.05
0.24359	0.035225	0.033333	0.096539	0.214286	0.075
0.019231	0	0	0.052823	0.017857	0.0625
0.051282	0.007828	0.066667	0.071038	0.357143	0.1875
0.057692	0.007828	0.033333	0.08561	0.410714	0.1625
0.019231	0	0	0.051002	0.035714	0.0625
0.019231	0	0	0.038251	0.321429	0.025
0.044872	0	0	0.051002	0.196429	0
0.019231	0	0	0.038251	0.214286	0.05
0	0.013699	0	0.014572	0.035714	0
0.00641	0	0	0.01275	0	0
0.051282	0.007828	0.066667	0.063752	0.321429	0.1875
0.038462	0.001957	0	0.051002	0.160714	0.05
0.012821	0.003914	0	0.010929	0.017857	0.05
0.00641	0.013699	0	0.038251	0.089286	0.0125
0.025641	0.005871	0.033333	0.136612	0.5	0.0875

Table S3: SNV frequency of mTOR pathway genes in 33 cancer types

	ACC	BLCA	BRCA	CESC	CHOL	COAD	DLBC	ESCA
AKT1	0	0.01942	0.0284	0.02768	0	0.03509	0	0.00543
AKT1S1	0	0.00728	0.00101	0.01038	0	0.01253	0.02703	0.0163
AKT2	0	0.00971	0.00609	0.02422	0	0.02256	0.02703	0.0163
CAB39	0	0.00728	0.00101	0.00692	0	0.00501	0	0.01087
CAB39L	0	0.00971	0.00304	0.01038	0	0.03759	0	0.00543
EEF2K	0	0.02184	0.0071	0.02076	0.03922	0.03759	0.02703	0.00543
EIF4B	0	0.01456	0.00304	0.02076	0	0.03008	0	0.0163
EIF4E	0.01087	0.00485	0.00203	0.0173	0	0.01754	0	0.01087
EIF4EBP1	0	0.00243	0	0	0	0	0	0
EIF4G1	0.02174	0.04126	0.00913	0.0346	0.01961	0.06266	0	0.01087
LAMTOR1	0	0	0	0.00346	0	0.01253	0.02703	0.00543
LAMTOR2	0	0.00485	0.00101	0.00346	0	0.00752	0	0
LAMTOR3	0	0.00243	0	0.00346	0	0.01003	0	0
LAMTOR4	0	0	0	0.01384	0	0.00251	0	0
LAMTOR5	0	0.01942	0	0	0	0.00251	0	0.00543
MLST8	0	0.01699	0	0.01038	0	0.01504	0	0.00543
MTOR	0	0.04369	0.02231	0.05536	0	0.10276	0	0.02717
PPM1A	0	0.01456	0.00203	0.01038	0.01961	0.02506	0	0.00543
PRKAA1	0	0.01456	0.01014	0.01038	0	0.02757	0	0.0163
PRKAA2	0	0.01699	0.00304	0.01038	0.01961	0.03509	0	0.00543
PRKAB1	0	0.01214	0.00304	0.00346	0	0.02005	0.02703	0.00543
PRKAB2	0	0	0.00304	0.00692	0.01961	0.01253	0	0.00543
PRKAG1	0.02174	0.01214	0.00304	0.0173	0.01961	0.03008	0	0.00543
PRKAG2	0.03261	0.00485	0.0071	0.01038	0	0.03008	0	0.01087
PRKAG3	0.01087	0.00485	0.00406	0.00692	0.01961	0.02256	0	0.00543
RHEB	0	0.00243	0.00101	0	0	0.00752	0	0
RPS6	0	0.03641	0.00913	0.01384	0	0.00501	0	0
RPS6KB1	0	0.00485	0.0071	0.01384	0	0.02256	0	0.0163
RPTOR	0.01087	0.0267	0.0142	0.04152	0	0.05764	0	0.0163
RRAGA	0	0.01214	0	0	0	0.01003	0	0
RRAGB	0.01087	0.00971	0.00101	0.0173	0	0.01754	0	0.0163
RRAGC	0	0	0.00101	0.00692	0	0.01504	0	0
RRAGD	0	0.00243	0.00507	0	0	0.01253	0	0.01087
SLC38A9	0.01087	0.01456	0.00913	0.01038	0	0.03759	0	0.01087
STK11	0.02174	0.00243	0.00203	0.04152	0.01961	0.02506	0.02703	0.0163
STRADA	0.02174	0.01942	0.00609	0.02768	0	0.02256	0	0.01087
STRADB	0	0.00728	0.00203	0.00692	0	0.00501	0	0
TSC1	0.01087	0.08981	0.0071	0.03806	0.01961	0.05263	0	0.02174
TSC2	0	0.04369	0.00913	0.06574	0	0.05263	0	0.04891
YWHAB	0	0.00485	0.00304	0.01038	0	0.01754	0	0.00543

GBM	HNSC	KICH	KIRC	KIRP	LAML	LGG	LIHC	LUAD
0.00513	0.00791	0	0	0	0	0.00198	0	0.00357
0	0.00198	0	0.00298	0.00356	0	0	0.00275	0.00535
0	0.01976	0	0.00298	0.01068	0	0.00198	0.00549	0.01783
0.00256	0	0	0.00298	0.00356	0	0.00395	0.00549	0.00357
0.00256	0.00593	0	0	0	0	0	0	0.00713
0.00513	0.00791	0	0	0.01068	0.00746	0	0.00275	0.01248
0.00769	0.01383	0	0	0.01068	0	0.00395	0	0.01426
0.00513	0.00198	0	0.00298	0.00356	0	0.00198	0.00549	0.00357
0	0.00395	0	0	0	0	0	0	0
0.01026	0.0415	0.01515	0.00595	0.01779	0.00746	0.00988	0.01648	0.0303
0.00256	0	0	0.00298	0.00356	0	0	0	0.00178
0	0.00395	0	0.00298	0.00356	0	0	0.00275	0.00178
0	0.00198	0	0	0	0	0.00198	0.00275	0
0.00256	0.00198	0	0	0.00712	0	0	0	0.00357
0.00256	0.00198	0	0.00298	0.00356	0	0.00198	0.00275	0.00357
0.01026	0.00395	0	0	0.00712	0	0.00198	0.01374	0.00535
0.01026	0.01779	0.0303	0.07143	0.02135	0.01493	0.01186	0.01648	0.04635
0	0.00198	0	0	0.00356	0	0.00198	0.00275	0.0107
0.00256	0.01186	0	0	0.00356	0	0.00198	0.00824	0.01426
0.00513	0.01186	0	0	0	0	0	0.00549	0.01783
0.00256	0.00593	0	0.00298	0.00356	0.00746	0.00198	0	0.00178
0	0.00198	0	0	0	0	0	0.00824	0.00535
0.00769	0.00593	0.01515	0.01488	0.00712	0	0.00395	0.00549	0.0107
0.00513	0.00791	0	0.00595	0.02135	0	0	0.01099	0.0107
0.01026	0.00395	0	0	0	0	0.00198	0.00549	0.01783
0	0.00198	0	0.00893	0.00356	0	0	0.00275	0.00713
0	0	0	0.00298	0.00356	0	0	0	0.00178
0.00513	0.01186	0	0.00893	0	0	0.00395	0.00549	0.00713
0.01026	0.01581	0.01515	0.00893	0.00356	0	0.00198	0.01923	0.03209
0.00769	0	0	0	0.00712	0	0.00395	0.00275	0.00178
0.00256	0.00198	0	0	0.00356	0	0.00395	0	0.01248
0	0.01186	0	0.00298	0	0	0.00198	0	0.01604
0.00513	0.00395	0	0	0	0	0.00198	0	0.01248
0.00256	0.00988	0	0.00298	0.00356	0	0.00198	0.00824	0.00535
0	0.00593	0	0.00298	0.00712	0	0	0.00549	0.12656
0.00256	0.01186	0	0.00298	0	0	0	0.01099	0.00357
0.00513	0.01779	0	0	0	0	0.00198	0.00275	0.00357
0.01538	0.00593	0.0303	0.01488	0.01779	0	0.00395	0.01648	0.01426
0.00513	0.00988	0.0303	0.01488	0.02847	0	0.00593	0.03297	0.02317
0.00256	0.00395	0	0.00298	0	0	0.00593	0	0.00357

LUSC	MESO	OV	PAAD	PCPG	PRAD	READ	SARC	SKCM
0.01222	0	0.00229	0	0	0.00413	0	0	0.0257
0.00815	0	0	0	0	0.00207	0	0.00422	0.00857
0.01222	0	0.00229	0	0	0.00207	0.00735	0.00844	0.03212
0.00815	0	0.00229	0	0	0.00207	0.01471	0.00844	0.00642
0.00815	0	0.00459	0	0	0.00207	0.02206	0.00422	0.01927
0.02648	0	0.00229	0	0	0.0062	0.02206	0.00422	0.04283
0.01222	0	0.00459	0.00633	0	0.00413	0.01471	0.00844	0.02784
0.00407	0	0	0.00633	0	0.00207	0.01471	0	0.01285
0.00407	0	0.00229	0	0	0	0	0	0.00428
0.02444	0	0.01376	0.00633	0	0.0062	0.05147	0.01266	0.0621
0	0	0	0	0	0	0	0.00422	0.00214
0.00204	0	0	0	0	0	0.00735	0	0.00214
0.00407	0	0	0.00633	0	0	0	0	0
0.00204	0	0	0	0	0	0.00735	0.00422	0.00428
0.00204	0	0.00688	0.00633	0	0.00207	0	0	0.00642
0.00611	0	0.00229	0.00633	0	0.00413	0.01471	0.00422	0.01713
0.0387	0	0.02064	0.01266	0.00562	0.0062	0.00735	0	0.07923
0.00815	0	0	0	0	0.00207	0.01471	0.00422	0.01285
0.00611	0	0	0	0	0	0.01471	0.00844	0.00642
0.01222	0	0.00688	0.00633	0	0.00413	0.02206	0.00422	0.11135
0	0	0.00229	0.00633	0	0	0.01471	0	0.01071
0.00407	0	0.00229	0.00633	0	0	0	0	0.01071
0.01426	0	0.00459	0	0.00562	0.00413	0.00735	0	0.0364
0.01018	0	0.00229	0	0	0.00207	0.03676	0	0.05567
0.02444	0	0.00229	0.00633	0	0.00207	0.02206	0	0.04497
0.00407	0	0.00229	0	0	0	0.01471	0	0.00428
0.01222	0.0125	0.00229	0.01266	0	0	0.02206	0.00422	0.00642
0.00407	0	0.00459	0	0	0	0	0.00844	0.01071
0.02648	0	0.00688	0.01266	0	0.0124	0.03676	0	0.07923
0.00407	0	0.00459	0	0	0.00207	0	0	0.00214
0.00407	0	0	0.00633	0	0.00207	0.00735	0	0.01071
0.00407	0	0.00459	0	0	0	0.00735	0	0.00428
0.01018	0	0.00459	0.01266	0	0	0.03676	0	0.00214
0.00204	0	0	0	0	0.00207	0.00735	0	0.01927
0.00815	0	0	0.01899	0	0.00207	0	0.00422	0.01499
0.00611	0	0.00229	0.00633	0	0.00207	0.00735	0	0.00642
0.00611	0	0	0.00633	0	0	0.01471	0	0.01713
0.02037	0.0125	0.01376	0.00633	0	0.00207	0.02206	0.01266	0.03426
0.02648	0	0.00917	0.01266	0	0.00826	0.02941	0.01688	0.07066
0.00204	0	0.00229	0.00633	0	0.00207	0	0.01688	0.00642

STAD	TGCT	THCA	THYM	UCEC	UCS	UVM
0.01386	0	0.01027	0.0082	0.04915	0	0
0.00693	0	0	0	0.03592	0	0
0.0254	0	0.00616	0	0.06994	0	0
0.00462	0	0	0	0.04915	0	0
0.01617	0	0	0.0082	0.06238	0	0
0.0254	0.0069	0	0	0.06805	0.01754	0
0.02079	0.0069	0	0.0082	0.05482	0.03509	0
0.00924	0.0069	0	0	0.03592	0.01754	0
0.00693	0	0	0	0.00567	0	0
0.06928	0	0	0.0082	0.10964	0	0
0.00231	0	0	0	0.03025	0	0
0.00462	0	0	0	0.01323	0	0
0.00693	0	0	0	0.01134	0	0
0	0	0	0	0.01134	0	0
0.00693	0	0.00205	0	0.0189	0.01754	0
0.03002	0	0	0	0.03403	0	0
0.07852	0	0	0.0082	0.12476	0	0.0125
0.00924	0	0	0	0.06994	0	0
0.01386	0	0	0	0.07183	0	0
0.03233	0	0	0	0.06049	0	0
0.0254	0	0	0	0.03592	0	0
0.00693	0	0	0	0.04348	0	0
0.02771	0	0	0	0.05482	0	0
0.02079	0	0	0	0.0794	0	0
0.01617	0	0	0	0.05482	0	0
0.00693	0	0	0	0.02079	0	0
0.00693	0	0	0	0.04537	0	0
0.01848	0	0	0	0.09641	0	0
0.04157	0	0.00205	0	0.09074	0	0
0.00231	0.0069	0	0	0.04537	0	0
0.00693	0	0	0	0.07183	0.01754	0
0.00462	0	0	0	0.03403	0	0
0.02079	0.0069	0.00205	0	0.05671	0	0
0.01386	0	0	0	0.05671	0.01754	0
0.01155	0.0069	0	0	0.03403	0	0
0.0254	0	0	0	0.06049	0	0
0.01848	0	0	0	0.04726	0	0
0.02771	0	0	0	0.10775	0.01754	0
0.05774	0	0	0	0.08885	0	0
0.00924	0	0	0.0082	0.07183	0	0

Table S4: LogFCs of mTOR pathway genes across cancer types

	BLCA	CHOL	COAD	ESCA	KICH	KIRC	KIRP	LIHC
AKT2	NA	0.60265	0.40257	0.34053	NA	-0.3199	NA	0.36222
LAMTOR5	0.52249	1.82587	0.41539	0.41353	-1.3091	-0.436	NA	0.93962
STK11	0.40822	1.23211	0.36706	NA	0.37868	0.25776	NA	0.83079
RRAGC	0.31475	1.84401	-0.4335	0.8118	NA	0.3416	0.5716	0.95713
AKT1S1	0.73953	1.30498	0.33678	0.36922	-0.0988	0.41751	0.26781	1.0829
EIF4B	-0.6276	1.67769	0.36631	NA	NA	0.37501	-0.2225	0.47486
EIF4G1	0.41223	0.57415	0.38086	0.67929	NA	-0.0899	NA	0.43976
PRKAG2	-1.3997	-0.4021	-0.4472	-0.4527	1.28332	-0.3213	-0.9273	-0.3104
STRADA	0.48303	2.32012	NA	NA	-1.1154	NA	0.39737	1.26302
PRKAA2	-2.3399	2.53176	-1.6804	-1.4111	-0.7666	-0.6595	-1.1204	2.54324
EEF2K	-0.6488	1.40212	-0.423	NA	-0.6597	0.81454	0.50016	0.90013
PRKAB1	NA	1.4625	NA	-0.6113	NA	-0.6159	NA	0.93717
CAB39	-0.199	1.29594	-0.7549	NA	-0.3922	-0.7835	-0.5377	0.51995
EIF4EBP1	1.68364	1.21916	1.60253	1.44906	-0.2198	2.07449	1.22888	0.86046
STRADB	0.53518	-0.3935	-0.803	NA	-1.0067	-0.3161	-0.4782	-0.0667
PRKAB2	NA	NA	-0.3366	-0.4869	NA	-0.1965	-0.4213	1.30186
RPS6	NA	1.81621	0.70181	NA	NA	0.55962	0.43486	0.96202
LAMTOR4	0.32125	1.67809	NA	0.72919	NA	0.31738	0.63362	1.32456
EIF4E	NA	1.14912	0.2817	NA	NA	-0.4899	-0.5098	0.31035
AKT1	0.24352	0.4807	NA	NA	0.65957	0.1086	NA	NA
RRAGA	-0.2074	0.6021	-0.0953	NA	-0.7614	0.21621	-0.1106	0.33246
PRKAG1	0.24953	1.60421	0.20664	0.41027	0.28526	NA	NA	0.91563
YWHAB	0.43099	2.0916	0.49207	0.62068	NA	-0.3894	-0.1857	0.81649
RPTOR	-0.3709	1.69792	0.22476	0.41568	0.29674	0.1621	0.60755	1.30199
RHEB	NA	1.05308	0.82528	0.9266	NA	-0.0836	0.39966	1.2152
SLC38A9	0.68748	0.78188	NA	0.44651	-0.5235	NA	0.33267	0.92746
PPM1A	-0.1771	NA	-0.7838	NA	0.6708	-0.5793	-0.4067	0.18741
PRKAG3	-0.2491	NA	0.12155	NA	-1.3028	2.20463	1.5876	1.14836
PRKAA1	NA	0.67095	-0.0852	NA	-0.2172	-0.1962	-0.4394	NA
LAMTOR3	NA	1.10038	-0.3793	NA	0.29758	-0.4541	-0.3002	0.42187
CAB39L	-0.5006	1.85575	NA	-1.7566	-0.8619	-1.6442	-1.1368	NA
RRAGB	-0.2258	2.29953	0.39084	NA	-0.228	-0.5115	-0.2249	1.10885
MLST8	0.51236	1.46796	0.80207	0.43776	0.41098	0.18753	0.37638	1.04551
LAMTOR1	0.49423	1.69473	NA	0.68833	0.26162	-0.0676	-0.0496	1.10142
RPS6KB1	NA	1.32357	0.36937	0.45997	-0.6503	0.34749	0.37786	0.73348
TSC2	NA	1.60288	0.22832	-0.167	NA	0.25205	0.13312	1.16734
TSC1	NA	1.93196	NA	NA	0.50946	NA	-0.1108	1.56729
MTOR	NA	0.45085	NA	NA	-0.6414	-1.1596	-0.6745	0.81927
RRAGD	-0.7138	NA	-0.0972	NA	1.00062	-0.5111	-0.3999	1.66105
LAMTOR2	1.0619	1.06243	0.29398	0.51668	-0.6035	0.32271	0.64783	1.20409

LUAD	LUSC	PCPG	PRAD	READ	SARC	STAD	THCA	THYM
0.35728	0.84693	-0.9684	0.21933	NA	NA	NA	-0.2965	-0.5235
0.5077	0.21589	NA	0.33189	NA	-1.8814	0.37025	0.11851	NA
NA	0.48986	NA	0.3372	NA	NA	NA	0.23701	NA
-0.1088	0.2361	NA	NA	NA	NA	0.23718	0.19741	NA
0.4447	0.89437	NA	0.51981	NA	NA	NA	-0.0758	NA
NA	NA	NA	0.24557	NA	NA	NA	NA	NA
0.58157	1.42416	-1.1033	0.45312	NA	NA	0.57297	NA	NA
NA	-0.2524	0.83691	NA	-0.7283	NA	-0.4131	-0.3872	NA
0.59664	0.53092	NA	NA	NA	NA	0.45996	NA	NA
1.41334	0.67992	NA	NA	-2.212	-3.6147	-1.4822	-0.6514	NA
NA	NA	NA	-0.4365	NA	1.71807	0.36265	NA	NA
NA	-0.643	-1.0802	-0.1678	NA	-2.1104	0.32873	0.2819	NA
NA	-0.2911	NA	NA	-0.617	NA	NA	NA	NA
1.73734	3.06466	1.21218	0.75982	1.66196	NA	0.80445	0.72628	NA
0.59533	NA	-1.5093	0.63304	NA	-3.2145	NA	1.01112	NA
0.5514	0.90884	NA	-0.511	-0.7474	NA	NA	-0.4492	NA
0.35513	NA	-0.4296	0.41963	NA	NA	-0.2775	0.11785	NA
NA	0.61975	NA	0.46124	NA	NA	NA	NA	NA
0.33402	0.41634	NA	NA	NA	NA	NA	-0.2573	NA
NA	NA	NA	0.19335	NA	NA	NA	0.2236	-0.4644
-0.3158	-0.2684	NA	-0.2335	NA	NA	-0.3234	-0.1118	NA
0.33013	0.60261	-0.7374	0.09119	NA	NA	0.18862	0.10413	NA
-0.0727	0.14312	NA	-0.1603	NA	NA	0.77528	0.07016	NA
0.29758	NA	NA	-0.307	NA	NA	0.32773	-0.1664	NA
0.77912	0.6431	NA	NA	NA	NA	0.29344	NA	NA
0.82377	0.26965	NA	NA	NA	NA	0.62997	-0.1661	NA
NA	NA	0.99388	NA	-0.7346	-1.05	-0.39	-0.2047	NA
-0.7974	NA	NA	-0.2546	NA	NA	NA	-0.3445	NA
0.40843	NA	NA	NA	NA	NA	0.81892	-0.3495	-0.6644
-0.0874	NA	NA	-0.1299	-0.6789	NA	NA	-0.2538	NA
-1.2606	-1.5517	-0.9035	NA	NA	NA	-1.7167	-0.9068	NA
-0.4011	NA	0.58363	-0.2638	NA	NA	NA	-0.2791	NA
0.60385	0.81518	NA	0.63824	1.07128	NA	0.25944	0.12117	NA
NA	NA	NA	0.21231	NA	NA	NA	-0.2791	0.54998
0.64736	0.53661	-0.5781	0.20908	NA	NA	0.54424	-0.2366	NA
0.45789	NA	1.19563	NA	NA	NA	NA	-0.0821	NA
NA	-0.128	NA	0.16599	NA	NA	0.5077	-0.3909	NA
0.53548	0.41521	NA	0.38315	NA	NA	0.54506	NA	NA
-0.3489	-0.2381	NA	NA	NA	NA	NA	-0.2793	NA
0.78537	0.36894	NA	0.6266	NA	NA	0.21994	0.38562	NA

UCEC
NA
0.91986
0.3881
-0.2688
0.48556
-0.9773
0.67348
NA
NA
-0.5995
-0.5617
0.70478
-0.6345
2.47919
0.425
-1.1013
NA
0.72481
NA
0.60956
-0.7331
0.2263
NA
-0.3859
NA
NA
-0.7439
0.40658
-0.7864
NA
-2.257
-1.156
0.77369
0.56143
-0.3212
0.4473
-0.4948
0.81188
NA
1.83067

Table S5: HRs of mTOR pathway genes across cancer types

	ACC	BLCA	CHOL	COAD	DLBC	ESCA	KICH	KIRC
PRKAB1	1.08941	NA	NA	NA	NA	NA	0.90666	NA
RPS6	1.00184	NA	NA	NA	NA	NA	NA	NA
TSC2	NA	NA	NA	NA	NA	NA	NA	NA
PPM1A	NA	NA	NA	NA	NA	NA	NA	0.84182
LAMTOR5	NA	NA	NA	NA	NA	NA	NA	0.98659
AKT1S1	1.04457	NA	NA	NA	NA	NA	NA	1.1049
EIF4G1	NA	1.00877	NA	NA	NA	NA	NA	NA
RRAGA	NA	NA	NA	NA	NA	NA	NA	0.98981
RRAGC	NA	NA	NA	NA	NA	NA	NA	NA
LAMTOR3	0.93078	0.94597	NA	NA	NA	NA	NA	0.84813
AKT1	NA	NA	1.22632	NA	NA	0.92279	NA	NA
LAMTOR1	NA	NA	NA	NA	NA	NA	1.04191	NA
CAB39L	0.6271	NA	NA	NA	NA	NA	NA	0.75566
YWHAB	NA	NA	NA	NA	NA	NA	NA	0.9874
STRADA	NA	NA	NA	NA	NA	0.37097	NA	1.74481
RRAGD	NA	NA	NA	NA	NA	NA	NA	0.97143
EIF4B	NA	NA	NA	NA	NA	NA	NA	0.98915
PRKAA2	NA	NA	NA	NA	NA	NA	NA	0.84781
EEF2K	NA	NA	NA	NA	NA	0.88565	1.49716	NA
PRKAB2	NA	NA	NA	NA	3.32376	NA	NA	NA
CAB39	NA	NA	NA	NA	NA	NA	NA	0.95879
TSC1	1.20399	NA	NA	NA	NA	NA	0.53111	NA
PRKAG2	NA	NA	NA	NA	NA	NA	NA	NA
PRKAG1	NA	NA	NA	NA	NA	NA	NA	0.92769
PRKAG3	NA	NA	NA	NA	NA	NA	2.80E+24	NA
STRADB	NA	NA	NA	NA	NA	NA	NA	NA
AKT2	1.06309	NA	NA	NA	NA	0.84525	NA	NA
EIF4E	NA	NA	NA	NA	NA	NA	NA	0.68556
RRAGB	NA	NA	NA	1.131	NA	NA	NA	NA
LAMTOR2	1.03409	NA	NA	NA	NA	NA	1.11738	1.01209
MTOR	1.17512	NA	NA	NA	NA	NA	NA	NA
EIF4EBP1	1.0417	NA	NA	NA	NA	NA	1.09419	1.0033
RPS6KB1	NA	NA	NA	NA	NA	NA	1.89477	NA
RHEB	1.04294	NA	1.17881	NA	NA	NA	1.12637	1.02829
LAMTOR4	NA	NA	NA	NA	NA	NA	1.0785	NA
STK11	NA	0.92985	NA	NA	NA	NA	NA	1.13278
PRKAA1	NA	0.96461	NA	NA	NA	NA	1.1152	0.937
RPTOR	1.11343	1.14264	NA	NA	NA	NA	NA	NA
MLST8	NA	NA	NA	NA	NA	NA	NA	NA
SLC38A9	NA	NA	NA	NA	NA	NA	56.4151	NA

KIRP	LAML	LIHC	LUAD	LUSC	MESO	OV	PAAD	PCPG
0.9591	NA	1.10559	NA	NA	NA	NA	NA	NA
1.00132	NA	NA	NA	NA	0.99911	NA	NA	0.96659
NA	NA	NA	NA	NA	NA	NA	NA	NA
NA	0.90496	NA	NA	NA	0.73956	NA	NA	NA
NA	NA	1.02203	NA	NA	NA	NA	NA	NA
1.09337	NA	NA	1.06481	NA	1.08234	NA	NA	0.58374
1.0351	NA	1.01788	1.00902	NA	NA	NA	1.02943	NA
NA	1.12919	NA	NA	NA	NA	NA	NA	NA
NA	NA	1.18036	NA	NA	NA	NA	NA	NA
NA	NA	1.10966	NA	NA	0.84921	NA	NA	NA
0.87276	NA	1.07513	NA	NA	NA	NA	NA	NA
NA	NA	NA	NA	NA	NA	NA	NA	NA
0.70131	NA	NA	NA	NA	NA	1.10803	NA	NA
NA	1.00566	1.02528	NA	NA	NA	NA	NA	NA
NA	NA	NA	NA	NA	NA	NA	0.55401	4.47043
NA	0.9336	1.05256	NA	NA	1.16376	NA	NA	NA
1.01601	NA	1.00988	NA	NA	NA	NA	NA	NA
NA	NA	NA	NA	NA	NA	NA	NA	NA
1.06226	NA	NA	NA	NA	NA	NA	NA	NA
1.31131	NA	NA	NA	NA	1.45205	NA	0.88535	NA
NA	NA	1.04616	NA	NA	NA	NA	NA	NA
NA	NA	1.22135	NA	NA	NA	NA	0.75906	NA
NA	NA	NA	0.88024	NA	NA	NA	NA	NA
NA	1.1988	1.14203	NA	NA	NA	NA	NA	NA
NA	NA	NA	NA	NA	NA	NA	NA	NA
1.06088	NA	NA	NA	NA	NA	NA	NA	NA
NA	NA	NA	NA	NA	NA	NA	NA	NA
NA	NA	2.08473	NA	NA	NA	NA	NA	NA
NA	NA	NA	NA	0.92748	NA	NA	NA	NA
NA	1.06069	NA	NA	NA	NA	NA	NA	NA
NA	NA	NA	NA	NA	NA	NA	NA	NA
1.01633	1.01242	1.00413	NA	NA	1.01116	NA	NA	NA
1.458	0.85153	NA	NA	NA	NA	NA	NA	NA
1.05835	NA	1.06239	NA	NA	1.03171	NA	1.06432	NA
NA	NA	NA	NA	NA	NA	NA	NA	NA
NA	NA	NA	NA	NA	NA	NA	NA	NA
NA	NA	1.05005	NA	NA	NA	NA	NA	NA
NA	0.9284	NA	NA	NA	NA	NA	NA	NA
NA	1.09593	1.04257	NA	NA	NA	NA	NA	0.44924
2.58472	NA	NA	NA	NA	NA	NA	NA	NA

PRAD	READ	SARC	SKCM	STAD	TGCT	THCA	THYM	UCEC
NA	NA	NA	NA	NA	NA	NA	NA	NA
NA	NA	NA	NA	NA	NA	NA	NA	NA
NA	NA	1.13009	NA	NA	NA	1.08186	NA	NA
NA	NA	1.38106	NA	1.13977	NA	NA	NA	NA
NA	NA	NA	NA	NA	NA	0.88861	NA	NA
1.13275	NA	NA	NA	NA	NA	NA	1.18119	NA
NA	NA	NA	1.00687	NA	NA	NA	NA	NA
NA	NA	NA	NA	NA	NA	NA	NA	NA
NA	NA	NA	NA	NA	NA	NA	NA	NA
NA	NA	NA	NA	NA	NA	NA	NA	NA
NA	NA	1.11349	1.02221	NA	NA	NA	NA	NA
NA	NA	0.95541	NA	NA	NA	NA	NA	NA
NA	1.11969	NA	NA	NA	NA	NA	1.17605	NA
NA	NA	NA	NA	NA	NA	NA	NA	NA
NA	NA	1.73506	NA	NA	NA	NA	NA	NA
NA	NA	NA	NA	NA	NA	NA	1.241	1.05691
NA	NA	1.04478	NA	NA	NA	NA	NA	NA
NA	NA	1.66287	NA	NA	NA	NA	NA	NA
NA	NA	NA	NA	NA	NA	NA	NA	NA
NA	NA	NA	NA	NA	NA	1.97598	1.28485	NA
NA	NA	NA	NA	NA	NA	NA	NA	NA
NA	NA	NA	NA	NA	NA	NA	NA	NA
NA	NA	NA	NA	NA	NA	NA	1.28795	NA
NA	NA	NA	NA	NA	NA	NA	NA	NA
NA	NA	NA	NA	7.30957	NA	NA	NA	6.69165
NA	NA	NA	NA	NA	NA	0.81127	NA	NA
NA	NA	NA	NA	NA	NA	1.03026	NA	NA
NA	NA	NA	NA	NA	NA	2.84299	NA	NA
1.22471	NA	NA	NA	NA	NA	NA	NA	NA
NA	NA	NA	1.00884	NA	NA	NA	NA	NA
NA	0.65239	1.18283	NA	NA	NA	NA	NA	NA
NA	NA	NA	NA	NA	NA	NA	NA	NA
NA	NA	1.18737	NA	NA	NA	NA	NA	NA
1.11642	NA	1.06309	NA	NA	NA	NA	NA	NA
NA	NA	NA	NA	NA	NA	NA	NA	NA
NA	NA	NA	NA	NA	NA	NA	NA	NA
NA	NA	NA	0.95071	NA	NA	NA	NA	1.07169
NA	NA	1.47525	1.04841	NA	NA	1.82046	0.73156	NA
NA	NA	NA	1.02544	NA	NA	NA	NA	NA
NA	NA	NA	NA	NA	NA	NA	NA	NA

UVM
NA
NA
0.81134
NA
NA
NA
0.94386
NA
NA
NA
NA
NA
NA
1.02817
NA
NA
NA
NA
NA
NA
NA
NA
NA
NA
NA
1.05324
0.7005
NA
NA
1.07563
NA
NA
NA
1.31403
1.05734
NA
NA
NA
NA
3.429

Table S6: Cluster and mTOR_score informations

ID	Cluster	mTOR_score P = 1.96e-14
TCGA-A3-3306-01A-01R-0864-07	C1	0.290522107
TCGA-A3-3329-01A-01R-0864-07	C2	0.390999944
TCGA-B8-5550-01A-01R-1541-07	C2	0.260471989
TCGA-A3-3378-01A-02R-1325-07	C3	-0.085558413
TCGA-A3-3343-01A-01R-0864-07	C2	0.366230617
TCGA-B0-5100-01A-01R-1420-07	C3	-0.228030548
TCGA-CJ-4869-01A-02R-1426-07	C3	-0.250182524
TCGA-CJ-4895-01A-01R-1305-07	C3	0.049423812
TCGA-B0-5691-01A-11R-1541-07	C2	-0.168239657
TCGA-B0-5121-01A-02R-1420-07	C3	-0.470214129
TCGA-B0-5098-01A-01R-1420-07	C2	-0.166531385
TCGA-A3-3308-01A-02R-1325-07	C2	0.010885035
TCGA-BP-4807-01A-01R-1305-07	C2	0.334925575
TCGA-B0-5707-01A-11R-1541-07	C3	0.05657192
TCGA-BP-4971-01A-01R-1334-07	C3	-0.411548837
TCGA-B8-4620-01A-02R-1325-07	C2	-0.414702796
TCGA-BP-4967-01A-01R-1334-07	C2	0.053395026
TCGA-BP-5198-01A-01R-1426-07	C2	-0.028763917
TCGA-BP-4326-01A-01R-1289-07	C2	-0.082618532
TCGA-CJ-4904-01A-02R-1426-07	C2	-0.064384449
TCGA-CZ-4853-01A-01R-1426-07	C2	0.1733768
TCGA-A3-3387-01A-01R-1541-07	C2	0.077056573
TCGA-A3-3326-01A-01R-0864-07	C2	0.010492811
TCGA-BP-4784-01A-01R-1305-07	C2	-0.083897962
TCGA-BP-4766-01A-01R-1289-07	C2	0.332722955
TCGA-CZ-5456-01A-01R-1503-07	C2	-0.048614572
TCGA-CZ-4857-01A-01R-1305-07	C2	-0.208361401
TCGA-B0-5400-01A-01R-1503-07	C3	-0.090643813
TCGA-BP-5010-01A-02R-1420-07	C3	-0.456320442
TCGA-CJ-4644-01A-02R-1325-07	C2	0.265016566
TCGA-CJ-6033-01A-11R-1672-07	C3	-0.0516457
TCGA-EU-5907-01A-11R-1672-07	C2	0.336616001
TCGA-B4-5836-01A-11R-1672-07	C2	0.175222403
TCGA-A3-3323-01A-02R-1325-07	C2	-0.291045352
TCGA-BP-4353-01A-02R-1289-07	C2	-0.187780567
TCGA-B0-5077-01A-01R-1334-07	C2	-0.059030968
TCGA-CZ-5984-01A-11R-1672-07	C2	-0.248696812
TCGA-B8-A54G-01A-11R-A266-07	C3	-0.186816827
TCGA-B0-4713-01A-01R-1277-07	C3	-0.182428501
TCGA-CZ-5463-01A-01R-1503-07	C2	0.089740389
TCGA-6D-AA2E-01A-11R-A37O-07	C3	0.272211519
TCGA-CJ-6032-01A-11R-1672-07	C2	0.274468127
TCGA-BP-4801-01A-02R-1420-07	C2	0.210537981
TCGA-EU-5904-01A-11R-1672-07	C2	-0.28225821
TCGA-B8-5158-01A-01R-1420-07	C2	-0.229092199
TCGA-BP-4969-01A-01R-1334-07	C3	0.10091351
TCGA-B8-5546-01A-01R-1541-07	C2	0.484032894

TCGA-B4-5844-01A-11R-1672-07	C2	0.156597401
TCGA-CZ-5452-01A-01R-1503-07	C3	0.029176034
TCGA-BP-4991-01A-01R-1334-07	C2	-0.16392819
TCGA-A3-3316-01A-01R-0864-07	C2	-0.219526072
TCGA-B8-A54E-01A-11R-A266-07	C3	0.2285716
TCGA-BP-5170-01A-01R-1426-07	C3	-0.271333753
TCGA-A3-A6NI-01A-11R-A33J-07	C2	0.052754109
TCGA-B0-5711-01A-11R-1672-07	C2	-0.177939887
TCGA-CZ-5451-01A-01R-1503-07	C2	0.159250436
TCGA-BP-4340-01A-01R-1289-07	C2	0.251316661
TCGA-BP-5180-01A-01R-1426-07	C3	-0.235190595
TCGA-CJ-4639-01A-02R-1325-07	C2	0.001023488
TCGA-B0-4821-01A-01R-1503-07	C3	-0.210514745
TCGA-CZ-4860-01A-01R-1305-07	C2	0.082706485
TCGA-B0-4813-01A-01R-1277-07	C3	-0.207418841
TCGA-B8-4619-01A-02R-1325-07	C1	0.216824322
TCGA-CJ-4892-01A-01R-1305-07	C2	0.221796979
TCGA-CJ-4916-01A-01R-1426-07	C3	-0.315594713
TCGA-BP-4964-01A-01R-1334-07	C2	-0.10136419
TCGA-B0-5712-01A-11R-1672-07	C2	0.106000832
TCGA-BP-4965-01A-01R-1334-07	C2	-0.149368646
TCGA-BP-5199-01A-01R-1426-07	C2	-0.321214838
TCGA-BP-4973-01A-01R-1334-07	C2	-0.051136597
TCGA-B2-5635-01A-01R-A277-07	C2	-0.1464586
TCGA-B0-4844-01A-01R-1277-07	C3	-0.068315865
TCGA-G6-A8L7-01A-11R-A37O-07	C3	-0.199107568
TCGA-B0-5088-01A-01R-1334-07	C3	-0.183641591
TCGA-A3-3324-01A-02R-1325-07	C2	0.084624852
TCGA-BP-4968-01A-01R-1334-07	C2	-0.022899074
TCGA-BP-4352-01A-01R-1289-07	C2	-0.127138206
TCGA-CJ-4872-01A-01R-1305-07	C2	-0.27865187
TCGA-DV-A4VZ-01A-11R-A266-07	C2	0.024151103
TCGA-BP-5006-01A-01R-1334-07	C2	0.166532787
TCGA-BP-5009-01A-01R-1334-07	C2	-0.485822873
TCGA-B0-4699-01A-01R-1277-07	C1	0.22466137
TCGA-B0-4834-01A-01R-1305-07	C1	0.280980291
TCGA-BP-4998-01A-01R-1334-07	C2	-0.275752287
TCGA-B0-5104-01A-01R-1420-07	C2	0.283580402
TCGA-B0-4945-01A-01R-1420-07	C2	-0.003442069
TCGA-B0-4842-01A-02R-1420-07	C3	-0.201117691
TCGA-AK-3447-01A-01R-1766-07	C1	0.2755227
TCGA-B8-5551-01A-01R-1541-07	C3	-0.060684842
TCGA-A3-3349-01A-01R-1188-07	C2	-0.21045454
TCGA-B0-4693-01A-01R-1277-07	C2	0.101239857
TCGA-A3-3346-01A-01R-1766-07	C3	0.063716312
TCGA-B0-5120-01A-01R-1420-07	C2	-0.24322083
TCGA-BP-5177-01A-01R-1426-07	C2	-0.330659841
TCGA-B8-4154-01A-01R-1188-07	C1	0.127730047
TCGA-B8-4153-01B-11R-1672-07	C3	-0.129901445

TCGA-CJ-6030-01A-11R-1672-07	C2	-0.181466936
TCGA-CW-5584-01A-01R-1541-07	C2	0.266607674
TCGA-B0-5696-01A-11R-1541-07	C3	0.298370866
TCGA-BP-4765-01A-01R-1289-07	C2	0.273158257
TCGA-BP-4782-01A-02R-1420-07	C2	0.087345762
TCGA-B8-5165-01A-01R-1420-07	C2	-0.124726791
TCGA-B2-3923-01A-02R-A277-07	C1	0.268914217
TCGA-AK-3425-01A-02R-1277-07	C2	-0.007357317
TCGA-AS-3778-01A-01R-A32Z-07	C2	0.054969625
TCGA-CW-6087-01A-11R-1672-07	C2	-0.059575116
TCGA-B0-5108-01A-01R-1420-07	C2	-0.379634239
TCGA-B0-5699-01A-11R-1541-07	C2	0.264695465
TCGA-B0-4814-01A-01R-1277-07	C2	0.179102096
TCGA-AK-3465-01A-02R-1325-07	C1	0.35940383
TCGA-CJ-4918-01A-01R-1426-07	C2	-0.319897853
TCGA-BP-4344-01A-01R-1289-07	C2	-0.172886909
TCGA-B4-5835-01A-11R-1672-07	C3	0.350976934
TCGA-A3-3372-01A-02R-1325-07	C2	-0.13190373
TCGA-BP-4981-01A-01R-1334-07	C3	-0.258166157
TCGA-BP-4170-01A-02R-1289-07	C2	0.236316857
TCGA-CJ-4643-01A-02R-1325-07	C2	0.011597403
TCGA-BP-4977-01A-01R-1334-07	C2	-0.109333763
TCGA-B2-5633-01A-01R-1541-07	C2	-0.415851317
TCGA-A3-3311-01A-02R-1325-07	C2	-0.000975274
TCGA-BP-5173-01A-01R-1426-07	C2	0.085497145
TCGA-BP-5007-01A-01R-1334-07	C2	-0.144924838
TCGA-A3-3362-01A-02R-1325-07	C2	0.290103751
TCGA-BP-4781-01A-01R-1305-07	C2	0.005887925
TCGA-A3-3365-01A-01R-0864-07	C2	0.049815524
TCGA-DV-A4W0-01A-11R-A266-07	C2	-0.230899434
TCGA-B0-5092-01A-01R-1420-07	C3	-0.361146742
TCGA-CJ-5683-01A-11R-1541-07	C2	0.184787897
TCGA-BP-4331-01A-01R-1289-07	C2	0.306736831
TCGA-B2-A4SR-01A-11R-A266-07	C2	-0.118546599
TCGA-BP-4976-01A-01R-1334-07	C2	0.157100167
TCGA-B2-4099-01A-02R-1188-07	C1	0.127861481
TCGA-B2-3923-01B-10R-A277-07	C1	0.054690821
TCGA-G6-A5PC-01A-11R-A33J-07	C3	-0.189974784
TCGA-B0-5812-01A-11R-1672-07	C2	-0.031294114
TCGA-BP-4341-01A-01R-1289-07	C3	0.017167103
TCGA-BP-4795-01A-02R-1420-07	C2	-0.214768874
TCGA-AK-3451-01A-02R-1188-07	C3	0.007669214
TCGA-CZ-5987-01A-11R-1672-07	C3	-0.211096063
TCGA-MM-A564-01A-11R-A266-07	C3	-0.135709675
TCGA-BP-4787-01A-01R-1305-07	C2	-0.315691949
TCGA-DV-5566-01A-01R-1541-07	C3	0.062396874
TCGA-BP-5190-01A-01R-1426-07	C3	-0.166384611
TCGA-BP-4162-01A-02R-1325-07	C2	-0.004257146
TCGA-BP-4343-01A-02R-1289-07	C2	0.050894081

TCGA-AK-3458-01A-01R-1503-07	C1	0.031093135
TCGA-CZ-5457-01A-01R-1503-07	C2	0.30746882
TCGA-A3-A8OX-01A-11R-A37O-07	C2	-0.085289827
TCGA-B0-4817-01A-01R-1277-07	C3	-0.277666012
TCGA-CJ-4888-01A-01R-1305-07	C2	-0.271267862
TCGA-BP-5191-01A-01R-1426-07	C3	-0.410934955
TCGA-B2-5635-01A-01R-1541-07	C2	-0.341730717
TCGA-T7-A92I-01A-11R-A37O-07	C2	0.294713323
TCGA-CJ-4901-01A-01R-1426-07	C2	-0.446359311
TCGA-G6-A8L8-01A-21R-A37O-07	C2	0.11300279
TCGA-BP-4173-01A-02R-1289-07	C2	-0.367753722
TCGA-AK-3434-01A-02R-1277-07	C2	-0.150614899
TCGA-BP-4329-01A-02R-1289-07	C2	0.162982384
TCGA-CZ-5462-01A-01R-1503-07	C2	0.048655964
TCGA-B0-4706-01A-01R-1503-07	C3	-0.125632916
TCGA-CJ-4920-01A-01R-1426-07	C2	-0.16926446
TCGA-B8-A54I-01A-21R-A33J-07	C3	-0.187245392
TCGA-CJ-4893-01A-01R-1305-07	C2	0.166051526
TCGA-BP-4960-01A-01R-1334-07	C3	-0.409845785
TCGA-B2-3924-01A-02R-1325-07	C2	0.0337603
TCGA-B2-3923-01A-02R-1325-07	C1	0.275396477
TCGA-BP-5185-01A-01R-1426-07	C3	-0.241424046
TCGA-A3-3357-01A-02R-1420-07	C2	0.163217234
TCGA-A3-A8CQ-01A-11R-A37O-07	C2	0.206177317
TCGA-BP-5176-01A-01R-1426-07	C3	0.305546403
TCGA-BP-4160-01A-02R-1289-07	C2	0.053723868
TCGA-B2-3924-01A-02R-A277-07	C2	0.158956344
TCGA-B0-4822-01A-01R-1277-07	C3	-0.325682934
TCGA-A3-3328-01A-01R-0864-07	C1	0.381104032
TCGA-CW-5583-01A-02R-1541-07	C2	0.088062839
TCGA-B8-4148-01A-02R-1325-07	C2	-0.086658369
TCGA-BP-4959-01A-01R-1334-07	C2	0.297232108
TCGA-B0-5097-01A-01R-1420-07	C2	-0.207847293
TCGA-CW-6090-01A-11R-1672-07	C2	-0.049901284
TCGA-B2-4102-01A-02R-1325-07	C2	0.075776155
TCGA-BP-4799-01A-01R-1305-07	C2	-0.377324986
TCGA-BP-4177-01A-02R-1420-07	C2	-0.16332681
TCGA-CZ-4858-01A-01R-1305-07	C2	-0.193905557
TCGA-AK-3450-01A-02R-1277-07	C2	0.126470603
TCGA-B0-5102-01A-01R-1420-07	C3	0.218282668
TCGA-BP-4167-01A-02R-1325-07	C3	-0.107375186
TCGA-BP-4989-01A-01R-1334-07	C3	-0.240984849
TCGA-BP-5200-01A-01R-1426-07	C2	-0.168305454
TCGA-B0-4818-01A-01R-1503-07	C2	0.205990933
TCGA-B0-4714-01A-01R-1277-07	C3	-0.149722805
TCGA-3Z-A93Z-01A-11R-A37O-07	C2	0.227255044
TCGA-DV-5574-01A-01R-1541-07	C2	-0.292843182
TCGA-BP-5194-01A-02R-1426-07	C2	-0.04330282
TCGA-BP-4961-01A-01R-1334-07	C2	-0.125007246

TCGA-BP-4963-01A-01R-1334-07	C2	-0.076829546
TCGA-AK-3460-01A-02R-1277-07	C2	0.013988468
TCGA-B8-5545-01A-01R-1672-07	C2	-0.136914522
TCGA-BP-4165-01A-02R-1289-07	C3	-0.060846739
TCGA-DV-A4VX-01A-11R-A266-07	C3	0.086382387
TCGA-B0-4846-01A-01R-1277-07	C2	-0.00187116
TCGA-CZ-5453-01A-01R-1503-07	C2	0.142624162
TCGA-B0-5095-01A-01R-1420-07	C2	-0.353792042
TCGA-BP-4972-01A-01R-1334-07	C2	0.114528789
TCGA-B0-4712-01A-01R-1503-07	C3	0.058648048
TCGA-BP-4164-01A-02R-1325-07	C2	0.307600055
TCGA-CZ-5454-01A-01R-1503-07	C2	0.070917932
TCGA-BP-5202-01A-02R-1426-07	C2	0.203395683
TCGA-CZ-5470-01A-01R-1503-07	C3	-0.115723016
TCGA-CJ-4902-01A-01R-1426-07	C2	-0.318867373
TCGA-CJ-4905-01A-02R-1426-07	C2	0.06199709
TCGA-B0-5075-01A-01R-1334-07	C2	0.099046029
TCGA-BP-4798-01A-01R-1305-07	C2	-0.020365606
TCGA-CJ-4882-01A-02R-1426-07	C3	-0.401882027
TCGA-B0-5706-01A-11R-1541-07	C2	-0.276067596
TCGA-CJ-4891-01A-01R-1305-07	C3	-0.073017063
TCGA-CZ-5988-01A-11R-1672-07	C2	-0.186393164
TCGA-AK-3440-01A-02R-1277-07	C1	0.436771702
TCGA-AK-3453-01A-02R-1277-07	C3	-0.290938935
TCGA-B0-5697-01A-11R-1541-07	C3	-0.088074506
TCGA-B0-4841-01A-01R-1277-07	C3	-0.334831783
TCGA-CJ-5679-01A-11R-1541-07	C3	-0.083213053
TCGA-B0-4845-01A-01R-1277-07	C2	-0.280574244
TCGA-CZ-5465-01A-01R-1503-07	C2	0.317547839
TCGA-CJ-5686-01A-11R-1672-07	C2	0.016946277
TCGA-BP-4345-01A-01R-1289-07	C2	-0.263713859
TCGA-B0-5693-01A-11R-1541-07	C2	0.16665435
TCGA-B0-4838-01A-01R-1305-07	C2	0.166286144
TCGA-B8-5549-01A-01R-1541-07	C2	-0.290383871
TCGA-CJ-4876-01A-01R-1305-07	C3	-0.049324789
TCGA-BP-4337-01A-01R-1289-07	C2	-0.065127278
TCGA-BP-4985-01A-01R-1334-07	C3	-0.060704719
TCGA-A3-3374-01A-02R-1325-07	C1	0.377393279
TCGA-A3-3373-01A-02R-1420-07	C2	0.026378874
TCGA-B0-5700-01A-11R-1541-07	C2	0.09352369
TCGA-B4-5378-01A-01R-1503-07	C2	-0.192207093
TCGA-B0-5709-01A-11R-1541-07	C2	-0.155337496
TCGA-BP-4351-01A-01R-1289-07	C2	-0.27125431
TCGA-BP-4774-01A-01R-1289-07	C2	-0.140267822
TCGA-BP-4986-01A-01R-1334-07	C2	-0.224991459
TCGA-B0-5113-01A-01R-1420-07	C2	-0.186627534
TCGA-A3-A6NL-01A-11R-A33J-07	C2	-0.069827465
TCGA-CJ-5680-01A-11R-1541-07	C2	0.151660579
TCGA-B0-5402-01A-01R-1503-07	C2	-0.124692579

TCGA-CZ-4862-01A-01R-1305-07	C2	-0.202146514
TCGA-CW-6097-01A-11R-1672-07	C2	-0.177682063
TCGA-B4-5843-01A-11R-1672-07	C2	0.110381104
TCGA-A3-3307-01A-01R-0864-07	C2	0.339826466
TCGA-B0-4688-01A-01R-1277-07	C2	-0.00631199
TCGA-CJ-5689-01A-11R-1541-07	C3	-0.061716889
TCGA-BP-4335-01A-01R-1289-07	C3	-0.290608063
TCGA-CJ-4908-01A-01R-1426-07	C2	-0.255598946
TCGA-CZ-5458-01A-01R-1503-07	C2	0.145396138
TCGA-B0-4690-01A-01R-1277-07	C2	-0.29878689
TCGA-CJ-4900-01A-01R-1334-07	C3	-0.334595536
TCGA-A3-3359-01A-01R-0864-07	C2	0.107212441
TCGA-CJ-4907-01A-01R-1426-07	C2	-0.155244009
TCGA-CZ-5460-01A-01R-1503-07	C3	0.110578953
TCGA-B8-4622-01A-02R-1277-07	C2	-0.142888188
TCGA-BP-4994-01A-01R-1334-07	C1	0.213087876
TCGA-B0-4703-01A-01R-1277-07	C2	-0.395714639
TCGA-BP-4346-01A-01R-1289-07	C2	-0.045858508
TCGA-B0-4839-01A-01R-1305-07	C3	-0.151289946
TCGA-CZ-5985-01A-11R-1672-07	C2	-0.132965991
TCGA-CZ-4865-01A-02R-1503-07	C3	0.030830221
TCGA-CJ-5675-01A-11R-1541-07	C2	-0.010608826
TCGA-AK-3429-01A-02R-1325-07	C3	0.006319646
TCGA-B0-4843-01A-01R-1277-07	C3	-0.267915389
TCGA-CJ-4886-01A-01R-1305-07	C2	-0.075964654
TCGA-B0-5083-01A-02R-1420-07	C1	0.211230708
TCGA-B0-5117-01A-01R-1420-07	C1	0.240486901
TCGA-BP-4325-01A-02R-1289-07	C2	0.227195542
TCGA-CW-5580-01A-01R-1672-07	C2	0.100252489
TCGA-BP-5000-01A-01R-1334-07	C3	-0.437710448
TCGA-B0-5106-01A-01R-1420-07	C3	-0.362831447
TCGA-CJ-6031-01A-11R-1672-07	C2	-0.403815018
TCGA-B0-4852-01A-01R-1503-07	C2	-0.092677696
TCGA-B0-5690-01A-11R-1541-07	C2	-0.215086415
TCGA-B0-4697-01A-01R-1277-07	C3	-0.347008956
TCGA-CZ-4859-01A-02R-1426-07	C2	0.397510692
TCGA-BP-4789-01A-01R-1305-07	C2	0.021600049
TCGA-CZ-5989-01A-11R-1672-07	C1	-0.059030429
TCGA-A3-3347-01A-02R-1325-07	C2	-0.268276907
TCGA-B0-4827-01A-02R-1420-07	C2	0.273884865
TCGA-BP-4760-01A-02R-1420-07	C2	-0.165415453
TCGA-CW-5581-01A-02R-1541-07	C2	0.365029513
TCGA-AK-3427-01A-01R-0864-07	C1	0.167324344
TCGA-AK-3445-01A-02R-1277-07	C3	-0.266966807
TCGA-BP-4763-01A-01R-1289-07	C2	-0.065184945
TCGA-BP-4776-01A-01R-1289-07	C3	-0.173307272
TCGA-BP-5008-01A-01R-1334-07	C2	-0.226823376
TCGA-B0-5119-01A-02R-1420-07	C2	0.180896963
TCGA-CJ-5684-01A-11R-1541-07	C3	0.078660266

TCGA-BP-4338-01A-01R-1289-07	C2	0.127637398
TCGA-B0-5085-01A-01R-1334-07	C3	-0.138918366
TCGA-A3-3380-01A-01R-0864-07	C2	-0.124764376
TCGA-B0-4691-01A-01R-1277-07	C3	-0.39830132
TCGA-B0-5695-01A-11R-1541-07	C2	0.289952055
TCGA-A3-3317-01A-02R-1325-07	C2	-0.155961805
TCGA-CJ-5672-01A-11R-1541-07	C3	-0.170902197
TCGA-CJ-5677-01A-11R-1541-07	C3	0.053191545
TCGA-A3-3367-01A-02R-1420-07	C2	0.031071417
TCGA-B8-A54K-01A-11R-A33J-07	C2	-0.091315619
TCGA-BP-4992-01A-01R-1334-07	C3	-0.373104941
TCGA-B0-5705-01A-11R-1541-07	C2	0.202312911
TCGA-CW-6088-01A-11R-1672-07	C2	0.125770795
TCGA-BP-4758-01A-01R-1289-07	C3	-0.133056751
TCGA-BP-4355-01A-01R-1289-07	C3	-0.223542486
TCGA-CZ-4866-01A-01R-1503-07	C2	0.359288053
TCGA-B8-4621-01A-01R-1503-07	C3	0.214555709
TCGA-BP-4995-01A-01R-1334-07	C1	0.225070028
TCGA-BP-5004-01A-01R-1334-07	C2	-0.042858274
TCGA-B8-5162-01A-01R-1420-07	C2	-0.141848914
TCGA-B0-4847-01A-01R-1277-07	C3	-0.325081123
TCGA-BP-4769-01A-01R-1289-07	C2	0.128458406
TCGA-CZ-5982-01A-11R-1672-07	C2	0.221881419
TCGA-CJ-4870-01A-01R-1305-07	C3	0.134482183
TCGA-AK-3454-01A-02R-1277-07	C3	-0.345179254
TCGA-BP-4975-01A-01R-1334-07	C2	0.177724846
TCGA-CW-5585-01A-01R-1541-07	C2	0.281179893
TCGA-B2-4098-01A-02R-1325-07	C2	0.056848629
TCGA-CJ-5678-01A-11R-1541-07	C3	-0.066046559
TCGA-B0-5702-01A-11R-1541-07	C3	-0.111143084
TCGA-CJ-4638-01A-02R-1325-07	C3	-0.271368862
TCGA-DV-5573-01A-01R-1541-07	C2	-0.070834328
TCGA-BP-5196-01A-01R-1426-07	C3	-0.352194225
TCGA-A3-3351-01A-02R-1325-07	C2	-0.149243965
TCGA-B8-A54D-01A-21R-A266-07	C2	-0.175192486
TCGA-A3-3322-01A-02R-1325-07	C2	0.050890163
TCGA-B8-A54H-01A-11R-A33J-07	C2	0.072608598
TCGA-A3-A8OW-01A-11R-A37O-07	C2	-0.015021514
TCGA-B0-4701-01A-01R-1277-07	C2	0.119583552
TCGA-B2-4101-01A-02R-1277-07	C2	-0.179892342
TCGA-A3-3320-01A-02R-1325-07	C2	0.119390977
TCGA-BP-4999-01A-01R-1334-07	C2	-0.173409604
TCGA-BP-4161-01A-02R-1325-07	C2	0.105471186
TCGA-B8-4143-01A-01R-1188-07	C3	0.001004801
TCGA-B0-5701-01A-11R-1541-07	C2	0.043791764
TCGA-BP-4797-01A-01R-1305-07	C2	0.061980203
TCGA-DV-A4W0-05A-11R-A266-07	C2	-0.141252357
TCGA-BP-4174-01A-02R-1289-07	C2	0.354868849
TCGA-A3-3385-01A-02R-1420-07	C2	0.250342653

TCGA-CZ-5469-01A-01R-1503-07	C3	-0.164694694
TCGA-BP-4970-01A-01R-1334-07	C2	-0.212687026
TCGA-BP-4330-01A-01R-1289-07	C2	-0.061675176
TCGA-BP-4349-01A-01R-1289-07	C2	-0.114312826
TCGA-AK-3461-01A-02R-1277-07	C2	0.081828229
TCGA-A3-3383-01A-02R-1325-07	C3	0.027487957
TCGA-BP-4159-01A-02R-1289-07	C2	-0.13543075
TCGA-B2-5635-01B-04R-A277-07	C1	-0.277736784
TCGA-CJ-4889-01A-01R-1305-07	C2	-0.199977513
TCGA-BP-4962-01A-01R-1334-07	C3	0.145620943
TCGA-CJ-5682-01A-11R-1541-07	C3	0.03855186
TCGA-BP-4347-01A-01R-1289-07	C2	-0.047249263
TCGA-BP-5186-01A-01R-1426-07	C2	0.320561377
TCGA-CZ-4856-01A-02R-1426-07	C2	0.045526564
TCGA-B0-4824-01A-01R-1277-07	C2	0.100173977
TCGA-A3-3376-01A-02R-1420-07	C2	-0.149655836
TCGA-CJ-4635-01A-02R-1305-07	C2	-0.311790324
TCGA-BP-5183-01A-01R-1426-07	C2	-0.123766155
TCGA-A3-A8OV-01A-11R-A37O-07	C3	0.233915724
TCGA-B8-5159-01A-01R-1420-07	C2	0.146726971
TCGA-B8-5163-01A-01R-1420-07	C2	-0.268711211
TCGA-CZ-5461-01A-01R-1503-07	C2	0.053148317
TCGA-GK-A6C7-01A-11R-A33J-07	C2	-0.011990765
TCGA-BP-4771-01A-01R-1289-07	C3	-0.224582914
TCGA-BP-4803-01A-01R-1305-07	C2	0.017595287
TCGA-B2-5639-01A-01R-1541-07	C2	0.332906322
TCGA-CZ-5455-01A-01R-1503-07	C2	-0.094066651
TCGA-CJ-4897-01A-03R-1426-07	C2	0.263029402
TCGA-B0-5107-01A-01R-1420-07	C3	0.001475339
TCGA-BP-4759-01A-01R-1289-07	C3	-0.021891026
TCGA-B0-4836-01A-01R-1305-07	C3	-0.112772743
TCGA-B8-A7U6-01A-12R-A37O-07	C2	0.002744067
TCGA-B2-5636-01A-02R-1541-07	C2	-0.17059113
TCGA-A3-3382-01A-02R-1325-07	C2	0.049100322
TCGA-CZ-5464-01A-01R-1503-07	C2	-0.388600634
TCGA-CJ-4634-01A-02R-1325-07	C2	0.304459469
TCGA-CJ-6028-01A-11R-1672-07	C2	0.024617602
TCGA-BP-4804-01A-02R-1305-07	C3	-0.340825469
TCGA-CJ-4912-01A-01R-1426-07	C3	-0.304858147
TCGA-CJ-4873-01A-01R-1305-07	C3	-0.313539394
TCGA-CJ-4894-01A-01R-1305-07	C2	-0.202952755
TCGA-CW-5588-01A-01R-1541-07	C2	-0.097676021
TCGA-A3-3325-01A-01R-0864-07	C2	-0.02275591
TCGA-CJ-4871-01A-01R-1305-07	C2	-0.009383417
TCGA-CJ-4640-01A-02R-1325-07	C3	-0.018196467
TCGA-BP-4762-01A-02R-1289-07	C2	0.041847728
TCGA-B0-5094-01A-01R-1420-07	C2	-0.249648487
TCGA-CZ-5467-01A-01R-1503-07	C2	0.146180168
TCGA-A3-3363-01A-01R-0864-07	C1	0.342947121

TCGA-CZ-5468-01A-01R-1503-07	C3	0.010578743
TCGA-B2-3924-01B-03R-A277-07	C1	-0.21478771
TCGA-BP-5181-01A-01R-1426-07	C2	0.246132346
TCGA-B8-5553-01A-01R-1541-07	C2	0.105173093
TCGA-BP-4761-01A-01R-1289-07	C3	-0.112148035
TCGA-B2-5633-01A-01R-A277-07	C2	-0.142609125
TCGA-A3-3358-01A-01R-1541-07	C2	0.141161868
TCGA-CZ-4864-01A-01R-1503-07	C2	0.048456032
TCGA-AK-3436-01A-02R-1325-07	C3	-0.016903865
TCGA-CZ-5466-01A-01R-1503-07	C2	-0.125583371
TCGA-B0-5080-01A-01R-1503-07	C2	-0.237932648
TCGA-CZ-4861-01A-01R-1305-07	C2	-0.150873125
TCGA-CJ-5671-01A-11R-1541-07	C2	-0.294921724
TCGA-AK-3431-01A-02R-1277-07	C2	0.0999422
TCGA-B0-4811-01A-01R-1503-07	C3	-0.306636214
TCGA-A3-3313-01A-02R-1325-07	C2	0.218486292
TCGA-B8-5552-01B-11R-1672-07	C2	-0.267747828
TCGA-CJ-6027-01A-11R-1672-07	C3	-0.166836718
TCGA-BP-4756-01A-01R-1289-07	C2	0.403553622
TCGA-A3-3319-01A-02R-1325-07	C2	-0.107608995
TCGA-MW-A4EC-01A-11R-A266-07	C2	0.151669096
TCGA-B0-4816-01A-01R-1503-07	C2	-0.012102995
TCGA-CZ-4863-01A-01R-1503-07	C2	0.091566972
TCGA-B0-5110-01A-01R-1420-07	C2	0.023630422
TCGA-CZ-5986-01A-11R-1672-07	C2	-0.084771963
TCGA-B0-4828-01A-01R-1277-07	C2	0.036690317
TCGA-B8-4151-01A-01R-1188-07	C2	0.336570484
TCGA-EU-5905-01A-11R-1672-07	C2	-0.42547759
TCGA-CJ-4903-01A-01R-1426-07	C2	0.025702069
TCGA-B0-5698-01A-11R-1672-07	C2	0.032168492
TCGA-B0-4815-01A-01R-1503-07	C2	-0.27629269
TCGA-B0-5081-01A-01R-1334-07	C2	-0.431189686
TCGA-A3-3352-01A-01R-0864-07	C2	0.270800861
TCGA-A3-A6NJ-01A-12R-A33J-07	C2	0.229416015
TCGA-BP-5184-01A-01R-1426-07	C2	0.130689972
TCGA-CJ-4899-01A-01R-1334-07	C2	-0.085024567
TCGA-CJ-4881-01A-01R-1305-07	C3	-0.246126928
TCGA-CJ-4878-01A-01R-1305-07	C2	0.214419022
TCGA-B0-4819-01A-01R-1277-07	C3	-0.449528151
TCGA-B0-4710-01A-01R-1503-07	C2	0.038292134
TCGA-A3-3331-01A-02R-1325-07	C2	-0.002467913
TCGA-BP-5174-01A-01R-1426-07	C3	0.098991615
TCGA-B0-5694-01A-11R-1541-07	C2	-0.374181261
TCGA-B0-5116-01A-02R-1420-07	C2	-0.145093517
TCGA-DV-5567-01A-01R-1541-07	C2	-0.113887648
TCGA-BP-5169-01A-01R-1426-07	C3	-0.209200092
TCGA-B0-4700-01A-02R-1541-07	C3	-0.281547761
TCGA-AK-3433-01A-02R-1277-07	C1	0.410429455
TCGA-CJ-4875-01A-01R-1305-07	C3	-0.210147741

TCGA-A3-3335-01A-01R-0864-07	C3	0.01995609
TCGA-B0-5115-01A-01R-1420-07	C2	-0.075033059
TCGA-BP-5195-01A-02R-1426-07	C2	-0.019972216
TCGA-BP-4354-01A-02R-1289-07	C2	-0.209135887
TCGA-BP-4974-01A-01R-1334-07	C2	-0.210558633
TCGA-CJ-4641-01A-02R-1325-07	C3	-0.11667659
TCGA-B4-5834-01A-11R-1672-07	C2	-0.057726239
TCGA-BP-4169-01A-02R-1289-07	C2	-0.024005347
TCGA-B2-5633-01B-04R-A277-07	C1	-0.29582728
TCGA-BP-4777-01A-01R-1289-07	C2	0.05495467
TCGA-B0-5399-01A-01R-1503-07	C2	-0.121719742
TCGA-B0-5099-01A-01R-1420-07	C2	0.074781105
TCGA-BP-4166-01A-02R-1289-07	C2	0.31858842
TCGA-CJ-4890-01A-01R-1305-07	C2	-0.337863787
TCGA-B0-5084-01A-01R-1334-07	C2	-0.290088392
TCGA-BP-5168-01A-01R-1420-07	C2	0.239228507
TCGA-BP-4770-01A-01R-1503-07	C3	-0.249553223
TCGA-BP-4332-01A-01R-1289-07	C2	0.031221283
TCGA-B4-5832-01A-11R-1672-07	C1	-0.053050163
TCGA-B0-4849-01A-01R-1277-07	C3	0.035930783
TCGA-B8-5164-01A-01R-1420-07	C2	0.12779752
TCGA-CW-6093-01A-11R-1672-07	C2	0.127875002
TCGA-MM-A84U-01A-11R-A37O-07	C3	-0.115336537
TCGA-CJ-4637-01A-02R-1325-07	C3	-0.016660202
TCGA-CJ-4642-01B-01R-1305-07	C2	-0.334233049
TCGA-BP-5178-01A-01R-1426-07	C3	-0.284156383
TCGA-BP-4790-01A-01R-1305-07	C2	-0.119380687
TCGA-B0-5692-01A-11R-1541-07	C3	-0.055525147
TCGA-CJ-4885-01A-01R-1305-07	C2	0.211040686
TCGA-BP-4768-01A-01R-1289-07	C2	0.123665243
TCGA-B0-5713-01A-11R-1672-07	C2	0.294290153
TCGA-CW-5587-01A-01R-1541-07	C2	0.123593839
TCGA-BP-5201-01A-01R-1426-07	C3	-0.37664159
TCGA-CW-5590-01A-01R-1541-07	C2	0.100243894
TCGA-A3-3370-01A-02R-1420-07	C2	-0.098447545
TCGA-B0-4848-01A-01R-1277-07	C3	0.261601841
TCGA-B0-4698-01A-01R-1503-07	C2	-0.028704676
TCGA-BP-4982-01A-01R-1334-07	C2	-0.138074844
TCGA-CJ-4636-01A-02R-1325-07	C2	-0.282121266
TCGA-AK-3455-01A-01R-0864-07	C1	0.285357941
TCGA-A3-A6NN-01A-12R-A33J-07	C2	0.084512496
TCGA-G6-A8L6-01A-11R-A37O-07	C3	-0.112017256
TCGA-B0-5703-01A-11R-1541-07	C2	-0.018577112
TCGA-BP-4176-01A-02R-1289-07	C2	-0.216506087
TCGA-B0-4707-01A-01R-1277-07	C3	-0.273643552
TCGA-CW-5591-01A-01R-1541-07	C2	0.204982713
TCGA-B4-5838-01A-11R-1672-07	C2	0.329794256
TCGA-BP-4775-01A-01R-1289-07	C2	-0.09610656
TCGA-BP-4158-01A-02R-1289-07	C2	0.133653807

TCGA-CJ-5676-01A-11R-1541-07	C3	-0.277269549
TCGA-DV-5565-01A-01R-1541-07	C3	-0.088518565
TCGA-BP-4163-01A-02R-1325-07	C2	0.221096997
TCGA-BP-4327-01A-01R-1289-07	C2	0.148054757
TCGA-BP-5182-01A-01R-1426-07	C3	0.171795266
TCGA-CJ-4884-01A-01R-1305-07	C3	0.005547216
TCGA-CZ-5459-01A-01R-1503-07	C2	-0.266489479
TCGA-B0-5096-01A-01R-1420-07	C2	0.057962025
TCGA-B8-A54J-01A-11R-A33J-07	C2	-0.131638245
TCGA-BP-4987-01A-01R-1334-07	C2	0.114498603
TCGA-B2-5641-01A-01R-1541-07	C2	-0.136648101
TCGA-AK-3426-01A-02R-1325-07	C3	-0.443500483
TCGA-B4-5377-01A-01R-1503-07	C2	0.373436305
TCGA-BP-4342-01A-01R-1289-07	C2	-0.279448311
TCGA-BP-4983-01A-01R-1334-07	C2	-0.062704158
TCGA-B0-4833-01A-01R-1305-07	C3	-0.209742992
TCGA-AS-3777-01A-01R-0864-07	C1	0.020696387
TCGA-CJ-4874-01A-01R-1305-07	C2	0.214593499
TCGA-CJ-4887-01A-01R-1305-07	C3	-0.087134299
TCGA-AK-3428-01A-02R-1277-07	C2	0.210086263
TCGA-B8-A54F-01A-11R-A266-07	C2	-0.186309069
TCGA-BP-5187-01A-01R-1426-07	C2	-0.372242502
TCGA-CJ-5681-01A-11R-1541-07	C2	0.369953456
TCGA-B0-4696-01A-01R-1277-07	C2	-0.1373644
TCGA-B0-5710-01A-11R-1672-07	C2	-0.240352152
TCGA-BP-5175-01A-01R-1426-07	C3	-0.372066408
TCGA-DV-5569-01A-01R-1541-07	C2	-0.193154935
TCGA-BP-5189-01A-02R-1426-07	C2	-0.184337428
TCGA-AK-3443-01A-02R-1325-07	C1	0.412703051
TCGA-CW-5589-01A-01R-1541-07	C2	-0.031774764
TCGA-B0-5109-01A-02R-1420-07	C3	-0.094838778
TCGA-B8-A8YJ-01A-13R-A39I-07	C2	-0.281329946
TCGA-B0-4810-01A-01R-1503-07	C2	-0.148979745
TCGA-BP-5001-01A-01R-1334-07	C3	-0.160116735
TCGA-EU-5906-01A-11R-1672-07	C2	-0.113752108
TCGA-BP-4993-01A-02R-1420-07	C2	-0.196206059
TCGA-B0-4823-01A-02R-1420-07	C2	-0.00184817
TCGA-DV-5576-01A-01R-1541-07	C2	-0.148650585
TCGA-MM-A563-01A-11R-A266-07	C3	-0.20753182
TCGA-BP-5192-01A-01R-1426-07	C2	0.169914106
TCGA-CJ-4868-01A-01R-1305-07	C3	-0.229656759
TCGA-A3-A8OU-01A-11R-A37O-07	C2	-0.055625342
TCGA-B0-4718-01A-01R-1277-07	C3	-0.171704384
TCGA-DV-5568-01A-01R-1541-07	C3	-0.114233325
TCGA-AK-3456-01A-02R-1325-07	C2	0.447884785
TCGA-B0-4837-01A-01R-1305-07	C3	-0.257218689
TCGA-B0-4694-01A-01R-1277-07	C3	0.073624075
TCGA-BP-4334-01A-01R-1289-07	C1	0.211329236
TCGA-B8-4146-01B-11R-1672-07	C1	-0.037176125

TCGA-CZ-4854-01A-01R-1305-07	C2	0.007608276
TCGA-DV-5575-01A-01R-1541-07	C2	-0.110554902

Table S7: Expression of mTOR gene in KIRC

	TCGA-A3-	TCGA-A3-	TCGA-B8-	TCGA-A3-	TCGA-A3-	TCGA-B0-	TCGA-CJ-	TCGA-CJ-
PRKAB1	DOWN	NOCHAN(	NOCHAN(	NOCHAN(	NOCHAN(	DOWN	DOWN	NOCHAN(
YWHAB	DOWN	DOWN	NOCHAN(	NOCHAN(	DOWN	DOWN	DOWN	NOCHAN(
CAB39	DOWN	DOWN	DOWN	DOWN	DOWN	DOWN	DOWN	DOWN
STK11	NOCHAN(	NOCHAN(	NOCHAN(	NOCHAN(	NOCHAN(	UP	UP	NOCHAN(
STRADA	NOCHAN(	NOCHAN(	NOCHAN(	UP	NOCHAN(	NOCHAN(	NOCHAN(	NOCHAN(
EIF4E	NOCHAN(	NOCHAN(	DOWN	DOWN	DOWN	DOWN	DOWN	DOWN
PRKAG2	NOCHAN(	NOCHAN(	NOCHAN(	DOWN	NOCHAN(	NOCHAN(	DOWN	DOWN
RPTOR	DOWN	NOCHAN(	UP	NOCHAN(	NOCHAN(	NOCHAN(	NOCHAN(	NOCHAN(
AKT1S1	NOCHAN(	UP	NOCHAN(	UP	UP	UP	UP	UP
PRKAA2	NOCHAN(	NOCHAN(	DOWN	DOWN	NOCHAN(	DOWN	DOWN	DOWN
PRKAG3	NOCHAN(	UP	NOCHAN(	NOCHAN(	NOCHAN(	NOCHAN(	NOCHAN(	NOCHAN(
LAMTOR1	NOCHAN(	NOCHAN(	NOCHAN(	NOCHAN(	NOCHAN(	NOCHAN(	NOCHAN(	NOCHAN(
STRADB	NOCHAN(	NOCHAN(	UP	NOCHAN(	NOCHAN(	DOWN	NOCHAN(	NOCHAN(
MLST8	UP	NOCHAN(	UP	NOCHAN(	NOCHAN(	NOCHAN(	NOCHAN(	NOCHAN(
EIF4G1	NOCHAN(	NOCHAN(	NOCHAN(	NOCHAN(	NOCHAN(	DOWN	NOCHAN(	NOCHAN(
RPS6	UP	NOCHAN(	NOCHAN(	DOWN	UP	UP	NOCHAN(	NOCHAN(
TSC1	NOCHAN(	NOCHAN(	NOCHAN(	NOCHAN(	UP	NOCHAN(	DOWN	NOCHAN(
EEF2K	NOCHAN(	NOCHAN(	UP	UP	NOCHAN(	NOCHAN(	NOCHAN(	NOCHAN(
LAMTOR3	NOCHAN(	NOCHAN(	DOWN	DOWN	NOCHAN(	DOWN	NOCHAN(	NOCHAN(
RRAGB	DOWN	NOCHAN(	DOWN	NOCHAN(	NOCHAN(	DOWN	DOWN	NOCHAN(
PPM1A	DOWN	NOCHAN(	DOWN	DOWN	NOCHAN(	DOWN	DOWN	DOWN
LAMTOR5	NOCHAN(	NOCHAN(	DOWN	DOWN	NOCHAN(	DOWN	DOWN	NOCHAN(
EIF4EBP1	UP	UP	UP	UP	UP	UP	UP	UP
PRKAB2	NOCHAN(	NOCHAN(	NOCHAN(	NOCHAN(	UP	DOWN	DOWN	NOCHAN(
SLC38A9	NOCHAN(	NOCHAN(	NOCHAN(	NOCHAN(	NOCHAN(	DOWN	DOWN	NOCHAN(
AKT2	NOCHAN(	NOCHAN(	NOCHAN(	NOCHAN(	NOCHAN(	NOCHAN(	DOWN	NOCHAN(
MTOR	DOWN	DOWN	NOCHAN(	NOCHAN(	DOWN	DOWN	DOWN	DOWN
PRKAG1	UP	NOCHAN(	NOCHAN(	NOCHAN(	NOCHAN(	DOWN	NOCHAN(	NOCHAN(
AKT1	NOCHAN(	NOCHAN(	NOCHAN(	NOCHAN(	NOCHAN(	UP	NOCHAN(	NOCHAN(
RHEB	NOCHAN(	NOCHAN(	NOCHAN(	DOWN	NOCHAN(	NOCHAN(	NOCHAN(	NOCHAN(
LAMTOR2	UP	NOCHAN(	NOCHAN(	NOCHAN(	NOCHAN(	UP	UP	NOCHAN(
PRKAA1	NOCHAN(	NOCHAN(	NOCHAN(	DOWN	NOCHAN(	DOWN	DOWN	DOWN
RPS6KB1	NOCHAN(	UP	UP	UP	NOCHAN(	NOCHAN(	NOCHAN(	UP
LAMTOR4	UP	NOCHAN(	NOCHAN(	DOWN	NOCHAN(	UP	NOCHAN(	NOCHAN(
RRAGA	NOCHAN(	NOCHAN(	UP	NOCHAN(	NOCHAN(	NOCHAN(	UP	NOCHAN(
RRAGD	NOCHAN(	NOCHAN(	NOCHAN(	NOCHAN(	NOCHAN(	DOWN	DOWN	NOCHAN(
CAB39L	NOCHAN(	DOWN	DOWN	DOWN	DOWN	NOCHAN(	DOWN	DOWN
RRAGC	UP	UP	NOCHAN(	UP	UP	NOCHAN(	UP	UP
EIF4B	UP	UP	NOCHAN(	DOWN	UP	NOCHAN(	NOCHAN(	NOCHAN(
TSC2	NOCHAN(	NOCHAN(	NOCHAN(	NOCHAN(	UP	NOCHAN(	NOCHAN(	NOCHAN(

TCGA-B0-	TCGA-B0-	TCGA-B0-	TCGA-A3-	TCGA-BP-	TCGA-B0-	TCGA-BP-	TCGA-B8-	TCGA-BP-
NOCHAN(	DOWN	DOWN	DOWN	NOCHAN(	NOCHAN(	DOWN	DOWN	DOWN
NOCHAN(	DOWN	DOWN	NOCHAN(	NOCHAN(	UP	DOWN	DOWN	DOWN
NOCHAN(	DOWN	DOWN	DOWN	NOCHAN(	DOWN	DOWN	DOWN	DOWN
NOCHAN(	NOCHAN(	NOCHAN(	NOCHAN(	NOCHAN(	NOCHAN(	NOCHAN(	NOCHAN(	NOCHAN(
NOCHAN(	NOCHAN(	UP	NOCHAN(	NOCHAN(	NOCHAN(	UP	NOCHAN(	NOCHAN(
DOWN	DOWN	DOWN	NOCHAN(	DOWN	DOWN	DOWN	DOWN	DOWN
NOCHAN(	DOWN	DOWN	DOWN	DOWN	DOWN	NOCHAN(	DOWN	NOCHAN(
NOCHAN(	DOWN	UP	UP	NOCHAN(	UP	DOWN	NOCHAN(	NOCHAN(
NOCHAN(	NOCHAN(	UP	NOCHAN(	NOCHAN(	UP	NOCHAN(	NOCHAN(	NOCHAN(
NOCHAN(	DOWN	DOWN	DOWN	UP	DOWN	DOWN	DOWN	NOCHAN(
NOCHAN(	NOCHAN(	UP	NOCHAN(	NOCHAN(	NOCHAN(	UP	NOCHAN(	NOCHAN(
NOCHAN(	NOCHAN(	NOCHAN(	NOCHAN(	NOCHAN(	NOCHAN(	DOWN	NOCHAN(	DOWN
NOCHAN(	NOCHAN(	NOCHAN(	NOCHAN(	NOCHAN(	NOCHAN(	NOCHAN(	NOCHAN(	NOCHAN(
NOCHAN(	DOWN	UP	NOCHAN(	NOCHAN(	UP	NOCHAN(	NOCHAN(	NOCHAN(
NOCHAN(	DOWN	UP	UP	NOCHAN(	UP	DOWN	NOCHAN(	NOCHAN(
NOCHAN(	NOCHAN(	NOCHAN(	NOCHAN(	NOCHAN(	NOCHAN(	NOCHAN(	NOCHAN(	NOCHAN(
NOCHAN(	NOCHAN(	NOCHAN(	NOCHAN(	NOCHAN(	DOWN	UP	NOCHAN(	NOCHAN(
NOCHAN(	NOCHAN(	UP	UP	NOCHAN(	NOCHAN(	UP	NOCHAN(	UP
NOCHAN(	DOWN	DOWN	DOWN	NOCHAN(	DOWN	DOWN	DOWN	NOCHAN(
DOWN	DOWN	NOCHAN(	NOCHAN(	DOWN	DOWN	NOCHAN(	NOCHAN(	NOCHAN(
NOCHAN(	DOWN	DOWN	NOCHAN(	NOCHAN(	DOWN	DOWN	DOWN	DOWN
NOCHAN(	NOCHAN(	DOWN	DOWN	NOCHAN(	NOCHAN(	DOWN	DOWN	NOCHAN(
NOCHAN(	UP	UP	UP	UP	UP	UP	UP	NOCHAN(
NOCHAN(	DOWN	DOWN	NOCHAN(	NOCHAN(	DOWN	NOCHAN(	NOCHAN(	NOCHAN(
NOCHAN(	DOWN	NOCHAN(	NOCHAN(	NOCHAN(	NOCHAN(	NOCHAN(	NOCHAN(	NOCHAN(
NOCHAN(	DOWN	NOCHAN(	NOCHAN(	NOCHAN(	NOCHAN(	NOCHAN(	DOWN	NOCHAN(
NOCHAN(	DOWN	DOWN	NOCHAN(	DOWN	NOCHAN(	DOWN	DOWN	DOWN
NOCHAN(	DOWN	NOCHAN(	NOCHAN(	NOCHAN(	NOCHAN(	DOWN	DOWN	DOWN
NOCHAN(	NOCHAN(	NOCHAN(	UP	UP	NOCHAN(	NOCHAN(	NOCHAN(	NOCHAN(
DOWN	DOWN	UP	NOCHAN(	NOCHAN(	NOCHAN(	DOWN	NOCHAN(	NOCHAN(
DOWN	NOCHAN(	UP	NOCHAN(	NOCHAN(	UP	NOCHAN(	NOCHAN(	DOWN
NOCHAN(	DOWN	DOWN	NOCHAN(	NOCHAN(	DOWN	DOWN	NOCHAN(	NOCHAN(
NOCHAN(	NOCHAN(	NOCHAN(	UP	NOCHAN(	NOCHAN(	NOCHAN(	UP	NOCHAN(
NOCHAN(	NOCHAN(	NOCHAN(	NOCHAN(	NOCHAN(	UP	NOCHAN(	NOCHAN(	NOCHAN(
NOCHAN(	NOCHAN(	DOWN	NOCHAN(	UP	NOCHAN(	DOWN	NOCHAN(	NOCHAN(
NOCHAN(	NOCHAN(	NOCHAN(	DOWN	NOCHAN(	NOCHAN(	DOWN	DOWN	NOCHAN(
NOCHAN(	DOWN	DOWN	DOWN	DOWN	NOCHAN(	DOWN	DOWN	DOWN
NOCHAN(	NOCHAN(	UP	NOCHAN(	UP	NOCHAN(	NOCHAN(	NOCHAN(	NOCHAN(
UP	NOCHAN(	UP	NOCHAN(	UP	DOWN	DOWN	NOCHAN(	UP
UP	NOCHAN(	UP	UP	NOCHAN(	NOCHAN(	UP	NOCHAN(	UP

TCGA-BP-	TCGA-BP-	TCGA-CJ-	TCGA-CZ-	TCGA-A3-	TCGA-A3-	TCGA-BP-	TCGA-BP-	TCGA-CZ-
DOWN	DOWN	NOCHAN(	NOCHAN(	DOWN	NOCHAN(	DOWN	NOCHAN(	DOWN
NOCHAN(	DOWN	NOCHAN(	DOWN	NOCHAN(	DOWN	NOCHAN(	NOCHAN(	NOCHAN(
DOWN	DOWN	DOWN	DOWN	DOWN	DOWN	DOWN	NOCHAN(	DOWN
NOCHAN(	NOCHAN(	NOCHAN(	NOCHAN(	NOCHAN(	NOCHAN(	UP	UP	NOCHAN(
DOWN	NOCHAN(	NOCHAN(	NOCHAN(	NOCHAN(	NOCHAN(	NOCHAN(	NOCHAN(	DOWN
DOWN	NOCHAN(	NOCHAN(	DOWN	DOWN	DOWN	NOCHAN(	DOWN	DOWN
NOCHAN(	DOWN	DOWN	NOCHAN(	NOCHAN(	NOCHAN(	DOWN	NOCHAN(	NOCHAN(
NOCHAN(	UP	NOCHAN(	NOCHAN(	NOCHAN(	NOCHAN(	NOCHAN(	UP	NOCHAN(
UP	UP	NOCHAN(	UP	NOCHAN(	UP	NOCHAN(	UP	UP
DOWN	DOWN	NOCHAN(	NOCHAN(	DOWN	NOCHAN(	DOWN	NOCHAN(	DOWN
NOCHAN(	NOCHAN(	NOCHAN(	NOCHAN(	NOCHAN(	UP	NOCHAN(	UP	NOCHAN(
NOCHAN(	DOWN	NOCHAN(	NOCHAN(	NOCHAN(	NOCHAN(	NOCHAN(	NOCHAN(	NOCHAN(
NOCHAN(	NOCHAN(	NOCHAN(	NOCHAN(	NOCHAN(	NOCHAN(	DOWN	NOCHAN(	NOCHAN(
NOCHAN(	NOCHAN(	DOWN	NOCHAN(	NOCHAN(	NOCHAN(	NOCHAN(	NOCHAN(	UP
UP	NOCHAN(	NOCHAN(	DOWN	NOCHAN(	NOCHAN(	NOCHAN(	NOCHAN(	UP
NOCHAN(	NOCHAN(	NOCHAN(	NOCHAN(	NOCHAN(	NOCHAN(	UP	DOWN	NOCHAN(
DOWN	NOCHAN(	NOCHAN(	NOCHAN(	NOCHAN(	UP	NOCHAN(	UP	DOWN
UP	NOCHAN(	NOCHAN(	NOCHAN(	UP	UP	UP	UP	NOCHAN(
DOWN	DOWN	DOWN	NOCHAN(	DOWN	DOWN	NOCHAN(	DOWN	DOWN
DOWN	DOWN	DOWN	DOWN	NOCHAN(	DOWN	NOCHAN(	DOWN	DOWN
DOWN	DOWN	DOWN	NOCHAN(	DOWN	DOWN	DOWN	NOCHAN(	DOWN
NOCHAN(	DOWN	DOWN	NOCHAN(	NOCHAN(	NOCHAN(	DOWN	NOCHAN(	NOCHAN(
UP	UP	UP	UP	UP	UP	UP	UP	UP
NOCHAN(	NOCHAN(	NOCHAN(	NOCHAN(	NOCHAN(	NOCHAN(	NOCHAN(	NOCHAN(	NOCHAN(
NOCHAN(	NOCHAN(	NOCHAN(	DOWN	NOCHAN(	NOCHAN(	NOCHAN(	NOCHAN(	DOWN
NOCHAN(	NOCHAN(	DOWN	NOCHAN(	NOCHAN(	NOCHAN(	DOWN	NOCHAN(	NOCHAN(
DOWN	DOWN	NOCHAN(	DOWN	NOCHAN(	DOWN	DOWN	NOCHAN(	DOWN
DOWN	DOWN	NOCHAN(	NOCHAN(	NOCHAN(	NOCHAN(	NOCHAN(	NOCHAN(	NOCHAN(
NOCHAN(	UP	NOCHAN(	NOCHAN(	NOCHAN(	NOCHAN(	UP	UP	NOCHAN(
NOCHAN(	DOWN	NOCHAN(	DOWN	NOCHAN(	DOWN	NOCHAN(	NOCHAN(	NOCHAN(
NOCHAN(	NOCHAN(	NOCHAN(	NOCHAN(	NOCHAN(	NOCHAN(	NOCHAN(	NOCHAN(	NOCHAN(
DOWN	NOCHAN(	NOCHAN(	DOWN	NOCHAN(	NOCHAN(	NOCHAN(	NOCHAN(	DOWN
NOCHAN(	NOCHAN(	UP	NOCHAN(	UP	NOCHAN(	UP	NOCHAN(	NOCHAN(
NOCHAN(	DOWN	NOCHAN(	NOCHAN(	NOCHAN(	NOCHAN(	NOCHAN(	NOCHAN(	NOCHAN(
NOCHAN(	NOCHAN(	UP	NOCHAN(	NOCHAN(	NOCHAN(	UP	DOWN	NOCHAN(
NOCHAN(	DOWN	NOCHAN(	NOCHAN(	NOCHAN(	NOCHAN(	DOWN	NOCHAN(	DOWN
DOWN	DOWN	DOWN	DOWN	DOWN	DOWN	NOCHAN(	NOCHAN(	DOWN
UP	DOWN	UP	UP	UP	NOCHAN(	NOCHAN(	UP	NOCHAN(
NOCHAN(	UP	UP	UP	UP	UP	UP	NOCHAN(	UP
NOCHAN(	NOCHAN(	NOCHAN(	NOCHAN(	NOCHAN(	UP	NOCHAN(	UP	UP

TCGA-CZ-	TCGA-B0-	TCGA-BP-	TCGA-CJ-	TCGA-CJ-	TCGA-EU-	TCGA-B4-	TCGA-A3-	TCGA-BP-
DOWN	NOCHAN(	DOWN	NOCHAN(	DOWN	DOWN	NOCHAN(	DOWN	NOCHAN(
DOWN	DOWN	DOWN	NOCHAN(	DOWN	NOCHAN(	NOCHAN(	DOWN	NOCHAN(
DOWN	DOWN	DOWN	NOCHAN(	DOWN	DOWN	DOWN	DOWN	NOCHAN(
NOCHAN(	UP	NOCHAN(	NOCHAN(	NOCHAN(	NOCHAN(	NOCHAN(	NOCHAN(	NOCHAN(
NOCHAN(	NOCHAN(	NOCHAN(	DOWN	NOCHAN(	NOCHAN(	NOCHAN(	NOCHAN(	NOCHAN(
DOWN	DOWN	DOWN	DOWN	NOCHAN(	DOWN	NOCHAN(	DOWN	NOCHAN(
NOCHAN(	DOWN	DOWN	NOCHAN(	DOWN	NOCHAN(	DOWN	NOCHAN(	NOCHAN(
NOCHAN(	NOCHAN(	NOCHAN(	NOCHAN(	NOCHAN(	NOCHAN(	NOCHAN(	NOCHAN(	NOCHAN(
NOCHAN(	UP	UP	UP	UP	NOCHAN(	UP	NOCHAN(	DOWN
NOCHAN(	DOWN	DOWN	NOCHAN(	DOWN	NOCHAN(	NOCHAN(	NOCHAN(	DOWN
NOCHAN(	NOCHAN(	NOCHAN(	NOCHAN(	NOCHAN(	NOCHAN(	NOCHAN(	NOCHAN(	NOCHAN(
NOCHAN(	UP	NOCHAN(	UP	NOCHAN(	NOCHAN(	NOCHAN(	DOWN	DOWN
NOCHAN(	NOCHAN(	NOCHAN(	NOCHAN(	NOCHAN(	NOCHAN(	NOCHAN(	NOCHAN(	NOCHAN(
NOCHAN(	UP	NOCHAN(	NOCHAN(	NOCHAN(	NOCHAN(	NOCHAN(	NOCHAN(	NOCHAN(
NOCHAN(	NOCHAN(	DOWN	NOCHAN(	NOCHAN(	NOCHAN(	NOCHAN(	NOCHAN(	DOWN
NOCHAN(	NOCHAN(	NOCHAN(	NOCHAN(	NOCHAN(	NOCHAN(	NOCHAN(	NOCHAN(	NOCHAN(
NOCHAN(	DOWN	NOCHAN(	NOCHAN(	DOWN	NOCHAN(	NOCHAN(	UP	NOCHAN(
NOCHAN(	UP	NOCHAN(	NOCHAN(	NOCHAN(	NOCHAN(	UP	UP	NOCHAN(
DOWN	DOWN	DOWN	DOWN	DOWN	DOWN	NOCHAN(	DOWN	DOWN
DOWN	DOWN	DOWN	DOWN	DOWN	DOWN	DOWN	NOCHAN(	DOWN
NOCHAN(	DOWN	DOWN	DOWN	DOWN	NOCHAN(	DOWN	DOWN	DOWN
DOWN	NOCHAN(	NOCHAN(	NOCHAN(	NOCHAN(	NOCHAN(	NOCHAN(	DOWN	DOWN
UP	UP	UP	UP	UP	UP	UP	UP	UP
NOCHAN(	DOWN	DOWN	NOCHAN(	NOCHAN(	NOCHAN(	NOCHAN(	UP	NOCHAN(
NOCHAN(	DOWN	DOWN	NOCHAN(	NOCHAN(	NOCHAN(	NOCHAN(	NOCHAN(	NOCHAN(
NOCHAN(	NOCHAN(	DOWN	NOCHAN(	NOCHAN(	NOCHAN(	NOCHAN(	NOCHAN(	DOWN
DOWN	DOWN	DOWN	DOWN	DOWN	DOWN	NOCHAN(	NOCHAN(	DOWN
NOCHAN(	DOWN	DOWN	NOCHAN(	DOWN	NOCHAN(	NOCHAN(	DOWN	NOCHAN(
NOCHAN(	NOCHAN(	NOCHAN(	NOCHAN(	NOCHAN(	NOCHAN(	NOCHAN(	NOCHAN(	NOCHAN(
NOCHAN(	NOCHAN(	DOWN	NOCHAN(	NOCHAN(	NOCHAN(	NOCHAN(	DOWN	NOCHAN(
NOCHAN(	UP	UP	NOCHAN(	UP	UP	NOCHAN(	DOWN	DOWN
NOCHAN(	DOWN	DOWN	NOCHAN(	DOWN	NOCHAN(	NOCHAN(	NOCHAN(	NOCHAN(
NOCHAN(	NOCHAN(	NOCHAN(	NOCHAN(	UP	NOCHAN(	NOCHAN(	UP	UP
NOCHAN(	UP	NOCHAN(	NOCHAN(	NOCHAN(	NOCHAN(	NOCHAN(	NOCHAN(	NOCHAN(
DOWN	UP	NOCHAN(	UP	UP	UP	UP	DOWN	NOCHAN(
DOWN	NOCHAN(	DOWN	NOCHAN(	DOWN	NOCHAN(	NOCHAN(	NOCHAN(	NOCHAN(
DOWN	DOWN	DOWN	NOCHAN(	DOWN	NOCHAN(	DOWN	DOWN	DOWN
NOCHAN(	UP	NOCHAN(	UP	NOCHAN(	NOCHAN(	NOCHAN(	NOCHAN(	UP
UP	DOWN	DOWN	NOCHAN(	NOCHAN(	UP	NOCHAN(	UP	UP
UP	NOCHAN(	NOCHAN(	NOCHAN(	NOCHAN(	NOCHAN(	NOCHAN(	UP	NOCHAN(

TCGA-B0-	TCGA-CZ-	TCGA-B8-	TCGA-B0-	TCGA-CZ-	TCGA-6D-	TCGA-CJ-	TCGA-BP-	TCGA-EU-
NOCHAN(	DOWN	NOCHAN(	DOWN	NOCHAN(	NOCHAN(	NOCHAN(	DOWN	NOCHAN(
DOWN	DOWN	DOWN	DOWN	NOCHAN(	DOWN	NOCHAN(	NOCHAN(	NOCHAN(
DOWN	DOWN	DOWN	DOWN	NOCHAN(	DOWN	NOCHAN(	DOWN	DOWN
NOCHAN(	NOCHAN(	UP	NOCHAN(	NOCHAN(	UP	NOCHAN(	NOCHAN(	NOCHAN(
NOCHAN(	NOCHAN(	NOCHAN(	NOCHAN(	NOCHAN(	NOCHAN(	NOCHAN(	NOCHAN(	NOCHAN(
DOWN	DOWN	DOWN	DOWN	NOCHAN(	DOWN	NOCHAN(	DOWN	DOWN
NOCHAN(	NOCHAN(	NOCHAN(	DOWN	NOCHAN(	DOWN	NOCHAN(	NOCHAN(	DOWN
NOCHAN(	NOCHAN(	NOCHAN(	NOCHAN(	NOCHAN(	UP	UP	NOCHAN(	NOCHAN(
NOCHAN(	UP	UP	UP	NOCHAN(	UP	NOCHAN(	UP	NOCHAN(
NOCHAN(	NOCHAN(	DOWN	DOWN	UP	DOWN	NOCHAN(	NOCHAN(	NOCHAN(
NOCHAN(	NOCHAN(	NOCHAN(	NOCHAN(	NOCHAN(	NOCHAN(	NOCHAN(	UP	NOCHAN(
DOWN	NOCHAN(	NOCHAN(	NOCHAN(	NOCHAN(	NOCHAN(	NOCHAN(	NOCHAN(	NOCHAN(
NOCHAN(	NOCHAN(	NOCHAN(	UP	NOCHAN(	NOCHAN(	NOCHAN(	NOCHAN(	NOCHAN(
UP	NOCHAN(	NOCHAN(	UP	NOCHAN(	UP	NOCHAN(	NOCHAN(	DOWN
NOCHAN(	NOCHAN(	NOCHAN(	NOCHAN(	DOWN	NOCHAN(	UP	DOWN	NOCHAN(
NOCHAN(	NOCHAN(	NOCHAN(	NOCHAN(	NOCHAN(	NOCHAN(	NOCHAN(	NOCHAN(	NOCHAN(
NOCHAN(	DOWN	NOCHAN(	NOCHAN(	NOCHAN(	UP	NOCHAN(	NOCHAN(	NOCHAN(
NOCHAN(	NOCHAN(	NOCHAN(	NOCHAN(	NOCHAN(	UP	NOCHAN(	NOCHAN(	NOCHAN(
DOWN	DOWN	NOCHAN(	DOWN	NOCHAN(	DOWN	NOCHAN(	DOWN	DOWN
NOCHAN(	NOCHAN(	NOCHAN(	DOWN	DOWN	NOCHAN(	DOWN	DOWN	DOWN
DOWN	DOWN	DOWN	DOWN	DOWN	NOCHAN(	NOCHAN(	DOWN	NOCHAN(
NOCHAN(	NOCHAN(	NOCHAN(	NOCHAN(	NOCHAN(	NOCHAN(	NOCHAN(	NOCHAN(	NOCHAN(
UP	UP	UP	UP	NOCHAN(	UP	UP	UP	UP
DOWN	DOWN	DOWN	DOWN	NOCHAN(	DOWN	NOCHAN(	NOCHAN(	NOCHAN(
NOCHAN(	NOCHAN(	NOCHAN(	DOWN	NOCHAN(	NOCHAN(	NOCHAN(	NOCHAN(	NOCHAN(
NOCHAN(	NOCHAN(	NOCHAN(	NOCHAN(	DOWN	NOCHAN(	NOCHAN(	NOCHAN(	NOCHAN(
DOWN	DOWN	DOWN	DOWN	NOCHAN(	NOCHAN(	NOCHAN(	NOCHAN(	DOWN
NOCHAN(	NOCHAN(	NOCHAN(	NOCHAN(	NOCHAN(	NOCHAN(	NOCHAN(	NOCHAN(	DOWN
UP	NOCHAN(	NOCHAN(	DOWN	DOWN	UP	NOCHAN(	NOCHAN(	NOCHAN(
NOCHAN(	NOCHAN(	NOCHAN(	NOCHAN(	NOCHAN(	NOCHAN(	NOCHAN(	DOWN	DOWN
DOWN	NOCHAN(	NOCHAN(	UP	DOWN	UP	NOCHAN(	NOCHAN(	NOCHAN(
NOCHAN(	NOCHAN(	DOWN	DOWN	UP	DOWN	NOCHAN(	NOCHAN(	NOCHAN(
NOCHAN(	NOCHAN(	NOCHAN(	NOCHAN(	UP	NOCHAN(	NOCHAN(	NOCHAN(	NOCHAN(
NOCHAN(	NOCHAN(	NOCHAN(	NOCHAN(	DOWN	UP	NOCHAN(	NOCHAN(	NOCHAN(
NOCHAN(	NOCHAN(	NOCHAN(	NOCHAN(	NOCHAN(	NOCHAN(	UP	NOCHAN(	NOCHAN(
NOCHAN(	DOWN	NOCHAN(	NOCHAN(	NOCHAN(	NOCHAN(	DOWN	NOCHAN(	NOCHAN(
DOWN	DOWN	DOWN	DOWN	NOCHAN(	NOCHAN(	NOCHAN(	NOCHAN(	DOWN
NOCHAN(	NOCHAN(	UP	UP	UP	UP	NOCHAN(	UP	NOCHAN(
UP	UP	NOCHAN(	NOCHAN(	UP	DOWN	NOCHAN(	UP	UP
UP	NOCHAN(	NOCHAN(	NOCHAN(	NOCHAN(	NOCHAN(	NOCHAN(	NOCHAN(	NOCHAN(

TCGA-B8-	TCGA-BP-	TCGA-B8-	TCGA-B4-	TCGA-CZ-	TCGA-BP-	TCGA-A3-	TCGA-B8-	TCGA-BP-
DOWN	NOCHANG	NOCHANG	NOCHANG	NOCHANG	DOWN	DOWN	DOWN	DOWN
NOCHANG	DOWN	NOCHANG	NOCHANG	DOWN	NOCHANG	NOCHANG	NOCHANG	DOWN
DOWN	DOWN	NOCHANG	DOWN	DOWN	DOWN	DOWN	DOWN	DOWN
NOCHANG	UP	NOCHANG	NOCHANG	UP	NOCHANG	NOCHANG	NOCHANG	NOCHANG
NOCHANG	NOCHANG	NOCHANG	NOCHANG	NOCHANG	NOCHANG	NOCHANG	NOCHANG	DOWN
DOWN	DOWN	NOCHANG	NOCHANG	DOWN	NOCHANG	DOWN	NOCHANG	DOWN
NOCHANG	NOCHANG	UP	NOCHANG	DOWN	NOCHANG	NOCHANG	DOWN	NOCHANG
NOCHANG	DOWN	UP	NOCHANG	NOCHANG	NOCHANG	NOCHANG	NOCHANG	NOCHANG
UP	UP	UP	NOCHANG	UP	NOCHANG	NOCHANG	NOCHANG	NOCHANG
NOCHANG	DOWN	NOCHANG	NOCHANG	NOCHANG	NOCHANG	DOWN	NOCHANG	DOWN
NOCHANG	NOCHANG	NOCHANG	NOCHANG	NOCHANG	NOCHANG	UP	NOCHANG	NOCHANG
NOCHANG	NOCHANG	NOCHANG	NOCHANG	NOCHANG	DOWN	DOWN	UP	NOCHANG
NOCHANG	NOCHANG	NOCHANG	NOCHANG	NOCHANG	NOCHANG	NOCHANG	NOCHANG	NOCHANG
NOCHANG	NOCHANG	UP	NOCHANG	UP	DOWN	NOCHANG	UP	UP
UP	NOCHANG	NOCHANG	NOCHANG	NOCHANG	NOCHANG	NOCHANG	NOCHANG	DOWN
NOCHANG	UP	NOCHANG	NOCHANG	NOCHANG	NOCHANG	NOCHANG	DOWN	UP
DOWN	UP	NOCHANG	NOCHANG	DOWN	UP	NOCHANG	UP	DOWN
NOCHANG	NOCHANG	UP	UP	NOCHANG	UP	UP	UP	NOCHANG
DOWN	DOWN	DOWN	DOWN	NOCHANG	NOCHANG	DOWN	NOCHANG	DOWN
DOWN	DOWN	NOCHANG	NOCHANG	DOWN	NOCHANG	DOWN	DOWN	DOWN
DOWN	DOWN	UP	NOCHANG	DOWN	NOCHANG	DOWN	NOCHANG	DOWN
NOCHANG	NOCHANG	NOCHANG	DOWN	NOCHANG	DOWN	DOWN	NOCHANG	NOCHANG
UP	UP	UP	UP	UP	UP	UP	UP	UP
DOWN	DOWN	DOWN	NOCHANG	DOWN	NOCHANG	NOCHANG	NOCHANG	DOWN
NOCHANG	NOCHANG	NOCHANG	NOCHANG	NOCHANG	NOCHANG	NOCHANG	NOCHANG	DOWN
DOWN	NOCHANG	NOCHANG	NOCHANG	NOCHANG	NOCHANG	NOCHANG	NOCHANG	DOWN
NOCHANG	DOWN	NOCHANG	NOCHANG	DOWN	NOCHANG	DOWN	NOCHANG	DOWN
NOCHANG	NOCHANG	UP	NOCHANG	NOCHANG	NOCHANG	NOCHANG	NOCHANG	NOCHANG
NOCHANG	NOCHANG	NOCHANG	NOCHANG	NOCHANG	NOCHANG	NOCHANG	NOCHANG	NOCHANG
NOCHANG	NOCHANG	UP	NOCHANG	NOCHANG	NOCHANG	NOCHANG	NOCHANG	NOCHANG
NOCHANG	NOCHANG	UP	NOCHANG	UP	DOWN	NOCHANG	NOCHANG	NOCHANG
NOCHANG	DOWN	DOWN	NOCHANG	DOWN	NOCHANG	NOCHANG	DOWN	DOWN
NOCHANG	NOCHANG	NOCHANG	UP	NOCHANG	UP	NOCHANG	UP	NOCHANG
NOCHANG	NOCHANG	NOCHANG	DOWN	NOCHANG	DOWN	NOCHANG	NOCHANG	UP
NOCHANG	UP	UP	UP	NOCHANG	NOCHANG	DOWN	NOCHANG	NOCHANG
DOWN	NOCHANG	UP	NOCHANG	DOWN	NOCHANG	NOCHANG	NOCHANG	DOWN
DOWN	DOWN	NOCHANG	DOWN	DOWN	DOWN	NOCHANG	NOCHANG	DOWN
NOCHANG	NOCHANG	UP	NOCHANG	UP	NOCHANG	NOCHANG	UP	UP
NOCHANG	DOWN	UP	NOCHANG	NOCHANG	UP	UP	DOWN	UP
NOCHANG	UP	NOCHANG	NOCHANG	NOCHANG	UP	NOCHANG	DOWN	NOCHANG

TCGA-A3-	TCGA-B0-	TCGA-CZ-	TCGA-BP-	TCGA-BP-	TCGA-CJ-	TCGA-B0-	TCGA-CZ-	TCGA-B0-
NOCHANG	DOWN	NOCHANG	NOCHANG	DOWN	DOWN	DOWN	DOWN	DOWN
DOWN	NOCHANG	DOWN	DOWN	DOWN	NOCHANG	DOWN	DOWN	DOWN
DOWN	DOWN	DOWN	NOCHANG	DOWN	NOCHANG	DOWN	NOCHANG	DOWN
UP	NOCHANG	NOCHANG	NOCHANG	NOCHANG	NOCHANG	UP	NOCHANG	UP
NOCHANG	NOCHANG	NOCHANG	UP	NOCHANG	NOCHANG	UP	DOWN	UP
DOWN	NOCHANG	DOWN	NOCHANG	DOWN	NOCHANG	DOWN	DOWN	DOWN
DOWN	NOCHANG	NOCHANG	NOCHANG	NOCHANG	NOCHANG	DOWN	NOCHANG	NOCHANG
NOCHANG	NOCHANG	NOCHANG	NOCHANG	NOCHANG	NOCHANG	NOCHANG	UP	NOCHANG
UP	NOCHANG	UP	UP	UP	NOCHANG	UP	NOCHANG	UP
NOCHANG	NOCHANG	NOCHANG	DOWN	NOCHANG	NOCHANG	DOWN	DOWN	DOWN
NOCHANG	NOCHANG	NOCHANG	NOCHANG	NOCHANG	NOCHANG	UP	NOCHANG	UP
NOCHANG	NOCHANG	NOCHANG	NOCHANG	NOCHANG	DOWN	NOCHANG	NOCHANG	NOCHANG
NOCHANG	NOCHANG	NOCHANG	NOCHANG	NOCHANG	NOCHANG	NOCHANG	NOCHANG	NOCHANG
UP	NOCHANG	UP	NOCHANG	UP	NOCHANG	UP	UP	UP
NOCHANG	NOCHANG	DOWN	NOCHANG	DOWN	NOCHANG	NOCHANG	NOCHANG	DOWN
NOCHANG	NOCHANG	NOCHANG	NOCHANG	NOCHANG	NOCHANG	UP	UP	UP
NOCHANG	NOCHANG	NOCHANG	UP	DOWN	NOCHANG	NOCHANG	DOWN	UP
NOCHANG	UP	UP	NOCHANG	NOCHANG	NOCHANG	NOCHANG	NOCHANG	NOCHANG
DOWN	DOWN	NOCHANG	NOCHANG	NOCHANG	NOCHANG	DOWN	DOWN	DOWN
DOWN	DOWN	DOWN	NOCHANG	DOWN	DOWN	DOWN	DOWN	NOCHANG
DOWN	DOWN	NOCHANG	UP	DOWN	NOCHANG	DOWN	DOWN	DOWN
NOCHANG	NOCHANG	NOCHANG	NOCHANG	NOCHANG	NOCHANG	NOCHANG	DOWN	NOCHANG
UP	UP	UP	NOCHANG	UP	UP	UP	UP	UP
DOWN	NOCHANG	NOCHANG	NOCHANG	NOCHANG	NOCHANG	DOWN	DOWN	NOCHANG
NOCHANG	NOCHANG	NOCHANG	NOCHANG	DOWN	NOCHANG	DOWN	NOCHANG	UP
NOCHANG	NOCHANG	NOCHANG	NOCHANG	NOCHANG	NOCHANG	NOCHANG	DOWN	NOCHANG
DOWN	DOWN	DOWN	DOWN	DOWN	DOWN	DOWN	DOWN	DOWN
NOCHANG	NOCHANG	NOCHANG	NOCHANG	NOCHANG	NOCHANG	DOWN	NOCHANG	DOWN
NOCHANG	NOCHANG	NOCHANG	NOCHANG	NOCHANG	NOCHANG	NOCHANG	NOCHANG	UP
DOWN	DOWN	DOWN	NOCHANG	NOCHANG	DOWN	NOCHANG	UP	NOCHANG
NOCHANG	NOCHANG	NOCHANG	NOCHANG	UP	DOWN	UP	DOWN	UP
DOWN	NOCHANG	NOCHANG	NOCHANG	DOWN	UP	NOCHANG	NOCHANG	DOWN
NOCHANG	UP	NOCHANG	UP	NOCHANG	UP	NOCHANG	UP	UP
NOCHANG	NOCHANG	NOCHANG	DOWN	NOCHANG	NOCHANG	NOCHANG	NOCHANG	NOCHANG
UP	NOCHANG	NOCHANG	UP	NOCHANG	UP	UP	UP	NOCHANG
NOCHANG	NOCHANG	NOCHANG	NOCHANG	NOCHANG	NOCHANG	DOWN	NOCHANG	NOCHANG
DOWN	NOCHANG	DOWN	DOWN	DOWN	DOWN	DOWN	DOWN	DOWN
NOCHANG	UP	UP	NOCHANG	UP	UP	NOCHANG	UP	NOCHANG
UP	UP	UP	UP	NOCHANG	UP	NOCHANG	UP	DOWN
UP	NOCHANG	NOCHANG	UP	NOCHANG	UP	UP	NOCHANG	NOCHANG

TCGA-B8-	TCGA-CJ-	TCGA-CJ-	TCGA-BP-	TCGA-B0-	TCGA-BP-	TCGA-BP-	TCGA-BP-	TCGA-B2-
NOCHAN(	NOCHAN(	DOWN	DOWN	NOCHAN(	NOCHAN(	DOWN	NOCHAN(	DOWN
NOCHAN(	DOWN	DOWN	DOWN	DOWN	NOCHAN(	DOWN	DOWN	DOWN
UP	DOWN	DOWN	DOWN	DOWN	NOCHAN(	DOWN	DOWN	NOCHAN(
NOCHAN(	UP	NOCHAN(	NOCHAN(	NOCHAN(	NOCHAN(	UP	NOCHAN(	NOCHAN(
NOCHAN(	NOCHAN(	NOCHAN(	NOCHAN(	NOCHAN(	NOCHAN(	NOCHAN(	NOCHAN(	DOWN
NOCHAN(	DOWN	DOWN	DOWN	DOWN	NOCHAN(	DOWN	DOWN	NOCHAN(
UP	NOCHAN(	DOWN	DOWN	DOWN	DOWN	DOWN	NOCHAN(	DOWN
UP	NOCHAN(	NOCHAN(	NOCHAN(	NOCHAN(	NOCHAN(	NOCHAN(	NOCHAN(	NOCHAN(
NOCHAN(	UP	NOCHAN(	NOCHAN(	UP	NOCHAN(	UP	NOCHAN(	DOWN
DOWN	NOCHAN(	DOWN	NOCHAN(	NOCHAN(	NOCHAN(	DOWN	NOCHAN(	UP
NOCHAN(	UP	UP	NOCHAN(	NOCHAN(	NOCHAN(	NOCHAN(	NOCHAN(	NOCHAN(
NOCHAN(	NOCHAN(	NOCHAN(	NOCHAN(	NOCHAN(	NOCHAN(	NOCHAN(	DOWN	DOWN
NOCHAN(	NOCHAN(	NOCHAN(	NOCHAN(	NOCHAN(	NOCHAN(	NOCHAN(	NOCHAN(	NOCHAN(
NOCHAN(	NOCHAN(	NOCHAN(	NOCHAN(	UP	NOCHAN(	NOCHAN(	NOCHAN(	DOWN
NOCHAN(	NOCHAN(	NOCHAN(	DOWN	NOCHAN(	NOCHAN(	NOCHAN(	NOCHAN(	DOWN
NOCHAN(	NOCHAN(	NOCHAN(	NOCHAN(	UP	NOCHAN(	NOCHAN(	NOCHAN(	NOCHAN(
UP	UP	UP	NOCHAN(	NOCHAN(	NOCHAN(	UP	UP	NOCHAN(
DOWN	NOCHAN(	NOCHAN(	NOCHAN(	NOCHAN(	UP	UP	NOCHAN(	UP
NOCHAN(	NOCHAN(	DOWN	DOWN	NOCHAN(	NOCHAN(	DOWN	DOWN	NOCHAN(
NOCHAN(	DOWN	DOWN	NOCHAN(	NOCHAN(	DOWN	DOWN	DOWN	NOCHAN(
DOWN	NOCHAN(	DOWN	NOCHAN(	DOWN	NOCHAN(	DOWN	DOWN	NOCHAN(
DOWN	NOCHAN(	NOCHAN(	NOCHAN(	NOCHAN(	NOCHAN(	NOCHAN(	NOCHAN(	DOWN
DOWN	UP	UP	UP	UP	NOCHAN(	UP	UP	NOCHAN(
NOCHAN(	NOCHAN(	DOWN	NOCHAN(	DOWN	NOCHAN(	NOCHAN(	NOCHAN(	NOCHAN(
NOCHAN(	NOCHAN(	NOCHAN(	NOCHAN(	NOCHAN(	NOCHAN(	NOCHAN(	NOCHAN(	UP
NOCHAN(	NOCHAN(	NOCHAN(	NOCHAN(	NOCHAN(	NOCHAN(	NOCHAN(	NOCHAN(	DOWN
NOCHAN(	DOWN	DOWN	DOWN	DOWN	DOWN	DOWN	DOWN	NOCHAN(
NOCHAN(	NOCHAN(	NOCHAN(	NOCHAN(	NOCHAN(	NOCHAN(	DOWN	NOCHAN(	NOCHAN(
NOCHAN(	NOCHAN(	NOCHAN(	NOCHAN(	DOWN	NOCHAN(	UP	NOCHAN(	NOCHAN(
UP	DOWN	DOWN	DOWN	NOCHAN(	DOWN	NOCHAN(	NOCHAN(	DOWN
DOWN	NOCHAN(	NOCHAN(	DOWN	UP	NOCHAN(	NOCHAN(	NOCHAN(	NOCHAN(
NOCHAN(	NOCHAN(	DOWN	NOCHAN(	NOCHAN(	NOCHAN(	NOCHAN(	NOCHAN(	UP
NOCHAN(	NOCHAN(	NOCHAN(	UP	NOCHAN(	UP	NOCHAN(	NOCHAN(	UP
NOCHAN(	NOCHAN(	NOCHAN(	NOCHAN(	NOCHAN(	NOCHAN(	NOCHAN(	NOCHAN(	NOCHAN(
DOWN	UP	NOCHAN(	NOCHAN(	UP	NOCHAN(	NOCHAN(	DOWN	NOCHAN(
UP	NOCHAN(	NOCHAN(	NOCHAN(	NOCHAN(	NOCHAN(	DOWN	NOCHAN(	NOCHAN(
NOCHAN(	DOWN	DOWN	DOWN	DOWN	DOWN	DOWN	DOWN	NOCHAN(
NOCHAN(	UP	NOCHAN(	UP	UP	NOCHAN(	NOCHAN(	UP	NOCHAN(
UP	NOCHAN(	NOCHAN(	UP	NOCHAN(	UP	NOCHAN(	UP	UP
UP	UP	NOCHAN(	NOCHAN(	NOCHAN(	NOCHAN(	UP	UP	DOWN

TCGA-B0-	TCGA-G6-	TCGA-B0-	TCGA-A3-	TCGA-BP-	TCGA-BP-	TCGA-CJ-	TCGA-DV	TCGA-BP-
DOWN	DOWN	NOCHAN(	NOCHAN(	NOCHAN(	DOWN	DOWN	NOCHAN(	DOWN
DOWN	DOWN	DOWN	DOWN	DOWN	DOWN	NOCHAN(	DOWN	NOCHAN(
DOWN	DOWN	DOWN	DOWN	DOWN	DOWN	DOWN	DOWN	DOWN
UP	UP	NOCHAN(	NOCHAN(	NOCHAN(	NOCHAN(	NOCHAN(	UP	NOCHAN(
NOCHAN(	NOCHAN(	NOCHAN(	NOCHAN(	NOCHAN(	UP	NOCHAN(	NOCHAN(	NOCHAN(
DOWN	DOWN	DOWN	DOWN	NOCHAN(	DOWN	DOWN	NOCHAN(	NOCHAN(
DOWN	DOWN	DOWN	NOCHAN(	DOWN	UP	DOWN	NOCHAN(	NOCHAN(
UP	NOCHAN(	NOCHAN(	NOCHAN(	NOCHAN(	NOCHAN(	NOCHAN(	DOWN	NOCHAN(
UP	UP	UP	UP	UP	UP	NOCHAN(	NOCHAN(	UP
DOWN	DOWN	DOWN	NOCHAN(	DOWN	DOWN	DOWN	DOWN	NOCHAN(
NOCHAN(	NOCHAN(	UP	UP	NOCHAN(	UP	NOCHAN(	NOCHAN(	NOCHAN(
NOCHAN(	NOCHAN(	NOCHAN(	NOCHAN(	NOCHAN(	DOWN	NOCHAN(	DOWN	NOCHAN(
NOCHAN(	NOCHAN(	NOCHAN(	NOCHAN(	NOCHAN(	DOWN	NOCHAN(	DOWN	NOCHAN(
UP	UP	NOCHAN(	NOCHAN(	NOCHAN(	UP	NOCHAN(	DOWN	UP
NOCHAN(	DOWN	NOCHAN(	DOWN	NOCHAN(	UP	DOWN	NOCHAN(	NOCHAN(
NOCHAN(	NOCHAN(	NOCHAN(	NOCHAN(	UP	UP	UP	NOCHAN(	UP
NOCHAN(	DOWN	UP	NOCHAN(	NOCHAN(	NOCHAN(	NOCHAN(	NOCHAN(	NOCHAN(
NOCHAN(	NOCHAN(	NOCHAN(	NOCHAN(	NOCHAN(	NOCHAN(	UP	NOCHAN(	NOCHAN(
DOWN	DOWN	DOWN	DOWN	NOCHAN(	DOWN	DOWN	DOWN	DOWN
DOWN	DOWN	DOWN	NOCHAN(	DOWN	DOWN	NOCHAN(	NOCHAN(	DOWN
DOWN	DOWN	DOWN	DOWN	DOWN	DOWN	DOWN	DOWN	NOCHAN(
NOCHAN(	NOCHAN(	NOCHAN(	NOCHAN(	NOCHAN(	NOCHAN(	DOWN	NOCHAN(	NOCHAN(
UP	UP	UP	UP	UP	UP	UP	UP	UP
DOWN	DOWN	DOWN	NOCHAN(	DOWN	NOCHAN(	NOCHAN(	NOCHAN(	DOWN
DOWN	DOWN	DOWN	NOCHAN(	DOWN	NOCHAN(	NOCHAN(	NOCHAN(	NOCHAN(
NOCHAN(	DOWN	NOCHAN(	NOCHAN(	NOCHAN(	NOCHAN(	DOWN	NOCHAN(	NOCHAN(
DOWN	DOWN	DOWN	NOCHAN(	DOWN	DOWN	DOWN	DOWN	DOWN
NOCHAN(	DOWN	DOWN	NOCHAN(	NOCHAN(	DOWN	NOCHAN(	DOWN	NOCHAN(
NOCHAN(	NOCHAN(	NOCHAN(	NOCHAN(	UP	NOCHAN(	NOCHAN(	UP	NOCHAN(
NOCHAN(	DOWN	DOWN	NOCHAN(	NOCHAN(	UP	NOCHAN(	NOCHAN(	NOCHAN(
UP	UP	NOCHAN(	NOCHAN(	NOCHAN(	UP	NOCHAN(	NOCHAN(	NOCHAN(
DOWN	DOWN	DOWN	NOCHAN(	NOCHAN(	DOWN	NOCHAN(	DOWN	NOCHAN(
NOCHAN(	NOCHAN(	NOCHAN(	UP	NOCHAN(	NOCHAN(	NOCHAN(	UP	NOCHAN(
NOCHAN(	UP	NOCHAN(	NOCHAN(	NOCHAN(	NOCHAN(	NOCHAN(	NOCHAN(	NOCHAN(
UP	NOCHAN(	NOCHAN(	NOCHAN(	UP	UP	UP	UP	UP
DOWN	NOCHAN(	NOCHAN(	NOCHAN(	NOCHAN(	DOWN	DOWN	DOWN	NOCHAN(
DOWN	DOWN	DOWN	NOCHAN(	DOWN	DOWN	DOWN	DOWN	DOWN
NOCHAN(	NOCHAN(	NOCHAN(	UP	NOCHAN(	NOCHAN(	NOCHAN(	NOCHAN(	NOCHAN(
DOWN	DOWN	NOCHAN(	UP	NOCHAN(	DOWN	NOCHAN(	NOCHAN(	UP
NOCHAN(	NOCHAN(	UP	NOCHAN(	NOCHAN(	UP	NOCHAN(	NOCHAN(	NOCHAN(

TCGA-BP-	TCGA-B0-	TCGA-B0-	TCGA-BP-	TCGA-B0-	TCGA-B0-	TCGA-B0-	TCGA-AK-	TCGA-B8-
DOWN	NOCHAN(	NOCHAN(	DOWN	NOCHAN(	NOCHAN(	NOCHAN(	UP	DOWN
DOWN	NOCHAN(	NOCHAN(	NOCHAN(	DOWN	NOCHAN(	DOWN	DOWN	NOCHAN(
DOWN	DOWN	NOCHAN(	DOWN	NOCHAN(	DOWN	DOWN	DOWN	NOCHAN(
NOCHAN(	UP	NOCHAN(	UP	NOCHAN(	NOCHAN(	NOCHAN(	DOWN	NOCHAN(
NOCHAN(	NOCHAN(	NOCHAN(	UP	NOCHAN(	NOCHAN(	DOWN	DOWN	NOCHAN(
DOWN	NOCHAN(	NOCHAN(	NOCHAN(	NOCHAN(	NOCHAN(	DOWN	UP	DOWN
DOWN	UP	UP	NOCHAN(	NOCHAN(	DOWN	DOWN	UP	DOWN
NOCHAN(	NOCHAN(	UP	NOCHAN(	NOCHAN(	NOCHAN(	UP	DOWN	UP
NOCHAN(	NOCHAN(	NOCHAN(	NOCHAN(	UP	UP	UP	DOWN	UP
DOWN	DOWN	DOWN	DOWN	NOCHAN(	NOCHAN(	DOWN	DOWN	DOWN
NOCHAN(	NOCHAN(	NOCHAN(	UP	UP	NOCHAN(	NOCHAN(	NOCHAN(	NOCHAN(
NOCHAN(	NOCHAN(	NOCHAN(	NOCHAN(	NOCHAN(	NOCHAN(	UP	UP	NOCHAN(
DOWN	DOWN	NOCHAN(	DOWN	NOCHAN(	NOCHAN(	NOCHAN(	DOWN	NOCHAN(
NOCHAN(	NOCHAN(	NOCHAN(	NOCHAN(	NOCHAN(	NOCHAN(	UP	UP	NOCHAN(
DOWN	UP	NOCHAN(	NOCHAN(	UP	NOCHAN(	NOCHAN(	DOWN	UP
NOCHAN(	NOCHAN(	NOCHAN(	NOCHAN(	NOCHAN(	NOCHAN(	NOCHAN(	UP	NOCHAN(
NOCHAN(	NOCHAN(	UP	NOCHAN(	NOCHAN(	UP	DOWN	DOWN	DOWN
NOCHAN(	NOCHAN(	DOWN	NOCHAN(	NOCHAN(	NOCHAN(	NOCHAN(	DOWN	UP
DOWN	NOCHAN(	NOCHAN(	DOWN	DOWN	NOCHAN(	DOWN	UP	DOWN
DOWN	UP	NOCHAN(	DOWN	NOCHAN(	NOCHAN(	DOWN	NOCHAN(	DOWN
DOWN	UP	UP	NOCHAN(	NOCHAN(	DOWN	DOWN	NOCHAN(	DOWN
NOCHAN(	DOWN	DOWN	DOWN	NOCHAN(	NOCHAN(	NOCHAN(	NOCHAN(	NOCHAN(
UP	UP	DOWN	NOCHAN(	UP	NOCHAN(	UP	DOWN	UP
DOWN	NOCHAN(	DOWN	NOCHAN(	NOCHAN(	NOCHAN(	DOWN	NOCHAN(	DOWN
DOWN	UP	NOCHAN(	NOCHAN(	NOCHAN(	NOCHAN(	DOWN	DOWN	NOCHAN(
NOCHAN(	NOCHAN(	DOWN	NOCHAN(	NOCHAN(	NOCHAN(	NOCHAN(	NOCHAN(	NOCHAN(
DOWN	NOCHAN(	NOCHAN(	NOCHAN(	NOCHAN(	DOWN	DOWN	DOWN	NOCHAN(
NOCHAN(	UP	NOCHAN(	NOCHAN(	NOCHAN(	NOCHAN(	DOWN	UP	NOCHAN(
NOCHAN(	UP	NOCHAN(	UP	NOCHAN(	UP	NOCHAN(	NOCHAN(	UP
NOCHAN(	NOCHAN(	NOCHAN(	NOCHAN(	NOCHAN(	NOCHAN(	DOWN	UP	NOCHAN(
NOCHAN(	NOCHAN(	DOWN	DOWN	DOWN	NOCHAN(	UP	UP	UP
DOWN	NOCHAN(	NOCHAN(	NOCHAN(	NOCHAN(	NOCHAN(	DOWN	DOWN	DOWN
NOCHAN(	NOCHAN(	NOCHAN(	UP	UP	UP	DOWN	DOWN	UP
NOCHAN(	NOCHAN(	DOWN	NOCHAN(	NOCHAN(	NOCHAN(	UP	UP	NOCHAN(
DOWN	UP	NOCHAN(	NOCHAN(	UP	UP	DOWN	DOWN	UP
DOWN	NOCHAN(	UP	DOWN	NOCHAN(	NOCHAN(	DOWN	NOCHAN(	DOWN
DOWN	NOCHAN(	NOCHAN(	DOWN	DOWN	NOCHAN(	DOWN	NOCHAN(	DOWN
NOCHAN(	NOCHAN(	NOCHAN(	NOCHAN(	UP	NOCHAN(	NOCHAN(	DOWN	UP
NOCHAN(	NOCHAN(	UP	UP	NOCHAN(	NOCHAN(	DOWN	DOWN	NOCHAN(
NOCHAN(	UP	NOCHAN(	UP	UP	NOCHAN(	DOWN	DOWN	NOCHAN(

TCGA-A3-	TCGA-B0-	TCGA-A3-	TCGA-B0-	TCGA-BP-	TCGA-B8-	TCGA-B8-	TCGA-CJ-	TCGA-CW
DOWN	NOCHAN(	DOWN	DOWN	DOWN	NOCHAN(	DOWN	DOWN	DOWN
NOCHAN(	NOCHAN(	DOWN	DOWN	NOCHAN(	DOWN	DOWN	NOCHAN(	NOCHAN(
DOWN	NOCHAN(	DOWN	DOWN	DOWN	DOWN	DOWN	NOCHAN(	DOWN
NOCHAN(	NOCHAN(	NOCHAN(	NOCHAN(	NOCHAN(	NOCHAN(	NOCHAN(	NOCHAN(	NOCHAN(
NOCHAN(	NOCHAN(	NOCHAN(	NOCHAN(	NOCHAN(	NOCHAN(	DOWN	NOCHAN(	NOCHAN(
DOWN	NOCHAN(	DOWN	DOWN	DOWN	NOCHAN(	DOWN	NOCHAN(	DOWN
NOCHAN(	DOWN	NOCHAN(	NOCHAN(	DOWN	NOCHAN(	DOWN	NOCHAN(	NOCHAN(
NOCHAN(	NOCHAN(	UP	NOCHAN(	NOCHAN(	DOWN	NOCHAN(	NOCHAN(	UP
UP	NOCHAN(	UP	NOCHAN(	NOCHAN(	NOCHAN(	UP	UP	UP
DOWN	NOCHAN(	DOWN	NOCHAN(	DOWN	NOCHAN(	DOWN	DOWN	NOCHAN(
UP	NOCHAN(	NOCHAN(	UP	NOCHAN(	NOCHAN(	UP	NOCHAN(	NOCHAN(
NOCHAN(	NOCHAN(	NOCHAN(	NOCHAN(	NOCHAN(	UP	NOCHAN(	NOCHAN(	NOCHAN(
NOCHAN(	NOCHAN(	NOCHAN(	DOWN	NOCHAN(	NOCHAN(	NOCHAN(	NOCHAN(	NOCHAN(
NOCHAN(	NOCHAN(	UP	NOCHAN(	NOCHAN(	NOCHAN(	NOCHAN(	NOCHAN(	UP
NOCHAN(	NOCHAN(	NOCHAN(	DOWN	NOCHAN(	DOWN	NOCHAN(	NOCHAN(	UP
NOCHAN(	NOCHAN(	NOCHAN(	UP	UP	UP	NOCHAN(	NOCHAN(	NOCHAN(
UP	NOCHAN(	DOWN	NOCHAN(	NOCHAN(	NOCHAN(	NOCHAN(	DOWN	NOCHAN(
UP	NOCHAN(	UP	UP	NOCHAN(	NOCHAN(	NOCHAN(	NOCHAN(	NOCHAN(
DOWN	NOCHAN(	DOWN	DOWN	DOWN	UP	DOWN	NOCHAN(	DOWN
NOCHAN(	DOWN	NOCHAN(	DOWN	DOWN	NOCHAN(	DOWN	NOCHAN(	DOWN
DOWN	NOCHAN(	DOWN	DOWN	DOWN	NOCHAN(	DOWN	DOWN	DOWN
NOCHAN(	NOCHAN(	NOCHAN(	NOCHAN(	NOCHAN(	NOCHAN(	NOCHAN(	NOCHAN(	NOCHAN(
UP	UP	UP	UP	UP	UP	UP	UP	UP
NOCHAN(	NOCHAN(	NOCHAN(	NOCHAN(	DOWN	NOCHAN(	DOWN	NOCHAN(	NOCHAN(
NOCHAN(	NOCHAN(	NOCHAN(	NOCHAN(	DOWN	NOCHAN(	DOWN	NOCHAN(	NOCHAN(
NOCHAN(	NOCHAN(	NOCHAN(	NOCHAN(	DOWN	NOCHAN(	DOWN	DOWN	NOCHAN(
NOCHAN(	DOWN	DOWN	DOWN	DOWN	DOWN	DOWN	DOWN	NOCHAN(
NOCHAN(	NOCHAN(	NOCHAN(	NOCHAN(	NOCHAN(	UP	DOWN	NOCHAN(	NOCHAN(
NOCHAN(	UP	UP	UP	NOCHAN(	NOCHAN(	NOCHAN(	NOCHAN(	NOCHAN(
DOWN	DOWN	NOCHAN(	DOWN	NOCHAN(	NOCHAN(	NOCHAN(	NOCHAN(	NOCHAN(
NOCHAN(	NOCHAN(	UP	NOCHAN(	NOCHAN(	UP	UP	NOCHAN(	NOCHAN(
NOCHAN(	NOCHAN(	DOWN	NOCHAN(	DOWN	NOCHAN(	DOWN	NOCHAN(	NOCHAN(
NOCHAN(	UP	NOCHAN(	NOCHAN(	NOCHAN(	NOCHAN(	NOCHAN(	UP	NOCHAN(
NOCHAN(	NOCHAN(	UP	NOCHAN(	NOCHAN(	UP	NOCHAN(	NOCHAN(	NOCHAN(
NOCHAN(	NOCHAN(	NOCHAN(	NOCHAN(	NOCHAN(	NOCHAN(	UP	NOCHAN(	NOCHAN(
NOCHAN(	NOCHAN(	DOWN	NOCHAN(	DOWN	NOCHAN(	NOCHAN(	NOCHAN(	NOCHAN(
DOWN	DOWN	DOWN	DOWN	DOWN	DOWN	DOWN	DOWN	DOWN
NOCHAN(	UP	NOCHAN(	NOCHAN(	NOCHAN(	UP	NOCHAN(	UP	UP
UP	UP	DOWN	UP	NOCHAN(	NOCHAN(	NOCHAN(	NOCHAN(	NOCHAN(
UP	UP	NOCHAN(	NOCHAN(	NOCHAN(	DOWN	NOCHAN(	DOWN	UP

TCGA-B0-	TCGA-BP-	TCGA-BP-	TCGA-B8-	TCGA-B2-	TCGA-AK-	TCGA-AS-	TCGA-CW	TCGA-B0-
NOCHAN(	NOCHAN(	NOCHAN(	DOWN	NOCHAN(	DOWN	NOCHAN(	DOWN	DOWN
NOCHAN(	NOCHAN(	NOCHAN(	NOCHAN(	NOCHAN(	DOWN	DOWN	UP	DOWN
DOWN	DOWN	DOWN	DOWN	NOCHAN(	DOWN	DOWN	NOCHAN(	DOWN
NOCHAN(	NOCHAN(	NOCHAN(	NOCHAN(	UP	NOCHAN(	NOCHAN(	NOCHAN(	NOCHAN(
NOCHAN(	NOCHAN(	NOCHAN(	NOCHAN(	DOWN	NOCHAN(	NOCHAN(	NOCHAN(	NOCHAN(
DOWN	NOCHAN(	NOCHAN(	NOCHAN(	UP	DOWN	DOWN	NOCHAN(	NOCHAN(
DOWN	NOCHAN(	DOWN	NOCHAN(	UP	NOCHAN(	NOCHAN(	DOWN	NOCHAN(
NOCHAN(	NOCHAN(	NOCHAN(	NOCHAN(	NOCHAN(	NOCHAN(	NOCHAN(	NOCHAN(	NOCHAN(
UP	UP	UP	NOCHAN(	DOWN	NOCHAN(	UP	NOCHAN(	NOCHAN(
DOWN	NOCHAN(	DOWN	NOCHAN(	NOCHAN(	DOWN	NOCHAN(	DOWN	DOWN
NOCHAN(	NOCHAN(	NOCHAN(	NOCHAN(	UP	NOCHAN(	NOCHAN(	NOCHAN(	NOCHAN(
UP	NOCHAN(	NOCHAN(	NOCHAN(	NOCHAN(	NOCHAN(	NOCHAN(	NOCHAN(	NOCHAN(
NOCHAN(	NOCHAN(	NOCHAN(	NOCHAN(	NOCHAN(	NOCHAN(	NOCHAN(	NOCHAN(	DOWN
UP	NOCHAN(	UP	NOCHAN(	NOCHAN(	NOCHAN(	NOCHAN(	NOCHAN(	NOCHAN(
NOCHAN(	NOCHAN(	UP	NOCHAN(	NOCHAN(	NOCHAN(	NOCHAN(	UP	NOCHAN(
NOCHAN(	NOCHAN(	NOCHAN(	NOCHAN(	UP	UP	NOCHAN(	NOCHAN(	NOCHAN(
DOWN	UP	NOCHAN(	NOCHAN(	UP	DOWN	UP	NOCHAN(	NOCHAN(
NOCHAN(	NOCHAN(	UP	UP	NOCHAN(	NOCHAN(	NOCHAN(	UP	NOCHAN(
DOWN	NOCHAN(	DOWN	DOWN	UP	DOWN	DOWN	NOCHAN(	DOWN
DOWN	NOCHAN(	DOWN	DOWN	UP	DOWN	NOCHAN(	DOWN	DOWN
NOCHAN(	NOCHAN(	NOCHAN(	NOCHAN(	UP	DOWN	DOWN	DOWN	DOWN
NOCHAN(	NOCHAN(	NOCHAN(	NOCHAN(	DOWN	NOCHAN(	NOCHAN(	DOWN	DOWN
UP	UP	UP	NOCHAN(	DOWN	UP	UP	UP	UP
DOWN	NOCHAN(	NOCHAN(	NOCHAN(	UP	NOCHAN(	NOCHAN(	DOWN	NOCHAN(
DOWN	NOCHAN(	NOCHAN(	NOCHAN(	UP	NOCHAN(	NOCHAN(	NOCHAN(	NOCHAN(
NOCHAN(	NOCHAN(	NOCHAN(	DOWN	UP	NOCHAN(	NOCHAN(	DOWN	NOCHAN(
DOWN	DOWN	NOCHAN(	NOCHAN(	NOCHAN(	DOWN	DOWN	NOCHAN(	DOWN
UP	NOCHAN(	NOCHAN(	NOCHAN(	UP	NOCHAN(	NOCHAN(	NOCHAN(	DOWN
NOCHAN(	NOCHAN(	UP	NOCHAN(	UP	UP	UP	NOCHAN(	NOCHAN(
NOCHAN(	NOCHAN(	NOCHAN(	DOWN	UP	NOCHAN(	DOWN	UP	NOCHAN(
NOCHAN(	NOCHAN(	NOCHAN(	DOWN	DOWN	NOCHAN(	NOCHAN(	NOCHAN(	NOCHAN(
DOWN	NOCHAN(	DOWN	NOCHAN(	UP	DOWN	NOCHAN(	NOCHAN(	NOCHAN(
NOCHAN(	UP	NOCHAN(	NOCHAN(	NOCHAN(	NOCHAN(	NOCHAN(	NOCHAN(	UP
NOCHAN(	NOCHAN(	NOCHAN(	NOCHAN(	UP	UP	NOCHAN(	NOCHAN(	NOCHAN(
UP	UP	NOCHAN(	NOCHAN(	NOCHAN(	NOCHAN(	UP	DOWN	NOCHAN(
NOCHAN(	NOCHAN(	DOWN	NOCHAN(	UP	NOCHAN(	NOCHAN(	NOCHAN(	DOWN
DOWN	DOWN	DOWN	NOCHAN(	UP	DOWN	DOWN	DOWN	DOWN
UP	NOCHAN(	NOCHAN(	NOCHAN(	DOWN	UP	UP	UP	NOCHAN(
UP	UP	UP	UP	UP	UP	UP	NOCHAN(	NOCHAN(
NOCHAN(	UP	UP	UP	NOCHAN(	NOCHAN(	UP	NOCHAN(	NOCHAN(

TCGA-B0-	TCGA-B0-	TCGA-AK-	TCGA-CJ-	TCGA-BP-	TCGA-B4-	TCGA-A3-	TCGA-BP-	TCGA-BP-
NOCHAN(	NOCHAN(	NOCHAN(	DOWN	DOWN	DOWN	NOCHAN(	DOWN	DOWN
DOWN	DOWN	NOCHAN(	NOCHAN(	NOCHAN(	DOWN	NOCHAN(	DOWN	NOCHAN(
DOWN	DOWN	DOWN	DOWN	DOWN	DOWN	DOWN	DOWN	NOCHAN(
NOCHAN(	NOCHAN(	UP	NOCHAN(	NOCHAN(	NOCHAN(	NOCHAN(	UP	NOCHAN(
NOCHAN(	NOCHAN(	NOCHAN(	NOCHAN(	NOCHAN(	NOCHAN(	NOCHAN(	NOCHAN(	NOCHAN(
DOWN	DOWN	NOCHAN(	DOWN	NOCHAN(	NOCHAN(	DOWN	DOWN	NOCHAN(
NOCHAN(	NOCHAN(	UP	DOWN	DOWN	DOWN	NOCHAN(	DOWN	NOCHAN(
NOCHAN(	NOCHAN(	NOCHAN(	NOCHAN(	NOCHAN(	UP	NOCHAN(	DOWN	NOCHAN(
UP	UP	NOCHAN(	NOCHAN(	NOCHAN(	UP	NOCHAN(	UP	NOCHAN(
NOCHAN(	NOCHAN(	DOWN	DOWN	NOCHAN(	DOWN	NOCHAN(	DOWN	NOCHAN(
UP	UP	NOCHAN(	NOCHAN(	UP	NOCHAN(	NOCHAN(	UP	NOCHAN(
NOCHAN(	NOCHAN(	NOCHAN(	NOCHAN(	NOCHAN(	UP	NOCHAN(	NOCHAN(	NOCHAN(
NOCHAN(	NOCHAN(	NOCHAN(	NOCHAN(	NOCHAN(	UP	NOCHAN(	NOCHAN(	NOCHAN(
NOCHAN(	UP	NOCHAN(	NOCHAN(	NOCHAN(	UP	NOCHAN(	UP	NOCHAN(
NOCHAN(	NOCHAN(	NOCHAN(	NOCHAN(	DOWN	UP	NOCHAN(	DOWN	NOCHAN(
NOCHAN(	UP	NOCHAN(	UP	NOCHAN(	NOCHAN(	NOCHAN(	NOCHAN(	UP
NOCHAN(	UP	NOCHAN(	NOCHAN(	UP	NOCHAN(	NOCHAN(	NOCHAN(	NOCHAN(
UP	NOCHAN(	NOCHAN(	NOCHAN(	NOCHAN(	UP	UP	NOCHAN(	NOCHAN(
NOCHAN(	DOWN	UP	DOWN	DOWN	NOCHAN(	DOWN	DOWN	DOWN
NOCHAN(	NOCHAN(	NOCHAN(	DOWN	NOCHAN(	NOCHAN(	NOCHAN(	DOWN	DOWN
NOCHAN(	DOWN	UP	DOWN	NOCHAN(	NOCHAN(	NOCHAN(	DOWN	NOCHAN(
NOCHAN(	NOCHAN(	DOWN	DOWN	NOCHAN(	NOCHAN(	DOWN	NOCHAN(	NOCHAN(
UP	UP	DOWN	UP	UP	UP	NOCHAN(	UP	UP
NOCHAN(	NOCHAN(	NOCHAN(	NOCHAN(	NOCHAN(	DOWN	NOCHAN(	DOWN	DOWN
NOCHAN(	NOCHAN(	NOCHAN(	NOCHAN(	NOCHAN(	UP	NOCHAN(	DOWN	NOCHAN(
NOCHAN(	NOCHAN(	NOCHAN(	NOCHAN(	NOCHAN(	NOCHAN(	NOCHAN(	NOCHAN(	NOCHAN(
DOWN	DOWN	NOCHAN(	DOWN	DOWN	DOWN	NOCHAN(	DOWN	DOWN
NOCHAN(	NOCHAN(	UP	DOWN	NOCHAN(	NOCHAN(	NOCHAN(	DOWN	NOCHAN(
NOCHAN(	NOCHAN(	UP	NOCHAN(	NOCHAN(	NOCHAN(	NOCHAN(	DOWN	NOCHAN(
DOWN	NOCHAN(	NOCHAN(	DOWN	DOWN	UP	NOCHAN(	NOCHAN(	NOCHAN(
NOCHAN(	NOCHAN(	NOCHAN(	NOCHAN(	NOCHAN(	UP	DOWN	NOCHAN(	NOCHAN(
NOCHAN(	NOCHAN(	NOCHAN(	NOCHAN(	NOCHAN(	DOWN	NOCHAN(	DOWN	NOCHAN(
NOCHAN(	UP	NOCHAN(	NOCHAN(	UP	UP	UP	NOCHAN(	NOCHAN(
NOCHAN(	NOCHAN(	NOCHAN(	NOCHAN(	NOCHAN(	UP	DOWN	NOCHAN(	NOCHAN(
UP	NOCHAN(	NOCHAN(	NOCHAN(	NOCHAN(	NOCHAN(	DOWN	NOCHAN(	NOCHAN(
NOCHAN(	NOCHAN(	NOCHAN(	NOCHAN(	NOCHAN(	NOCHAN(	DOWN	DOWN	NOCHAN(
DOWN	DOWN	NOCHAN(	DOWN	NOCHAN(	DOWN	DOWN	DOWN	NOCHAN(
UP	UP	NOCHAN(	NOCHAN(	NOCHAN(	UP	UP	NOCHAN(	NOCHAN(
UP	UP	NOCHAN(	UP	UP	DOWN	UP	DOWN	UP
NOCHAN(	UP	UP	NOCHAN(	NOCHAN(	NOCHAN(	UP	UP	NOCHAN(

TCGA-CJ-	TCGA-BP-	TCGA-B2-	TCGA-A3-	TCGA-BP-	TCGA-BP-	TCGA-A3-	TCGA-BP-	TCGA-A3-
NOCHANG	DOWN	DOWN	DOWN	NOCHANG	NOCHANG	NOCHANG	DOWN	NOCHANG
NOCHANG	DOWN	NOCHANG	NOCHANG	NOCHANG	DOWN	NOCHANG	NOCHANG	NOCHANG
DOWN	DOWN	DOWN	DOWN	DOWN	DOWN	DOWN	NOCHANG	DOWN
NOCHANG	NOCHANG	NOCHANG	NOCHANG	NOCHANG	NOCHANG	NOCHANG	NOCHANG	NOCHANG
NOCHANG	NOCHANG	NOCHANG	NOCHANG	NOCHANG	NOCHANG	NOCHANG	NOCHANG	NOCHANG
DOWN	NOCHANG	DOWN	DOWN	DOWN	DOWN	NOCHANG	DOWN	NOCHANG
DOWN	NOCHANG	DOWN	DOWN	DOWN	NOCHANG	DOWN	NOCHANG	NOCHANG
NOCHANG	NOCHANG	NOCHANG	NOCHANG	NOCHANG	NOCHANG	NOCHANG	NOCHANG	NOCHANG
NOCHANG	NOCHANG	UP	UP	UP	NOCHANG	UP	NOCHANG	NOCHANG
NOCHANG	NOCHANG	NOCHANG	NOCHANG	DOWN	NOCHANG	NOCHANG	NOCHANG	NOCHANG
UP	NOCHANG	NOCHANG	NOCHANG	NOCHANG	NOCHANG	NOCHANG	NOCHANG	NOCHANG
NOCHANG	NOCHANG	NOCHANG	NOCHANG	NOCHANG	NOCHANG	NOCHANG	NOCHANG	NOCHANG
NOCHANG	NOCHANG	NOCHANG	NOCHANG	NOCHANG	NOCHANG	NOCHANG	NOCHANG	NOCHANG
NOCHANG	UP	NOCHANG	UP	UP	NOCHANG	NOCHANG	NOCHANG	NOCHANG
NOCHANG	NOCHANG	NOCHANG	NOCHANG	DOWN	NOCHANG	NOCHANG	NOCHANG	DOWN
NOCHANG	NOCHANG	DOWN	NOCHANG	UP	NOCHANG	NOCHANG	NOCHANG	UP
UP	UP	NOCHANG	NOCHANG	NOCHANG	UP	NOCHANG	NOCHANG	NOCHANG
UP	UP	NOCHANG	UP	NOCHANG	NOCHANG	NOCHANG	NOCHANG	UP
DOWN	NOCHANG	DOWN	DOWN	DOWN	NOCHANG	DOWN	DOWN	NOCHANG
NOCHANG	NOCHANG	DOWN	DOWN	DOWN	DOWN	DOWN	DOWN	NOCHANG
NOCHANG	NOCHANG	DOWN	DOWN	DOWN	DOWN	NOCHANG	DOWN	NOCHANG
NOCHANG	NOCHANG	DOWN	NOCHANG	DOWN	NOCHANG	NOCHANG	DOWN	NOCHANG
UP	UP	UP	UP	UP	UP	UP	UP	NOCHANG
NOCHANG	NOCHANG	NOCHANG	NOCHANG	NOCHANG	NOCHANG	NOCHANG	NOCHANG	NOCHANG
NOCHANG	NOCHANG	NOCHANG	NOCHANG	NOCHANG	NOCHANG	NOCHANG	NOCHANG	NOCHANG
NOCHANG	NOCHANG	NOCHANG	NOCHANG	DOWN	NOCHANG	NOCHANG	NOCHANG	NOCHANG
NOCHANG	DOWN	NOCHANG	DOWN	DOWN	DOWN	NOCHANG	DOWN	DOWN
NOCHANG	NOCHANG	NOCHANG	NOCHANG	NOCHANG	NOCHANG	NOCHANG	NOCHANG	NOCHANG
NOCHANG	NOCHANG	NOCHANG	NOCHANG	NOCHANG	NOCHANG	NOCHANG	NOCHANG	NOCHANG
NOCHANG	NOCHANG	DOWN	NOCHANG	NOCHANG	DOWN	NOCHANG	NOCHANG	NOCHANG
NOCHANG	NOCHANG	NOCHANG	NOCHANG	NOCHANG	NOCHANG	NOCHANG	NOCHANG	NOCHANG
NOCHANG	NOCHANG	NOCHANG	NOCHANG	NOCHANG	NOCHANG	NOCHANG	NOCHANG	NOCHANG
UP	UP	NOCHANG	NOCHANG	NOCHANG	NOCHANG	NOCHANG	UP	UP
NOCHANG	NOCHANG	NOCHANG	NOCHANG	NOCHANG	NOCHANG	NOCHANG	NOCHANG	NOCHANG
NOCHANG	NOCHANG	NOCHANG	NOCHANG	UP	NOCHANG	UP	NOCHANG	UP
NOCHANG	NOCHANG	NOCHANG	NOCHANG	NOCHANG	NOCHANG	NOCHANG	NOCHANG	NOCHANG
DOWN	DOWN	NOCHANG	DOWN	DOWN	NOCHANG	DOWN	NOCHANG	NOCHANG
NOCHANG	NOCHANG	NOCHANG	UP	NOCHANG	NOCHANG	NOCHANG	NOCHANG	UP
NOCHANG	UP	UP	UP	UP	UP	UP	UP	UP
UP	NOCHANG	UP	NOCHANG	NOCHANG	UP	UP	NOCHANG	NOCHANG

TCGA-DV	TCGA-B0-	TCGA-CJ-	TCGA-BP-	TCGA-B2-	TCGA-BP-	TCGA-B2-	TCGA-B2-	TCGA-G6-
DOWN	NOCHAN(	NOCHAN(	NOCHAN(	DOWN	NOCHAN(	DOWN	NOCHAN(	NOCHAN(
DOWN	DOWN	DOWN	NOCHAN(	NOCHAN(	NOCHAN(	NOCHAN(	UP	DOWN
DOWN	DOWN	DOWN	DOWN	NOCHAN(	DOWN	DOWN	NOCHAN(	DOWN
NOCHAN(	UP	NOCHAN(	UP	UP	NOCHAN(	NOCHAN(	NOCHAN(	UP
NOCHAN(	UP	NOCHAN(	UP	NOCHAN(	NOCHAN(	NOCHAN(	NOCHAN(	DOWN
NOCHAN(	DOWN	DOWN	DOWN	NOCHAN(	DOWN	NOCHAN(	UP	DOWN
NOCHAN(	DOWN	NOCHAN(	DOWN	NOCHAN(	NOCHAN(	NOCHAN(	UP	DOWN
NOCHAN(	NOCHAN(	NOCHAN(	NOCHAN(	NOCHAN(	NOCHAN(	DOWN	DOWN	NOCHAN(
NOCHAN(	UP	UP	UP	NOCHAN(	NOCHAN(	UP	DOWN	UP
NOCHAN(	DOWN	NOCHAN(	NOCHAN(	DOWN	UP	DOWN	UP	DOWN
NOCHAN(	NOCHAN(	NOCHAN(	NOCHAN(	NOCHAN(	NOCHAN(	NOCHAN(	NOCHAN(	NOCHAN(
DOWN	NOCHAN(	NOCHAN(	NOCHAN(	NOCHAN(	NOCHAN(	UP	DOWN	NOCHAN(
NOCHAN(	NOCHAN(	NOCHAN(	NOCHAN(	NOCHAN(	NOCHAN(	NOCHAN(	NOCHAN(	NOCHAN(
NOCHAN(	UP	UP	UP	NOCHAN(	NOCHAN(	UP	DOWN	UP
NOCHAN(	NOCHAN(	NOCHAN(	NOCHAN(	NOCHAN(	NOCHAN(	DOWN	DOWN	DOWN
NOCHAN(	NOCHAN(	NOCHAN(	UP	NOCHAN(	NOCHAN(	UP	DOWN	NOCHAN(
NOCHAN(	UP	NOCHAN(	UP	UP	NOCHAN(	NOCHAN(	UP	NOCHAN(
UP	NOCHAN(	UP	NOCHAN(	UP	NOCHAN(	NOCHAN(	NOCHAN(	NOCHAN(
NOCHAN(	DOWN	DOWN	DOWN	DOWN	NOCHAN(	NOCHAN(	UP	DOWN
NOCHAN(	DOWN	DOWN	NOCHAN(	NOCHAN(	DOWN	NOCHAN(	UP	DOWN
NOCHAN(	DOWN	DOWN	DOWN	NOCHAN(	NOCHAN(	DOWN	UP	DOWN
NOCHAN(	NOCHAN(	NOCHAN(	NOCHAN(	DOWN	NOCHAN(	NOCHAN(	DOWN	NOCHAN(
UP	UP	UP	UP	NOCHAN(	UP	UP	DOWN	UP
NOCHAN(	DOWN	DOWN	NOCHAN(	NOCHAN(	NOCHAN(	NOCHAN(	UP	DOWN
DOWN	NOCHAN(	NOCHAN(	NOCHAN(	NOCHAN(	NOCHAN(	NOCHAN(	UP	DOWN
NOCHAN(	NOCHAN(	NOCHAN(	NOCHAN(	NOCHAN(	NOCHAN(	NOCHAN(	NOCHAN(	NOCHAN(
DOWN	DOWN	DOWN	DOWN	NOCHAN(	DOWN	DOWN	NOCHAN(	DOWN
NOCHAN(	NOCHAN(	NOCHAN(	NOCHAN(	NOCHAN(	NOCHAN(	UP	NOCHAN(	DOWN
UP	NOCHAN(	NOCHAN(	NOCHAN(	UP	NOCHAN(	NOCHAN(	DOWN	NOCHAN(
NOCHAN(	NOCHAN(	NOCHAN(	DOWN	DOWN	DOWN	NOCHAN(	NOCHAN(	DOWN
NOCHAN(	UP	NOCHAN(	NOCHAN(	DOWN	DOWN	UP	DOWN	UP
NOCHAN(	DOWN	NOCHAN(	NOCHAN(	NOCHAN(	NOCHAN(	NOCHAN(	UP	DOWN
UP	NOCHAN(	NOCHAN(	NOCHAN(	UP	NOCHAN(	NOCHAN(	UP	DOWN
NOCHAN(	NOCHAN(	UP	NOCHAN(	NOCHAN(	NOCHAN(	UP	DOWN	UP
NOCHAN(	NOCHAN(	UP	UP	NOCHAN(	NOCHAN(	NOCHAN(	DOWN	DOWN
NOCHAN(	NOCHAN(	NOCHAN(	NOCHAN(	NOCHAN(	NOCHAN(	NOCHAN(	UP	NOCHAN(
DOWN	DOWN	DOWN	NOCHAN(	DOWN	NOCHAN(	NOCHAN(	NOCHAN(	DOWN
UP	NOCHAN(	NOCHAN(	NOCHAN(	NOCHAN(	NOCHAN(	UP	NOCHAN(	NOCHAN(
NOCHAN(	NOCHAN(	NOCHAN(	NOCHAN(	UP	UP	UP	UP	DOWN
NOCHAN(	UP	NOCHAN(	UP	UP	NOCHAN(	NOCHAN(	DOWN	NOCHAN(

TCGA-B0-	TCGA-BP-	TCGA-BP-	TCGA-AK-	TCGA-CZ-	TCGA-MN	TCGA-BP-	TCGA-DV	TCGA-BP-
DOWN	NOCHAN(	DOWN	NOCHAN(	NOCHAN(	DOWN	DOWN	DOWN	NOCHAN(
NOCHAN(	DOWN	NOCHAN(	DOWN	DOWN	DOWN	DOWN	NOCHAN(	DOWN
NOCHAN(	DOWN	DOWN	DOWN	DOWN	DOWN	DOWN	DOWN	DOWN
NOCHAN(	UP	NOCHAN(	NOCHAN(	NOCHAN(	UP	NOCHAN(	NOCHAN(	NOCHAN(
NOCHAN(	NOCHAN(	NOCHAN(	DOWN	NOCHAN(	NOCHAN(	NOCHAN(	NOCHAN(	NOCHAN(
NOCHAN(	DOWN	NOCHAN(	DOWN	DOWN	DOWN	DOWN	DOWN	DOWN
NOCHAN(	NOCHAN(	DOWN	DOWN	DOWN	NOCHAN(	DOWN	DOWN	DOWN
NOCHAN(	NOCHAN(	NOCHAN(	DOWN	NOCHAN(	DOWN	NOCHAN(	NOCHAN(	NOCHAN(
NOCHAN(	UP	NOCHAN(	UP	UP	NOCHAN(	UP	UP	UP
NOCHAN(	NOCHAN(	DOWN	DOWN	NOCHAN(	DOWN	DOWN	NOCHAN(	DOWN
NOCHAN(	UP	NOCHAN(	NOCHAN(	NOCHAN(	NOCHAN(	NOCHAN(	NOCHAN(	NOCHAN(
NOCHAN(	NOCHAN(	NOCHAN(	UP	NOCHAN(	NOCHAN(	NOCHAN(	NOCHAN(	NOCHAN(
NOCHAN(	NOCHAN(	DOWN	NOCHAN(	NOCHAN(	DOWN	NOCHAN(	NOCHAN(	NOCHAN(
NOCHAN(	NOCHAN(	NOCHAN(	UP	UP	NOCHAN(	UP	UP	NOCHAN(
NOCHAN(	NOCHAN(	NOCHAN(	DOWN	DOWN	DOWN	NOCHAN(	NOCHAN(	NOCHAN(
NOCHAN(	NOCHAN(	NOCHAN(	UP	NOCHAN(	UP	NOCHAN(	NOCHAN(	NOCHAN(
NOCHAN(	UP	NOCHAN(	DOWN	DOWN	NOCHAN(	DOWN	NOCHAN(	NOCHAN(
NOCHAN(	NOCHAN(	NOCHAN(	DOWN	NOCHAN(	NOCHAN(	NOCHAN(	NOCHAN(	NOCHAN(
NOCHAN(	DOWN	DOWN	NOCHAN(	DOWN	DOWN	DOWN	DOWN	DOWN
NOCHAN(	NOCHAN(	NOCHAN(	DOWN	DOWN	DOWN	DOWN	DOWN	DOWN
NOCHAN(	DOWN	DOWN	DOWN	DOWN	DOWN	DOWN	DOWN	DOWN
NOCHAN(	NOCHAN(	DOWN	UP	NOCHAN(	NOCHAN(	NOCHAN(	NOCHAN(	NOCHAN(
UP	UP	NOCHAN(	UP	UP	UP	UP	UP	UP
NOCHAN(	NOCHAN(	NOCHAN(	DOWN	NOCHAN(	NOCHAN(	DOWN	DOWN	DOWN
NOCHAN(	NOCHAN(	NOCHAN(	DOWN	DOWN	NOCHAN(	NOCHAN(	NOCHAN(	NOCHAN(
DOWN	NOCHAN(	NOCHAN(	DOWN	NOCHAN(	NOCHAN(	DOWN	NOCHAN(	DOWN
DOWN	DOWN	NOCHAN(	DOWN	DOWN	DOWN	DOWN	DOWN	DOWN
NOCHAN(	DOWN	NOCHAN(	UP	NOCHAN(	DOWN	NOCHAN(	NOCHAN(	NOCHAN(
NOCHAN(	NOCHAN(	UP	DOWN	DOWN	NOCHAN(	NOCHAN(	UP	DOWN
NOCHAN(	NOCHAN(	NOCHAN(	NOCHAN(	NOCHAN(	NOCHAN(	NOCHAN(	NOCHAN(	DOWN
NOCHAN(	UP	NOCHAN(	UP	UP	UP	NOCHAN(	NOCHAN(	UP
NOCHAN(	DOWN	NOCHAN(	DOWN	DOWN	NOCHAN(	NOCHAN(	DOWN	DOWN
UP	UP	NOCHAN(	NOCHAN(	NOCHAN(	NOCHAN(	NOCHAN(	NOCHAN(	NOCHAN(
NOCHAN(	NOCHAN(	NOCHAN(	UP	NOCHAN(	UP	NOCHAN(	NOCHAN(	NOCHAN(
NOCHAN(	NOCHAN(	NOCHAN(	UP	DOWN	NOCHAN(	NOCHAN(	UP	NOCHAN(
NOCHAN(	NOCHAN(	DOWN	NOCHAN(	NOCHAN(	NOCHAN(	DOWN	DOWN	NOCHAN(
DOWN	DOWN	NOCHAN(	DOWN	DOWN	DOWN	DOWN	DOWN	DOWN
UP	NOCHAN(	NOCHAN(	UP	UP	NOCHAN(	NOCHAN(	UP	NOCHAN(
UP	NOCHAN(	UP	DOWN	NOCHAN(	NOCHAN(	NOCHAN(	NOCHAN(	DOWN
NOCHAN(	UP	UP	DOWN	NOCHAN(	NOCHAN(	NOCHAN(	NOCHAN(	NOCHAN(

TCGA-BP-	TCGA-BP-	TCGA-AK-	TCGA-CZ-	TCGA-A3-	TCGA-B0-	TCGA-CJ-	TCGA-BP-	TCGA-B2-
NOCHAN(	DOWN	NOCHAN(	NOCHAN(	NOCHAN(	NOCHAN(	NOCHAN(	DOWN	NOCHAN(
NOCHAN(	NOCHAN(	DOWN	DOWN	DOWN	DOWN	NOCHAN(	DOWN	NOCHAN(
NOCHAN(	NOCHAN(	DOWN	DOWN	DOWN	DOWN	DOWN	DOWN	DOWN
NOCHAN(	DOWN	NOCHAN(	NOCHAN(	UP	NOCHAN(	NOCHAN(	NOCHAN(	NOCHAN(
NOCHAN(	DOWN	NOCHAN(	NOCHAN(	NOCHAN(	NOCHAN(	NOCHAN(	NOCHAN(	NOCHAN(
NOCHAN(	NOCHAN(	DOWN	DOWN	NOCHAN(	DOWN	NOCHAN(	DOWN	DOWN
NOCHAN(	NOCHAN(	NOCHAN(	NOCHAN(	DOWN	DOWN	DOWN	DOWN	NOCHAN(
NOCHAN(	UP	DOWN	NOCHAN(	NOCHAN(	NOCHAN(	NOCHAN(	NOCHAN(	NOCHAN(
NOCHAN(	DOWN	NOCHAN(	UP	UP	UP	UP	NOCHAN(	UP
NOCHAN(	DOWN	NOCHAN(	NOCHAN(	NOCHAN(	NOCHAN(	DOWN	DOWN	NOCHAN(
NOCHAN(	NOCHAN(	UP	NOCHAN(	NOCHAN(	UP	NOCHAN(	NOCHAN(	NOCHAN(
NOCHAN(	NOCHAN(	UP	NOCHAN(	NOCHAN(	DOWN	NOCHAN(	DOWN	NOCHAN(
NOCHAN(	NOCHAN(	NOCHAN(	UP	NOCHAN(	NOCHAN(	NOCHAN(	NOCHAN(	NOCHAN(
NOCHAN(	NOCHAN(	UP	NOCHAN(	NOCHAN(	NOCHAN(	NOCHAN(	NOCHAN(	NOCHAN(
NOCHAN(	DOWN	DOWN	DOWN	NOCHAN(	DOWN	NOCHAN(	DOWN	NOCHAN(
NOCHAN(	UP	UP	UP	NOCHAN(	NOCHAN(	NOCHAN(	NOCHAN(	NOCHAN(
NOCHAN(	DOWN	NOCHAN(	NOCHAN(	NOCHAN(	NOCHAN(	NOCHAN(	DOWN	NOCHAN(
UP	NOCHAN(	NOCHAN(	UP	NOCHAN(	NOCHAN(	NOCHAN(	NOCHAN(	NOCHAN(
DOWN	NOCHAN(	NOCHAN(	DOWN	DOWN	DOWN	DOWN	DOWN	DOWN
DOWN	NOCHAN(	DOWN	DOWN	NOCHAN(	DOWN	DOWN	DOWN	DOWN
NOCHAN(	NOCHAN(	DOWN	NOCHAN(	DOWN	DOWN	DOWN	DOWN	DOWN
NOCHAN(	NOCHAN(	UP	NOCHAN(	NOCHAN(	NOCHAN(	DOWN	NOCHAN(	NOCHAN(
NOCHAN(	UP	UP	UP	UP	UP	UP	UP	UP
NOCHAN(	NOCHAN(	NOCHAN(	NOCHAN(	NOCHAN(	DOWN	NOCHAN(	DOWN	NOCHAN(
NOCHAN(	NOCHAN(	NOCHAN(	NOCHAN(	NOCHAN(	DOWN	NOCHAN(	DOWN	NOCHAN(
NOCHAN(	DOWN	DOWN	NOCHAN(	NOCHAN(	DOWN	DOWN	DOWN	NOCHAN(
DOWN	DOWN	DOWN	DOWN	NOCHAN(	DOWN	DOWN	DOWN	NOCHAN(
NOCHAN(	UP	UP	NOCHAN(	NOCHAN(	DOWN	NOCHAN(	DOWN	NOCHAN(
NOCHAN(	NOCHAN(	DOWN	NOCHAN(	UP	NOCHAN(	NOCHAN(	NOCHAN(	NOCHAN(
NOCHAN(	UP	NOCHAN(	NOCHAN(	NOCHAN(	NOCHAN(	NOCHAN(	DOWN	DOWN
DOWN	NOCHAN(	UP	NOCHAN(	NOCHAN(	NOCHAN(	NOCHAN(	UP	NOCHAN(
NOCHAN(	UP	NOCHAN(	NOCHAN(	NOCHAN(	DOWN	DOWN	DOWN	NOCHAN(
UP	UP	NOCHAN(	UP	NOCHAN(	NOCHAN(	NOCHAN(	DOWN	NOCHAN(
NOCHAN(	NOCHAN(	UP	NOCHAN(	NOCHAN(	NOCHAN(	NOCHAN(	NOCHAN(	NOCHAN(
NOCHAN(	UP	NOCHAN(	UP	NOCHAN(	DOWN	NOCHAN(	NOCHAN(	NOCHAN(
NOCHAN(	NOCHAN(	NOCHAN(	NOCHAN(	NOCHAN(	NOCHAN(	NOCHAN(	DOWN	NOCHAN(
DOWN	DOWN	NOCHAN(	DOWN	DOWN	DOWN	DOWN	DOWN	DOWN
NOCHAN(	UP	UP	NOCHAN(	NOCHAN(	NOCHAN(	UP	NOCHAN(	NOCHAN(
UP	UP	NOCHAN(	UP	NOCHAN(	DOWN	NOCHAN(	DOWN	NOCHAN(
UP	DOWN	DOWN	NOCHAN(	UP	NOCHAN(	NOCHAN(	DOWN	UP

TCGA-T7-	TCGA-CJ-	TCGA-G6-	TCGA-BP-	TCGA-AK-	TCGA-BP-	TCGA-CZ-	TCGA-B0-	TCGA-CJ-
NOCHAN(	DOWN	NOCHAN(	DOWN	NOCHAN(	DOWN	NOCHAN(	DOWN	DOWN
NOCHAN(	DOWN	DOWN	NOCHAN(	DOWN	NOCHAN(	NOCHAN(	DOWN	DOWN
NOCHAN(	DOWN	DOWN	NOCHAN(	DOWN	DOWN	NOCHAN(	DOWN	DOWN
UP	NOCHAN(	UP	NOCHAN(	NOCHAN(	NOCHAN(	NOCHAN(	UP	NOCHAN(
NOCHAN(	NOCHAN(	NOCHAN(	NOCHAN(	NOCHAN(	NOCHAN(	NOCHAN(	NOCHAN(	NOCHAN(
NOCHAN(	DOWN	DOWN	DOWN	DOWN	NOCHAN(	NOCHAN(	DOWN	DOWN
DOWN	NOCHAN(	DOWN	NOCHAN(	NOCHAN(	DOWN	DOWN	DOWN	DOWN
UP	NOCHAN(	NOCHAN(	NOCHAN(	NOCHAN(	DOWN	NOCHAN(	NOCHAN(	NOCHAN(
UP	NOCHAN(	UP	NOCHAN(	NOCHAN(	NOCHAN(	UP	UP	UP
DOWN	DOWN	DOWN	DOWN	DOWN	NOCHAN(	NOCHAN(	DOWN	DOWN
NOCHAN(	UP	UP	NOCHAN(	NOCHAN(	NOCHAN(	NOCHAN(	NOCHAN(	UP
NOCHAN(	NOCHAN(	NOCHAN(	NOCHAN(	NOCHAN(	NOCHAN(	NOCHAN(	NOCHAN(	NOCHAN(
NOCHAN(	NOCHAN(	NOCHAN(	NOCHAN(	NOCHAN(	NOCHAN(	NOCHAN(	NOCHAN(	NOCHAN(
NOCHAN(	NOCHAN(	NOCHAN(	NOCHAN(	NOCHAN(	NOCHAN(	NOCHAN(	UP	UP
NOCHAN(	NOCHAN(	NOCHAN(	NOCHAN(	NOCHAN(	DOWN	NOCHAN(	NOCHAN(	NOCHAN(
DOWN	NOCHAN(	NOCHAN(	NOCHAN(	UP	UP	NOCHAN(	UP	NOCHAN(
UP	NOCHAN(	UP	NOCHAN(	NOCHAN(	NOCHAN(	DOWN	UP	NOCHAN(
NOCHAN(	NOCHAN(	NOCHAN(	NOCHAN(	NOCHAN(	NOCHAN(	NOCHAN(	NOCHAN(	UP
NOCHAN(	DOWN	NOCHAN(	DOWN	NOCHAN(	NOCHAN(	NOCHAN(	DOWN	DOWN
NOCHAN(	DOWN	NOCHAN(	DOWN	DOWN	NOCHAN(	NOCHAN(	NOCHAN(	DOWN
NOCHAN(	DOWN	DOWN	DOWN	DOWN	NOCHAN(	DOWN	DOWN	DOWN
DOWN	DOWN	NOCHAN(	DOWN	NOCHAN(	NOCHAN(	NOCHAN(	NOCHAN(	DOWN
UP	UP	UP	UP	UP	UP	UP	UP	UP
NOCHAN(	DOWN	NOCHAN(	DOWN	NOCHAN(	NOCHAN(	NOCHAN(	NOCHAN(	NOCHAN(
NOCHAN(	NOCHAN(	NOCHAN(	NOCHAN(	DOWN	DOWN	NOCHAN(	NOCHAN(	NOCHAN(
NOCHAN(	DOWN	NOCHAN(	NOCHAN(	DOWN	DOWN	NOCHAN(	NOCHAN(	NOCHAN(
DOWN	DOWN	DOWN	NOCHAN(	DOWN	DOWN	DOWN	DOWN	DOWN
NOCHAN(	DOWN	NOCHAN(	NOCHAN(	NOCHAN(	NOCHAN(	NOCHAN(	DOWN	NOCHAN(
NOCHAN(	UP	UP	NOCHAN(	NOCHAN(	NOCHAN(	DOWN	NOCHAN(	NOCHAN(
NOCHAN(	NOCHAN(	NOCHAN(	NOCHAN(	NOCHAN(	NOCHAN(	NOCHAN(	NOCHAN(	NOCHAN(
NOCHAN(	NOCHAN(	UP	NOCHAN(	UP	NOCHAN(	UP	NOCHAN(	NOCHAN(
NOCHAN(	DOWN	DOWN	NOCHAN(	NOCHAN(	NOCHAN(	NOCHAN(	DOWN	NOCHAN(
UP	UP	NOCHAN(	NOCHAN(	NOCHAN(	NOCHAN(	UP	NOCHAN(	NOCHAN(
NOCHAN(	NOCHAN(	UP	NOCHAN(	NOCHAN(	NOCHAN(	NOCHAN(	NOCHAN(	NOCHAN(
NOCHAN(	NOCHAN(	UP	NOCHAN(	NOCHAN(	UP	UP	NOCHAN(	NOCHAN(
NOCHAN(	DOWN	NOCHAN(	DOWN	NOCHAN(	NOCHAN(	NOCHAN(	DOWN	NOCHAN(
UP	DOWN	DOWN	DOWN	DOWN	NOCHAN(	NOCHAN(	DOWN	DOWN
NOCHAN(	NOCHAN(	NOCHAN(	NOCHAN(	UP	UP	NOCHAN(	UP	NOCHAN(
DOWN	NOCHAN(	NOCHAN(	NOCHAN(	NOCHAN(	UP	NOCHAN(	NOCHAN(	UP
NOCHAN(	NOCHAN(	UP	NOCHAN(	NOCHAN(	NOCHAN(	NOCHAN(	UP	UP

TCGA-B8-	TCGA-CJ-	TCGA-BP-	TCGA-B2-	TCGA-B2-	TCGA-BP-	TCGA-A3-	TCGA-A3-	TCGA-BP-
NOCHAN	NOCHAN	DOWN	NOCHAN	NOCHAN	DOWN	NOCHAN	NOCHAN	NOCHAN
DOWN	NOCHAN	DOWN	NOCHAN	UP	DOWN	NOCHAN	DOWN	NOCHAN
DOWN	NOCHAN	DOWN	DOWN	DOWN	DOWN	DOWN	DOWN	DOWN
UP	NOCHAN	NOCHAN	NOCHAN	UP	NOCHAN	NOCHAN	UP	NOCHAN
NOCHAN	NOCHAN	NOCHAN	NOCHAN	DOWN	DOWN	NOCHAN	NOCHAN	NOCHAN
DOWN	NOCHAN	DOWN	NOCHAN	UP	DOWN	DOWN	DOWN	DOWN
NOCHAN	DOWN	NOCHAN	NOCHAN	UP	DOWN	NOCHAN	NOCHAN	DOWN
NOCHAN	NOCHAN	NOCHAN	NOCHAN	NOCHAN	NOCHAN	NOCHAN	NOCHAN	UP
UP	NOCHAN	UP	NOCHAN	NOCHAN	UP	NOCHAN	UP	UP
DOWN	NOCHAN	DOWN	NOCHAN	NOCHAN	DOWN	NOCHAN	NOCHAN	DOWN
UP	NOCHAN	UP	NOCHAN	NOCHAN	UP	NOCHAN	UP	NOCHAN
NOCHAN	NOCHAN	NOCHAN	NOCHAN	NOCHAN	DOWN	NOCHAN	NOCHAN	UP
NOCHAN	NOCHAN	DOWN	NOCHAN	DOWN	NOCHAN	NOCHAN	NOCHAN	NOCHAN
UP	NOCHAN	NOCHAN	NOCHAN	UP	NOCHAN	NOCHAN	NOCHAN	UP
NOCHAN	NOCHAN	NOCHAN	NOCHAN	NOCHAN	DOWN	NOCHAN	UP	NOCHAN
NOCHAN	NOCHAN	NOCHAN	UP	NOCHAN	NOCHAN	NOCHAN	NOCHAN	NOCHAN
NOCHAN	NOCHAN	NOCHAN	NOCHAN	UP	NOCHAN	NOCHAN	NOCHAN	NOCHAN
NOCHAN	NOCHAN	NOCHAN	NOCHAN	NOCHAN	NOCHAN	NOCHAN	UP	NOCHAN
DOWN	NOCHAN	DOWN	DOWN	UP	DOWN	NOCHAN	DOWN	DOWN
DOWN	DOWN	DOWN	DOWN	NOCHAN	DOWN	DOWN	NOCHAN	DOWN
DOWN	DOWN	DOWN	NOCHAN	UP	DOWN	NOCHAN	DOWN	DOWN
NOCHAN	NOCHAN	NOCHAN	NOCHAN	DOWN	NOCHAN	NOCHAN	NOCHAN	NOCHAN
UP	UP	UP	UP	DOWN	UP	UP	UP	UP
DOWN	NOCHAN	DOWN	NOCHAN	UP	DOWN	DOWN	NOCHAN	DOWN
NOCHAN	NOCHAN	DOWN	NOCHAN	NOCHAN	DOWN	NOCHAN	NOCHAN	NOCHAN
DOWN	NOCHAN	DOWN	NOCHAN	UP	DOWN	NOCHAN	NOCHAN	NOCHAN
DOWN	DOWN	DOWN	DOWN	NOCHAN	DOWN	DOWN	NOCHAN	DOWN
DOWN	NOCHAN	DOWN	NOCHAN	UP	DOWN	NOCHAN	NOCHAN	NOCHAN
NOCHAN	NOCHAN	NOCHAN	NOCHAN	UP	DOWN	NOCHAN	UP	NOCHAN
NOCHAN	DOWN	NOCHAN	NOCHAN	NOCHAN	DOWN	DOWN	NOCHAN	NOCHAN
UP	NOCHAN	UP	NOCHAN	DOWN	UP	NOCHAN	NOCHAN	UP
DOWN	NOCHAN	DOWN	NOCHAN	UP	DOWN	NOCHAN	NOCHAN	DOWN
NOCHAN	NOCHAN	NOCHAN	NOCHAN	NOCHAN	DOWN	NOCHAN	NOCHAN	NOCHAN
UP	NOCHAN	NOCHAN	NOCHAN	NOCHAN	UP	DOWN	NOCHAN	NOCHAN
NOCHAN	UP	NOCHAN	NOCHAN	DOWN	NOCHAN	UP	NOCHAN	UP
DOWN	DOWN	DOWN	NOCHAN	UP	DOWN	NOCHAN	NOCHAN	NOCHAN
DOWN	NOCHAN	DOWN	DOWN	UP	DOWN	DOWN	NOCHAN	DOWN
NOCHAN	NOCHAN	NOCHAN	NOCHAN	DOWN	NOCHAN	UP	NOCHAN	UP
DOWN	NOCHAN	NOCHAN	UP	UP	DOWN	UP	NOCHAN	NOCHAN
NOCHAN	NOCHAN	UP	NOCHAN	UP	DOWN	NOCHAN	UP	NOCHAN

TCGA-BP-	TCGA-B2-	TCGA-B0-	TCGA-A3-	TCGA-CW	TCGA-B8-	TCGA-BP-	TCGA-B0-	TCGA-CW
NOCHAN(	DOWN	DOWN	UP	NOCHAN(	DOWN	NOCHAN(	DOWN	DOWN
NOCHAN(	NOCHAN(	DOWN	NOCHAN(	DOWN	NOCHAN(	NOCHAN(	NOCHAN(	DOWN
DOWN	NOCHAN(	DOWN	NOCHAN(	DOWN	DOWN	DOWN	DOWN	DOWN
NOCHAN(	NOCHAN(	UP	UP	NOCHAN(	UP	NOCHAN(	NOCHAN(	NOCHAN(
NOCHAN(	NOCHAN(	NOCHAN(	NOCHAN(	NOCHAN(	NOCHAN(	NOCHAN(	NOCHAN(	DOWN
NOCHAN(	NOCHAN(	DOWN	UP	DOWN	NOCHAN(	DOWN	NOCHAN(	DOWN
NOCHAN(	NOCHAN(	NOCHAN(	UP	NOCHAN(	NOCHAN(	DOWN	NOCHAN(	NOCHAN(
NOCHAN(	NOCHAN(	UP	NOCHAN(	UP	NOCHAN(	NOCHAN(	NOCHAN(	NOCHAN(
NOCHAN(	DOWN	UP	UP	UP	UP	UP	UP	NOCHAN(
NOCHAN(	NOCHAN(	DOWN	DOWN	NOCHAN(	NOCHAN(	NOCHAN(	DOWN	DOWN
NOCHAN(	UP	NOCHAN(	NOCHAN(	NOCHAN(	NOCHAN(	NOCHAN(	NOCHAN(	UP
NOCHAN(	NOCHAN(	DOWN	UP	NOCHAN(	NOCHAN(	NOCHAN(	NOCHAN(	NOCHAN(
NOCHAN(	NOCHAN(	DOWN	NOCHAN(	NOCHAN(	NOCHAN(	NOCHAN(	NOCHAN(	DOWN
NOCHAN(	DOWN	NOCHAN(	UP	NOCHAN(	NOCHAN(	NOCHAN(	NOCHAN(	NOCHAN(
NOCHAN(	NOCHAN(	DOWN	NOCHAN(	NOCHAN(	NOCHAN(	NOCHAN(	UP	NOCHAN(
NOCHAN(	UP	NOCHAN(	NOCHAN(	NOCHAN(	NOCHAN(	NOCHAN(	NOCHAN(	UP
NOCHAN(	NOCHAN(	NOCHAN(	UP	UP	UP	NOCHAN(	NOCHAN(	NOCHAN(
NOCHAN(	UP	NOCHAN(	DOWN	UP	UP	NOCHAN(	UP	NOCHAN(
NOCHAN(	NOCHAN(	DOWN	UP	NOCHAN(	DOWN	DOWN	DOWN	DOWN
DOWN	NOCHAN(	DOWN	UP	DOWN	NOCHAN(	DOWN	DOWN	NOCHAN(
NOCHAN(	NOCHAN(	DOWN	UP	NOCHAN(	NOCHAN(	DOWN	DOWN	DOWN
NOCHAN(	NOCHAN(	DOWN	DOWN	NOCHAN(	NOCHAN(	NOCHAN(	DOWN	NOCHAN(
UP	UP	UP	DOWN	UP	NOCHAN(	UP	UP	UP
NOCHAN(	NOCHAN(	DOWN	DOWN	NOCHAN(	NOCHAN(	DOWN	NOCHAN(	NOCHAN(
NOCHAN(	UP	NOCHAN(	NOCHAN(	NOCHAN(	NOCHAN(	DOWN	NOCHAN(	NOCHAN(
NOCHAN(	DOWN	NOCHAN(	NOCHAN(	NOCHAN(	NOCHAN(	NOCHAN(	NOCHAN(	DOWN
DOWN	NOCHAN(	DOWN	DOWN	NOCHAN(	NOCHAN(	NOCHAN(	DOWN	DOWN
NOCHAN(	NOCHAN(	DOWN	UP	NOCHAN(	NOCHAN(	NOCHAN(	DOWN	NOCHAN(
NOCHAN(	NOCHAN(	NOCHAN(	NOCHAN(	NOCHAN(	UP	NOCHAN(	NOCHAN(	NOCHAN(
NOCHAN(	NOCHAN(	NOCHAN(	UP	DOWN	NOCHAN(	NOCHAN(	NOCHAN(	NOCHAN(
NOCHAN(	NOCHAN(	UP	DOWN	DOWN	NOCHAN(	NOCHAN(	NOCHAN(	NOCHAN(
NOCHAN(	UP	DOWN	NOCHAN(	NOCHAN(	NOCHAN(	DOWN	NOCHAN(	NOCHAN(
UP	UP	NOCHAN(	UP	NOCHAN(	UP	NOCHAN(	UP	UP
NOCHAN(	NOCHAN(	UP	NOCHAN(	NOCHAN(	NOCHAN(	NOCHAN(	DOWN	NOCHAN(
UP	NOCHAN(	NOCHAN(	NOCHAN(	NOCHAN(	NOCHAN(	NOCHAN(	NOCHAN(	NOCHAN(
NOCHAN(	NOCHAN(	DOWN	UP	NOCHAN(	NOCHAN(	NOCHAN(	DOWN	NOCHAN(
DOWN	DOWN	DOWN	NOCHAN(	NOCHAN(	DOWN	DOWN	DOWN	DOWN
UP	NOCHAN(	NOCHAN(	DOWN	NOCHAN(	NOCHAN(	UP	NOCHAN(	UP
UP	UP	DOWN	NOCHAN(	UP	NOCHAN(	NOCHAN(	UP	UP
DOWN	NOCHAN(	UP	NOCHAN(	UP	UP	NOCHAN(	NOCHAN(	NOCHAN(

TCGA-B2-	TCGA-BP-	TCGA-BP-	TCGA-CZ-	TCGA-AK-	TCGA-B0-	TCGA-BP-	TCGA-BP-	TCGA-BP-
NOCHAN(	DOWN	DOWN	DOWN	NOCHAN(	NOCHAN(	DOWN	DOWN	NOCHAN(
NOCHAN(	NOCHAN(	DOWN	DOWN	NOCHAN(	DOWN	DOWN	DOWN	NOCHAN(
NOCHAN(	DOWN	DOWN	DOWN	NOCHAN(	DOWN	DOWN	DOWN	DOWN
NOCHAN(	DOWN	NOCHAN(	NOCHAN(	NOCHAN(	UP	NOCHAN(	NOCHAN(	NOCHAN(
UP	DOWN	NOCHAN(	NOCHAN(	NOCHAN(	NOCHAN(	DOWN	UP	NOCHAN(
DOWN	NOCHAN(	DOWN	DOWN	NOCHAN(	DOWN	DOWN	NOCHAN(	NOCHAN(
NOCHAN(	DOWN	DOWN	NOCHAN(	DOWN	DOWN	NOCHAN(	NOCHAN(	DOWN
NOCHAN(	DOWN	UP	NOCHAN(	NOCHAN(	NOCHAN(	NOCHAN(	DOWN	UP
UP	DOWN	NOCHAN(	NOCHAN(	UP	UP	UP	NOCHAN(	UP
NOCHAN(	DOWN	DOWN	DOWN	NOCHAN(	DOWN	DOWN	DOWN	DOWN
UP	NOCHAN(	NOCHAN(	NOCHAN(	NOCHAN(	NOCHAN(	NOCHAN(	UP	NOCHAN(
NOCHAN(	DOWN	NOCHAN(	NOCHAN(	UP	NOCHAN(	UP	DOWN	NOCHAN(
NOCHAN(	NOCHAN(	DOWN	DOWN	NOCHAN(	NOCHAN(	NOCHAN(	NOCHAN(	NOCHAN(
NOCHAN(	DOWN	NOCHAN(	UP	NOCHAN(	NOCHAN(	UP	NOCHAN(	NOCHAN(
NOCHAN(	DOWN	NOCHAN(	UP	NOCHAN(	NOCHAN(	DOWN	DOWN	NOCHAN(
NOCHAN(	NOCHAN(	UP	NOCHAN(	NOCHAN(	NOCHAN(	UP	NOCHAN(	NOCHAN(
UP	DOWN	NOCHAN(	DOWN	NOCHAN(	NOCHAN(	DOWN	UP	NOCHAN(
NOCHAN(	DOWN	NOCHAN(	UP	NOCHAN(	NOCHAN(	NOCHAN(	NOCHAN(	NOCHAN(
DOWN	DOWN	NOCHAN(	DOWN	NOCHAN(	NOCHAN(	DOWN	DOWN	DOWN
DOWN	DOWN	NOCHAN(	DOWN	DOWN	UP	DOWN	DOWN	DOWN
DOWN	DOWN	DOWN	DOWN	DOWN	NOCHAN(	DOWN	DOWN	NOCHAN(
NOCHAN(	DOWN	NOCHAN(	DOWN	NOCHAN(	NOCHAN(	NOCHAN(	NOCHAN(	DOWN
NOCHAN(	UP	NOCHAN(	UP	UP	UP	UP	UP	UP
NOCHAN(	DOWN	NOCHAN(	UP	NOCHAN(	DOWN	NOCHAN(	NOCHAN(	DOWN
NOCHAN(	NOCHAN(	NOCHAN(	NOCHAN(	NOCHAN(	NOCHAN(	NOCHAN(	NOCHAN(	NOCHAN(
NOCHAN(	DOWN	DOWN	DOWN	NOCHAN(	NOCHAN(	NOCHAN(	NOCHAN(	NOCHAN(
NOCHAN(	DOWN	DOWN	DOWN	DOWN	DOWN	DOWN	DOWN	NOCHAN(
NOCHAN(	DOWN	NOCHAN(	DOWN	NOCHAN(	NOCHAN(	NOCHAN(	NOCHAN(	DOWN
NOCHAN(	DOWN	NOCHAN(	NOCHAN(	DOWN	NOCHAN(	NOCHAN(	NOCHAN(	NOCHAN(
DOWN	UP	NOCHAN(	UP	DOWN	NOCHAN(	NOCHAN(	NOCHAN(	NOCHAN(
NOCHAN(	DOWN	NOCHAN(	UP	UP	NOCHAN(	UP	NOCHAN(	DOWN
NOCHAN(	NOCHAN(	NOCHAN(	DOWN	NOCHAN(	DOWN	DOWN	NOCHAN(	NOCHAN(
UP	NOCHAN(	UP	NOCHAN(	NOCHAN(	UP	NOCHAN(	UP	UP
NOCHAN(	DOWN	NOCHAN(	UP	UP	NOCHAN(	NOCHAN(	NOCHAN(	DOWN
NOCHAN(	NOCHAN(	NOCHAN(	NOCHAN(	UP	UP	NOCHAN(	NOCHAN(	NOCHAN(
NOCHAN(	NOCHAN(	NOCHAN(	DOWN	NOCHAN(	NOCHAN(	DOWN	DOWN	NOCHAN(
NOCHAN(	DOWN	NOCHAN(	DOWN	NOCHAN(	DOWN	DOWN	DOWN	DOWN
NOCHAN(	NOCHAN(	NOCHAN(	UP	UP	NOCHAN(	UP	NOCHAN(	NOCHAN(
NOCHAN(	DOWN	UP	NOCHAN(	DOWN	NOCHAN(	DOWN	NOCHAN(	UP
UP	DOWN	NOCHAN(	NOCHAN(	NOCHAN(	NOCHAN(	DOWN	UP	NOCHAN(

TCGA-B0-	TCGA-B0-	TCGA-3Z-	TCGA-DV	TCGA-BP-	TCGA-BP-	TCGA-BP-	TCGA-AK-	TCGA-B8-
NOCHANG	NOCHANG	NOCHANG	DOWN	DOWN	DOWN	NOCHANG	NOCHANG	DOWN
DOWN	DOWN	DOWN	NOCHANG	NOCHANG	NOCHANG	DOWN	DOWN	NOCHANG
DOWN	DOWN	DOWN	DOWN	DOWN	DOWN	DOWN	DOWN	NOCHANG
UP	UP	UP	NOCHANG	NOCHANG	NOCHANG	NOCHANG	NOCHANG	NOCHANG
NOCHANG	UP	NOCHANG	NOCHANG	NOCHANG	NOCHANG	NOCHANG	NOCHANG	NOCHANG
NOCHANG	DOWN	DOWN	NOCHANG	NOCHANG	NOCHANG	DOWN	NOCHANG	NOCHANG
NOCHANG	NOCHANG	DOWN	NOCHANG	NOCHANG	NOCHANG	NOCHANG	NOCHANG	DOWN
NOCHANG	DOWN	NOCHANG	NOCHANG	NOCHANG	NOCHANG	NOCHANG	NOCHANG	NOCHANG
UP	UP	UP	NOCHANG	NOCHANG	NOCHANG	NOCHANG	NOCHANG	NOCHANG
NOCHANG	DOWN	DOWN	DOWN	NOCHANG	NOCHANG	NOCHANG	NOCHANG	DOWN
NOCHANG	UP	UP	NOCHANG	NOCHANG	UP	NOCHANG	NOCHANG	NOCHANG
NOCHANG	NOCHANG	NOCHANG	NOCHANG	NOCHANG	NOCHANG	NOCHANG	NOCHANG	NOCHANG
NOCHANG	NOCHANG	NOCHANG	DOWN	NOCHANG	NOCHANG	NOCHANG	NOCHANG	DOWN
NOCHANG	NOCHANG	UP	NOCHANG	NOCHANG	NOCHANG	NOCHANG	NOCHANG	NOCHANG
NOCHANG	DOWN	NOCHANG	NOCHANG	NOCHANG	NOCHANG	UP	DOWN	NOCHANG
NOCHANG	NOCHANG	UP	NOCHANG	NOCHANG	NOCHANG	DOWN	UP	NOCHANG
UP	UP	NOCHANG	NOCHANG	NOCHANG	NOCHANG	NOCHANG	NOCHANG	NOCHANG
NOCHANG	NOCHANG	NOCHANG	NOCHANG	NOCHANG	UP	UP	NOCHANG	NOCHANG
NOCHANG	DOWN	DOWN	DOWN	DOWN	DOWN	DOWN	NOCHANG	DOWN
NOCHANG	NOCHANG	DOWN	DOWN	NOCHANG	DOWN	DOWN	DOWN	DOWN
NOCHANG	DOWN	DOWN	DOWN	NOCHANG	NOCHANG	NOCHANG	NOCHANG	NOCHANG
NOCHANG	NOCHANG	NOCHANG	DOWN	NOCHANG	DOWN	NOCHANG	NOCHANG	DOWN
UP	UP	UP	UP	UP	UP	UP	UP	UP
NOCHANG	NOCHANG	NOCHANG	NOCHANG	NOCHANG	NOCHANG	NOCHANG	NOCHANG	NOCHANG
NOCHANG	NOCHANG	NOCHANG	NOCHANG	NOCHANG	NOCHANG	NOCHANG	NOCHANG	NOCHANG
NOCHANG	NOCHANG	NOCHANG	DOWN	DOWN	NOCHANG	NOCHANG	DOWN	NOCHANG
DOWN	DOWN	DOWN	DOWN	DOWN	NOCHANG	NOCHANG	DOWN	NOCHANG
NOCHANG	DOWN	NOCHANG	NOCHANG	NOCHANG	NOCHANG	NOCHANG	NOCHANG	NOCHANG
NOCHANG	NOCHANG	NOCHANG	NOCHANG	NOCHANG	UP	UP	NOCHANG	UP
NOCHANG	NOCHANG	NOCHANG	NOCHANG	DOWN	DOWN	NOCHANG	NOCHANG	NOCHANG
NOCHANG	NOCHANG	UP	NOCHANG	NOCHANG	DOWN	NOCHANG	NOCHANG	NOCHANG
NOCHANG	DOWN	NOCHANG	NOCHANG	NOCHANG	NOCHANG	NOCHANG	NOCHANG	NOCHANG
UP	UP	NOCHANG	NOCHANG	NOCHANG	UP	UP	UP	NOCHANG
NOCHANG	NOCHANG	UP	NOCHANG	NOCHANG	NOCHANG	NOCHANG	NOCHANG	NOCHANG
NOCHANG	DOWN	UP	NOCHANG	NOCHANG	NOCHANG	DOWN	NOCHANG	UP
DOWN	NOCHANG	NOCHANG	NOCHANG	NOCHANG	NOCHANG	NOCHANG	NOCHANG	NOCHANG
NOCHANG	DOWN	NOCHANG	DOWN	DOWN	DOWN	DOWN	DOWN	DOWN
NOCHANG	NOCHANG	UP	UP	UP	NOCHANG	NOCHANG	UP	NOCHANG
NOCHANG	DOWN	UP	NOCHANG	UP	UP	UP	NOCHANG	UP
NOCHANG	UP	NOCHANG	NOCHANG	NOCHANG	UP	UP	NOCHANG	NOCHANG

TCGA-BP-	TCGA-DV	TCGA-B0-	TCGA-CZ-	TCGA-B0-	TCGA-BP-	TCGA-B0-	TCGA-BP-	TCGA-CZ-
DOWN	NOCHAN(	DOWN	DOWN	DOWN	NOCHAN(	DOWN	NOCHAN(	DOWN
DOWN	DOWN	NOCHAN(	DOWN	NOCHAN(	DOWN	NOCHAN(	DOWN	DOWN
DOWN	DOWN	DOWN	DOWN	DOWN	NOCHAN(	DOWN	NOCHAN(	DOWN
UP	NOCHAN(	NOCHAN(	NOCHAN(	NOCHAN(	NOCHAN(	NOCHAN(	NOCHAN(	DOWN
NOCHAN(	NOCHAN(	NOCHAN(	NOCHAN(	NOCHAN(	NOCHAN(	DOWN	NOCHAN(	NOCHAN(
DOWN	DOWN	NOCHAN(	NOCHAN(	DOWN	DOWN	DOWN	NOCHAN(	DOWN
DOWN	NOCHAN(	NOCHAN(	DOWN	DOWN	NOCHAN(	NOCHAN(	NOCHAN(	NOCHAN(
NOCHAN(	NOCHAN(	NOCHAN(	NOCHAN(	NOCHAN(	NOCHAN(	DOWN	NOCHAN(	DOWN
UP	UP	NOCHAN(	UP	UP	NOCHAN(	UP	NOCHAN(	NOCHAN(
DOWN	DOWN	NOCHAN(	NOCHAN(	DOWN	NOCHAN(	DOWN	NOCHAN(	NOCHAN(
NOCHAN(	NOCHAN(	UP	NOCHAN(	NOCHAN(	UP	NOCHAN(	NOCHAN(	NOCHAN(
NOCHAN(	UP	NOCHAN(	NOCHAN(	NOCHAN(	NOCHAN(	UP	NOCHAN(	NOCHAN(
NOCHAN(	NOCHAN(	NOCHAN(	NOCHAN(	DOWN	NOCHAN(	NOCHAN(	NOCHAN(	NOCHAN(
NOCHAN(	UP	NOCHAN(	UP	NOCHAN(	NOCHAN(	UP	NOCHAN(	NOCHAN(
DOWN	NOCHAN(	NOCHAN(	NOCHAN(	NOCHAN(	NOCHAN(	NOCHAN(	NOCHAN(	DOWN
UP	NOCHAN(	NOCHAN(	NOCHAN(	NOCHAN(	NOCHAN(	UP	NOCHAN(	UP
NOCHAN(	NOCHAN(	NOCHAN(	NOCHAN(	NOCHAN(	NOCHAN(	DOWN	NOCHAN(	NOCHAN(
NOCHAN(	NOCHAN(	NOCHAN(	NOCHAN(	UP	UP	NOCHAN(	UP	NOCHAN(
DOWN	NOCHAN(	NOCHAN(	DOWN	DOWN	NOCHAN(	DOWN	NOCHAN(	NOCHAN(
DOWN	DOWN	NOCHAN(	DOWN	NOCHAN(	DOWN	DOWN	DOWN	DOWN
DOWN	DOWN	NOCHAN(	NOCHAN(	DOWN	NOCHAN(	DOWN	NOCHAN(	DOWN
NOCHAN(	NOCHAN(	NOCHAN(	NOCHAN(	DOWN	NOCHAN(	NOCHAN(	NOCHAN(	NOCHAN(
UP	UP	UP	UP	UP	UP	UP	UP	UP
NOCHAN(	DOWN	NOCHAN(	NOCHAN(	NOCHAN(	NOCHAN(	NOCHAN(	NOCHAN(	NOCHAN(
NOCHAN(	NOCHAN(	NOCHAN(	DOWN	NOCHAN(	NOCHAN(	NOCHAN(	NOCHAN(	NOCHAN(
NOCHAN(	NOCHAN(	NOCHAN(	NOCHAN(	NOCHAN(	NOCHAN(	DOWN	NOCHAN(	DOWN
DOWN	DOWN	DOWN	DOWN	DOWN	DOWN	DOWN	DOWN	DOWN
NOCHAN(	NOCHAN(	NOCHAN(	NOCHAN(	NOCHAN(	NOCHAN(	NOCHAN(	NOCHAN(	NOCHAN(
NOCHAN(	DOWN	NOCHAN(	NOCHAN(	NOCHAN(	NOCHAN(	NOCHAN(	NOCHAN(	NOCHAN(
NOCHAN(	NOCHAN(	NOCHAN(	NOCHAN(	NOCHAN(	DOWN	UP	NOCHAN(	NOCHAN(
UP	UP	NOCHAN(	NOCHAN(	NOCHAN(	DOWN	UP	NOCHAN(	NOCHAN(
DOWN	DOWN	NOCHAN(	NOCHAN(	NOCHAN(	NOCHAN(	DOWN	NOCHAN(	NOCHAN(
NOCHAN(	NOCHAN(	UP	NOCHAN(	UP	UP	NOCHAN(	UP	NOCHAN(
NOCHAN(	UP	NOCHAN(	NOCHAN(	NOCHAN(	NOCHAN(	UP	NOCHAN(	NOCHAN(
NOCHAN(	UP	UP	UP	NOCHAN(	NOCHAN(	UP	NOCHAN(	NOCHAN(
NOCHAN(	DOWN	NOCHAN(	NOCHAN(	DOWN	NOCHAN(	DOWN	NOCHAN(	NOCHAN(
DOWN	DOWN	DOWN	DOWN	DOWN	DOWN	DOWN	DOWN	DOWN
NOCHAN(	UP	UP	NOCHAN(	NOCHAN(	UP	UP	UP	UP
NOCHAN(	NOCHAN(	NOCHAN(	NOCHAN(	UP	UP	NOCHAN(	UP	UP
UP	NOCHAN(	NOCHAN(	NOCHAN(	UP	UP	NOCHAN(	UP	NOCHAN(

TCGA-BP-	TCGA-CZ-	TCGA-CJ-	TCGA-CJ-	TCGA-B0-	TCGA-BP-	TCGA-CJ-	TCGA-B0-	TCGA-CJ-
DOWN	DOWN	DOWN	NOCHAN(	NOCHAN(	NOCHAN(	DOWN	DOWN	DOWN
DOWN	DOWN	NOCHAN(	NOCHAN(	DOWN	NOCHAN(	DOWN	DOWN	DOWN
DOWN	DOWN	DOWN	DOWN	DOWN	DOWN	DOWN	DOWN	DOWN
NOCHAN(	NOCHAN(	NOCHAN(	NOCHAN(	NOCHAN(	UP	UP	NOCHAN(	UP
NOCHAN(	NOCHAN(	NOCHAN(	NOCHAN(	NOCHAN(	NOCHAN(	NOCHAN(	NOCHAN(	NOCHAN(
DOWN	DOWN	DOWN	NOCHAN(	DOWN	DOWN	DOWN	DOWN	DOWN
NOCHAN(	DOWN	NOCHAN(	NOCHAN(	DOWN	NOCHAN(	DOWN	DOWN	NOCHAN(
NOCHAN(	NOCHAN(	NOCHAN(	NOCHAN(	NOCHAN(	NOCHAN(	NOCHAN(	UP	NOCHAN(
NOCHAN(	UP	NOCHAN(	NOCHAN(	UP	UP	UP	NOCHAN(	UP
NOCHAN(	DOWN	DOWN	NOCHAN(	NOCHAN(	DOWN	DOWN	NOCHAN(	DOWN
NOCHAN(	NOCHAN(	NOCHAN(	NOCHAN(	UP	NOCHAN(	UP	NOCHAN(	NOCHAN(
NOCHAN(	NOCHAN(	NOCHAN(	NOCHAN(	NOCHAN(	NOCHAN(	NOCHAN(	DOWN	NOCHAN(
NOCHAN(	NOCHAN(	NOCHAN(	DOWN	NOCHAN(	NOCHAN(	NOCHAN(	NOCHAN(	NOCHAN(
NOCHAN(	NOCHAN(	NOCHAN(	NOCHAN(	NOCHAN(	UP	NOCHAN(	NOCHAN(	UP
NOCHAN(	NOCHAN(	NOCHAN(	NOCHAN(	NOCHAN(	NOCHAN(	NOCHAN(	NOCHAN(	NOCHAN(
NOCHAN(	UP	NOCHAN(	NOCHAN(	NOCHAN(	NOCHAN(	NOCHAN(	NOCHAN(	NOCHAN(
NOCHAN(	NOCHAN(	NOCHAN(	NOCHAN(	NOCHAN(	NOCHAN(	NOCHAN(	NOCHAN(	NOCHAN(
NOCHAN(	NOCHAN(	UP	UP	UP	UP	NOCHAN(	NOCHAN(	NOCHAN(
DOWN	NOCHAN(	DOWN	DOWN	NOCHAN(	DOWN	DOWN	DOWN	DOWN
DOWN	DOWN	NOCHAN(	DOWN	DOWN	DOWN	DOWN	DOWN	DOWN
DOWN	DOWN	DOWN	DOWN	DOWN	DOWN	DOWN	NOCHAN(	DOWN
NOCHAN(	NOCHAN(	NOCHAN(	DOWN	NOCHAN(	DOWN	DOWN	DOWN	NOCHAN(
UP	UP	UP	UP	UP	UP	UP	UP	UP
DOWN	DOWN	NOCHAN(	NOCHAN(	DOWN	NOCHAN(	DOWN	NOCHAN(	DOWN
NOCHAN(	NOCHAN(	NOCHAN(	DOWN	NOCHAN(	NOCHAN(	NOCHAN(	NOCHAN(	DOWN
NOCHAN(	NOCHAN(	NOCHAN(	NOCHAN(	NOCHAN(	NOCHAN(	NOCHAN(	NOCHAN(	DOWN
NOCHAN(	DOWN	NOCHAN(	NOCHAN(	DOWN	NOCHAN(	DOWN	DOWN	DOWN
NOCHAN(	NOCHAN(	NOCHAN(	NOCHAN(	NOCHAN(	NOCHAN(	DOWN	NOCHAN(	DOWN
NOCHAN(	NOCHAN(	NOCHAN(	UP	NOCHAN(	NOCHAN(	NOCHAN(	NOCHAN(	NOCHAN(
NOCHAN(	NOCHAN(	NOCHAN(	NOCHAN(	NOCHAN(	UP	NOCHAN(	NOCHAN(	NOCHAN(
DOWN	UP	NOCHAN(	NOCHAN(	NOCHAN(	NOCHAN(	NOCHAN(	NOCHAN(	UP
NOCHAN(	DOWN	NOCHAN(	NOCHAN(	NOCHAN(	DOWN	NOCHAN(	NOCHAN(	DOWN
NOCHAN(	NOCHAN(	UP	NOCHAN(	NOCHAN(	NOCHAN(	NOCHAN(	NOCHAN(	NOCHAN(
NOCHAN(	NOCHAN(	NOCHAN(	NOCHAN(	NOCHAN(	NOCHAN(	NOCHAN(	NOCHAN(	UP
UP	UP	NOCHAN(	NOCHAN(	NOCHAN(	NOCHAN(	UP	NOCHAN(	DOWN
NOCHAN(	NOCHAN(	DOWN	NOCHAN(	NOCHAN(	DOWN	DOWN	NOCHAN(	NOCHAN(
DOWN	DOWN	DOWN	NOCHAN(	DOWN	DOWN	DOWN	DOWN	DOWN
NOCHAN(	NOCHAN(	NOCHAN(	NOCHAN(	NOCHAN(	UP	UP	NOCHAN(	UP
UP	NOCHAN(	NOCHAN(	UP	UP	NOCHAN(	NOCHAN(	NOCHAN(	DOWN
NOCHAN(	DOWN	UP	UP	NOCHAN(	UP	UP	NOCHAN(	NOCHAN(

TCGA-CZ-	TCGA-AK-	TCGA-AK-	TCGA-B0-	TCGA-B0-	TCGA-CJ-	TCGA-B0-	TCGA-CZ-	TCGA-CJ-
DOWN	NOCHANG	DOWN	NOCHANG	DOWN	DOWN	NOCHANG	DOWN	NOCHANG
NOCHANG	NOCHANG	DOWN	NOCHANG	DOWN	DOWN	DOWN	DOWN	DOWN
DOWN	NOCHANG	DOWN	DOWN	DOWN	DOWN	DOWN	DOWN	DOWN
DOWN	NOCHANG	UP	NOCHANG	NOCHANG	NOCHANG	UP	NOCHANG	NOCHANG
NOCHANG	DOWN	NOCHANG	NOCHANG	DOWN	DOWN	NOCHANG	NOCHANG	NOCHANG
DOWN	UP	DOWN	DOWN	DOWN	DOWN	DOWN	DOWN	DOWN
NOCHANG	UP	DOWN	DOWN	DOWN	NOCHANG	DOWN	NOCHANG	DOWN
NOCHANG	NOCHANG	NOCHANG	UP	DOWN	UP	NOCHANG	NOCHANG	NOCHANG
DOWN	NOCHANG	UP	NOCHANG	NOCHANG	UP	UP	NOCHANG	UP
NOCHANG	NOCHANG	DOWN	DOWN	DOWN	DOWN	DOWN	NOCHANG	NOCHANG
NOCHANG	NOCHANG	UP	NOCHANG	NOCHANG	NOCHANG	UP	NOCHANG	NOCHANG
DOWN	UP	NOCHANG	NOCHANG	NOCHANG	NOCHANG	NOCHANG	NOCHANG	NOCHANG
NOCHANG	DOWN	NOCHANG	NOCHANG	NOCHANG	NOCHANG	NOCHANG	NOCHANG	NOCHANG
DOWN	UP	NOCHANG	UP	NOCHANG	UP	NOCHANG	UP	NOCHANG
NOCHANG	NOCHANG	NOCHANG	NOCHANG	DOWN	DOWN	NOCHANG	NOCHANG	NOCHANG
NOCHANG	UP	NOCHANG	NOCHANG	NOCHANG	NOCHANG	NOCHANG	UP	NOCHANG
NOCHANG	UP	UP	NOCHANG	DOWN	DOWN	NOCHANG	NOCHANG	NOCHANG
UP	DOWN	NOCHANG	NOCHANG	NOCHANG	NOCHANG	UP	UP	NOCHANG
NOCHANG	UP	DOWN	DOWN	DOWN	DOWN	DOWN	DOWN	NOCHANG
DOWN	UP	DOWN	DOWN	DOWN	DOWN	NOCHANG	NOCHANG	DOWN
NOCHANG	UP	DOWN	DOWN	DOWN	DOWN	DOWN	DOWN	DOWN
NOCHANG	DOWN	NOCHANG	NOCHANG	NOCHANG	DOWN	DOWN	NOCHANG	NOCHANG
UP	DOWN	UP	UP	UP	UP	UP	UP	UP
NOCHANG	NOCHANG	DOWN	DOWN	DOWN	DOWN	NOCHANG	NOCHANG	NOCHANG
DOWN	NOCHANG	NOCHANG	NOCHANG	DOWN	NOCHANG	NOCHANG	NOCHANG	NOCHANG
DOWN	NOCHANG	NOCHANG	NOCHANG	DOWN	DOWN	NOCHANG	NOCHANG	NOCHANG
NOCHANG	DOWN	DOWN	NOCHANG	DOWN	DOWN	DOWN	DOWN	NOCHANG
NOCHANG	UP	DOWN	NOCHANG	DOWN	NOCHANG	NOCHANG	NOCHANG	NOCHANG
NOCHANG	UP	NOCHANG	NOCHANG	NOCHANG	NOCHANG	UP	NOCHANG	NOCHANG
NOCHANG	UP	DOWN	NOCHANG	DOWN	NOCHANG	NOCHANG	NOCHANG	NOCHANG
NOCHANG	DOWN	UP	NOCHANG	UP	UP	NOCHANG	NOCHANG	NOCHANG
NOCHANG	NOCHANG	DOWN	DOWN	DOWN	DOWN	NOCHANG	NOCHANG	NOCHANG
NOCHANG	DOWN	NOCHANG	NOCHANG	NOCHANG	NOCHANG	UP	UP	NOCHANG
NOCHANG	NOCHANG	NOCHANG	NOCHANG	UP	UP	NOCHANG	NOCHANG	NOCHANG
UP	NOCHANG	DOWN	NOCHANG	NOCHANG	NOCHANG	NOCHANG	UP	UP
DOWN	UP	DOWN	NOCHANG	DOWN	NOCHANG	NOCHANG	NOCHANG	NOCHANG
DOWN	DOWN	NOCHANG	DOWN	DOWN	DOWN	DOWN	DOWN	DOWN
UP	UP	NOCHANG	NOCHANG	NOCHANG	NOCHANG	NOCHANG	UP	NOCHANG
NOCHANG	UP	DOWN	NOCHANG	DOWN	NOCHANG	NOCHANG	UP	UP
NOCHANG	NOCHANG	NOCHANG	NOCHANG	DOWN	NOCHANG	UP	NOCHANG	NOCHANG

TCGA-BP-	TCGA-B0-	TCGA-B0-	TCGA-B8-	TCGA-CJ-	TCGA-BP-	TCGA-BP-	TCGA-A3-	TCGA-A3-
DOWN	DOWN	NOCHAN(	DOWN	DOWN	NOCHAN(	DOWN	NOCHAN(	DOWN
NOCHAN(	NOCHAN(	NOCHAN(	NOCHAN(	DOWN	DOWN	NOCHAN(	DOWN	NOCHAN(
DOWN	DOWN	DOWN	DOWN	DOWN	DOWN	DOWN	DOWN	DOWN
NOCHAN(	NOCHAN(	UP	NOCHAN(	UP	NOCHAN(	DOWN	NOCHAN(	NOCHAN(
NOCHAN(	NOCHAN(	NOCHAN(	NOCHAN(	NOCHAN(	UP	NOCHAN(	NOCHAN(	NOCHAN(
NOCHAN(	NOCHAN(	NOCHAN(	DOWN	DOWN	DOWN	DOWN	NOCHAN(	DOWN
DOWN	NOCHAN(	DOWN	NOCHAN(	DOWN	NOCHAN(	DOWN	UP	DOWN
UP	NOCHAN(	NOCHAN(	UP	NOCHAN(	NOCHAN(	NOCHAN(	NOCHAN(	NOCHAN(
NOCHAN(	UP	UP	NOCHAN(	UP	UP	NOCHAN(	NOCHAN(	UP
DOWN	NOCHAN(	NOCHAN(	NOCHAN(	DOWN	DOWN	DOWN	DOWN	DOWN
NOCHAN(	NOCHAN(	NOCHAN(	NOCHAN(	NOCHAN(	UP	NOCHAN(	UP	NOCHAN(
NOCHAN(	NOCHAN(	NOCHAN(	NOCHAN(	NOCHAN(	NOCHAN(	NOCHAN(	NOCHAN(	NOCHAN(
DOWN	NOCHAN(	NOCHAN(	NOCHAN(	NOCHAN(	NOCHAN(	DOWN	NOCHAN(	NOCHAN(
NOCHAN(	NOCHAN(	UP	NOCHAN(	NOCHAN(	UP	DOWN	NOCHAN(	NOCHAN(
NOCHAN(	NOCHAN(	NOCHAN(	NOCHAN(	NOCHAN(	NOCHAN(	NOCHAN(	NOCHAN(	NOCHAN(
NOCHAN(	NOCHAN(	NOCHAN(	NOCHAN(	NOCHAN(	NOCHAN(	NOCHAN(	NOCHAN(	NOCHAN(
NOCHAN(	NOCHAN(	NOCHAN(	NOCHAN(	NOCHAN(	NOCHAN(	DOWN	NOCHAN(	NOCHAN(
NOCHAN(	NOCHAN(	UP	NOCHAN(	NOCHAN(	UP	NOCHAN(	NOCHAN(	NOCHAN(
DOWN	NOCHAN(	DOWN	DOWN	NOCHAN(	DOWN	DOWN	NOCHAN(	DOWN
DOWN	NOCHAN(	DOWN	DOWN	DOWN	DOWN	DOWN	NOCHAN(	DOWN
DOWN	NOCHAN(	NOCHAN(	NOCHAN(	DOWN	DOWN	DOWN	NOCHAN(	NOCHAN(
DOWN	NOCHAN(	NOCHAN(	NOCHAN(	NOCHAN(	NOCHAN(	NOCHAN(	DOWN	DOWN
UP	UP	NOCHAN(	UP	NOCHAN(	UP	UP	NOCHAN(	NOCHAN(
NOCHAN(	DOWN	NOCHAN(	NOCHAN(	DOWN	NOCHAN(	DOWN	NOCHAN(	DOWN
NOCHAN(	NOCHAN(	NOCHAN(	NOCHAN(	NOCHAN(	DOWN	NOCHAN(	NOCHAN(	NOCHAN(
DOWN	NOCHAN(	NOCHAN(	NOCHAN(	NOCHAN(	NOCHAN(	NOCHAN(	NOCHAN(	NOCHAN(
DOWN	DOWN	NOCHAN(	DOWN	DOWN	DOWN	DOWN	DOWN	DOWN
NOCHAN(	NOCHAN(	NOCHAN(	NOCHAN(	NOCHAN(	NOCHAN(	NOCHAN(	UP	NOCHAN(
UP	NOCHAN(	NOCHAN(	NOCHAN(	NOCHAN(	NOCHAN(	NOCHAN(	UP	NOCHAN(
NOCHAN(	NOCHAN(	NOCHAN(	NOCHAN(	NOCHAN(	NOCHAN(	NOCHAN(	UP	NOCHAN(
UP	NOCHAN(	NOCHAN(	DOWN	UP	UP	NOCHAN(	NOCHAN(	NOCHAN(
NOCHAN(	NOCHAN(	NOCHAN(	NOCHAN(	NOCHAN(	NOCHAN(	DOWN	DOWN	NOCHAN(
NOCHAN(	UP	UP	NOCHAN(	NOCHAN(	NOCHAN(	UP	DOWN	NOCHAN(
NOCHAN(	NOCHAN(	NOCHAN(	NOCHAN(	NOCHAN(	NOCHAN(	NOCHAN(	NOCHAN(	DOWN
NOCHAN(	NOCHAN(	NOCHAN(	NOCHAN(	UP	NOCHAN(	UP	NOCHAN(	UP
DOWN	NOCHAN(	NOCHAN(	NOCHAN(	NOCHAN(	NOCHAN(	NOCHAN(	NOCHAN(	NOCHAN(
DOWN	DOWN	DOWN	DOWN	NOCHAN(	DOWN	DOWN	NOCHAN(	DOWN
NOCHAN(	NOCHAN(	NOCHAN(	NOCHAN(	NOCHAN(	NOCHAN(	UP	NOCHAN(	NOCHAN(
DOWN	UP	UP	UP	NOCHAN(	NOCHAN(	NOCHAN(	NOCHAN(	UP
NOCHAN(	NOCHAN(	UP	NOCHAN(	NOCHAN(	UP	DOWN	NOCHAN(	NOCHAN(

TCGA-B0-	TCGA-B4-	TCGA-B0-	TCGA-BP-	TCGA-BP-	TCGA-BP-	TCGA-B0-	TCGA-A3-	TCGA-CJ-
DOWN	DOWN	DOWN	NOCHAN(	DOWN	DOWN	NOCHAN(	NOCHAN(	UP
DOWN	DOWN	NOCHAN(	DOWN	NOCHAN(	NOCHAN(	DOWN	DOWN	DOWN
DOWN	DOWN	NOCHAN(	DOWN	DOWN	DOWN	NOCHAN(	DOWN	DOWN
UP	NOCHAN(	NOCHAN(	UP	UP	NOCHAN(	NOCHAN(	UP	NOCHAN(
DOWN	NOCHAN(	NOCHAN(	UP	NOCHAN(	NOCHAN(	NOCHAN(	NOCHAN(	NOCHAN(
DOWN	NOCHAN(	DOWN	DOWN	NOCHAN(	NOCHAN(	DOWN	DOWN	DOWN
DOWN	DOWN	DOWN	NOCHAN(	DOWN	NOCHAN(	NOCHAN(	NOCHAN(	DOWN
NOCHAN(	NOCHAN(	UP	NOCHAN(	NOCHAN(	NOCHAN(	NOCHAN(	NOCHAN(	UP
UP	NOCHAN(	UP	UP	UP	NOCHAN(	NOCHAN(	UP	UP
DOWN	DOWN	NOCHAN(	DOWN	DOWN	DOWN	NOCHAN(	NOCHAN(	NOCHAN(
NOCHAN(	NOCHAN(	NOCHAN(	UP	UP	NOCHAN(	UP	NOCHAN(	NOCHAN(
NOCHAN(	DOWN	NOCHAN(	DOWN	NOCHAN(	DOWN	NOCHAN(	NOCHAN(	NOCHAN(
NOCHAN(	DOWN	NOCHAN(	NOCHAN(	NOCHAN(	NOCHAN(	NOCHAN(	NOCHAN(	NOCHAN(
UP	NOCHAN(	NOCHAN(	NOCHAN(	NOCHAN(	NOCHAN(	NOCHAN(	NOCHAN(	UP
DOWN	NOCHAN(	NOCHAN(	NOCHAN(	NOCHAN(	NOCHAN(	NOCHAN(	NOCHAN(	NOCHAN(
UP	NOCHAN(	NOCHAN(	NOCHAN(	NOCHAN(	NOCHAN(	NOCHAN(	NOCHAN(	NOCHAN(
NOCHAN(	NOCHAN(	NOCHAN(	NOCHAN(	NOCHAN(	NOCHAN(	NOCHAN(	NOCHAN(	NOCHAN(
NOCHAN(	NOCHAN(	UP	UP	NOCHAN(	UP	NOCHAN(	NOCHAN(	NOCHAN(
DOWN	NOCHAN(	DOWN	DOWN	DOWN	DOWN	DOWN	DOWN	NOCHAN(
DOWN	NOCHAN(	DOWN	NOCHAN(	DOWN	DOWN	NOCHAN(	DOWN	NOCHAN(
DOWN	NOCHAN(	DOWN	DOWN	DOWN	DOWN	NOCHAN(	DOWN	DOWN
NOCHAN(	DOWN	NOCHAN(	NOCHAN(	NOCHAN(	DOWN	DOWN	NOCHAN(	NOCHAN(
UP	NOCHAN(	UP	UP	UP	NOCHAN(	UP	UP	UP
DOWN	NOCHAN(	NOCHAN(	NOCHAN(	DOWN	UP	NOCHAN(	NOCHAN(	DOWN
NOCHAN(	NOCHAN(	NOCHAN(	NOCHAN(	NOCHAN(	NOCHAN(	NOCHAN(	NOCHAN(	DOWN
NOCHAN(	NOCHAN(	NOCHAN(	NOCHAN(	NOCHAN(	NOCHAN(	NOCHAN(	NOCHAN(	NOCHAN(
DOWN	DOWN	NOCHAN(	DOWN	DOWN	DOWN	NOCHAN(	NOCHAN(	NOCHAN(
NOCHAN(	NOCHAN(	NOCHAN(	NOCHAN(	NOCHAN(	NOCHAN(	NOCHAN(	NOCHAN(	NOCHAN(
NOCHAN(	UP	UP	NOCHAN(	UP	NOCHAN(	NOCHAN(	UP	NOCHAN(
NOCHAN(	NOCHAN(	NOCHAN(	NOCHAN(	NOCHAN(	NOCHAN(	DOWN	DOWN	NOCHAN(
UP	DOWN	NOCHAN(	NOCHAN(	NOCHAN(	NOCHAN(	DOWN	NOCHAN(	NOCHAN(
DOWN	NOCHAN(	NOCHAN(	NOCHAN(	NOCHAN(	NOCHAN(	NOCHAN(	NOCHAN(	NOCHAN(
NOCHAN(	UP	NOCHAN(	NOCHAN(	NOCHAN(	UP	UP	NOCHAN(	NOCHAN(
NOCHAN(	NOCHAN(	NOCHAN(	NOCHAN(	NOCHAN(	DOWN	NOCHAN(	NOCHAN(	NOCHAN(
UP	NOCHAN(	NOCHAN(	NOCHAN(	UP	NOCHAN(	DOWN	NOCHAN(	DOWN
NOCHAN(	NOCHAN(	NOCHAN(	NOCHAN(	NOCHAN(	NOCHAN(	NOCHAN(	NOCHAN(	UP
DOWN	NOCHAN(	DOWN	DOWN	DOWN	NOCHAN(	DOWN	DOWN	NOCHAN(
NOCHAN(	NOCHAN(	NOCHAN(	NOCHAN(	NOCHAN(	NOCHAN(	NOCHAN(	NOCHAN(	UP
UP	UP	NOCHAN(	NOCHAN(	NOCHAN(	UP	UP	UP	NOCHAN(
NOCHAN(	NOCHAN(	NOCHAN(	UP	UP	UP	UP	UP	UP

TCGA-B0-	TCGA-CZ-	TCGA-CW	TCGA-B4-	TCGA-A3-	TCGA-B0-	TCGA-CJ-	TCGA-BP-	TCGA-CJ-
NOCHAN(	DOWN	DOWN	NOCHAN(	NOCHAN(	DOWN	DOWN	NOCHAN(	DOWN
DOWN	NOCHAN(	NOCHAN(	NOCHAN(	NOCHAN(	NOCHAN(	NOCHAN(	DOWN	NOCHAN(
DOWN	NOCHAN(	DOWN	NOCHAN(	DOWN	NOCHAN(	DOWN	DOWN	DOWN
NOCHAN(	UP	NOCHAN(	NOCHAN(	NOCHAN(	NOCHAN(	NOCHAN(	NOCHAN(	NOCHAN(
NOCHAN(	NOCHAN(	NOCHAN(	NOCHAN(	NOCHAN(	NOCHAN(	NOCHAN(	NOCHAN(	NOCHAN(
DOWN	NOCHAN(	DOWN	NOCHAN(	NOCHAN(	NOCHAN(	NOCHAN(	DOWN	NOCHAN(
NOCHAN(	NOCHAN(	NOCHAN(	NOCHAN(	NOCHAN(	DOWN	DOWN	NOCHAN(	NOCHAN(
NOCHAN(	NOCHAN(	NOCHAN(	NOCHAN(	NOCHAN(	UP	UP	DOWN	NOCHAN(
NOCHAN(	NOCHAN(	NOCHAN(	NOCHAN(	NOCHAN(	UP	UP	NOCHAN(	NOCHAN(
DOWN	NOCHAN(	NOCHAN(	NOCHAN(	NOCHAN(	DOWN	DOWN	NOCHAN(	DOWN
NOCHAN(	NOCHAN(	NOCHAN(	NOCHAN(	NOCHAN(	NOCHAN(	NOCHAN(	UP	NOCHAN(
NOCHAN(	NOCHAN(	NOCHAN(	NOCHAN(	NOCHAN(	NOCHAN(	NOCHAN(	NOCHAN(	NOCHAN(
NOCHAN(	NOCHAN(	NOCHAN(	NOCHAN(	NOCHAN(	NOCHAN(	NOCHAN(	NOCHAN(	DOWN
NOCHAN(	NOCHAN(	NOCHAN(	NOCHAN(	NOCHAN(	NOCHAN(	NOCHAN(	NOCHAN(	NOCHAN(
NOCHAN(	NOCHAN(	UP	NOCHAN(	NOCHAN(	UP	NOCHAN(	DOWN	NOCHAN(
NOCHAN(	NOCHAN(	NOCHAN(	NOCHAN(	NOCHAN(	NOCHAN(	NOCHAN(	NOCHAN(	NOCHAN(
NOCHAN(	NOCHAN(	DOWN	NOCHAN(	NOCHAN(	NOCHAN(	DOWN	UP	NOCHAN(
UP	UP	NOCHAN(	NOCHAN(	NOCHAN(	NOCHAN(	NOCHAN(	NOCHAN(	UP
NOCHAN(	DOWN	DOWN	NOCHAN(	NOCHAN(	DOWN	DOWN	DOWN	DOWN
DOWN	DOWN	DOWN	DOWN	NOCHAN(	UP	DOWN	NOCHAN(	NOCHAN(
DOWN	NOCHAN(	DOWN	DOWN	NOCHAN(	DOWN	DOWN	DOWN	DOWN
NOCHAN(	DOWN	NOCHAN(	NOCHAN(	NOCHAN(	DOWN	NOCHAN(	NOCHAN(	DOWN
UP	NOCHAN(	UP	UP	UP	UP	UP	UP	UP
NOCHAN(	NOCHAN(	DOWN	UP	NOCHAN(	NOCHAN(	NOCHAN(	NOCHAN(	NOCHAN(
DOWN	NOCHAN(	NOCHAN(	NOCHAN(	NOCHAN(	NOCHAN(	NOCHAN(	NOCHAN(	NOCHAN(
DOWN	NOCHAN(	NOCHAN(	NOCHAN(	NOCHAN(	NOCHAN(	NOCHAN(	NOCHAN(	NOCHAN(
DOWN	NOCHAN(	NOCHAN(	DOWN	DOWN	NOCHAN(	DOWN	DOWN	NOCHAN(
NOCHAN(	NOCHAN(	NOCHAN(	NOCHAN(	NOCHAN(	NOCHAN(	NOCHAN(	DOWN	NOCHAN(
NOCHAN(	UP	NOCHAN(	NOCHAN(	NOCHAN(	UP	NOCHAN(	NOCHAN(	UP
DOWN	NOCHAN(	UP	NOCHAN(	NOCHAN(	UP	NOCHAN(	NOCHAN(	NOCHAN(
NOCHAN(	NOCHAN(	NOCHAN(	NOCHAN(	NOCHAN(	NOCHAN(	UP	NOCHAN(	NOCHAN(
NOCHAN(	NOCHAN(	NOCHAN(	NOCHAN(	NOCHAN(	DOWN	NOCHAN(	NOCHAN(	NOCHAN(
UP	UP	UP	UP	UP	NOCHAN(	NOCHAN(	UP	UP
NOCHAN(	NOCHAN(	NOCHAN(	NOCHAN(	NOCHAN(	NOCHAN(	NOCHAN(	NOCHAN(	NOCHAN(
NOCHAN(	NOCHAN(	NOCHAN(	NOCHAN(	UP	NOCHAN(	NOCHAN(	NOCHAN(	NOCHAN(
DOWN	NOCHAN(	NOCHAN(	NOCHAN(	NOCHAN(	DOWN	NOCHAN(	NOCHAN(	NOCHAN(
DOWN	DOWN	DOWN	NOCHAN(	NOCHAN(	DOWN	DOWN	DOWN	DOWN
NOCHAN(	UP	UP	UP	UP	NOCHAN(	UP	NOCHAN(	NOCHAN(
UP	NOCHAN(	NOCHAN(	UP	UP	NOCHAN(	NOCHAN(	NOCHAN(	UP
NOCHAN(	NOCHAN(	NOCHAN(	NOCHAN(	UP	NOCHAN(	NOCHAN(	UP	UP

TCGA-CZ-	TCGA-B0-	TCGA-CJ-	TCGA-A3-	TCGA-CJ-	TCGA-CZ-	TCGA-B8-	TCGA-BP-	TCGA-B0-
NOCHAN(	DOWN	DOWN	NOCHAN(	NOCHAN(	DOWN	NOCHAN(	NOCHAN(	DOWN
DOWN	NOCHAN(	DOWN	NOCHAN(	NOCHAN(	DOWN	NOCHAN(	NOCHAN(	NOCHAN(
DOWN	DOWN	DOWN	DOWN	DOWN	DOWN	DOWN	NOCHAN(	DOWN
NOCHAN(	NOCHAN(	UP	NOCHAN(	NOCHAN(	UP	NOCHAN(	NOCHAN(	NOCHAN(
NOCHAN(	NOCHAN(	NOCHAN(	NOCHAN(	NOCHAN(	NOCHAN(	NOCHAN(	NOCHAN(	UP
DOWN	NOCHAN(	DOWN	NOCHAN(	DOWN	DOWN	DOWN	UP	DOWN
NOCHAN(	NOCHAN(	NOCHAN(	NOCHAN(	NOCHAN(	NOCHAN(	NOCHAN(	UP	DOWN
UP	NOCHAN(	NOCHAN(	NOCHAN(	NOCHAN(	NOCHAN(	NOCHAN(	NOCHAN(	NOCHAN(
UP	NOCHAN(	UP	NOCHAN(	NOCHAN(	UP	NOCHAN(	NOCHAN(	UP
NOCHAN(	DOWN	DOWN	NOCHAN(	DOWN	DOWN	NOCHAN(	NOCHAN(	DOWN
NOCHAN(	NOCHAN(	NOCHAN(	UP	NOCHAN(	NOCHAN(	NOCHAN(	NOCHAN(	NOCHAN(
NOCHAN(	DOWN	NOCHAN(	NOCHAN(	NOCHAN(	NOCHAN(	DOWN	NOCHAN(	NOCHAN(
NOCHAN(	NOCHAN(	NOCHAN(	NOCHAN(	NOCHAN(	NOCHAN(	NOCHAN(	NOCHAN(	DOWN
NOCHAN(	NOCHAN(	NOCHAN(	NOCHAN(	NOCHAN(	UP	NOCHAN(	UP	NOCHAN(
NOCHAN(	UP	NOCHAN(	NOCHAN(	NOCHAN(	NOCHAN(	NOCHAN(	NOCHAN(	NOCHAN(
NOCHAN(	NOCHAN(	NOCHAN(	NOCHAN(	NOCHAN(	UP	NOCHAN(	NOCHAN(	NOCHAN(
NOCHAN(	NOCHAN(	NOCHAN(	UP	NOCHAN(	DOWN	NOCHAN(	NOCHAN(	NOCHAN(
NOCHAN(	UP	NOCHAN(	NOCHAN(	NOCHAN(	NOCHAN(	UP	NOCHAN(	NOCHAN(
NOCHAN(	DOWN	DOWN	NOCHAN(	DOWN	NOCHAN(	DOWN	NOCHAN(	DOWN
NOCHAN(	DOWN	DOWN	DOWN	DOWN	NOCHAN(	DOWN	NOCHAN(	DOWN
NOCHAN(	DOWN	DOWN	NOCHAN(	DOWN	DOWN	NOCHAN(	UP	DOWN
NOCHAN(	DOWN	NOCHAN(	NOCHAN(	DOWN	NOCHAN(	NOCHAN(	DOWN	DOWN
UP	UP	UP	UP	UP	UP	UP	UP	UP
NOCHAN(	NOCHAN(	DOWN	NOCHAN(	NOCHAN(	DOWN	NOCHAN(	NOCHAN(	NOCHAN(
DOWN	NOCHAN(	NOCHAN(	NOCHAN(	NOCHAN(	DOWN	NOCHAN(	NOCHAN(	NOCHAN(
NOCHAN(	DOWN	NOCHAN(	NOCHAN(	NOCHAN(	NOCHAN(	NOCHAN(	NOCHAN(	NOCHAN(
DOWN	DOWN	DOWN	NOCHAN(	NOCHAN(	DOWN	DOWN	NOCHAN(	NOCHAN(
NOCHAN(	NOCHAN(	NOCHAN(	NOCHAN(	NOCHAN(	DOWN	NOCHAN(	UP	NOCHAN(
NOCHAN(	NOCHAN(	NOCHAN(	UP	NOCHAN(	UP	NOCHAN(	UP	NOCHAN(
NOCHAN(	UP	NOCHAN(	NOCHAN(	NOCHAN(	NOCHAN(	NOCHAN(	NOCHAN(	NOCHAN(
NOCHAN(	NOCHAN(	NOCHAN(	NOCHAN(	NOCHAN(	UP	NOCHAN(	DOWN	NOCHAN(
NOCHAN(	NOCHAN(	DOWN	NOCHAN(	NOCHAN(	DOWN	NOCHAN(	NOCHAN(	NOCHAN(
NOCHAN(	UP	UP	NOCHAN(	NOCHAN(	NOCHAN(	UP	NOCHAN(	NOCHAN(
NOCHAN(	NOCHAN(	NOCHAN(	NOCHAN(	NOCHAN(	UP	NOCHAN(	NOCHAN(	NOCHAN(
NOCHAN(	NOCHAN(	NOCHAN(	NOCHAN(	NOCHAN(	UP	DOWN	DOWN	UP
DOWN	DOWN	NOCHAN(	NOCHAN(	NOCHAN(	NOCHAN(	NOCHAN(	NOCHAN(	DOWN
DOWN	DOWN	DOWN	DOWN	DOWN	DOWN	DOWN	NOCHAN(	DOWN
UP	UP	UP	UP	NOCHAN(	UP	NOCHAN(	NOCHAN(	NOCHAN(
NOCHAN(	NOCHAN(	NOCHAN(	NOCHAN(	UP	NOCHAN(	UP	UP	NOCHAN(
NOCHAN(	NOCHAN(	NOCHAN(	UP	NOCHAN(	NOCHAN(	NOCHAN(	UP	UP

TCGA-BP-	TCGA-B0-	TCGA-CZ-	TCGA-CZ-	TCGA-CJ-	TCGA-AK-	TCGA-B0-	TCGA-CJ-	TCGA-B0-
NOCHANG	DOWN	NOCHANG	DOWN	NOCHANG	NOCHANG	DOWN	DOWN	NOCHANG
NOCHANG	DOWN	DOWN	NOCHANG	DOWN	NOCHANG	DOWN	NOCHANG	NOCHANG
DOWN	DOWN	DOWN	DOWN	DOWN	DOWN	DOWN	DOWN	DOWN
UP	NOCHANG	NOCHANG	NOCHANG	NOCHANG	NOCHANG	NOCHANG	NOCHANG	NOCHANG
UP	DOWN	NOCHANG	NOCHANG	NOCHANG	NOCHANG	NOCHANG	NOCHANG	DOWN
DOWN	DOWN	DOWN	NOCHANG	DOWN	DOWN	DOWN	NOCHANG	UP
NOCHANG	DOWN	DOWN	DOWN	DOWN	DOWN	DOWN	NOCHANG	NOCHANG
NOCHANG	NOCHANG	NOCHANG	NOCHANG	NOCHANG	NOCHANG	NOCHANG	NOCHANG	NOCHANG
UP	UP	NOCHANG	NOCHANG	UP	UP	UP	NOCHANG	NOCHANG
DOWN	DOWN	NOCHANG	DOWN	NOCHANG	DOWN	DOWN	NOCHANG	DOWN
NOCHANG	NOCHANG	NOCHANG	NOCHANG	NOCHANG	NOCHANG	NOCHANG	NOCHANG	NOCHANG
NOCHANG	NOCHANG	NOCHANG	NOCHANG	NOCHANG	NOCHANG	DOWN	NOCHANG	UP
NOCHANG	DOWN	NOCHANG	NOCHANG	NOCHANG	NOCHANG	NOCHANG	NOCHANG	DOWN
NOCHANG	UP	NOCHANG	NOCHANG	NOCHANG	NOCHANG	NOCHANG	NOCHANG	NOCHANG
NOCHANG	DOWN	NOCHANG	NOCHANG	NOCHANG	DOWN	DOWN	NOCHANG	NOCHANG
NOCHANG	UP	DOWN	NOCHANG	NOCHANG	UP	UP	NOCHANG	UP
UP	DOWN	NOCHANG	DOWN	NOCHANG	NOCHANG	NOCHANG	NOCHANG	NOCHANG
UP	NOCHANG	NOCHANG	NOCHANG	NOCHANG	NOCHANG	NOCHANG	UP	NOCHANG
DOWN	DOWN	NOCHANG	NOCHANG	DOWN	NOCHANG	DOWN	NOCHANG	UP
NOCHANG	DOWN	DOWN	NOCHANG	DOWN	DOWN	DOWN	NOCHANG	NOCHANG
DOWN	DOWN	DOWN	NOCHANG	DOWN	DOWN	DOWN	NOCHANG	NOCHANG
NOCHANG	NOCHANG	NOCHANG	NOCHANG	NOCHANG	NOCHANG	NOCHANG	NOCHANG	DOWN
UP	UP	UP	UP	UP	UP	UP	UP	NOCHANG
NOCHANG	DOWN	NOCHANG	DOWN	NOCHANG	DOWN	NOCHANG	NOCHANG	UP
NOCHANG	DOWN	NOCHANG	NOCHANG	DOWN	NOCHANG	NOCHANG	NOCHANG	NOCHANG
NOCHANG	DOWN	NOCHANG	NOCHANG	NOCHANG	DOWN	DOWN	NOCHANG	NOCHANG
NOCHANG	DOWN	DOWN	DOWN	DOWN	DOWN	DOWN	DOWN	DOWN
NOCHANG	DOWN	NOCHANG	NOCHANG	NOCHANG	NOCHANG	DOWN	NOCHANG	UP
NOCHANG	NOCHANG	NOCHANG	UP	NOCHANG	NOCHANG	NOCHANG	NOCHANG	UP
DOWN	NOCHANG	NOCHANG	NOCHANG	NOCHANG	NOCHANG	DOWN	NOCHANG	NOCHANG
NOCHANG	UP	NOCHANG	UP	UP	UP	UP	NOCHANG	NOCHANG
NOCHANG	DOWN	NOCHANG	NOCHANG	DOWN	NOCHANG	DOWN	NOCHANG	DOWN
NOCHANG	NOCHANG	UP	NOCHANG	NOCHANG	NOCHANG	NOCHANG	UP	DOWN
NOCHANG	UP	NOCHANG	NOCHANG	NOCHANG	NOCHANG	NOCHANG	NOCHANG	NOCHANG
NOCHANG	UP	DOWN	UP	NOCHANG	UP	UP	UP	DOWN
NOCHANG	NOCHANG	NOCHANG	DOWN	NOCHANG	NOCHANG	DOWN	NOCHANG	NOCHANG
DOWN	DOWN	DOWN	DOWN	NOCHANG	NOCHANG	DOWN	DOWN	DOWN
UP	NOCHANG	UP	UP	UP	UP	NOCHANG	UP	NOCHANG
NOCHANG	NOCHANG	UP	NOCHANG	NOCHANG	NOCHANG	DOWN	UP	UP
UP	NOCHANG	NOCHANG	DOWN	NOCHANG	DOWN	NOCHANG	NOCHANG	NOCHANG

TCGA-B0-	TCGA-BP-	TCGA-CW	TCGA-BP-	TCGA-B0-	TCGA-CJ-	TCGA-B0-	TCGA-B0-	TCGA-B0-
NOCHAN(	NOCHAN(	DOWN	DOWN	DOWN	DOWN	DOWN	DOWN	DOWN
DOWN	DOWN	DOWN	DOWN	DOWN	NOCHAN(	DOWN	NOCHAN(	DOWN
DOWN	DOWN	DOWN	DOWN	DOWN	DOWN	DOWN	DOWN	DOWN
UP	NOCHAN(	NOCHAN(	NOCHAN(	UP	NOCHAN(	NOCHAN(	NOCHAN(	UP
DOWN	NOCHAN(	NOCHAN(	NOCHAN(	NOCHAN(	DOWN	NOCHAN(	NOCHAN(	UP
NOCHAN(	NOCHAN(	DOWN	DOWN	DOWN	DOWN	DOWN	DOWN	DOWN
UP	NOCHAN(	NOCHAN(	DOWN	NOCHAN(	DOWN	NOCHAN(	DOWN	NOCHAN(
NOCHAN(	NOCHAN(	NOCHAN(	NOCHAN(	NOCHAN(	NOCHAN(	DOWN	NOCHAN(	NOCHAN(
NOCHAN(	NOCHAN(	UP	UP	NOCHAN(	NOCHAN(	NOCHAN(	NOCHAN(	UP
DOWN	NOCHAN(	NOCHAN(	DOWN	DOWN	DOWN	NOCHAN(	DOWN	DOWN
NOCHAN(	UP	NOCHAN(	NOCHAN(	NOCHAN(	NOCHAN(	UP	UP	NOCHAN(
NOCHAN(	NOCHAN(	NOCHAN(	NOCHAN(	DOWN	NOCHAN(	NOCHAN(	NOCHAN(	NOCHAN(
DOWN	NOCHAN(	NOCHAN(	NOCHAN(	NOCHAN(	NOCHAN(	NOCHAN(	NOCHAN(	DOWN
NOCHAN(	NOCHAN(	NOCHAN(	NOCHAN(	NOCHAN(	NOCHAN(	NOCHAN(	NOCHAN(	NOCHAN(
NOCHAN(	NOCHAN(	UP	DOWN	NOCHAN(	NOCHAN(	NOCHAN(	NOCHAN(	NOCHAN(
NOCHAN(	NOCHAN(	NOCHAN(	NOCHAN(	NOCHAN(	NOCHAN(	NOCHAN(	NOCHAN(	UP
NOCHAN(	UP	NOCHAN(	NOCHAN(	UP	DOWN	NOCHAN(	NOCHAN(	UP
DOWN	UP	UP	NOCHAN(	UP	NOCHAN(	NOCHAN(	UP	NOCHAN(
NOCHAN(	NOCHAN(	DOWN	DOWN	DOWN	DOWN	DOWN	DOWN	DOWN
DOWN	NOCHAN(	NOCHAN(	DOWN	NOCHAN(	DOWN	NOCHAN(	DOWN	DOWN
NOCHAN(	NOCHAN(	DOWN	DOWN	DOWN	DOWN	NOCHAN(	DOWN	DOWN
DOWN	NOCHAN(	NOCHAN(	NOCHAN(	NOCHAN(	NOCHAN(	NOCHAN(	NOCHAN(	NOCHAN(
DOWN	UP	UP	UP	UP	UP	UP	UP	UP
NOCHAN(	NOCHAN(	NOCHAN(	DOWN	NOCHAN(	DOWN	NOCHAN(	NOCHAN(	DOWN
NOCHAN(	NOCHAN(	NOCHAN(	DOWN	NOCHAN(	NOCHAN(	NOCHAN(	NOCHAN(	NOCHAN(
UP	NOCHAN(	NOCHAN(	DOWN	NOCHAN(	DOWN	NOCHAN(	NOCHAN(	NOCHAN(
DOWN	DOWN	DOWN	DOWN	DOWN	DOWN	DOWN	NOCHAN(	NOCHAN(
UP	NOCHAN(	NOCHAN(	DOWN	NOCHAN(	DOWN	NOCHAN(	DOWN	DOWN
UP	NOCHAN(	NOCHAN(	NOCHAN(	NOCHAN(	NOCHAN(	NOCHAN(	NOCHAN(	NOCHAN(
NOCHAN(	NOCHAN(	NOCHAN(	NOCHAN(	NOCHAN(	NOCHAN(	UP	NOCHAN(	NOCHAN(
DOWN	NOCHAN(	NOCHAN(	UP	NOCHAN(	NOCHAN(	NOCHAN(	NOCHAN(	UP
DOWN	NOCHAN(	NOCHAN(	DOWN	NOCHAN(	DOWN	NOCHAN(	NOCHAN(	DOWN
DOWN	UP	UP	NOCHAN(	UP	NOCHAN(	UP	NOCHAN(	UP
NOCHAN(	NOCHAN(	NOCHAN(	NOCHAN(	NOCHAN(	NOCHAN(	NOCHAN(	NOCHAN(	NOCHAN(
DOWN	NOCHAN(	NOCHAN(	NOCHAN(	NOCHAN(	NOCHAN(	NOCHAN(	UP	NOCHAN(
NOCHAN(	NOCHAN(	NOCHAN(	DOWN	DOWN	NOCHAN(	NOCHAN(	DOWN	DOWN
NOCHAN(	DOWN	DOWN	DOWN	DOWN	DOWN	DOWN	DOWN	DOWN
DOWN	UP	UP	NOCHAN(	NOCHAN(	UP	UP	NOCHAN(	NOCHAN(
DOWN	UP	UP	DOWN	NOCHAN(	NOCHAN(	UP	NOCHAN(	DOWN
NOCHAN(	NOCHAN(	NOCHAN(	NOCHAN(	UP	NOCHAN(	NOCHAN(	UP	UP

TCGA-CZ-	TCGA-BP-	TCGA-CZ-	TCGA-A3-	TCGA-B0-	TCGA-BP-	TCGA-CW	TCGA-AK-	TCGA-AK-
NOCHAN(	DOWN	DOWN	DOWN	NOCHAN(	DOWN	NOCHAN(	NOCHAN(	DOWN
NOCHAN(	NOCHAN(	NOCHAN(	NOCHAN(	NOCHAN(	NOCHAN(	NOCHAN(	DOWN	DOWN
DOWN	DOWN	UP	NOCHAN(	DOWN	DOWN	DOWN	DOWN	DOWN
NOCHAN(	NOCHAN(	DOWN	NOCHAN(	NOCHAN(	NOCHAN(	NOCHAN(	NOCHAN(	NOCHAN(
DOWN	NOCHAN(	DOWN	NOCHAN(	NOCHAN(	NOCHAN(	NOCHAN(	DOWN	NOCHAN(
NOCHAN(	NOCHAN(	UP	NOCHAN(	DOWN	DOWN	DOWN	UP	DOWN
DOWN	NOCHAN(	NOCHAN(	NOCHAN(	DOWN	DOWN	DOWN	UP	DOWN
NOCHAN(	NOCHAN(	DOWN	NOCHAN(	UP	NOCHAN(	NOCHAN(	DOWN	NOCHAN(
UP	NOCHAN(	DOWN	UP	UP	NOCHAN(	NOCHAN(	NOCHAN(	UP
NOCHAN(	NOCHAN(	UP	DOWN	NOCHAN(	NOCHAN(	NOCHAN(	DOWN	DOWN
NOCHAN(	NOCHAN(	UP	UP	NOCHAN(	NOCHAN(	NOCHAN(	NOCHAN(	NOCHAN(
NOCHAN(	NOCHAN(	DOWN	DOWN	NOCHAN(	DOWN	NOCHAN(	UP	NOCHAN(
NOCHAN(	NOCHAN(	UP	NOCHAN(	NOCHAN(	DOWN	NOCHAN(	NOCHAN(	NOCHAN(
UP	NOCHAN(	DOWN	NOCHAN(	NOCHAN(	NOCHAN(	UP	UP	NOCHAN(
NOCHAN(	NOCHAN(	DOWN	NOCHAN(	NOCHAN(	NOCHAN(	NOCHAN(	DOWN	NOCHAN(
NOCHAN(	NOCHAN(	DOWN	DOWN	NOCHAN(	NOCHAN(	NOCHAN(	UP	NOCHAN(
NOCHAN(	NOCHAN(	NOCHAN(	NOCHAN(	NOCHAN(	NOCHAN(	NOCHAN(	DOWN	DOWN
NOCHAN(	UP	NOCHAN(	UP	UP	NOCHAN(	UP	DOWN	NOCHAN(
NOCHAN(	DOWN	UP	DOWN	DOWN	NOCHAN(	NOCHAN(	UP	DOWN
DOWN	DOWN	DOWN	NOCHAN(	DOWN	NOCHAN(	DOWN	NOCHAN(	UP
NOCHAN(	NOCHAN(	UP	DOWN	NOCHAN(	DOWN	NOCHAN(	DOWN	DOWN
NOCHAN(	NOCHAN(	NOCHAN(	NOCHAN(	DOWN	DOWN	NOCHAN(	NOCHAN(	NOCHAN(
UP	UP	DOWN	UP	UP	NOCHAN(	UP	UP	UP
NOCHAN(	NOCHAN(	UP	NOCHAN(	DOWN	NOCHAN(	NOCHAN(	DOWN	NOCHAN(
NOCHAN(	NOCHAN(	UP	NOCHAN(	DOWN	NOCHAN(	NOCHAN(	NOCHAN(	NOCHAN(
NOCHAN(	DOWN	DOWN	NOCHAN(	NOCHAN(	NOCHAN(	NOCHAN(	DOWN	NOCHAN(
DOWN	DOWN	DOWN	DOWN	NOCHAN(	DOWN	NOCHAN(	DOWN	DOWN
NOCHAN(	NOCHAN(	DOWN	NOCHAN(	NOCHAN(	NOCHAN(	NOCHAN(	UP	NOCHAN(
NOCHAN(	NOCHAN(	DOWN	NOCHAN(	UP	UP	NOCHAN(	DOWN	NOCHAN(
NOCHAN(	DOWN	NOCHAN(	NOCHAN(	DOWN	NOCHAN(	DOWN	UP	DOWN
UP	DOWN	DOWN	NOCHAN(	NOCHAN(	NOCHAN(	NOCHAN(	NOCHAN(	NOCHAN(
NOCHAN(	NOCHAN(	UP	NOCHAN(	DOWN	NOCHAN(	NOCHAN(	NOCHAN(	DOWN
NOCHAN(	NOCHAN(	UP	UP	NOCHAN(	UP	NOCHAN(	DOWN	NOCHAN(
NOCHAN(	NOCHAN(	DOWN	NOCHAN(	NOCHAN(	NOCHAN(	NOCHAN(	UP	NOCHAN(
UP	NOCHAN(	DOWN	NOCHAN(	UP	NOCHAN(	UP	NOCHAN(	NOCHAN(
NOCHAN(	NOCHAN(	NOCHAN(	DOWN	NOCHAN(	DOWN	NOCHAN(	NOCHAN(	NOCHAN(
DOWN	DOWN	NOCHAN(	DOWN	DOWN	NOCHAN(	DOWN	NOCHAN(	DOWN
UP	UP	UP	UP	NOCHAN(	NOCHAN(	NOCHAN(	NOCHAN(	NOCHAN(
UP	UP	NOCHAN(	UP	NOCHAN(	UP	UP	NOCHAN(	NOCHAN(
UP	NOCHAN(	DOWN	UP	NOCHAN(	UP	UP	DOWN	NOCHAN(

TCGA-BP-	TCGA-BP-	TCGA-BP-	TCGA-B0-	TCGA-CJ-	TCGA-BP-	TCGA-B0-	TCGA-A3-	TCGA-B0-
DOWN	DOWN	DOWN	NOCHAN(	DOWN	DOWN	DOWN	NOCHAN(	DOWN
NOCHAN(	DOWN	DOWN	NOCHAN(	NOCHAN(	NOCHAN(	DOWN	NOCHAN(	DOWN
NOCHAN(	DOWN	DOWN	DOWN	DOWN	DOWN	DOWN	DOWN	DOWN
DOWN	NOCHAN(	NOCHAN(	NOCHAN(	NOCHAN(	NOCHAN(	UP	NOCHAN(	NOCHAN(
NOCHAN(	NOCHAN(	NOCHAN(	NOCHAN(	NOCHAN(	DOWN	NOCHAN(	NOCHAN(	NOCHAN(
NOCHAN(	DOWN	NOCHAN(	NOCHAN(	DOWN	DOWN	DOWN	NOCHAN(	DOWN
NOCHAN(	DOWN	DOWN	NOCHAN(	NOCHAN(	NOCHAN(	NOCHAN(	NOCHAN(	NOCHAN(
NOCHAN(	NOCHAN(	NOCHAN(	NOCHAN(	UP	NOCHAN(	NOCHAN(	NOCHAN(	NOCHAN(
NOCHAN(	UP	NOCHAN(	UP	UP	NOCHAN(	UP	NOCHAN(	UP
NOCHAN(	DOWN	NOCHAN(	NOCHAN(	DOWN	DOWN	DOWN	DOWN	DOWN
NOCHAN(	NOCHAN(	UP	NOCHAN(	NOCHAN(	NOCHAN(	UP	NOCHAN(	UP
DOWN	NOCHAN(	DOWN	NOCHAN(	NOCHAN(	NOCHAN(	NOCHAN(	NOCHAN(	NOCHAN(
NOCHAN(	NOCHAN(	NOCHAN(	NOCHAN(	NOCHAN(	NOCHAN(	NOCHAN(	NOCHAN(	NOCHAN(
NOCHAN(	NOCHAN(	NOCHAN(	NOCHAN(	NOCHAN(	NOCHAN(	NOCHAN(	NOCHAN(	UP
DOWN	DOWN	NOCHAN(	NOCHAN(	NOCHAN(	NOCHAN(	DOWN	DOWN	DOWN
NOCHAN(	UP	NOCHAN(	NOCHAN(	NOCHAN(	NOCHAN(	NOCHAN(	UP	NOCHAN(
DOWN	NOCHAN(	NOCHAN(	NOCHAN(	NOCHAN(	DOWN	UP	NOCHAN(	DOWN
NOCHAN(	NOCHAN(	NOCHAN(	UP	NOCHAN(	NOCHAN(	NOCHAN(	UP	NOCHAN(
NOCHAN(	DOWN	DOWN	DOWN	DOWN	DOWN	DOWN	NOCHAN(	DOWN
DOWN	DOWN	DOWN	DOWN	DOWN	DOWN	DOWN	DOWN	DOWN
DOWN	DOWN	NOCHAN(	NOCHAN(	DOWN	DOWN	DOWN	DOWN	DOWN
NOCHAN(	NOCHAN(	NOCHAN(	NOCHAN(	NOCHAN(	NOCHAN(	NOCHAN(	DOWN	NOCHAN(
UP	UP	UP	UP	UP	UP	UP	UP	UP
NOCHAN(	DOWN	NOCHAN(	NOCHAN(	DOWN	DOWN	DOWN	NOCHAN(	DOWN
NOCHAN(	DOWN	NOCHAN(	DOWN	NOCHAN(	NOCHAN(	NOCHAN(	NOCHAN(	DOWN
DOWN	NOCHAN(	NOCHAN(	NOCHAN(	NOCHAN(	DOWN	NOCHAN(	NOCHAN(	NOCHAN(
DOWN	DOWN	DOWN	DOWN	DOWN	DOWN	DOWN	DOWN	DOWN
NOCHAN(	DOWN	NOCHAN(	NOCHAN(	NOCHAN(	NOCHAN(	DOWN	NOCHAN(	NOCHAN(
NOCHAN(	NOCHAN(	UP	NOCHAN(	NOCHAN(	NOCHAN(	NOCHAN(	NOCHAN(	DOWN
NOCHAN(	NOCHAN(	NOCHAN(	NOCHAN(	DOWN	NOCHAN(	DOWN	DOWN	UP
NOCHAN(	UP	DOWN	NOCHAN(	NOCHAN(	NOCHAN(	UP	DOWN	UP
UP	DOWN	NOCHAN(	NOCHAN(	NOCHAN(	NOCHAN(	DOWN	NOCHAN(	DOWN
UP	NOCHAN(	UP	NOCHAN(	NOCHAN(	NOCHAN(	NOCHAN(	NOCHAN(	NOCHAN(
NOCHAN(	UP	NOCHAN(	NOCHAN(	NOCHAN(	NOCHAN(	NOCHAN(	NOCHAN(	UP
NOCHAN(	NOCHAN(	NOCHAN(	UP	UP	UP	DOWN	UP	NOCHAN(
NOCHAN(	NOCHAN(	NOCHAN(	NOCHAN(	NOCHAN(	DOWN	NOCHAN(	NOCHAN(	DOWN
NOCHAN(	DOWN	DOWN	DOWN	DOWN	DOWN	DOWN	DOWN	DOWN
UP	NOCHAN(	NOCHAN(	NOCHAN(	UP	NOCHAN(	DOWN	UP	NOCHAN(
UP	NOCHAN(	UP	UP	UP	UP	DOWN	UP	NOCHAN(
DOWN	NOCHAN(	NOCHAN(	NOCHAN(	NOCHAN(	DOWN	UP	UP	NOCHAN(

TCGA-B0-	TCGA-A3-	TCGA-CJ-	TCGA-CJ-	TCGA-A3-	TCGA-B8-	TCGA-BP-	TCGA-B0-	TCGA-CW
NOCHAN(	DOWN	NOCHAN(	DOWN	NOCHAN(	NOCHAN(	NOCHAN(	NOCHAN(	DOWN
DOWN	NOCHAN(	NOCHAN(	DOWN	DOWN	DOWN	DOWN	NOCHAN(	NOCHAN(
DOWN	DOWN	DOWN	DOWN	DOWN	DOWN	DOWN	DOWN	NOCHAN(
NOCHAN(	NOCHAN(	NOCHAN(	NOCHAN(	NOCHAN(	UP	UP	NOCHAN(	NOCHAN(
NOCHAN(	UP	NOCHAN(	NOCHAN(	NOCHAN(	NOCHAN(	UP	DOWN	NOCHAN(
DOWN	NOCHAN(	DOWN	DOWN	DOWN	DOWN	DOWN	DOWN	NOCHAN(
DOWN	NOCHAN(	DOWN	NOCHAN(	DOWN	DOWN	DOWN	NOCHAN(	NOCHAN(
NOCHAN(	NOCHAN(	NOCHAN(	NOCHAN(	NOCHAN(	NOCHAN(	UP	NOCHAN(	NOCHAN(
UP	NOCHAN(	UP	UP	NOCHAN(	NOCHAN(	UP	UP	UP
NOCHAN(	NOCHAN(	DOWN	DOWN	NOCHAN(	DOWN	DOWN	NOCHAN(	NOCHAN(
NOCHAN(	UP	NOCHAN(	NOCHAN(	UP	NOCHAN(	UP	NOCHAN(	UP
NOCHAN(	NOCHAN(	NOCHAN(	NOCHAN(	NOCHAN(	DOWN	DOWN	NOCHAN(	NOCHAN(
NOCHAN(	NOCHAN(	NOCHAN(	NOCHAN(	NOCHAN(	DOWN	NOCHAN(	NOCHAN(	NOCHAN(
NOCHAN(	NOCHAN(	UP	NOCHAN(	NOCHAN(	NOCHAN(	NOCHAN(	NOCHAN(	NOCHAN(
NOCHAN(	NOCHAN(	NOCHAN(	NOCHAN(	NOCHAN(	NOCHAN(	DOWN	NOCHAN(	NOCHAN(
UP	DOWN	NOCHAN(	NOCHAN(	UP	NOCHAN(	NOCHAN(	NOCHAN(	NOCHAN(
NOCHAN(	NOCHAN(	NOCHAN(	DOWN	UP	UP	UP	NOCHAN(	UP
NOCHAN(	UP	NOCHAN(	NOCHAN(	NOCHAN(	NOCHAN(	NOCHAN(	NOCHAN(	UP
DOWN	NOCHAN(	DOWN	NOCHAN(	DOWN	NOCHAN(	DOWN	DOWN	NOCHAN(
DOWN	NOCHAN(	DOWN	DOWN	NOCHAN(	UP	DOWN	DOWN	DOWN
NOCHAN(	DOWN	DOWN	DOWN	NOCHAN(	DOWN	DOWN	NOCHAN(	NOCHAN(
NOCHAN(	NOCHAN(	NOCHAN(	NOCHAN(	NOCHAN(	NOCHAN(	DOWN	NOCHAN(	NOCHAN(
UP	UP	UP	UP	UP	NOCHAN(	UP	UP	NOCHAN(
DOWN	NOCHAN(	DOWN	DOWN	NOCHAN(	NOCHAN(	DOWN	DOWN	NOCHAN(
NOCHAN(	NOCHAN(	NOCHAN(	NOCHAN(	NOCHAN(	NOCHAN(	NOCHAN(	NOCHAN(	NOCHAN(
NOCHAN(	NOCHAN(	NOCHAN(	NOCHAN(	NOCHAN(	NOCHAN(	NOCHAN(	NOCHAN(	NOCHAN(
DOWN	NOCHAN(	DOWN	DOWN	DOWN	DOWN	DOWN	DOWN	NOCHAN(
NOCHAN(	NOCHAN(	NOCHAN(	NOCHAN(	NOCHAN(	NOCHAN(	DOWN	NOCHAN(	NOCHAN(
NOCHAN(	NOCHAN(	NOCHAN(	NOCHAN(	NOCHAN(	UP	UP	NOCHAN(	NOCHAN(
NOCHAN(	NOCHAN(	DOWN	UP	DOWN	NOCHAN(	DOWN	DOWN	DOWN
NOCHAN(	NOCHAN(	UP	UP	NOCHAN(	DOWN	NOCHAN(	NOCHAN(	NOCHAN(
NOCHAN(	NOCHAN(	DOWN	DOWN	NOCHAN(	NOCHAN(	DOWN	NOCHAN(	NOCHAN(
NOCHAN(	UP	NOCHAN(	NOCHAN(	UP	UP	UP	NOCHAN(	UP
NOCHAN(	NOCHAN(	NOCHAN(	UP	NOCHAN(	NOCHAN(	NOCHAN(	NOCHAN(	NOCHAN(
UP	DOWN	NOCHAN(	NOCHAN(	NOCHAN(	NOCHAN(	DOWN	UP	NOCHAN(
NOCHAN(	NOCHAN(	NOCHAN(	NOCHAN(	NOCHAN(	NOCHAN(	DOWN	NOCHAN(	NOCHAN(
DOWN	NOCHAN(	DOWN	DOWN	DOWN	NOCHAN(	DOWN	DOWN	DOWN
NOCHAN(	UP	NOCHAN(	UP	UP	NOCHAN(	DOWN	NOCHAN(	UP
UP	UP	NOCHAN(	DOWN	UP	UP	DOWN	UP	UP
NOCHAN(	UP	NOCHAN(	NOCHAN(	NOCHAN(	UP	UP	NOCHAN(	UP

TCGA-BP-	TCGA-BP-	TCGA-CZ-	TCGA-B8-	TCGA-BP-	TCGA-BP-	TCGA-B8-	TCGA-B0-	TCGA-BP-
DOWN	DOWN	UP	NOCHANG	NOCHANG	NOCHANG	DOWN	DOWN	NOCHANG
DOWN	DOWN	NOCHANG	NOCHANG	NOCHANG	DOWN	NOCHANG	DOWN	NOCHANG
DOWN	DOWN	DOWN	NOCHANG	NOCHANG	DOWN	NOCHANG	DOWN	DOWN
NOCHANG	UP	NOCHANG	NOCHANG	NOCHANG	NOCHANG	NOCHANG	UP	UP
NOCHANG	UP	NOCHANG	NOCHANG	NOCHANG	NOCHANG	NOCHANG	NOCHANG	NOCHANG
DOWN	DOWN	DOWN	DOWN	NOCHANG	NOCHANG	NOCHANG	DOWN	NOCHANG
DOWN	NOCHANG	DOWN	DOWN	UP	NOCHANG	DOWN	DOWN	NOCHANG
NOCHANG	NOCHANG	NOCHANG	UP	NOCHANG	NOCHANG	NOCHANG	NOCHANG	NOCHANG
UP	NOCHANG	NOCHANG	NOCHANG	NOCHANG	UP	NOCHANG	UP	UP
DOWN	DOWN	NOCHANG	DOWN	NOCHANG	DOWN	DOWN	DOWN	DOWN
UP	NOCHANG	NOCHANG	NOCHANG	NOCHANG	NOCHANG	NOCHANG	UP	NOCHANG
NOCHANG	DOWN	NOCHANG	NOCHANG	NOCHANG	NOCHANG	NOCHANG	NOCHANG	DOWN
NOCHANG	NOCHANG	NOCHANG	NOCHANG	NOCHANG	NOCHANG	NOCHANG	NOCHANG	DOWN
NOCHANG	NOCHANG	NOCHANG	UP	NOCHANG	NOCHANG	NOCHANG	NOCHANG	NOCHANG
DOWN	NOCHANG	NOCHANG	NOCHANG	NOCHANG	NOCHANG	NOCHANG	DOWN	NOCHANG
NOCHANG	NOCHANG	NOCHANG	DOWN	NOCHANG	NOCHANG	UP	NOCHANG	NOCHANG
NOCHANG	UP	UP	NOCHANG	NOCHANG	UP	NOCHANG	UP	NOCHANG
NOCHANG	NOCHANG	UP	NOCHANG	NOCHANG	NOCHANG	NOCHANG	NOCHANG	UP
DOWN	DOWN	NOCHANG	DOWN	NOCHANG	NOCHANG	DOWN	DOWN	DOWN
DOWN	NOCHANG	NOCHANG	DOWN	NOCHANG	NOCHANG	DOWN	DOWN	NOCHANG
DOWN	DOWN	DOWN	NOCHANG	UP	DOWN	DOWN	DOWN	NOCHANG
NOCHANG	NOCHANG	NOCHANG	NOCHANG	NOCHANG	DOWN	DOWN	NOCHANG	DOWN
UP	UP	UP	UP	UP	UP	UP	UP	UP
DOWN	NOCHANG	NOCHANG	DOWN	NOCHANG	NOCHANG	NOCHANG	DOWN	NOCHANG
NOCHANG	NOCHANG	NOCHANG	NOCHANG	NOCHANG	NOCHANG	NOCHANG	NOCHANG	NOCHANG
DOWN	NOCHANG	NOCHANG	NOCHANG	NOCHANG	NOCHANG	DOWN	NOCHANG	NOCHANG
DOWN	DOWN	NOCHANG	NOCHANG	NOCHANG	DOWN	DOWN	DOWN	DOWN
DOWN	NOCHANG	UP	NOCHANG	NOCHANG	NOCHANG	NOCHANG	DOWN	NOCHANG
NOCHANG	NOCHANG	NOCHANG	NOCHANG	UP	NOCHANG	NOCHANG	NOCHANG	UP
DOWN	NOCHANG	NOCHANG	NOCHANG	NOCHANG	NOCHANG	NOCHANG	DOWN	NOCHANG
UP	NOCHANG	NOCHANG	NOCHANG	DOWN	NOCHANG	NOCHANG	UP	NOCHANG
DOWN	NOCHANG	NOCHANG	DOWN	NOCHANG	NOCHANG	DOWN	DOWN	NOCHANG
NOCHANG	UP	UP	NOCHANG	NOCHANG	NOCHANG	NOCHANG	NOCHANG	UP
NOCHANG	NOCHANG	NOCHANG	NOCHANG	NOCHANG	DOWN	NOCHANG	UP	NOCHANG
NOCHANG	NOCHANG	UP	NOCHANG	DOWN	UP	NOCHANG	DOWN	NOCHANG
NOCHANG	DOWN	NOCHANG	NOCHANG	NOCHANG	NOCHANG	NOCHANG	DOWN	NOCHANG
DOWN	NOCHANG	NOCHANG	DOWN	NOCHANG	DOWN	DOWN	DOWN	NOCHANG
NOCHANG	NOCHANG	UP	UP	NOCHANG	NOCHANG	NOCHANG	NOCHANG	NOCHANG
NOCHANG	NOCHANG	NOCHANG	NOCHANG	UP	UP	UP	DOWN	NOCHANG
NOCHANG	UP	NOCHANG	NOCHANG	UP	NOCHANG	NOCHANG	NOCHANG	UP

TCGA-CZ-	TCGA-CJ-	TCGA-AK-	TCGA-BP-	TCGA-CW	TCGA-B2-	TCGA-CJ-	TCGA-B0-	TCGA-CJ-
DOWN	DOWN	DOWN	DOWN	NOCHAN(	NOCHAN(	DOWN	DOWN	NOCHAN(
NOCHAN(	NOCHAN(	DOWN	DOWN	DOWN	NOCHAN(	NOCHAN(	DOWN	DOWN
NOCHAN(	DOWN	DOWN	DOWN	DOWN	DOWN	DOWN	DOWN	DOWN
NOCHAN(	NOCHAN(	UP	NOCHAN(	NOCHAN(	NOCHAN(	NOCHAN(	UP	NOCHAN(
NOCHAN(	DOWN	NOCHAN(	NOCHAN(	NOCHAN(	NOCHAN(	DOWN	DOWN	NOCHAN(
NOCHAN(	DOWN	DOWN	DOWN	DOWN	DOWN	DOWN	DOWN	DOWN
NOCHAN(	DOWN	NOCHAN(	DOWN	NOCHAN(	DOWN	DOWN	DOWN	DOWN
NOCHAN(	NOCHAN(	UP	NOCHAN(	UP	NOCHAN(	DOWN	DOWN	NOCHAN(
NOCHAN(	UP	UP	NOCHAN(	UP	NOCHAN(	UP	UP	UP
NOCHAN(	DOWN	DOWN	NOCHAN(	NOCHAN(	DOWN	DOWN	DOWN	DOWN
NOCHAN(	NOCHAN(	UP	UP	NOCHAN(	UP	NOCHAN(	NOCHAN(	UP
DOWN	NOCHAN(	DOWN	NOCHAN(	NOCHAN(	NOCHAN(	UP	UP	NOCHAN(
NOCHAN(	NOCHAN(	DOWN	NOCHAN(	NOCHAN(	NOCHAN(	NOCHAN(	DOWN	NOCHAN(
NOCHAN(	NOCHAN(	NOCHAN(	NOCHAN(	NOCHAN(	NOCHAN(	NOCHAN(	UP	UP
UP	NOCHAN(	DOWN	NOCHAN(	NOCHAN(	NOCHAN(	NOCHAN(	DOWN	DOWN
NOCHAN(	UP	NOCHAN(	UP	NOCHAN(	NOCHAN(	UP	UP	NOCHAN(
NOCHAN(	NOCHAN(	NOCHAN(	NOCHAN(	NOCHAN(	NOCHAN(	NOCHAN(	DOWN	NOCHAN(
UP	NOCHAN(	NOCHAN(	NOCHAN(	UP	UP	NOCHAN(	DOWN	NOCHAN(
NOCHAN(	DOWN	DOWN	DOWN	NOCHAN(	DOWN	NOCHAN(	DOWN	DOWN
DOWN	DOWN	DOWN	DOWN	DOWN	NOCHAN(	DOWN	DOWN	DOWN
NOCHAN(	DOWN	DOWN	NOCHAN(	NOCHAN(	NOCHAN(	DOWN	DOWN	DOWN
NOCHAN(	NOCHAN(	DOWN	NOCHAN(	NOCHAN(	DOWN	NOCHAN(	NOCHAN(	NOCHAN(
UP	UP	UP	UP	UP	UP	UP	UP	UP
NOCHAN(	NOCHAN(	DOWN	NOCHAN(	NOCHAN(	NOCHAN(	DOWN	DOWN	DOWN
NOCHAN(	DOWN	NOCHAN(	DOWN	NOCHAN(	NOCHAN(	DOWN	DOWN	DOWN
NOCHAN(	NOCHAN(	NOCHAN(	NOCHAN(	NOCHAN(	NOCHAN(	DOWN	DOWN	NOCHAN(
NOCHAN(	DOWN	DOWN	DOWN	DOWN	DOWN	DOWN	DOWN	DOWN
NOCHAN(	NOCHAN(	DOWN	NOCHAN(	NOCHAN(	NOCHAN(	NOCHAN(	DOWN	NOCHAN(
NOCHAN(	NOCHAN(	NOCHAN(	NOCHAN(	NOCHAN(	DOWN	DOWN	DOWN	DOWN
NOCHAN(	NOCHAN(	NOCHAN(	DOWN	DOWN	NOCHAN(	NOCHAN(	DOWN	NOCHAN(
NOCHAN(	NOCHAN(	UP	NOCHAN(	NOCHAN(	NOCHAN(	UP	UP	UP
NOCHAN(	DOWN	DOWN	NOCHAN(	NOCHAN(	DOWN	DOWN	DOWN	DOWN
UP	NOCHAN(	NOCHAN(	NOCHAN(	NOCHAN(	NOCHAN(	NOCHAN(	DOWN	NOCHAN(
NOCHAN(	NOCHAN(	NOCHAN(	NOCHAN(	NOCHAN(	NOCHAN(	UP	UP	NOCHAN(
UP	UP	NOCHAN(	NOCHAN(	UP	DOWN	UP	DOWN	DOWN
NOCHAN(	NOCHAN(	DOWN	NOCHAN(	NOCHAN(	NOCHAN(	NOCHAN(	DOWN	NOCHAN(
DOWN	DOWN	DOWN	DOWN	NOCHAN(	DOWN	DOWN	DOWN	DOWN
UP	UP	DOWN	NOCHAN(	NOCHAN(	NOCHAN(	UP	NOCHAN(	NOCHAN(
NOCHAN(	UP	DOWN	UP	UP	NOCHAN(	UP	DOWN	DOWN
NOCHAN(	NOCHAN(	UP	NOCHAN(	UP	UP	DOWN	UP	UP

TCGA-DV	TCGA-BP-	TCGA-A3-	TCGA-B8-	TCGA-A3-	TCGA-B8-	TCGA-A3-	TCGA-B0-	TCGA-B2-
NOCHAN(	DOWN	NOCHAN(	NOCHAN(	DOWN	NOCHAN(	NOCHAN(	NOCHAN(	NOCHAN(
NOCHAN(	NOCHAN(	DOWN	DOWN	DOWN	NOCHAN(	DOWN	DOWN	NOCHAN(
NOCHAN(	DOWN	DOWN	DOWN	DOWN	DOWN	DOWN	DOWN	DOWN
NOCHAN(	NOCHAN(	UP	UP	NOCHAN(	UP	UP	UP	NOCHAN(
NOCHAN(	NOCHAN(	UP	UP	NOCHAN(	NOCHAN(	NOCHAN(	NOCHAN(	NOCHAN(
NOCHAN(	DOWN	DOWN	DOWN	DOWN	DOWN	DOWN	NOCHAN(	DOWN
DOWN	DOWN	NOCHAN(	NOCHAN(	NOCHAN(	DOWN	NOCHAN(	DOWN	NOCHAN(
NOCHAN(	NOCHAN(	NOCHAN(	NOCHAN(	NOCHAN(	NOCHAN(	NOCHAN(	NOCHAN(	NOCHAN(
NOCHAN(	UP	UP	NOCHAN(	NOCHAN(	UP	UP	UP	NOCHAN(
DOWN	DOWN	NOCHAN(	DOWN	NOCHAN(	DOWN	DOWN	NOCHAN(	DOWN
UP	NOCHAN(	NOCHAN(	NOCHAN(	UP	NOCHAN(	NOCHAN(	UP	NOCHAN(
NOCHAN(	NOCHAN(	DOWN	NOCHAN(	DOWN	NOCHAN(	NOCHAN(	NOCHAN(	NOCHAN(
NOCHAN(	NOCHAN(	NOCHAN(	NOCHAN(	NOCHAN(	NOCHAN(	NOCHAN(	NOCHAN(	NOCHAN(
NOCHAN(	UP	NOCHAN(	NOCHAN(	NOCHAN(	NOCHAN(	NOCHAN(	NOCHAN(	NOCHAN(
NOCHAN(	NOCHAN(	NOCHAN(	NOCHAN(	DOWN	UP	NOCHAN(	UP	NOCHAN(
NOCHAN(	NOCHAN(	NOCHAN(	NOCHAN(	NOCHAN(	NOCHAN(	NOCHAN(	UP	NOCHAN(
NOCHAN(	NOCHAN(	UP	UP	UP	NOCHAN(	UP	NOCHAN(	UP
UP	NOCHAN(	UP	UP	UP	UP	UP	NOCHAN(	NOCHAN(
DOWN	DOWN	DOWN	NOCHAN(	DOWN	DOWN	DOWN	DOWN	DOWN
DOWN	NOCHAN(	NOCHAN(	NOCHAN(	NOCHAN(	DOWN	NOCHAN(	DOWN	NOCHAN(
DOWN	DOWN	DOWN	DOWN	NOCHAN(	NOCHAN(	NOCHAN(	DOWN	DOWN
NOCHAN(	NOCHAN(	NOCHAN(	NOCHAN(	DOWN	NOCHAN(	NOCHAN(	NOCHAN(	DOWN
UP	UP	NOCHAN(	UP	UP	UP	UP	UP	UP
NOCHAN(	DOWN	NOCHAN(	DOWN	NOCHAN(	NOCHAN(	NOCHAN(	NOCHAN(	NOCHAN(
NOCHAN(	NOCHAN(	NOCHAN(	NOCHAN(	NOCHAN(	NOCHAN(	NOCHAN(	NOCHAN(	NOCHAN(
NOCHAN(	NOCHAN(	NOCHAN(	NOCHAN(	NOCHAN(	NOCHAN(	NOCHAN(	NOCHAN(	NOCHAN(
NOCHAN(	DOWN	NOCHAN(	DOWN	DOWN	NOCHAN(	NOCHAN(	DOWN	NOCHAN(
NOCHAN(	NOCHAN(	NOCHAN(	NOCHAN(	NOCHAN(	NOCHAN(	NOCHAN(	NOCHAN(	NOCHAN(
NOCHAN(	NOCHAN(	NOCHAN(	NOCHAN(	UP	NOCHAN(	NOCHAN(	UP	NOCHAN(
NOCHAN(	NOCHAN(	DOWN	NOCHAN(	DOWN	NOCHAN(	DOWN	NOCHAN(	DOWN
NOCHAN(	NOCHAN(	NOCHAN(	NOCHAN(	NOCHAN(	NOCHAN(	NOCHAN(	NOCHAN(	NOCHAN(
NOCHAN(	DOWN	NOCHAN(	NOCHAN(	NOCHAN(	NOCHAN(	NOCHAN(	NOCHAN(	NOCHAN(
NOCHAN(	NOCHAN(	UP	NOCHAN(	NOCHAN(	NOCHAN(	UP	UP	UP
NOCHAN(	NOCHAN(	NOCHAN(	NOCHAN(	NOCHAN(	NOCHAN(	NOCHAN(	NOCHAN(	NOCHAN(
NOCHAN(	UP	NOCHAN(	NOCHAN(	DOWN	NOCHAN(	NOCHAN(	NOCHAN(	NOCHAN(
DOWN	DOWN	NOCHAN(	NOCHAN(	NOCHAN(	NOCHAN(	NOCHAN(	NOCHAN(	NOCHAN(
DOWN	DOWN	NOCHAN(	DOWN	NOCHAN(	DOWN	DOWN	DOWN	DOWN
UP	NOCHAN(	NOCHAN(	UP	NOCHAN(	NOCHAN(	NOCHAN(	UP	NOCHAN(
NOCHAN(	NOCHAN(	UP	DOWN	UP	NOCHAN(	NOCHAN(	UP	UP
UP	NOCHAN(	UP	NOCHAN(	UP	UP	UP	NOCHAN(	UP

TCGA-A3-	TCGA-BP-	TCGA-BP-	TCGA-B8-	TCGA-B0-	TCGA-BP-	TCGA-DV	TCGA-BP-	TCGA-A3-
NOCHANG	NOCHANG	NOCHANG	DOWN	NOCHANG	DOWN	NOCHANG	UP	NOCHANG
NOCHANG	NOCHANG	NOCHANG	DOWN	NOCHANG	NOCHANG	NOCHANG	DOWN	DOWN
DOWN	DOWN	DOWN	DOWN	DOWN	DOWN	NOCHANG	NOCHANG	DOWN
NOCHANG	NOCHANG	NOCHANG	UP	NOCHANG	NOCHANG	UP	NOCHANG	NOCHANG
NOCHANG	NOCHANG	NOCHANG	UP	DOWN	NOCHANG	NOCHANG	NOCHANG	NOCHANG
NOCHANG	NOCHANG	DOWN	DOWN	DOWN	DOWN	NOCHANG	NOCHANG	DOWN
NOCHANG	NOCHANG	NOCHANG	UP	DOWN	DOWN	DOWN	DOWN	NOCHANG
NOCHANG	NOCHANG	NOCHANG	UP	NOCHANG	NOCHANG	NOCHANG	NOCHANG	NOCHANG
NOCHANG	NOCHANG	UP	UP	UP	UP	NOCHANG	UP	NOCHANG
NOCHANG	NOCHANG	NOCHANG	DOWN	NOCHANG	NOCHANG	DOWN	NOCHANG	NOCHANG
UP	NOCHANG	UP	UP	NOCHANG	NOCHANG	NOCHANG	NOCHANG	NOCHANG
NOCHANG	DOWN	NOCHANG	NOCHANG	NOCHANG	NOCHANG	DOWN	NOCHANG	NOCHANG
NOCHANG	NOCHANG	NOCHANG	NOCHANG	NOCHANG	NOCHANG	DOWN	NOCHANG	NOCHANG
NOCHANG	NOCHANG	NOCHANG	NOCHANG	UP	NOCHANG	NOCHANG	UP	NOCHANG
NOCHANG	DOWN	NOCHANG	NOCHANG	NOCHANG	NOCHANG	NOCHANG	NOCHANG	NOCHANG
NOCHANG	NOCHANG	NOCHANG	UP	NOCHANG	NOCHANG	NOCHANG	UP	UP
UP	UP	NOCHANG	UP	NOCHANG	NOCHANG	UP	NOCHANG	UP
UP	UP	UP	UP	NOCHANG	NOCHANG	NOCHANG	NOCHANG	NOCHANG
NOCHANG	DOWN	DOWN	DOWN	NOCHANG	NOCHANG	NOCHANG	NOCHANG	NOCHANG
NOCHANG	DOWN	NOCHANG	NOCHANG	DOWN	DOWN	NOCHANG	DOWN	DOWN
NOCHANG	NOCHANG	NOCHANG	DOWN	DOWN	DOWN	NOCHANG	DOWN	NOCHANG
NOCHANG	DOWN	NOCHANG	NOCHANG	NOCHANG	NOCHANG	NOCHANG	NOCHANG	NOCHANG
UP	NOCHANG	UP	UP	UP	UP	NOCHANG	NOCHANG	UP
NOCHANG	NOCHANG	NOCHANG	NOCHANG	NOCHANG	DOWN	NOCHANG	NOCHANG	NOCHANG
NOCHANG	NOCHANG	NOCHANG	NOCHANG	NOCHANG	NOCHANG	NOCHANG	NOCHANG	NOCHANG
NOCHANG	NOCHANG	NOCHANG	NOCHANG	NOCHANG	NOCHANG	NOCHANG	NOCHANG	NOCHANG
DOWN	DOWN	DOWN	DOWN	DOWN	NOCHANG	NOCHANG	DOWN	DOWN
NOCHANG	NOCHANG	NOCHANG	NOCHANG	NOCHANG	NOCHANG	NOCHANG	UP	NOCHANG
NOCHANG	NOCHANG	NOCHANG	NOCHANG	NOCHANG	NOCHANG	UP	NOCHANG	NOCHANG
NOCHANG	DOWN	NOCHANG	NOCHANG	NOCHANG	NOCHANG	NOCHANG	NOCHANG	NOCHANG
DOWN	DOWN	NOCHANG	NOCHANG	UP	NOCHANG	DOWN	UP	NOCHANG
NOCHANG	NOCHANG	NOCHANG	DOWN	NOCHANG	NOCHANG	NOCHANG	NOCHANG	NOCHANG
UP	UP	UP	UP	NOCHANG	NOCHANG	UP	NOCHANG	NOCHANG
DOWN	NOCHANG	NOCHANG	NOCHANG	NOCHANG	NOCHANG	NOCHANG	NOCHANG	NOCHANG
NOCHANG	NOCHANG	NOCHANG	NOCHANG	NOCHANG	NOCHANG	NOCHANG	NOCHANG	NOCHANG
NOCHANG	NOCHANG	NOCHANG	NOCHANG	NOCHANG	NOCHANG	DOWN	NOCHANG	UP
NOCHANG	DOWN	DOWN	DOWN	NOCHANG	DOWN	NOCHANG	NOCHANG	DOWN
UP	NOCHANG	UP	UP	UP	UP	NOCHANG	NOCHANG	NOCHANG
UP	UP	NOCHANG	NOCHANG	NOCHANG	NOCHANG	UP	UP	UP
UP	UP	UP	UP	NOCHANG	NOCHANG	NOCHANG	NOCHANG	NOCHANG

TCGA-CZ-	TCGA-BP-	TCGA-BP-	TCGA-BP-	TCGA-AK-	TCGA-A3-	TCGA-BP-	TCGA-B2-	TCGA-CJ-
DOWN	DOWN	NOCHAN(	NOCHAN(	DOWN	DOWN	DOWN	NOCHAN(	DOWN
DOWN	NOCHAN(	NOCHAN(	DOWN	NOCHAN(	DOWN	DOWN	NOCHAN(	NOCHAN(
DOWN	NOCHAN(	DOWN	DOWN	DOWN	DOWN	DOWN	NOCHAN(	NOCHAN(
NOCHAN(	NOCHAN(	NOCHAN(	UP	NOCHAN(	UP	NOCHAN(	NOCHAN(	NOCHAN(
NOCHAN(	NOCHAN(	NOCHAN(	UP	NOCHAN(	DOWN	DOWN	UP	NOCHAN(
DOWN	DOWN	DOWN	DOWN	NOCHAN(	DOWN	DOWN	NOCHAN(	NOCHAN(
DOWN	DOWN	NOCHAN(	DOWN	DOWN	DOWN	DOWN	NOCHAN(	DOWN
NOCHAN(	NOCHAN(	NOCHAN(	NOCHAN(	NOCHAN(	NOCHAN(	DOWN	NOCHAN(	NOCHAN(
UP	NOCHAN(	NOCHAN(	UP	NOCHAN(	UP	NOCHAN(	DOWN	UP
DOWN	NOCHAN(	NOCHAN(	DOWN	NOCHAN(	DOWN	DOWN	UP	NOCHAN(
NOCHAN(	NOCHAN(	UP	NOCHAN(	UP	NOCHAN(	NOCHAN(	UP	NOCHAN(
NOCHAN(	NOCHAN(	NOCHAN(	NOCHAN(	NOCHAN(	UP	NOCHAN(	DOWN	NOCHAN(
NOCHAN(	DOWN	NOCHAN(	NOCHAN(	NOCHAN(	NOCHAN(	NOCHAN(	NOCHAN(	NOCHAN(
UP	NOCHAN(	NOCHAN(	NOCHAN(	NOCHAN(	UP	NOCHAN(	DOWN	NOCHAN(
NOCHAN(	NOCHAN(	NOCHAN(	NOCHAN(	NOCHAN(	NOCHAN(	DOWN	DOWN	NOCHAN(
UP	NOCHAN(	NOCHAN(	NOCHAN(	NOCHAN(	UP	UP	DOWN	NOCHAN(
NOCHAN(	NOCHAN(	UP	UP	NOCHAN(	NOCHAN(	NOCHAN(	UP	NOCHAN(
UP	UP	UP	UP	UP	NOCHAN(	NOCHAN(	UP	UP
DOWN	DOWN	DOWN	DOWN	NOCHAN(	DOWN	NOCHAN(	NOCHAN(	DOWN
DOWN	DOWN	NOCHAN(	DOWN	DOWN	NOCHAN(	DOWN	NOCHAN(	NOCHAN(
DOWN	DOWN	NOCHAN(	DOWN	NOCHAN(	DOWN	DOWN	UP	NOCHAN(
NOCHAN(	NOCHAN(	NOCHAN(	NOCHAN(	NOCHAN(	NOCHAN(	NOCHAN(	DOWN	DOWN
UP	NOCHAN(	UP	UP	UP	UP	UP	DOWN	NOCHAN(
DOWN	NOCHAN(	NOCHAN(	NOCHAN(	NOCHAN(	NOCHAN(	DOWN	NOCHAN(	NOCHAN(
DOWN	NOCHAN(	NOCHAN(	NOCHAN(	NOCHAN(	DOWN	DOWN	UP	NOCHAN(
NOCHAN(	NOCHAN(	NOCHAN(	NOCHAN(	NOCHAN(	NOCHAN(	DOWN	NOCHAN(	NOCHAN(
DOWN	NOCHAN(	DOWN	DOWN	NOCHAN(	DOWN	DOWN	NOCHAN(	NOCHAN(
NOCHAN(	NOCHAN(	NOCHAN(	NOCHAN(	NOCHAN(	NOCHAN(	NOCHAN(	DOWN	NOCHAN(
DOWN	NOCHAN(	UP	UP	NOCHAN(	NOCHAN(	NOCHAN(	DOWN	UP
NOCHAN(	DOWN	DOWN	NOCHAN(	NOCHAN(	DOWN	NOCHAN(	DOWN	NOCHAN(
UP	DOWN	NOCHAN(	NOCHAN(	NOCHAN(	UP	NOCHAN(	DOWN	NOCHAN(
DOWN	NOCHAN(	NOCHAN(	NOCHAN(	NOCHAN(	DOWN	NOCHAN(	UP	NOCHAN(
NOCHAN(	NOCHAN(	UP	NOCHAN(	UP	NOCHAN(	NOCHAN(	UP	NOCHAN(
UP	NOCHAN(	NOCHAN(	NOCHAN(	NOCHAN(	UP	UP	DOWN	NOCHAN(
NOCHAN(	NOCHAN(	NOCHAN(	NOCHAN(	UP	UP	NOCHAN(	DOWN	NOCHAN(
DOWN	NOCHAN(	NOCHAN(	DOWN	NOCHAN(	NOCHAN(	NOCHAN(	NOCHAN(	NOCHAN(
DOWN	DOWN	DOWN	DOWN	NOCHAN(	DOWN	DOWN	NOCHAN(	DOWN
NOCHAN(	NOCHAN(	NOCHAN(	NOCHAN(	UP	UP	UP	UP	NOCHAN(
NOCHAN(	UP	UP	NOCHAN(	NOCHAN(	NOCHAN(	NOCHAN(	NOCHAN(	NOCHAN(
NOCHAN(	NOCHAN(	UP	UP	UP	NOCHAN(	DOWN	NOCHAN(	UP

TCGA-BP-	TCGA-CJ-	TCGA-BP-	TCGA-BP-	TCGA-CZ-	TCGA-B0-	TCGA-A3-	TCGA-CJ-	TCGA-BP-
NOCHANG	DOWN	NOCHANG	NOCHANG	DOWN	NOCHANG	NOCHANG	DOWN	NOCHANG
DOWN	DOWN	NOCHANG	NOCHANG	NOCHANG	NOCHANG	NOCHANG	DOWN	DOWN
DOWN	DOWN	DOWN	DOWN	DOWN	DOWN	DOWN	DOWN	DOWN
NOCHANG	UP	UP	NOCHANG	NOCHANG	NOCHANG	NOCHANG	NOCHANG	NOCHANG
NOCHANG	DOWN	UP	NOCHANG	NOCHANG	NOCHANG	UP	NOCHANG	NOCHANG
DOWN	DOWN	NOCHANG	DOWN	DOWN	DOWN	NOCHANG	DOWN	DOWN
NOCHANG	DOWN	DOWN	NOCHANG	DOWN	NOCHANG	NOCHANG	UP	NOCHANG
NOCHANG	NOCHANG	NOCHANG	NOCHANG	NOCHANG	NOCHANG	NOCHANG	NOCHANG	NOCHANG
UP	UP	UP	UP	UP	UP	NOCHANG	NOCHANG	UP
DOWN	DOWN	NOCHANG	NOCHANG	NOCHANG	NOCHANG	NOCHANG	NOCHANG	NOCHANG
NOCHANG	NOCHANG	NOCHANG	UP	NOCHANG	UP	UP	UP	UP
UP	NOCHANG	NOCHANG	NOCHANG	NOCHANG	NOCHANG	NOCHANG	NOCHANG	NOCHANG
NOCHANG	NOCHANG	NOCHANG	NOCHANG	NOCHANG	NOCHANG	NOCHANG	NOCHANG	NOCHANG
UP	UP	NOCHANG	NOCHANG	NOCHANG	NOCHANG	NOCHANG	NOCHANG	UP
NOCHANG	NOCHANG	NOCHANG	NOCHANG	NOCHANG	NOCHANG	NOCHANG	DOWN	NOCHANG
NOCHANG	NOCHANG	NOCHANG	NOCHANG	NOCHANG	NOCHANG	NOCHANG	NOCHANG	NOCHANG
NOCHANG	DOWN	UP	NOCHANG	NOCHANG	UP	UP	NOCHANG	NOCHANG
NOCHANG	UP	NOCHANG	UP	NOCHANG	NOCHANG	UP	NOCHANG	NOCHANG
DOWN	DOWN	DOWN	NOCHANG	DOWN	NOCHANG	DOWN	DOWN	DOWN
DOWN	DOWN	NOCHANG	NOCHANG	DOWN	NOCHANG	DOWN	NOCHANG	DOWN
DOWN	DOWN	DOWN	NOCHANG	NOCHANG	NOCHANG	NOCHANG	DOWN	DOWN
NOCHANG	NOCHANG	NOCHANG	NOCHANG	NOCHANG	NOCHANG	DOWN	DOWN	NOCHANG
UP	UP	NOCHANG	UP	UP	UP	UP	UP	UP
DOWN	NOCHANG	NOCHANG	NOCHANG	NOCHANG	NOCHANG	NOCHANG	NOCHANG	NOCHANG
DOWN	NOCHANG	NOCHANG	NOCHANG	NOCHANG	NOCHANG	NOCHANG	NOCHANG	NOCHANG
NOCHANG	NOCHANG	NOCHANG	NOCHANG	NOCHANG	NOCHANG	NOCHANG	NOCHANG	NOCHANG
DOWN	DOWN	NOCHANG	DOWN	DOWN	DOWN	NOCHANG	DOWN	NOCHANG
NOCHANG	NOCHANG	NOCHANG	NOCHANG	UP	NOCHANG	NOCHANG	DOWN	NOCHANG
NOCHANG	NOCHANG	NOCHANG	UP	NOCHANG	NOCHANG	UP	NOCHANG	NOCHANG
NOCHANG	NOCHANG	NOCHANG	DOWN	DOWN	NOCHANG	DOWN	NOCHANG	NOCHANG
UP	UP	NOCHANG	NOCHANG	NOCHANG	NOCHANG	DOWN	NOCHANG	NOCHANG
DOWN	NOCHANG	NOCHANG	NOCHANG	DOWN	NOCHANG	NOCHANG	NOCHANG	DOWN
NOCHANG	NOCHANG	NOCHANG	NOCHANG	NOCHANG	UP	UP	NOCHANG	NOCHANG
UP	NOCHANG	DOWN	NOCHANG	NOCHANG	NOCHANG	NOCHANG	NOCHANG	NOCHANG
NOCHANG	NOCHANG	NOCHANG	UP	NOCHANG	UP	NOCHANG	DOWN	DOWN
NOCHANG	NOCHANG	NOCHANG	NOCHANG	NOCHANG	NOCHANG	NOCHANG	NOCHANG	NOCHANG
DOWN	DOWN	NOCHANG	DOWN	DOWN	DOWN	DOWN	DOWN	DOWN
UP	UP	NOCHANG	UP	NOCHANG	UP	NOCHANG	NOCHANG	UP
NOCHANG	NOCHANG	NOCHANG	UP	UP	NOCHANG	UP	UP	NOCHANG
NOCHANG	NOCHANG	UP	UP	DOWN	UP	UP	UP	UP

TCGA-A3-	TCGA-B8-	TCGA-B8-	TCGA-CZ-	TCGA-GK-	TCGA-BP-	TCGA-BP-	TCGA-B2-	TCGA-CZ-
DOWN	NOCHAN(	DOWN	DOWN	DOWN	DOWN	DOWN	DOWN	DOWN
DOWN	DOWN	NOCHAN(	NOCHAN(	DOWN	DOWN	DOWN	NOCHAN(	NOCHAN(
DOWN	NOCHAN(	NOCHAN(	NOCHAN(	DOWN	DOWN	DOWN	NOCHAN(	NOCHAN(
UP	NOCHAN(	NOCHAN(	NOCHAN(	UP	UP	NOCHAN(	NOCHAN(	NOCHAN(
NOCHAN(	NOCHAN(	NOCHAN(	NOCHAN(	NOCHAN(	UP	NOCHAN(	NOCHAN(	NOCHAN(
DOWN	DOWN	NOCHAN(	DOWN	DOWN	DOWN	DOWN	DOWN	NOCHAN(
NOCHAN(	NOCHAN(	DOWN	DOWN	DOWN	NOCHAN(	NOCHAN(	DOWN	NOCHAN(
NOCHAN(	UP	NOCHAN(	NOCHAN(	UP	NOCHAN(	NOCHAN(	NOCHAN(	NOCHAN(
UP	NOCHAN(	DOWN	NOCHAN(	UP	UP	NOCHAN(	UP	NOCHAN(
NOCHAN(	NOCHAN(	DOWN	NOCHAN(	DOWN	DOWN	NOCHAN(	NOCHAN(	NOCHAN(
NOCHAN(	NOCHAN(	NOCHAN(	NOCHAN(	NOCHAN(	NOCHAN(	NOCHAN(	NOCHAN(	NOCHAN(
NOCHAN(	NOCHAN(	DOWN	NOCHAN(	NOCHAN(	NOCHAN(	DOWN	NOCHAN(	NOCHAN(
NOCHAN(	NOCHAN(	NOCHAN(	NOCHAN(	NOCHAN(	NOCHAN(	NOCHAN(	NOCHAN(	NOCHAN(
UP	NOCHAN(	DOWN	UP	NOCHAN(	NOCHAN(	UP	UP	NOCHAN(
NOCHAN(	NOCHAN(	NOCHAN(	NOCHAN(	NOCHAN(	NOCHAN(	DOWN	UP	NOCHAN(
UP	NOCHAN(	NOCHAN(	NOCHAN(	NOCHAN(	NOCHAN(	NOCHAN(	NOCHAN(	NOCHAN(
NOCHAN(	NOCHAN(	DOWN	DOWN	NOCHAN(	NOCHAN(	NOCHAN(	NOCHAN(	NOCHAN(
NOCHAN(	NOCHAN(	NOCHAN(	NOCHAN(	UP	NOCHAN(	NOCHAN(	NOCHAN(	NOCHAN(
NOCHAN(	DOWN	DOWN	DOWN	DOWN	DOWN	DOWN	NOCHAN(	NOCHAN(
NOCHAN(	DOWN	DOWN	NOCHAN(	DOWN	DOWN	DOWN	DOWN	NOCHAN(
DOWN	DOWN	DOWN	DOWN	DOWN	DOWN	DOWN	DOWN	DOWN
NOCHAN(	NOCHAN(	DOWN	NOCHAN(	NOCHAN(	NOCHAN(	DOWN	NOCHAN(	NOCHAN(
UP	UP	NOCHAN(	UP	UP	UP	UP	UP	UP
NOCHAN(	NOCHAN(	DOWN	NOCHAN(	NOCHAN(	DOWN	NOCHAN(	DOWN	NOCHAN(
NOCHAN(	NOCHAN(	NOCHAN(	NOCHAN(	NOCHAN(	NOCHAN(	NOCHAN(	NOCHAN(	NOCHAN(
NOCHAN(	NOCHAN(	DOWN	NOCHAN(	NOCHAN(	NOCHAN(	DOWN	NOCHAN(	DOWN
DOWN	DOWN	NOCHAN(	DOWN	DOWN	DOWN	NOCHAN(	NOCHAN(	NOCHAN(
NOCHAN(	NOCHAN(	NOCHAN(	NOCHAN(	NOCHAN(	NOCHAN(	NOCHAN(	NOCHAN(	NOCHAN(
NOCHAN(	NOCHAN(	NOCHAN(	NOCHAN(	UP	NOCHAN(	NOCHAN(	NOCHAN(	NOCHAN(
NOCHAN(	DOWN	NOCHAN(	NOCHAN(	NOCHAN(	NOCHAN(	NOCHAN(	NOCHAN(	NOCHAN(
UP	NOCHAN(	NOCHAN(	NOCHAN(	NOCHAN(	UP	DOWN	UP	NOCHAN(
DOWN	NOCHAN(	NOCHAN(	NOCHAN(	NOCHAN(	DOWN	NOCHAN(	NOCHAN(	NOCHAN(
NOCHAN(	NOCHAN(	UP	UP	NOCHAN(	NOCHAN(	NOCHAN(	NOCHAN(	UP
UP	DOWN	DOWN	NOCHAN(	NOCHAN(	NOCHAN(	NOCHAN(	NOCHAN(	NOCHAN(
NOCHAN(	NOCHAN(	UP	NOCHAN(	UP	NOCHAN(	DOWN	NOCHAN(	NOCHAN(
NOCHAN(	NOCHAN(	DOWN	NOCHAN(	NOCHAN(	NOCHAN(	NOCHAN(	NOCHAN(	NOCHAN(
DOWN	NOCHAN(	DOWN	NOCHAN(	NOCHAN(	DOWN	NOCHAN(	NOCHAN(	DOWN
UP	UP	NOCHAN(	UP	NOCHAN(	NOCHAN(	UP	UP	UP
NOCHAN(	UP	NOCHAN(	UP	UP	NOCHAN(	UP	NOCHAN(	UP
UP	NOCHAN(	NOCHAN(	NOCHAN(	UP	UP	UP	NOCHAN(	NOCHAN(

TCGA-CJ-	TCGA-B0-	TCGA-BP-	TCGA-B0-	TCGA-B8-	TCGA-B2-	TCGA-A3-	TCGA-CZ-	TCGA-CJ-
NOCHAN(	DOWN	DOWN	DOWN	DOWN	DOWN	DOWN	DOWN	DOWN
NOCHAN(	DOWN	DOWN	NOCHAN(	DOWN	NOCHAN(	DOWN	NOCHAN(	NOCHAN(
DOWN	DOWN	DOWN	DOWN	DOWN	NOCHAN(	DOWN	DOWN	DOWN
NOCHAN(	UP	NOCHAN(	UP	UP	UP	NOCHAN(	NOCHAN(	NOCHAN(
NOCHAN(	NOCHAN(	DOWN	NOCHAN(	NOCHAN(	NOCHAN(	NOCHAN(	NOCHAN(	NOCHAN(
DOWN	DOWN	DOWN	DOWN	NOCHAN(	NOCHAN(	DOWN	DOWN	NOCHAN(
NOCHAN(	DOWN	NOCHAN(	NOCHAN(	DOWN	NOCHAN(	NOCHAN(	DOWN	NOCHAN(
NOCHAN(	UP	NOCHAN(	UP	NOCHAN(	UP	NOCHAN(	NOCHAN(	NOCHAN(
NOCHAN(	UP	UP	UP	UP	NOCHAN(	NOCHAN(	NOCHAN(	UP
NOCHAN(	DOWN	DOWN	DOWN	DOWN	NOCHAN(	NOCHAN(	DOWN	NOCHAN(
NOCHAN(	UP	NOCHAN(	NOCHAN(	UP	NOCHAN(	NOCHAN(	NOCHAN(	UP
NOCHAN(	UP	NOCHAN(	NOCHAN(	NOCHAN(	DOWN	DOWN	NOCHAN(	NOCHAN(
NOCHAN(	NOCHAN(	NOCHAN(	DOWN	NOCHAN(	DOWN	NOCHAN(	NOCHAN(	NOCHAN(
NOCHAN(	UP	NOCHAN(	UP	NOCHAN(	NOCHAN(	NOCHAN(	NOCHAN(	NOCHAN(
NOCHAN(	NOCHAN(	DOWN	DOWN	UP	NOCHAN(	DOWN	NOCHAN(	NOCHAN(
UP	UP	UP	NOCHAN(	UP	NOCHAN(	NOCHAN(	NOCHAN(	NOCHAN(
NOCHAN(	NOCHAN(	NOCHAN(	DOWN	NOCHAN(	NOCHAN(	NOCHAN(	NOCHAN(	UP
NOCHAN(	NOCHAN(	NOCHAN(	UP	NOCHAN(	UP	NOCHAN(	NOCHAN(	UP
NOCHAN(	DOWN	DOWN	DOWN	DOWN	NOCHAN(	DOWN	DOWN	NOCHAN(
DOWN	DOWN	DOWN	DOWN	NOCHAN(	NOCHAN(	UP	NOCHAN(	NOCHAN(
DOWN	DOWN	DOWN	DOWN	DOWN	NOCHAN(	DOWN	DOWN	NOCHAN(
NOCHAN(	NOCHAN(	NOCHAN(	NOCHAN(	NOCHAN(	DOWN	NOCHAN(	NOCHAN(	NOCHAN(
UP	UP	UP	UP	UP	UP	UP	UP	UP
NOCHAN(	DOWN	DOWN	NOCHAN(	DOWN	NOCHAN(	NOCHAN(	DOWN	NOCHAN(
NOCHAN(	DOWN	DOWN	NOCHAN(	NOCHAN(	NOCHAN(	NOCHAN(	NOCHAN(	NOCHAN(
NOCHAN(	NOCHAN(	DOWN	NOCHAN(	NOCHAN(	NOCHAN(	NOCHAN(	NOCHAN(	NOCHAN(
DOWN	DOWN	DOWN	DOWN	DOWN	NOCHAN(	NOCHAN(	DOWN	NOCHAN(
NOCHAN(	UP	NOCHAN(	NOCHAN(	NOCHAN(	DOWN	NOCHAN(	NOCHAN(	NOCHAN(
NOCHAN(	NOCHAN(	NOCHAN(	NOCHAN(	UP	UP	NOCHAN(	NOCHAN(	NOCHAN(
NOCHAN(	NOCHAN(	NOCHAN(	NOCHAN(	NOCHAN(	NOCHAN(	NOCHAN(	NOCHAN(	NOCHAN(
DOWN	UP	NOCHAN(	UP	NOCHAN(	DOWN	NOCHAN(	NOCHAN(	NOCHAN(
NOCHAN(	DOWN	DOWN	NOCHAN(	NOCHAN(	NOCHAN(	NOCHAN(	NOCHAN(	NOCHAN(
NOCHAN(	NOCHAN(	NOCHAN(	NOCHAN(	NOCHAN(	UP	UP	NOCHAN(	UP
NOCHAN(	NOCHAN(	UP	NOCHAN(	NOCHAN(	NOCHAN(	NOCHAN(	NOCHAN(	NOCHAN(
UP	DOWN	NOCHAN(	NOCHAN(	UP	NOCHAN(	DOWN	NOCHAN(	UP
NOCHAN(	DOWN	NOCHAN(	DOWN	NOCHAN(	DOWN	NOCHAN(	DOWN	NOCHAN(
NOCHAN(	DOWN	DOWN	DOWN	DOWN	NOCHAN(	DOWN	DOWN	DOWN
NOCHAN(	NOCHAN(	NOCHAN(	NOCHAN(	NOCHAN(	NOCHAN(	NOCHAN(	NOCHAN(	UP
UP	NOCHAN(	NOCHAN(	NOCHAN(	NOCHAN(	UP	UP	NOCHAN(	NOCHAN(
NOCHAN(	NOCHAN(	NOCHAN(	NOCHAN(	UP	UP	UP	NOCHAN(	UP

TCGA-CJ-	TCGA-BP-	TCGA-CJ-	TCGA-CJ-	TCGA-CJ-	TCGA-CW	TCGA-A3-	TCGA-CJ-	TCGA-CJ-
DOWN	DOWN	DOWN	DOWN	DOWN	DOWN	NOCHAN(	DOWN	DOWN
NOCHAN(	NOCHAN(	DOWN	DOWN	NOCHAN(	NOCHAN(	NOCHAN(	NOCHAN(	NOCHAN(
NOCHAN(	DOWN	DOWN	DOWN	DOWN	DOWN	DOWN	DOWN	NOCHAN(
NOCHAN(	UP	UP	UP	UP	NOCHAN(	UP	NOCHAN(	NOCHAN(
NOCHAN(	NOCHAN(	UP	UP	NOCHAN(	DOWN	NOCHAN(	DOWN	NOCHAN(
NOCHAN(	DOWN	DOWN	DOWN	NOCHAN(	DOWN	DOWN	NOCHAN(	NOCHAN(
NOCHAN(	NOCHAN(	DOWN	DOWN	NOCHAN(	NOCHAN(	NOCHAN(	DOWN	DOWN
UP	NOCHAN(	NOCHAN(	NOCHAN(	NOCHAN(	NOCHAN(	NOCHAN(	NOCHAN(	NOCHAN(
NOCHAN(	UP	UP	UP	UP	UP	UP	NOCHAN(	UP
NOCHAN(	DOWN	DOWN	DOWN	DOWN	DOWN	DOWN	NOCHAN(	DOWN
NOCHAN(	NOCHAN(	NOCHAN(	UP	NOCHAN(	NOCHAN(	NOCHAN(	NOCHAN(	NOCHAN(
DOWN	NOCHAN(	DOWN	NOCHAN(	NOCHAN(	NOCHAN(	NOCHAN(	NOCHAN(	NOCHAN(
NOCHAN(	NOCHAN(	NOCHAN(	NOCHAN(	NOCHAN(	NOCHAN(	NOCHAN(	NOCHAN(	NOCHAN(
NOCHAN(	NOCHAN(	UP	UP	NOCHAN(	UP	UP	UP	NOCHAN(
NOCHAN(	DOWN	DOWN	DOWN	NOCHAN(	NOCHAN(	NOCHAN(	NOCHAN(	NOCHAN(
NOCHAN(	NOCHAN(	NOCHAN(	NOCHAN(	UP	UP	NOCHAN(	NOCHAN(	UP
NOCHAN(	NOCHAN(	NOCHAN(	NOCHAN(	UP	DOWN	NOCHAN(	DOWN	NOCHAN(
UP	NOCHAN(	NOCHAN(	NOCHAN(	UP	NOCHAN(	UP	NOCHAN(	NOCHAN(
NOCHAN(	DOWN	DOWN	DOWN	DOWN	DOWN	DOWN	DOWN	NOCHAN(
NOCHAN(	DOWN	DOWN	DOWN	NOCHAN(	DOWN	NOCHAN(	NOCHAN(	DOWN
NOCHAN(	DOWN	DOWN	DOWN	DOWN	DOWN	NOCHAN(	DOWN	DOWN
DOWN	NOCHAN(	NOCHAN(	NOCHAN(	NOCHAN(	DOWN	NOCHAN(	NOCHAN(	NOCHAN(
UP	UP	UP	UP	NOCHAN(	UP	NOCHAN(	UP	UP
NOCHAN(	NOCHAN(	DOWN	DOWN	NOCHAN(	DOWN	NOCHAN(	DOWN	NOCHAN(
NOCHAN(	NOCHAN(	NOCHAN(	NOCHAN(	NOCHAN(	NOCHAN(	NOCHAN(	DOWN	NOCHAN(
NOCHAN(	NOCHAN(	NOCHAN(	NOCHAN(	NOCHAN(	NOCHAN(	NOCHAN(	NOCHAN(	NOCHAN(
NOCHAN(	DOWN	DOWN	DOWN	DOWN	DOWN	DOWN	DOWN	DOWN
NOCHAN(	NOCHAN(	DOWN	NOCHAN(	NOCHAN(	NOCHAN(	NOCHAN(	NOCHAN(	UP
NOCHAN(	NOCHAN(	NOCHAN(	NOCHAN(	NOCHAN(	NOCHAN(	UP	NOCHAN(	DOWN
NOCHAN(	NOCHAN(	DOWN	DOWN	DOWN	NOCHAN(	NOCHAN(	NOCHAN(	NOCHAN(
DOWN	NOCHAN(	NOCHAN(	UP	NOCHAN(	NOCHAN(	NOCHAN(	NOCHAN(	UP
NOCHAN(	DOWN	DOWN	DOWN	NOCHAN(	NOCHAN(	NOCHAN(	NOCHAN(	DOWN
UP	NOCHAN(	UP	NOCHAN(	NOCHAN(	NOCHAN(	UP	NOCHAN(	NOCHAN(
DOWN	NOCHAN(	UP	UP	NOCHAN(	NOCHAN(	NOCHAN(	NOCHAN(	NOCHAN(
NOCHAN(	NOCHAN(	DOWN	NOCHAN(	NOCHAN(	NOCHAN(	NOCHAN(	NOCHAN(	UP
NOCHAN(	DOWN	DOWN	NOCHAN(	NOCHAN(	NOCHAN(	NOCHAN(	NOCHAN(	NOCHAN(
DOWN	DOWN	DOWN	DOWN	DOWN	DOWN	DOWN	DOWN	DOWN
UP	NOCHAN(	NOCHAN(	NOCHAN(	NOCHAN(	NOCHAN(	NOCHAN(	NOCHAN(	UP
UP	NOCHAN(	NOCHAN(	NOCHAN(	UP	UP	UP	UP	NOCHAN(
NOCHAN(	UP	NOCHAN(	UP	UP	UP	UP	NOCHAN(	DOWN

TCGA-BP-	TCGA-B0-	TCGA-CZ-	TCGA-A3-	TCGA-CZ-	TCGA-B2-	TCGA-BP-	TCGA-B8-	TCGA-BP-
DOWN	NOCHAN(	NOCHAN(	UP	DOWN	NOCHAN(	NOCHAN(	NOCHAN(	DOWN
NOCHAN(	DOWN	DOWN	NOCHAN(	NOCHAN(	DOWN	NOCHAN(	NOCHAN(	DOWN
NOCHAN(	DOWN	DOWN	NOCHAN(	DOWN	NOCHAN(	DOWN	DOWN	DOWN
NOCHAN(	NOCHAN(	NOCHAN(	NOCHAN(	NOCHAN(	NOCHAN(	NOCHAN(	NOCHAN(	NOCHAN(
NOCHAN(	NOCHAN(	NOCHAN(	UP	NOCHAN(	UP	NOCHAN(	NOCHAN(	NOCHAN(
NOCHAN(	DOWN	NOCHAN(	NOCHAN(	DOWN	UP	DOWN	DOWN	DOWN
DOWN	NOCHAN(	DOWN	NOCHAN(	NOCHAN(	UP	DOWN	DOWN	NOCHAN(
NOCHAN(	NOCHAN(	NOCHAN(	NOCHAN(	UP	UP	NOCHAN(	UP	UP
NOCHAN(	UP	NOCHAN(	NOCHAN(	UP	DOWN	UP	UP	UP
NOCHAN(	NOCHAN(	NOCHAN(	DOWN	DOWN	UP	NOCHAN(	NOCHAN(	DOWN
NOCHAN(	NOCHAN(	NOCHAN(	UP	NOCHAN(	UP	NOCHAN(	NOCHAN(	NOCHAN(
NOCHAN(	DOWN	NOCHAN(	NOCHAN(	NOCHAN(	DOWN	NOCHAN(	NOCHAN(	UP
NOCHAN(	NOCHAN(	DOWN	NOCHAN(	NOCHAN(	NOCHAN(	NOCHAN(	NOCHAN(	DOWN
NOCHAN(	UP	NOCHAN(	UP	NOCHAN(	DOWN	NOCHAN(	NOCHAN(	UP
DOWN	NOCHAN(	NOCHAN(	DOWN	UP	DOWN	NOCHAN(	NOCHAN(	NOCHAN(
UP	NOCHAN(	UP	UP	NOCHAN(	DOWN	UP	NOCHAN(	NOCHAN(
NOCHAN(	NOCHAN(	NOCHAN(	NOCHAN(	NOCHAN(	UP	NOCHAN(	NOCHAN(	DOWN
NOCHAN(	NOCHAN(	NOCHAN(	NOCHAN(	NOCHAN(	UP	UP	NOCHAN(	UP
NOCHAN(	DOWN	DOWN	NOCHAN(	DOWN	DOWN	DOWN	DOWN	DOWN
DOWN	DOWN	NOCHAN(	UP	DOWN	NOCHAN(	NOCHAN(	DOWN	DOWN
NOCHAN(	DOWN	DOWN	NOCHAN(	DOWN	UP	NOCHAN(	DOWN	DOWN
NOCHAN(	NOCHAN(	NOCHAN(	NOCHAN(	NOCHAN(	DOWN	NOCHAN(	NOCHAN(	NOCHAN(
UP	UP	UP	UP	UP	NOCHAN(	UP	UP	UP
NOCHAN(	NOCHAN(	NOCHAN(	NOCHAN(	DOWN	NOCHAN(	NOCHAN(	DOWN	DOWN
NOCHAN(	NOCHAN(	DOWN	UP	NOCHAN(	UP	NOCHAN(	NOCHAN(	DOWN
NOCHAN(	NOCHAN(	NOCHAN(	NOCHAN(	DOWN	NOCHAN(	NOCHAN(	NOCHAN(	NOCHAN(
DOWN	DOWN	DOWN	DOWN	DOWN	NOCHAN(	DOWN	NOCHAN(	DOWN
NOCHAN(	NOCHAN(	NOCHAN(	UP	NOCHAN(	DOWN	NOCHAN(	NOCHAN(	NOCHAN(
UP	DOWN	NOCHAN(	NOCHAN(	NOCHAN(	NOCHAN(	NOCHAN(	NOCHAN(	DOWN
NOCHAN(	NOCHAN(	NOCHAN(	UP	UP	NOCHAN(	DOWN	NOCHAN(	NOCHAN(
NOCHAN(	NOCHAN(	NOCHAN(	NOCHAN(	UP	DOWN	NOCHAN(	NOCHAN(	UP
NOCHAN(	NOCHAN(	NOCHAN(	NOCHAN(	DOWN	UP	NOCHAN(	NOCHAN(	DOWN
NOCHAN(	NOCHAN(	UP	NOCHAN(	UP	UP	NOCHAN(	NOCHAN(	DOWN
NOCHAN(	NOCHAN(	NOCHAN(	UP	UP	DOWN	NOCHAN(	NOCHAN(	UP
NOCHAN(	DOWN	UP	NOCHAN(	NOCHAN(	DOWN	UP	NOCHAN(	NOCHAN(
NOCHAN(	DOWN	NOCHAN(	NOCHAN(	DOWN	UP	NOCHAN(	NOCHAN(	DOWN
NOCHAN(	DOWN	DOWN	NOCHAN(	DOWN	DOWN	DOWN	DOWN	DOWN
NOCHAN(	NOCHAN(	NOCHAN(	UP	UP	NOCHAN(	NOCHAN(	NOCHAN(	UP
UP	UP	UP	NOCHAN(	DOWN	UP	UP	UP	DOWN
NOCHAN(	UP	UP	NOCHAN(	NOCHAN(	NOCHAN(	NOCHAN(	UP	NOCHAN(

TCGA-B2-	TCGA-A3-	TCGA-CZ-	TCGA-AK-	TCGA-CZ-	TCGA-B0-	TCGA-CZ-	TCGA-CJ-	TCGA-AK-
DOWN	NOCHANG	NOCHANG	NOCHANG	DOWN	DOWN	DOWN	DOWN	DOWN
DOWN	NOCHANG	NOCHANG	DOWN	DOWN	NOCHANG	DOWN	DOWN	NOCHANG
NOCHANG	DOWN	NOCHANG	DOWN	DOWN	DOWN	DOWN	DOWN	DOWN
NOCHANG	NOCHANG	NOCHANG	NOCHANG	NOCHANG	NOCHANG	NOCHANG	NOCHANG	NOCHANG
DOWN	NOCHANG	UP	DOWN	NOCHANG	NOCHANG	NOCHANG	NOCHANG	DOWN
UP	DOWN	NOCHANG	DOWN	DOWN	DOWN	DOWN	DOWN	NOCHANG
DOWN	DOWN	DOWN	NOCHANG	NOCHANG	DOWN	NOCHANG	DOWN	DOWN
DOWN	NOCHANG	NOCHANG	NOCHANG	NOCHANG	NOCHANG	NOCHANG	NOCHANG	NOCHANG
DOWN	NOCHANG	UP	UP	UP	UP	NOCHANG	UP	NOCHANG
UP	NOCHANG	NOCHANG	DOWN	NOCHANG	DOWN	NOCHANG	DOWN	NOCHANG
NOCHANG	NOCHANG	UP	NOCHANG	NOCHANG	NOCHANG	NOCHANG	NOCHANG	NOCHANG
DOWN	NOCHANG	NOCHANG	NOCHANG	NOCHANG	NOCHANG	NOCHANG	NOCHANG	NOCHANG
NOCHANG	NOCHANG	NOCHANG	NOCHANG	NOCHANG	DOWN	NOCHANG	DOWN	DOWN
DOWN	UP	NOCHANG	UP	UP	NOCHANG	NOCHANG	UP	NOCHANG
DOWN	NOCHANG	DOWN	DOWN	NOCHANG	NOCHANG	NOCHANG	NOCHANG	NOCHANG
NOCHANG	UP	NOCHANG	UP	NOCHANG	UP	UP	NOCHANG	UP
NOCHANG	NOCHANG	UP	DOWN	DOWN	NOCHANG	NOCHANG	DOWN	DOWN
UP	NOCHANG	NOCHANG	NOCHANG	NOCHANG	NOCHANG	NOCHANG	NOCHANG	NOCHANG
NOCHANG	NOCHANG	DOWN	DOWN	DOWN	DOWN	DOWN	DOWN	NOCHANG
NOCHANG	NOCHANG	DOWN	DOWN	DOWN	DOWN	DOWN	DOWN	NOCHANG
NOCHANG	NOCHANG	DOWN	DOWN	DOWN	DOWN	DOWN	DOWN	NOCHANG
DOWN	NOCHANG	NOCHANG	NOCHANG	NOCHANG	DOWN	NOCHANG	DOWN	DOWN
UP	UP	NOCHANG	UP	UP	UP	UP	UP	UP
NOCHANG	NOCHANG	NOCHANG	NOCHANG	DOWN	DOWN	DOWN	NOCHANG	NOCHANG
UP	NOCHANG	UP	DOWN	NOCHANG	NOCHANG	NOCHANG	NOCHANG	NOCHANG
DOWN	NOCHANG	NOCHANG	NOCHANG	NOCHANG	NOCHANG	DOWN	NOCHANG	DOWN
NOCHANG	DOWN	DOWN	DOWN	DOWN	DOWN	DOWN	DOWN	DOWN
UP	NOCHANG	NOCHANG	UP	NOCHANG	NOCHANG	NOCHANG	DOWN	NOCHANG
NOCHANG	NOCHANG	NOCHANG	DOWN	NOCHANG	NOCHANG	NOCHANG	NOCHANG	NOCHANG
NOCHANG	NOCHANG	DOWN	NOCHANG	NOCHANG	NOCHANG	NOCHANG	NOCHANG	NOCHANG
NOCHANG	NOCHANG	NOCHANG	NOCHANG	NOCHANG	NOCHANG	NOCHANG	NOCHANG	NOCHANG
UP	NOCHANG	NOCHANG	NOCHANG	DOWN	NOCHANG	NOCHANG	NOCHANG	NOCHANG
UP	NOCHANG	UP	NOCHANG	NOCHANG	UP	UP	NOCHANG	UP
UP	NOCHANG	NOCHANG	UP	NOCHANG	NOCHANG	NOCHANG	NOCHANG	NOCHANG
DOWN	NOCHANG	NOCHANG	NOCHANG	NOCHANG	UP	UP	NOCHANG	UP
NOCHANG	NOCHANG	NOCHANG	DOWN	NOCHANG	DOWN	NOCHANG	NOCHANG	NOCHANG
NOCHANG	DOWN	NOCHANG	DOWN	DOWN	DOWN	DOWN	DOWN	DOWN
NOCHANG	NOCHANG	NOCHANG	NOCHANG	UP	NOCHANG	NOCHANG	NOCHANG	NOCHANG
UP	UP	UP	UP	UP	NOCHANG	UP	NOCHANG	UP
DOWN	NOCHANG	UP	NOCHANG	NOCHANG	NOCHANG	NOCHANG	UP	NOCHANG

TCGA-B0-	TCGA-A3-	TCGA-B8-	TCGA-CJ-	TCGA-BP-	TCGA-A3-	TCGA-MV	TCGA-B0-	TCGA-CZ-
NOCHAN(	NOCHAN(	DOWN	DOWN	NOCHAN(	DOWN	DOWN	DOWN	NOCHAN(
DOWN	NOCHAN(	NOCHAN(	NOCHAN(	DOWN	NOCHAN(	NOCHAN(	NOCHAN(	NOCHAN(
DOWN	DOWN	NOCHAN(	DOWN	NOCHAN(	DOWN	DOWN	DOWN	DOWN
NOCHAN(	NOCHAN(	NOCHAN(	NOCHAN(	NOCHAN(	NOCHAN(	NOCHAN(	UP	NOCHAN(
NOCHAN(	NOCHAN(	NOCHAN(	NOCHAN(	UP	NOCHAN(	NOCHAN(	NOCHAN(	NOCHAN(
DOWN	NOCHAN(	NOCHAN(	NOCHAN(	NOCHAN(	DOWN	NOCHAN(	DOWN	NOCHAN(
NOCHAN(	DOWN	DOWN	NOCHAN(	UP	NOCHAN(	DOWN	NOCHAN(	DOWN
DOWN	UP	NOCHAN(	UP	UP	NOCHAN(	NOCHAN(	NOCHAN(	NOCHAN(
UP	NOCHAN(	NOCHAN(	UP	NOCHAN(	UP	UP	UP	UP
DOWN	DOWN	NOCHAN(	DOWN	NOCHAN(	NOCHAN(	NOCHAN(	DOWN	NOCHAN(
UP	UP	NOCHAN(	NOCHAN(	UP	NOCHAN(	NOCHAN(	UP	UP
DOWN	NOCHAN(	NOCHAN(	NOCHAN(	NOCHAN(	DOWN	NOCHAN(	NOCHAN(	NOCHAN(
NOCHAN(	UP	NOCHAN(	NOCHAN(	UP	NOCHAN(	NOCHAN(	DOWN	NOCHAN(
NOCHAN(	NOCHAN(	NOCHAN(	NOCHAN(	UP	UP	NOCHAN(	NOCHAN(	NOCHAN(
DOWN	NOCHAN(	NOCHAN(	DOWN	NOCHAN(	UP	NOCHAN(	NOCHAN(	NOCHAN(
UP	NOCHAN(	NOCHAN(	NOCHAN(	NOCHAN(	DOWN	UP	UP	NOCHAN(
UP	NOCHAN(	NOCHAN(	DOWN	UP	DOWN	NOCHAN(	NOCHAN(	NOCHAN(
NOCHAN(	UP	UP	NOCHAN(	UP	UP	NOCHAN(	UP	NOCHAN(
DOWN	NOCHAN(	DOWN	NOCHAN(	DOWN	DOWN	NOCHAN(	DOWN	NOCHAN(
NOCHAN(	NOCHAN(	DOWN	DOWN	DOWN	DOWN	NOCHAN(	NOCHAN(	DOWN
DOWN	NOCHAN(	NOCHAN(	DOWN	NOCHAN(	DOWN	DOWN	DOWN	DOWN
NOCHAN(	DOWN	NOCHAN(	NOCHAN(	NOCHAN(	NOCHAN(	NOCHAN(	NOCHAN(	NOCHAN(
UP	UP	UP	UP	UP	UP	UP	UP	UP
DOWN	NOCHAN(	NOCHAN(	NOCHAN(	NOCHAN(	NOCHAN(	NOCHAN(	NOCHAN(	NOCHAN(
NOCHAN(	NOCHAN(	NOCHAN(	NOCHAN(	NOCHAN(	NOCHAN(	NOCHAN(	NOCHAN(	NOCHAN(
NOCHAN(	NOCHAN(	NOCHAN(	DOWN	NOCHAN(	NOCHAN(	NOCHAN(	NOCHAN(	NOCHAN(
DOWN	DOWN	NOCHAN(	DOWN	NOCHAN(	NOCHAN(	DOWN	DOWN	DOWN
DOWN	NOCHAN(	NOCHAN(	NOCHAN(	NOCHAN(	UP	NOCHAN(	DOWN	NOCHAN(
NOCHAN(	NOCHAN(	NOCHAN(	DOWN	NOCHAN(	DOWN	UP	UP	NOCHAN(
DOWN	UP	NOCHAN(	NOCHAN(	NOCHAN(	NOCHAN(	NOCHAN(	NOCHAN(	NOCHAN(
NOCHAN(	NOCHAN(	NOCHAN(	UP	UP	NOCHAN(	UP	NOCHAN(	NOCHAN(
DOWN	NOCHAN(	NOCHAN(	NOCHAN(	DOWN	DOWN	NOCHAN(	NOCHAN(	NOCHAN(
NOCHAN(	UP	NOCHAN(	UP	UP	NOCHAN(	NOCHAN(	NOCHAN(	NOCHAN(
NOCHAN(	DOWN	NOCHAN(	NOCHAN(	NOCHAN(	DOWN	UP	NOCHAN(	NOCHAN(
DOWN	NOCHAN(	NOCHAN(	NOCHAN(	NOCHAN(	DOWN	UP	UP	UP
NOCHAN(	NOCHAN(	NOCHAN(	DOWN	UP	DOWN	NOCHAN(	DOWN	NOCHAN(
DOWN	NOCHAN(	DOWN	DOWN	NOCHAN(	DOWN	DOWN	DOWN	DOWN
NOCHAN(	NOCHAN(	NOCHAN(	UP	UP	NOCHAN(	NOCHAN(	UP	NOCHAN(
NOCHAN(	UP	UP	NOCHAN(	NOCHAN(	NOCHAN(	NOCHAN(	NOCHAN(	NOCHAN(
UP	NOCHAN(	UP	NOCHAN(	UP	UP	NOCHAN(	NOCHAN(	NOCHAN(

TCGA-B0-	TCGA-CZ-	TCGA-B0-	TCGA-B8-	TCGA-EU-	TCGA-CJ-	TCGA-B0-	TCGA-B0-	TCGA-B0-
DOWN	DOWN	NOCHAN(	NOCHAN(	DOWN	NOCHAN(	DOWN	DOWN	DOWN
NOCHAN(	DOWN	DOWN	DOWN	NOCHAN(	NOCHAN(	DOWN	DOWN	DOWN
DOWN	DOWN	DOWN	DOWN	DOWN	DOWN	DOWN	DOWN	DOWN
NOCHAN(	NOCHAN(	NOCHAN(	NOCHAN(	NOCHAN(	NOCHAN(	NOCHAN(	NOCHAN(	UP
NOCHAN(	NOCHAN(	NOCHAN(	NOCHAN(	NOCHAN(	NOCHAN(	NOCHAN(	UP	NOCHAN(
NOCHAN(	NOCHAN(	DOWN	NOCHAN(	DOWN	DOWN	NOCHAN(	DOWN	DOWN
NOCHAN(	NOCHAN(	DOWN	NOCHAN(	DOWN	NOCHAN(	NOCHAN(	NOCHAN(	DOWN
NOCHAN(	NOCHAN(	NOCHAN(	NOCHAN(	NOCHAN(	NOCHAN(	NOCHAN(	NOCHAN(	NOCHAN(
NOCHAN(	NOCHAN(	UP	UP	NOCHAN(	NOCHAN(	NOCHAN(	UP	UP
NOCHAN(	UP	NOCHAN(	NOCHAN(	DOWN	NOCHAN(	NOCHAN(	DOWN	DOWN
UP	NOCHAN(	UP	NOCHAN(	NOCHAN(	UP	NOCHAN(	UP	NOCHAN(
NOCHAN(	NOCHAN(	NOCHAN(	UP	DOWN	NOCHAN(	NOCHAN(	NOCHAN(	NOCHAN(
NOCHAN(	NOCHAN(	NOCHAN(	NOCHAN(	NOCHAN(	NOCHAN(	NOCHAN(	NOCHAN(	DOWN
NOCHAN(	DOWN	UP	NOCHAN(	NOCHAN(	NOCHAN(	NOCHAN(	UP	NOCHAN(
NOCHAN(	DOWN	NOCHAN(	NOCHAN(	NOCHAN(	NOCHAN(	NOCHAN(	NOCHAN(	NOCHAN(
NOCHAN(	NOCHAN(	NOCHAN(	UP	NOCHAN(	NOCHAN(	NOCHAN(	NOCHAN(	NOCHAN(
UP	UP	UP	NOCHAN(	DOWN	NOCHAN(	NOCHAN(	NOCHAN(	NOCHAN(
UP	NOCHAN(	NOCHAN(	NOCHAN(	UP	UP	NOCHAN(	UP	NOCHAN(
DOWN	NOCHAN(	DOWN	NOCHAN(	DOWN	DOWN	DOWN	DOWN	DOWN
NOCHAN(	DOWN	NOCHAN(	DOWN	NOCHAN(	NOCHAN(	DOWN	DOWN	DOWN
DOWN	NOCHAN(	DOWN	NOCHAN(	DOWN	DOWN	DOWN	DOWN	DOWN
NOCHAN(	DOWN	NOCHAN(	NOCHAN(	DOWN	NOCHAN(	NOCHAN(	NOCHAN(	DOWN
UP	NOCHAN(	UP	UP	UP	UP	UP	UP	UP
NOCHAN(	NOCHAN(	NOCHAN(	NOCHAN(	NOCHAN(	NOCHAN(	DOWN	NOCHAN(	DOWN
NOCHAN(	NOCHAN(	NOCHAN(	NOCHAN(	NOCHAN(	NOCHAN(	DOWN	NOCHAN(	NOCHAN(
NOCHAN(	DOWN	NOCHAN(	NOCHAN(	NOCHAN(	NOCHAN(	NOCHAN(	NOCHAN(	NOCHAN(
NOCHAN(	NOCHAN(	DOWN	DOWN	DOWN	DOWN	DOWN	DOWN	DOWN
NOCHAN(	NOCHAN(	NOCHAN(	UP	NOCHAN(	NOCHAN(	NOCHAN(	NOCHAN(	NOCHAN(
NOCHAN(	NOCHAN(	NOCHAN(	NOCHAN(	NOCHAN(	NOCHAN(	NOCHAN(	NOCHAN(	NOCHAN(
NOCHAN(	DOWN	NOCHAN(	NOCHAN(	DOWN	NOCHAN(	NOCHAN(	NOCHAN(	NOCHAN(
DOWN	NOCHAN(	UP	NOCHAN(	DOWN	NOCHAN(	NOCHAN(	UP	NOCHAN(
NOCHAN(	UP	NOCHAN(	NOCHAN(	NOCHAN(	NOCHAN(	NOCHAN(	NOCHAN(	DOWN
UP	UP	UP	NOCHAN(	NOCHAN(	NOCHAN(	NOCHAN(	UP	NOCHAN(
NOCHAN(	DOWN	NOCHAN(	NOCHAN(	NOCHAN(	NOCHAN(	NOCHAN(	NOCHAN(	NOCHAN(
NOCHAN(	NOCHAN(	NOCHAN(	UP	NOCHAN(	NOCHAN(	NOCHAN(	DOWN	NOCHAN(
NOCHAN(	NOCHAN(	NOCHAN(	NOCHAN(	DOWN	NOCHAN(	NOCHAN(	DOWN	DOWN
NOCHAN(	DOWN	DOWN	DOWN	DOWN	DOWN	DOWN	DOWN	DOWN
NOCHAN(	UP	NOCHAN(	UP	NOCHAN(	NOCHAN(	UP	UP	NOCHAN(
UP	UP	NOCHAN(	UP	UP	UP	NOCHAN(	NOCHAN(	NOCHAN(
UP	NOCHAN(	UP	NOCHAN(	NOCHAN(	NOCHAN(	NOCHAN(	UP	NOCHAN(

TCGA-A3-	TCGA-A3-	TCGA-BP-	TCGA-CJ-	TCGA-CJ-	TCGA-CJ-	TCGA-B0-	TCGA-B0-	TCGA-A3-
NOCHAN(	NOCHAN(	DOWN	NOCHAN(	DOWN	NOCHAN(	DOWN	DOWN	NOCHAN(
NOCHAN(	NOCHAN(	NOCHAN(	DOWN	DOWN	DOWN	DOWN	DOWN	NOCHAN(
DOWN	DOWN	DOWN	DOWN	DOWN	DOWN	DOWN	DOWN	DOWN
NOCHAN(	UP	NOCHAN(	NOCHAN(	UP	UP	NOCHAN(	UP	NOCHAN(
NOCHAN(	NOCHAN(	NOCHAN(	NOCHAN(	NOCHAN(	NOCHAN(	UP	NOCHAN(	NOCHAN(
DOWN	DOWN	DOWN	NOCHAN(	DOWN	NOCHAN(	DOWN	DOWN	DOWN
NOCHAN(	DOWN	DOWN	DOWN	NOCHAN(	DOWN	NOCHAN(	NOCHAN(	NOCHAN(
NOCHAN(	NOCHAN(	NOCHAN(	NOCHAN(	NOCHAN(	NOCHAN(	NOCHAN(	NOCHAN(	NOCHAN(
NOCHAN(	UP	UP	NOCHAN(	UP	UP	UP	UP	UP
NOCHAN(	NOCHAN(	NOCHAN(	NOCHAN(	DOWN	NOCHAN(	DOWN	NOCHAN(	NOCHAN(
NOCHAN(	NOCHAN(	NOCHAN(	UP	NOCHAN(	NOCHAN(	NOCHAN(	UP	NOCHAN(
NOCHAN(	NOCHAN(	NOCHAN(	NOCHAN(	NOCHAN(	NOCHAN(	DOWN	NOCHAN(	NOCHAN(
NOCHAN(	NOCHAN(	NOCHAN(	NOCHAN(	NOCHAN(	NOCHAN(	NOCHAN(	NOCHAN(	NOCHAN(
NOCHAN(	NOCHAN(	NOCHAN(	NOCHAN(	NOCHAN(	DOWN	NOCHAN(	NOCHAN(	NOCHAN(
NOCHAN(	NOCHAN(	NOCHAN(	NOCHAN(	NOCHAN(	UP	NOCHAN(	NOCHAN(	NOCHAN(
NOCHAN(	NOCHAN(	UP	NOCHAN(	NOCHAN(	NOCHAN(	NOCHAN(	NOCHAN(	NOCHAN(
NOCHAN(	NOCHAN(	NOCHAN(	NOCHAN(	NOCHAN(	NOCHAN(	NOCHAN(	NOCHAN(	UP
UP	NOCHAN(	NOCHAN(	UP	NOCHAN(	UP	NOCHAN(	NOCHAN(	UP
DOWN	DOWN	NOCHAN(	NOCHAN(	DOWN	NOCHAN(	DOWN	DOWN	DOWN
NOCHAN(	DOWN	NOCHAN(	NOCHAN(	DOWN	NOCHAN(	DOWN	NOCHAN(	DOWN
NOCHAN(	DOWN	NOCHAN(	NOCHAN(	DOWN	NOCHAN(	DOWN	NOCHAN(	NOCHAN(
NOCHAN(	NOCHAN(	NOCHAN(	NOCHAN(	NOCHAN(	NOCHAN(	NOCHAN(	NOCHAN(	NOCHAN(
UP	UP	UP	UP	UP	UP	UP	UP	UP
NOCHAN(	DOWN	NOCHAN(	NOCHAN(	NOCHAN(	NOCHAN(	DOWN	NOCHAN(	NOCHAN(
NOCHAN(	NOCHAN(	NOCHAN(	NOCHAN(	NOCHAN(	NOCHAN(	NOCHAN(	NOCHAN(	NOCHAN(
NOCHAN(	NOCHAN(	NOCHAN(	NOCHAN(	NOCHAN(	NOCHAN(	NOCHAN(	NOCHAN(	NOCHAN(
DOWN	DOWN	DOWN	NOCHAN(	DOWN	DOWN	DOWN	DOWN	NOCHAN(
NOCHAN(	NOCHAN(	NOCHAN(	NOCHAN(	NOCHAN(	NOCHAN(	DOWN	NOCHAN(	NOCHAN(
NOCHAN(	UP	NOCHAN(	UP	NOCHAN(	NOCHAN(	NOCHAN(	UP	NOCHAN(
DOWN	NOCHAN(	DOWN	NOCHAN(	UP	NOCHAN(	NOCHAN(	NOCHAN(	DOWN
NOCHAN(	NOCHAN(	NOCHAN(	DOWN	UP	NOCHAN(	UP	NOCHAN(	DOWN
NOCHAN(	DOWN	NOCHAN(	NOCHAN(	NOCHAN(	NOCHAN(	DOWN	NOCHAN(	NOCHAN(
UP	NOCHAN(	NOCHAN(	UP	NOCHAN(	UP	NOCHAN(	UP	UP
NOCHAN(	NOCHAN(	NOCHAN(	NOCHAN(	NOCHAN(	NOCHAN(	NOCHAN(	NOCHAN(	DOWN
NOCHAN(	UP	UP	NOCHAN(	NOCHAN(	UP	NOCHAN(	NOCHAN(	NOCHAN(
NOCHAN(	NOCHAN(	NOCHAN(	NOCHAN(	DOWN	NOCHAN(	DOWN	NOCHAN(	NOCHAN(
DOWN	DOWN	DOWN	DOWN	DOWN	DOWN	DOWN	DOWN	DOWN
UP	UP	UP	UP	NOCHAN(	UP	NOCHAN(	NOCHAN(	NOCHAN(
UP	UP	NOCHAN(	UP	DOWN	NOCHAN(	DOWN	UP	NOCHAN(
UP	UP	NOCHAN(	UP	NOCHAN(	UP	NOCHAN(	UP	UP

TCGA-BP-	TCGA-B0-	TCGA-B0-	TCGA-DV	TCGA-BP-	TCGA-B0-	TCGA-AK-	TCGA-CJ-	TCGA-A3-
NOCHANG	DOWN	DOWN	DOWN	DOWN	DOWN	NOCHANG	DOWN	DOWN
DOWN	DOWN	DOWN	DOWN	DOWN	NOCHANG	NOCHANG	DOWN	DOWN
DOWN	DOWN	DOWN	DOWN	DOWN	DOWN	NOCHANG	DOWN	DOWN
NOCHANG	NOCHANG	NOCHANG	NOCHANG	NOCHANG	NOCHANG	NOCHANG	UP	NOCHANG
NOCHANG	NOCHANG	DOWN	NOCHANG	NOCHANG	NOCHANG	DOWN	NOCHANG	NOCHANG
DOWN	DOWN	DOWN	DOWN	DOWN	DOWN	UP	DOWN	DOWN
DOWN	NOCHANG	NOCHANG	DOWN	NOCHANG	DOWN	UP	NOCHANG	NOCHANG
NOCHANG	NOCHANG	NOCHANG	UP	NOCHANG	UP	NOCHANG	DOWN	NOCHANG
UP	UP	UP	NOCHANG	UP	UP	DOWN	NOCHANG	NOCHANG
DOWN	NOCHANG	NOCHANG	NOCHANG	DOWN	DOWN	NOCHANG	DOWN	NOCHANG
NOCHANG	NOCHANG	NOCHANG	NOCHANG	NOCHANG	NOCHANG	NOCHANG	UP	NOCHANG
NOCHANG	NOCHANG	DOWN	DOWN	NOCHANG	NOCHANG	NOCHANG	NOCHANG	NOCHANG
NOCHANG	NOCHANG	DOWN	DOWN	DOWN	NOCHANG	DOWN	NOCHANG	NOCHANG
UP	NOCHANG	UP	NOCHANG	UP	NOCHANG	NOCHANG	NOCHANG	UP
NOCHANG	NOCHANG	NOCHANG	NOCHANG	UP	NOCHANG	NOCHANG	DOWN	NOCHANG
NOCHANG	NOCHANG	UP	NOCHANG	NOCHANG	NOCHANG	UP	UP	NOCHANG
NOCHANG	NOCHANG	NOCHANG	NOCHANG	NOCHANG	NOCHANG	UP	UP	NOCHANG
NOCHANG	NOCHANG	UP	NOCHANG	NOCHANG	NOCHANG	NOCHANG	NOCHANG	NOCHANG
DOWN	DOWN	DOWN	NOCHANG	DOWN	DOWN	UP	DOWN	NOCHANG
DOWN	DOWN	DOWN	NOCHANG	DOWN	DOWN	UP	NOCHANG	DOWN
DOWN	DOWN	DOWN	NOCHANG	DOWN	DOWN	UP	DOWN	DOWN
NOCHANG	NOCHANG	NOCHANG	DOWN	NOCHANG	NOCHANG	DOWN	NOCHANG	NOCHANG
UP	UP	UP	NOCHANG	UP	UP	DOWN	UP	UP
DOWN	DOWN	NOCHANG	NOCHANG	DOWN	DOWN	UP	NOCHANG	NOCHANG
NOCHANG	DOWN	DOWN	NOCHANG	DOWN	DOWN	NOCHANG	NOCHANG	DOWN
NOCHANG	DOWN	DOWN	NOCHANG	NOCHANG	NOCHANG	NOCHANG	NOCHANG	NOCHANG
DOWN	DOWN	DOWN	NOCHANG	DOWN	DOWN	NOCHANG	DOWN	DOWN
NOCHANG	DOWN	NOCHANG	DOWN	DOWN	NOCHANG	UP	DOWN	NOCHANG
NOCHANG	NOCHANG	NOCHANG	UP	DOWN	NOCHANG	UP	DOWN	DOWN
NOCHANG	NOCHANG	NOCHANG	NOCHANG	NOCHANG	NOCHANG	NOCHANG	NOCHANG	NOCHANG
UP	NOCHANG	NOCHANG	DOWN	UP	UP	DOWN	UP	NOCHANG
DOWN	NOCHANG	NOCHANG	NOCHANG	DOWN	DOWN	NOCHANG	NOCHANG	DOWN
NOCHANG	NOCHANG	NOCHANG	NOCHANG	NOCHANG	NOCHANG	NOCHANG	NOCHANG	NOCHANG
NOCHANG	NOCHANG	NOCHANG	NOCHANG	UP	NOCHANG	NOCHANG	UP	NOCHANG
UP	DOWN	NOCHANG	NOCHANG	DOWN	UP	DOWN	NOCHANG	NOCHANG
NOCHANG	NOCHANG	NOCHANG	NOCHANG	DOWN	DOWN	UP	NOCHANG	NOCHANG
DOWN	DOWN	DOWN	NOCHANG	DOWN	DOWN	NOCHANG	DOWN	DOWN
UP	NOCHANG	UP	NOCHANG	NOCHANG	UP	NOCHANG	NOCHANG	UP
DOWN	NOCHANG	UP	UP	DOWN	DOWN	UP	DOWN	NOCHANG
NOCHANG	NOCHANG	NOCHANG	UP	NOCHANG	NOCHANG	NOCHANG	NOCHANG	NOCHANG

TCGA-B0-	TCGA-BP-	TCGA-BP-	TCGA-BP-	TCGA-CJ-	TCGA-B4-	TCGA-BP-	TCGA-B2-	TCGA-BP-
DOWN	DOWN	DOWN	DOWN	NOCHAN(	NOCHAN(	DOWN	DOWN	NOCHAN(
DOWN	NOCHAN(	NOCHAN(	NOCHAN(	NOCHAN(	NOCHAN(	NOCHAN(	DOWN	NOCHAN(
DOWN	DOWN	NOCHAN(	DOWN	DOWN	DOWN	DOWN	NOCHAN(	NOCHAN(
NOCHAN(	NOCHAN(	NOCHAN(	UP	NOCHAN(	NOCHAN(	NOCHAN(	NOCHAN(	NOCHAN(
NOCHAN(	NOCHAN(	UP	UP	NOCHAN(	NOCHAN(	NOCHAN(	UP	NOCHAN(
DOWN	DOWN	NOCHAN(	DOWN	DOWN	NOCHAN(	NOCHAN(	NOCHAN(	NOCHAN(
NOCHAN(	NOCHAN(	DOWN	DOWN	NOCHAN(	NOCHAN(	NOCHAN(	NOCHAN(	DOWN
NOCHAN(	NOCHAN(	NOCHAN(	NOCHAN(	NOCHAN(	NOCHAN(	NOCHAN(	UP	NOCHAN(
UP	NOCHAN(	NOCHAN(	NOCHAN(	UP	NOCHAN(	NOCHAN(	DOWN	NOCHAN(
NOCHAN(	DOWN	DOWN	DOWN	DOWN	NOCHAN(	DOWN	UP	NOCHAN(
NOCHAN(	NOCHAN(	UP	UP	NOCHAN(	NOCHAN(	NOCHAN(	UP	UP
NOCHAN(	NOCHAN(	NOCHAN(	DOWN	NOCHAN(	NOCHAN(	NOCHAN(	DOWN	NOCHAN(
NOCHAN(	DOWN	NOCHAN(	NOCHAN(	NOCHAN(	DOWN	NOCHAN(	NOCHAN(	NOCHAN(
NOCHAN(	NOCHAN(	NOCHAN(	NOCHAN(	NOCHAN(	NOCHAN(	NOCHAN(	DOWN	NOCHAN(
NOCHAN(	NOCHAN(	UP	NOCHAN(	NOCHAN(	NOCHAN(	NOCHAN(	NOCHAN(	DOWN
UP	NOCHAN(	NOCHAN(	NOCHAN(	NOCHAN(	NOCHAN(	NOCHAN(	DOWN	NOCHAN(
UP	NOCHAN(	NOCHAN(	UP	NOCHAN(	NOCHAN(	NOCHAN(	UP	NOCHAN(
NOCHAN(	UP	NOCHAN(	UP	NOCHAN(	UP	UP	UP	NOCHAN(
DOWN	DOWN	DOWN	DOWN	DOWN	NOCHAN(	DOWN	DOWN	NOCHAN(
NOCHAN(	NOCHAN(	DOWN	NOCHAN(	DOWN	DOWN	DOWN	NOCHAN(	DOWN
NOCHAN(	DOWN	DOWN	DOWN	DOWN	NOCHAN(	DOWN	NOCHAN(	DOWN
NOCHAN(	DOWN	NOCHAN(	DOWN	NOCHAN(	NOCHAN(	DOWN	DOWN	NOCHAN(
UP	UP	UP	UP	UP	UP	UP	NOCHAN(	UP
NOCHAN(	NOCHAN(	NOCHAN(	NOCHAN(	DOWN	NOCHAN(	NOCHAN(	NOCHAN(	NOCHAN(
NOCHAN(	NOCHAN(	NOCHAN(	NOCHAN(	NOCHAN(	DOWN	NOCHAN(	UP	NOCHAN(
NOCHAN(	NOCHAN(	NOCHAN(	NOCHAN(	NOCHAN(	DOWN	NOCHAN(	NOCHAN(	NOCHAN(
DOWN	NOCHAN(	DOWN	NOCHAN(	NOCHAN(	DOWN	NOCHAN(	NOCHAN(	DOWN
NOCHAN(	NOCHAN(	NOCHAN(	NOCHAN(	NOCHAN(	DOWN	NOCHAN(	DOWN	NOCHAN(
NOCHAN(	NOCHAN(	NOCHAN(	UP	NOCHAN(	NOCHAN(	UP	NOCHAN(	NOCHAN(
DOWN	NOCHAN(	UP	NOCHAN(	NOCHAN(	DOWN	NOCHAN(	DOWN	NOCHAN(
NOCHAN(	NOCHAN(	NOCHAN(	NOCHAN(	NOCHAN(	DOWN	NOCHAN(	DOWN	NOCHAN(
NOCHAN(	NOCHAN(	NOCHAN(	NOCHAN(	NOCHAN(	NOCHAN(	NOCHAN(	NOCHAN(	NOCHAN(
UP	NOCHAN(	UP	UP	NOCHAN(	NOCHAN(	UP	UP	NOCHAN(
NOCHAN(	NOCHAN(	NOCHAN(	NOCHAN(	NOCHAN(	NOCHAN(	NOCHAN(	DOWN	NOCHAN(
NOCHAN(	NOCHAN(	NOCHAN(	NOCHAN(	UP	NOCHAN(	UP	DOWN	NOCHAN(
NOCHAN(	NOCHAN(	NOCHAN(	DOWN	DOWN	NOCHAN(	NOCHAN(	NOCHAN(	NOCHAN(
DOWN	DOWN	DOWN	DOWN	DOWN	DOWN	DOWN	NOCHAN(	DOWN
UP	NOCHAN(	UP	NOCHAN(	UP	UP	NOCHAN(	NOCHAN(	UP
UP	UP	NOCHAN(	UP	NOCHAN(	UP	NOCHAN(	NOCHAN(	NOCHAN(
UP	UP	NOCHAN(	UP	NOCHAN(	NOCHAN(	UP	UP	NOCHAN(

TCGA-B0-	TCGA-B0-	TCGA-BP-	TCGA-CJ-	TCGA-B0-	TCGA-BP-	TCGA-BP-	TCGA-BP-	TCGA-B4-
NOCHAN(	NOCHAN(	DOWN	DOWN	DOWN	NOCHAN(	DOWN	DOWN	DOWN
DOWN	DOWN	NOCHAN(	NOCHAN(	DOWN	DOWN	DOWN	DOWN	UP
DOWN	DOWN	DOWN	NOCHAN(	DOWN	DOWN	DOWN	DOWN	UP
NOCHAN(	NOCHAN(	NOCHAN(	NOCHAN(	NOCHAN(	NOCHAN(	NOCHAN(	NOCHAN(	DOWN
NOCHAN(	UP	NOCHAN(	NOCHAN(	NOCHAN(	NOCHAN(	NOCHAN(	NOCHAN(	DOWN
DOWN	DOWN	NOCHAN(	NOCHAN(	DOWN	NOCHAN(	DOWN	NOCHAN(	UP
NOCHAN(	DOWN	DOWN	DOWN	NOCHAN(	NOCHAN(	DOWN	NOCHAN(	NOCHAN(
NOCHAN(	UP	NOCHAN(	NOCHAN(	NOCHAN(	NOCHAN(	NOCHAN(	NOCHAN(	DOWN
UP	UP	NOCHAN(	NOCHAN(	NOCHAN(	UP	UP	NOCHAN(	DOWN
NOCHAN(	NOCHAN(	DOWN	DOWN	DOWN	NOCHAN(	DOWN	NOCHAN(	UP
UP	NOCHAN(	NOCHAN(	NOCHAN(	NOCHAN(	NOCHAN(	NOCHAN(	UP	NOCHAN(
NOCHAN(	DOWN	NOCHAN(	NOCHAN(	DOWN	NOCHAN(	UP	NOCHAN(	DOWN
NOCHAN(	UP	NOCHAN(	NOCHAN(	NOCHAN(	NOCHAN(	DOWN	NOCHAN(	UP
NOCHAN(	NOCHAN(	NOCHAN(	NOCHAN(	UP	NOCHAN(	NOCHAN(	NOCHAN(	DOWN
NOCHAN(	NOCHAN(	DOWN	NOCHAN(	NOCHAN(	DOWN	NOCHAN(	NOCHAN(	DOWN
UP	NOCHAN(	UP	NOCHAN(	UP	NOCHAN(	UP	UP	DOWN
NOCHAN(	UP	NOCHAN(	NOCHAN(	NOCHAN(	NOCHAN(	NOCHAN(	UP	DOWN
UP	UP	NOCHAN(	NOCHAN(	NOCHAN(	NOCHAN(	NOCHAN(	UP	NOCHAN(
DOWN	DOWN	NOCHAN(	DOWN	DOWN	DOWN	DOWN	NOCHAN(	UP
NOCHAN(	DOWN	DOWN	DOWN	NOCHAN(	DOWN	NOCHAN(	NOCHAN(	NOCHAN(
NOCHAN(	NOCHAN(	NOCHAN(	DOWN	DOWN	NOCHAN(	DOWN	NOCHAN(	UP
NOCHAN(	NOCHAN(	NOCHAN(	DOWN	NOCHAN(	NOCHAN(	DOWN	NOCHAN(	NOCHAN(
UP	UP	UP	UP	UP	UP	UP	UP	NOCHAN(
NOCHAN(	NOCHAN(	NOCHAN(	NOCHAN(	NOCHAN(	NOCHAN(	NOCHAN(	NOCHAN(	NOCHAN(
NOCHAN(	NOCHAN(	NOCHAN(	NOCHAN(	NOCHAN(	NOCHAN(	DOWN	NOCHAN(	UP
NOCHAN(	NOCHAN(	NOCHAN(	DOWN	DOWN	DOWN	NOCHAN(	NOCHAN(	DOWN
DOWN	DOWN	DOWN	DOWN	DOWN	DOWN	DOWN	DOWN	DOWN
NOCHAN(	NOCHAN(	NOCHAN(	NOCHAN(	NOCHAN(	NOCHAN(	NOCHAN(	NOCHAN(	DOWN
NOCHAN(	UP	NOCHAN(	NOCHAN(	DOWN	NOCHAN(	NOCHAN(	UP	DOWN
DOWN	DOWN	NOCHAN(	NOCHAN(	UP	DOWN	NOCHAN(	NOCHAN(	UP
NOCHAN(	NOCHAN(	NOCHAN(	NOCHAN(	UP	NOCHAN(	UP	NOCHAN(	DOWN
NOCHAN(	NOCHAN(	NOCHAN(	NOCHAN(	DOWN	NOCHAN(	NOCHAN(	NOCHAN(	UP
NOCHAN(	NOCHAN(	NOCHAN(	UP	NOCHAN(	NOCHAN(	UP	UP	UP
NOCHAN(	NOCHAN(	NOCHAN(	NOCHAN(	UP	NOCHAN(	NOCHAN(	NOCHAN(	DOWN
NOCHAN(	UP	NOCHAN(	NOCHAN(	UP	UP	NOCHAN(	NOCHAN(	DOWN
NOCHAN(	NOCHAN(	NOCHAN(	NOCHAN(	NOCHAN(	NOCHAN(	NOCHAN(	NOCHAN(	DOWN
DOWN	DOWN	NOCHAN(	DOWN	DOWN	DOWN	DOWN	NOCHAN(	DOWN
UP	NOCHAN(	NOCHAN(	UP	NOCHAN(	UP	UP	UP	UP
UP	NOCHAN(	NOCHAN(	NOCHAN(	NOCHAN(	UP	DOWN	UP	NOCHAN(
UP	UP	NOCHAN(	NOCHAN(	UP	NOCHAN(	DOWN	UP	DOWN

TCGA-B0-	TCGA-B8-	TCGA-CW	TCGA-MN	TCGA-CJ-	TCGA-CJ-	TCGA-BP-	TCGA-BP-	TCGA-B0-
DOWN	DOWN	DOWN	NOCHAN(	DOWN	DOWN	DOWN	DOWN	NOCHAN(
DOWN	NOCHAN(	NOCHAN(	DOWN	NOCHAN(	NOCHAN(	DOWN	NOCHAN(	DOWN
DOWN	NOCHAN(	DOWN	DOWN	NOCHAN(	DOWN	DOWN	NOCHAN(	DOWN
UP	NOCHAN(	NOCHAN(	UP	NOCHAN(	UP	NOCHAN(	UP	UP
UP	NOCHAN(	NOCHAN(	NOCHAN(	UP	NOCHAN(	DOWN	NOCHAN(	NOCHAN(
NOCHAN(	NOCHAN(	NOCHAN(	DOWN	DOWN	UP	DOWN	DOWN	DOWN
NOCHAN(	DOWN	NOCHAN(	DOWN	NOCHAN(	NOCHAN(	NOCHAN(	NOCHAN(	DOWN
NOCHAN(	NOCHAN(	NOCHAN(	NOCHAN(	UP	NOCHAN(	NOCHAN(	NOCHAN(	NOCHAN(
UP	NOCHAN(	NOCHAN(	UP	UP	NOCHAN(	NOCHAN(	NOCHAN(	UP
DOWN	NOCHAN(	NOCHAN(	DOWN	DOWN	DOWN	DOWN	NOCHAN(	DOWN
UP	NOCHAN(	NOCHAN(	NOCHAN(	NOCHAN(	UP	NOCHAN(	NOCHAN(	NOCHAN(
NOCHAN(	NOCHAN(	NOCHAN(	NOCHAN(	NOCHAN(	NOCHAN(	NOCHAN(	DOWN	NOCHAN(
NOCHAN(	NOCHAN(	NOCHAN(	NOCHAN(	NOCHAN(	DOWN	NOCHAN(	NOCHAN(	NOCHAN(
NOCHAN(	NOCHAN(	NOCHAN(	UP	NOCHAN(	NOCHAN(	NOCHAN(	NOCHAN(	UP
NOCHAN(	NOCHAN(	UP	NOCHAN(	NOCHAN(	DOWN	NOCHAN(	NOCHAN(	NOCHAN(
NOCHAN(	NOCHAN(	NOCHAN(	NOCHAN(	NOCHAN(	NOCHAN(	NOCHAN(	NOCHAN(	NOCHAN(
UP	NOCHAN(	NOCHAN(	NOCHAN(	NOCHAN(	UP	DOWN	NOCHAN(	NOCHAN(
NOCHAN(	NOCHAN(	UP	NOCHAN(	UP	UP	NOCHAN(	UP	NOCHAN(
DOWN	NOCHAN(	DOWN	DOWN	DOWN	DOWN	DOWN	DOWN	DOWN
NOCHAN(	DOWN	NOCHAN(	DOWN	DOWN	DOWN	DOWN	DOWN	DOWN
NOCHAN(	DOWN	NOCHAN(	DOWN	DOWN	DOWN	DOWN	NOCHAN(	DOWN
NOCHAN(	NOCHAN(	DOWN	NOCHAN(	NOCHAN(	DOWN	NOCHAN(	DOWN	NOCHAN(
UP	NOCHAN(	UP	UP	UP	UP	UP	NOCHAN(	UP
NOCHAN(	DOWN	NOCHAN(	DOWN	DOWN	DOWN	DOWN	NOCHAN(	DOWN
NOCHAN(	NOCHAN(	NOCHAN(	DOWN	NOCHAN(	UP	DOWN	NOCHAN(	NOCHAN(
NOCHAN(	NOCHAN(	NOCHAN(	NOCHAN(	NOCHAN(	NOCHAN(	DOWN	NOCHAN(	NOCHAN(
DOWN	NOCHAN(	NOCHAN(	DOWN	DOWN	NOCHAN(	DOWN	DOWN	DOWN
NOCHAN(	NOCHAN(	NOCHAN(	DOWN	NOCHAN(	NOCHAN(	DOWN	NOCHAN(	NOCHAN(
NOCHAN(	NOCHAN(	NOCHAN(	NOCHAN(	NOCHAN(	UP	NOCHAN(	UP	NOCHAN(
NOCHAN(	DOWN	NOCHAN(	NOCHAN(	NOCHAN(	DOWN	NOCHAN(	DOWN	NOCHAN(
NOCHAN(	NOCHAN(	NOCHAN(	UP	UP	UP	UP	DOWN	UP
NOCHAN(	NOCHAN(	NOCHAN(	DOWN	NOCHAN(	DOWN	DOWN	NOCHAN(	DOWN
UP	NOCHAN(	UP	NOCHAN(	UP	UP	NOCHAN(	UP	NOCHAN(
NOCHAN(	DOWN	NOCHAN(	NOCHAN(	NOCHAN(	NOCHAN(	NOCHAN(	DOWN	NOCHAN(
NOCHAN(	UP	UP	NOCHAN(	NOCHAN(	DOWN	NOCHAN(	NOCHAN(	NOCHAN(
NOCHAN(	NOCHAN(	NOCHAN(	DOWN	NOCHAN(	NOCHAN(	DOWN	NOCHAN(	NOCHAN(
DOWN	DOWN	DOWN	DOWN	DOWN	NOCHAN(	DOWN	NOCHAN(	DOWN
UP	NOCHAN(	NOCHAN(	NOCHAN(	UP	NOCHAN(	NOCHAN(	UP	UP
NOCHAN(	UP	UP	DOWN	NOCHAN(	DOWN	NOCHAN(	UP	NOCHAN(
UP	NOCHAN(	NOCHAN(	NOCHAN(	NOCHAN(	UP	NOCHAN(	UP	NOCHAN(

TCGA-CJ-	TCGA-BP-	TCGA-B0-	TCGA-CW	TCGA-BP-	TCGA-CW	TCGA-A3-	TCGA-B0-	TCGA-B0-
NOCHAN(	DOWN	NOCHAN(	NOCHAN(	DOWN	NOCHAN(	NOCHAN(	DOWN	DOWN
DOWN	DOWN	NOCHAN(	NOCHAN(	DOWN	NOCHAN(	DOWN	NOCHAN(	UP
DOWN	DOWN	DOWN	NOCHAN(	DOWN	DOWN	DOWN	DOWN	UP
NOCHAN(	NOCHAN(	NOCHAN(	NOCHAN(	UP	NOCHAN(	NOCHAN(	NOCHAN(	UP
DOWN	NOCHAN(	NOCHAN(	NOCHAN(	NOCHAN(	NOCHAN(	UP	NOCHAN(	UP
NOCHAN(	DOWN	NOCHAN(	DOWN	DOWN	DOWN	DOWN	NOCHAN(	NOCHAN(
NOCHAN(	NOCHAN(	NOCHAN(	NOCHAN(	DOWN	DOWN	NOCHAN(	NOCHAN(	DOWN
NOCHAN(	NOCHAN(	NOCHAN(	NOCHAN(	NOCHAN(	NOCHAN(	NOCHAN(	UP	NOCHAN(
UP	NOCHAN(	UP	UP	UP	UP	NOCHAN(	UP	UP
NOCHAN(	NOCHAN(	NOCHAN(	NOCHAN(	DOWN	DOWN	NOCHAN(	NOCHAN(	DOWN
NOCHAN(	UP	NOCHAN(	NOCHAN(	UP	NOCHAN(	UP	NOCHAN(	NOCHAN(
UP	DOWN	NOCHAN(	NOCHAN(	NOCHAN(	NOCHAN(	NOCHAN(	NOCHAN(	NOCHAN(
NOCHAN(	NOCHAN(	NOCHAN(	NOCHAN(	NOCHAN(	DOWN	NOCHAN(	NOCHAN(	NOCHAN(
UP	UP	NOCHAN(	NOCHAN(	NOCHAN(	NOCHAN(	NOCHAN(	UP	NOCHAN(
DOWN	NOCHAN(	NOCHAN(	NOCHAN(	NOCHAN(	NOCHAN(	NOCHAN(	NOCHAN(	NOCHAN(
UP	NOCHAN(	NOCHAN(	NOCHAN(	NOCHAN(	NOCHAN(	NOCHAN(	NOCHAN(	NOCHAN(
NOCHAN(	NOCHAN(	NOCHAN(	NOCHAN(	NOCHAN(	NOCHAN(	UP	DOWN	NOCHAN(
NOCHAN(	NOCHAN(	NOCHAN(	NOCHAN(	NOCHAN(	NOCHAN(	NOCHAN(	NOCHAN(	NOCHAN(
NOCHAN(	DOWN	NOCHAN(	DOWN	DOWN	DOWN	DOWN	DOWN	DOWN
DOWN	NOCHAN(	DOWN	DOWN	DOWN	NOCHAN(	DOWN	DOWN	DOWN
NOCHAN(	DOWN	NOCHAN(	DOWN	DOWN	DOWN	NOCHAN(	DOWN	DOWN
NOCHAN(	NOCHAN(	NOCHAN(	NOCHAN(	NOCHAN(	NOCHAN(	NOCHAN(	NOCHAN(	DOWN
NOCHAN(	UP	UP	UP	UP	UP	UP	UP	UP
NOCHAN(	NOCHAN(	DOWN	NOCHAN(	DOWN	NOCHAN(	NOCHAN(	NOCHAN(	DOWN
NOCHAN(	NOCHAN(	NOCHAN(	NOCHAN(	DOWN	NOCHAN(	NOCHAN(	NOCHAN(	NOCHAN(
DOWN	NOCHAN(	NOCHAN(	NOCHAN(	NOCHAN(	DOWN	NOCHAN(	NOCHAN(	NOCHAN(
DOWN	DOWN	DOWN	NOCHAN(	DOWN	NOCHAN(	NOCHAN(	DOWN	DOWN
NOCHAN(	NOCHAN(	NOCHAN(	NOCHAN(	NOCHAN(	NOCHAN(	NOCHAN(	NOCHAN(	DOWN
NOCHAN(	NOCHAN(	NOCHAN(	NOCHAN(	NOCHAN(	NOCHAN(	UP	NOCHAN(	UP
NOCHAN(	NOCHAN(	NOCHAN(	DOWN	NOCHAN(	NOCHAN(	DOWN	NOCHAN(	NOCHAN(
NOCHAN(	NOCHAN(	NOCHAN(	NOCHAN(	NOCHAN(	NOCHAN(	DOWN	UP	UP
NOCHAN(	NOCHAN(	NOCHAN(	NOCHAN(	DOWN	NOCHAN(	NOCHAN(	NOCHAN(	NOCHAN(
UP	UP	UP	NOCHAN(	NOCHAN(	NOCHAN(	NOCHAN(	UP	UP
NOCHAN(	NOCHAN(	NOCHAN(	NOCHAN(	NOCHAN(	NOCHAN(	DOWN	NOCHAN(	NOCHAN(
UP	DOWN	UP	NOCHAN(	UP	NOCHAN(	NOCHAN(	NOCHAN(	NOCHAN(
NOCHAN(	NOCHAN(	NOCHAN(	NOCHAN(	NOCHAN(	NOCHAN(	NOCHAN(	NOCHAN(	DOWN
DOWN	NOCHAN(	DOWN	NOCHAN(	DOWN	DOWN	DOWN	NOCHAN(	DOWN
UP	NOCHAN(	UP	UP	UP	NOCHAN(	NOCHAN(	UP	UP
NOCHAN(	UP	UP	UP	NOCHAN(	UP	UP	NOCHAN(	DOWN
NOCHAN(	UP	NOCHAN(	UP	NOCHAN(	UP	UP	NOCHAN(	UP

TCGA-BP-	TCGA-CJ-	TCGA-AK-	TCGA-A3-	TCGA-G6-	TCGA-B0-	TCGA-BP-	TCGA-B0-	TCGA-CW
NOCHAN(	NOCHAN(	NOCHAN(	NOCHAN(	DOWN	DOWN	DOWN	NOCHAN(	NOCHAN(
NOCHAN(	DOWN	NOCHAN(	DOWN	DOWN	DOWN	NOCHAN(	DOWN	UP
DOWN	DOWN	DOWN	DOWN	DOWN	DOWN	NOCHAN(	DOWN	NOCHAN(
NOCHAN(	NOCHAN(	NOCHAN(	UP	UP	NOCHAN(	NOCHAN(	NOCHAN(	NOCHAN(
NOCHAN(	NOCHAN(	NOCHAN(	NOCHAN(	NOCHAN(	NOCHAN(	NOCHAN(	NOCHAN(	NOCHAN(
DOWN	DOWN	NOCHAN(	DOWN	DOWN	DOWN	NOCHAN(	DOWN	NOCHAN(
NOCHAN(	NOCHAN(	NOCHAN(	NOCHAN(	DOWN	DOWN	NOCHAN(	DOWN	DOWN
NOCHAN(	NOCHAN(	NOCHAN(	UP	NOCHAN(	NOCHAN(	NOCHAN(	UP	NOCHAN(
NOCHAN(	NOCHAN(	NOCHAN(	UP	UP	UP	NOCHAN(	UP	NOCHAN(
NOCHAN(	DOWN	NOCHAN(	NOCHAN(	DOWN	DOWN	DOWN	DOWN	NOCHAN(
NOCHAN(	NOCHAN(	UP	NOCHAN(	NOCHAN(	NOCHAN(	UP	NOCHAN(	NOCHAN(
NOCHAN(	NOCHAN(	UP	NOCHAN(	NOCHAN(	NOCHAN(	NOCHAN(	DOWN	NOCHAN(
NOCHAN(	NOCHAN(	NOCHAN(	NOCHAN(	NOCHAN(	NOCHAN(	NOCHAN(	NOCHAN(	NOCHAN(
NOCHAN(	NOCHAN(	UP	NOCHAN(	UP	UP	NOCHAN(	UP	NOCHAN(
NOCHAN(	DOWN	NOCHAN(	NOCHAN(	DOWN	NOCHAN(	NOCHAN(	NOCHAN(	UP
NOCHAN(	NOCHAN(	UP	NOCHAN(	UP	NOCHAN(	NOCHAN(	DOWN	NOCHAN(
UP	NOCHAN(	NOCHAN(	NOCHAN(	NOCHAN(	NOCHAN(	NOCHAN(	DOWN	NOCHAN(
UP	UP	NOCHAN(	UP	NOCHAN(	NOCHAN(	NOCHAN(	NOCHAN(	NOCHAN(
DOWN	NOCHAN(	UP	DOWN	DOWN	NOCHAN(	NOCHAN(	DOWN	NOCHAN(
NOCHAN(	DOWN	DOWN	DOWN	DOWN	DOWN	DOWN	DOWN	NOCHAN(
NOCHAN(	DOWN	DOWN	NOCHAN(	DOWN	DOWN	DOWN	DOWN	NOCHAN(
NOCHAN(	NOCHAN(	NOCHAN(	DOWN	NOCHAN(	DOWN	NOCHAN(	NOCHAN(	NOCHAN(
UP	UP	UP	UP	UP	UP	UP	UP	NOCHAN(
NOCHAN(	DOWN	NOCHAN(	NOCHAN(	DOWN	NOCHAN(	NOCHAN(	DOWN	NOCHAN(
NOCHAN(	NOCHAN(	NOCHAN(	NOCHAN(	DOWN	DOWN	NOCHAN(	NOCHAN(	NOCHAN(
NOCHAN(	DOWN	NOCHAN(	NOCHAN(	NOCHAN(	NOCHAN(	DOWN	NOCHAN(	NOCHAN(
DOWN	DOWN	DOWN	NOCHAN(	DOWN	DOWN	DOWN	DOWN	NOCHAN(
NOCHAN(	NOCHAN(	UP	NOCHAN(	DOWN	DOWN	NOCHAN(	NOCHAN(	NOCHAN(
NOCHAN(	NOCHAN(	DOWN	UP	NOCHAN(	NOCHAN(	NOCHAN(	NOCHAN(	NOCHAN(
DOWN	NOCHAN(	NOCHAN(	DOWN	DOWN	NOCHAN(	NOCHAN(	DOWN	NOCHAN(
NOCHAN(	NOCHAN(	UP	NOCHAN(	UP	NOCHAN(	UP	UP	NOCHAN(
NOCHAN(	NOCHAN(	NOCHAN(	NOCHAN(	DOWN	NOCHAN(	NOCHAN(	DOWN	NOCHAN(
UP	NOCHAN(	NOCHAN(	UP	UP	NOCHAN(	NOCHAN(	NOCHAN(	NOCHAN(
NOCHAN(	NOCHAN(	NOCHAN(	NOCHAN(	UP	NOCHAN(	NOCHAN(	NOCHAN(	NOCHAN(
NOCHAN(	NOCHAN(	NOCHAN(	NOCHAN(	NOCHAN(	NOCHAN(	NOCHAN(	DOWN	NOCHAN(
NOCHAN(	NOCHAN(	NOCHAN(	NOCHAN(	NOCHAN(	NOCHAN(	NOCHAN(	DOWN	NOCHAN(
DOWN	DOWN	NOCHAN(	DOWN	DOWN	DOWN	DOWN	DOWN	NOCHAN(
NOCHAN(	UP	UP	NOCHAN(	NOCHAN(	NOCHAN(	NOCHAN(	NOCHAN(	NOCHAN(
UP	NOCHAN(	NOCHAN(	UP	NOCHAN(	NOCHAN(	UP	DOWN	NOCHAN(
UP	UP	NOCHAN(	UP	NOCHAN(	NOCHAN(	NOCHAN(	NOCHAN(	NOCHAN(

TCGA-B4-	TCGA-BP-	TCGA-BP-	TCGA-CJ-	TCGA-DV	TCGA-BP-	TCGA-BP-	TCGA-BP-	TCGA-CJ-
NOCHAN(	NOCHAN(	NOCHAN(	DOWN	DOWN	NOCHAN(	NOCHAN(	NOCHAN(	NOCHAN(
NOCHAN(	NOCHAN(	DOWN	DOWN	NOCHAN(	NOCHAN(	DOWN	NOCHAN(	DOWN
DOWN	DOWN	DOWN	DOWN	DOWN	DOWN	DOWN	DOWN	DOWN
NOCHAN(	NOCHAN(	NOCHAN(	UP	NOCHAN(	NOCHAN(	UP	NOCHAN(	UP
NOCHAN(	NOCHAN(	NOCHAN(	DOWN	NOCHAN(	NOCHAN(	NOCHAN(	NOCHAN(	NOCHAN(
DOWN	NOCHAN(	DOWN	DOWN	NOCHAN(	DOWN	DOWN	DOWN	DOWN
DOWN	NOCHAN(	NOCHAN(	DOWN	DOWN	DOWN	NOCHAN(	DOWN	DOWN
NOCHAN(	UP	NOCHAN(	NOCHAN(	NOCHAN(	NOCHAN(	NOCHAN(	UP	NOCHAN(
UP	UP	NOCHAN(	UP	UP	UP	UP	NOCHAN(	UP
NOCHAN(	NOCHAN(	NOCHAN(	DOWN	DOWN	NOCHAN(	DOWN	DOWN	DOWN
NOCHAN(	UP	NOCHAN(	NOCHAN(	NOCHAN(	NOCHAN(	NOCHAN(	NOCHAN(	NOCHAN(
NOCHAN(	NOCHAN(	NOCHAN(	NOCHAN(	NOCHAN(	NOCHAN(	NOCHAN(	NOCHAN(	NOCHAN(
NOCHAN(	NOCHAN(	NOCHAN(	DOWN	DOWN	NOCHAN(	NOCHAN(	NOCHAN(	NOCHAN(
NOCHAN(	NOCHAN(	NOCHAN(	UP	NOCHAN(	UP	UP	NOCHAN(	NOCHAN(
NOCHAN(	NOCHAN(	DOWN	NOCHAN(	UP	UP	NOCHAN(	NOCHAN(	NOCHAN(
NOCHAN(	NOCHAN(	NOCHAN(	UP	NOCHAN(	NOCHAN(	NOCHAN(	NOCHAN(	NOCHAN(
NOCHAN(	UP	UP	DOWN	NOCHAN(	NOCHAN(	UP	NOCHAN(	NOCHAN(
UP	UP	NOCHAN(	NOCHAN(	NOCHAN(	UP	UP	NOCHAN(	NOCHAN(
NOCHAN(	NOCHAN(	NOCHAN(	DOWN	DOWN	NOCHAN(	DOWN	DOWN	DOWN
NOCHAN(	NOCHAN(	NOCHAN(	DOWN	DOWN	NOCHAN(	NOCHAN(	DOWN	DOWN
DOWN	NOCHAN(	DOWN	DOWN	DOWN	DOWN	DOWN	DOWN	DOWN
NOCHAN(	DOWN	NOCHAN(	NOCHAN(	NOCHAN(	NOCHAN(	NOCHAN(	NOCHAN(	NOCHAN(
UP	UP	UP	UP	UP	UP	UP	UP	UP
DOWN	DOWN	NOCHAN(	DOWN	DOWN	DOWN	NOCHAN(	DOWN	DOWN
DOWN	NOCHAN(	NOCHAN(	DOWN	NOCHAN(	NOCHAN(	NOCHAN(	NOCHAN(	NOCHAN(
NOCHAN(	NOCHAN(	NOCHAN(	DOWN	DOWN	NOCHAN(	NOCHAN(	NOCHAN(	NOCHAN(
DOWN	DOWN	DOWN	DOWN	DOWN	DOWN	DOWN	DOWN	DOWN
NOCHAN(	DOWN	NOCHAN(	DOWN	DOWN	UP	NOCHAN(	NOCHAN(	NOCHAN(
NOCHAN(	UP	NOCHAN(	NOCHAN(	NOCHAN(	NOCHAN(	UP	UP	NOCHAN(
NOCHAN(	NOCHAN(	NOCHAN(	DOWN	NOCHAN(	NOCHAN(	NOCHAN(	NOCHAN(	NOCHAN(
NOCHAN(	DOWN	NOCHAN(	UP	NOCHAN(	NOCHAN(	NOCHAN(	NOCHAN(	NOCHAN(
NOCHAN(	NOCHAN(	NOCHAN(	DOWN	DOWN	NOCHAN(	DOWN	DOWN	DOWN
UP	UP	UP	NOCHAN(	NOCHAN(	UP	NOCHAN(	NOCHAN(	NOCHAN(
DOWN	NOCHAN(	NOCHAN(	NOCHAN(	NOCHAN(	DOWN	NOCHAN(	NOCHAN(	NOCHAN(
UP	NOCHAN(	NOCHAN(	UP	UP	NOCHAN(	NOCHAN(	NOCHAN(	NOCHAN(
NOCHAN(	NOCHAN(	NOCHAN(	DOWN	DOWN	NOCHAN(	NOCHAN(	NOCHAN(	NOCHAN(
DOWN	DOWN	DOWN	DOWN	DOWN	DOWN	DOWN	DOWN	DOWN
UP	NOCHAN(	UP	NOCHAN(	UP	NOCHAN(	NOCHAN(	UP	UP
UP	NOCHAN(	UP	NOCHAN(	DOWN	UP	NOCHAN(	UP	NOCHAN(
NOCHAN(	UP	UP	NOCHAN(	NOCHAN(	UP	UP	NOCHAN(	NOCHAN(

TCGA-CZ-	TCGA-B0-	TCGA-B8-	TCGA-BP-	TCGA-B2-	TCGA-AK-	TCGA-B4-	TCGA-BP-	TCGA-BP-
DOWN	DOWN	DOWN	NOCHANG	DOWN	DOWN	DOWN	DOWN	DOWN
DOWN	NOCHANG	NOCHANG	DOWN	NOCHANG	DOWN	NOCHANG	DOWN	DOWN
DOWN	DOWN	DOWN	DOWN	DOWN	DOWN	DOWN	DOWN	DOWN
NOCHANG	NOCHANG	UP	NOCHANG	NOCHANG	NOCHANG	NOCHANG	UP	NOCHANG
NOCHANG	NOCHANG	NOCHANG	NOCHANG	NOCHANG	NOCHANG	NOCHANG	NOCHANG	NOCHANG
DOWN	NOCHANG	DOWN	DOWN	DOWN	DOWN	NOCHANG	DOWN	NOCHANG
DOWN	NOCHANG	DOWN	NOCHANG	DOWN	DOWN	NOCHANG	DOWN	NOCHANG
NOCHANG	NOCHANG	NOCHANG	NOCHANG	UP	NOCHANG	UP	NOCHANG	UP
NOCHANG	UP	UP	NOCHANG	NOCHANG	UP	UP	NOCHANG	UP
DOWN	NOCHANG	DOWN	NOCHANG	NOCHANG	DOWN	NOCHANG	DOWN	DOWN
NOCHANG	NOCHANG	NOCHANG	UP	NOCHANG	NOCHANG	NOCHANG	NOCHANG	NOCHANG
NOCHANG	NOCHANG	NOCHANG	NOCHANG	NOCHANG	NOCHANG	NOCHANG	DOWN	DOWN
NOCHANG	NOCHANG	NOCHANG	NOCHANG	NOCHANG	NOCHANG	NOCHANG	NOCHANG	NOCHANG
UP	NOCHANG	NOCHANG	NOCHANG	NOCHANG	NOCHANG	NOCHANG	NOCHANG	NOCHANG
DOWN	NOCHANG	UP	NOCHANG	NOCHANG	DOWN	NOCHANG	NOCHANG	UP
UP	NOCHANG	NOCHANG	UP	NOCHANG	UP	NOCHANG	NOCHANG	NOCHANG
NOCHANG	DOWN	NOCHANG	UP	NOCHANG	NOCHANG	NOCHANG	NOCHANG	NOCHANG
NOCHANG	NOCHANG	NOCHANG	UP	NOCHANG	NOCHANG	NOCHANG	UP	UP
DOWN	DOWN	DOWN	NOCHANG	DOWN	DOWN	NOCHANG	DOWN	DOWN
DOWN	DOWN	NOCHANG	NOCHANG	DOWN	DOWN	DOWN	NOCHANG	DOWN
DOWN	DOWN	DOWN	NOCHANG	DOWN	DOWN	NOCHANG	DOWN	DOWN
NOCHANG	NOCHANG	NOCHANG	NOCHANG	NOCHANG	NOCHANG	NOCHANG	DOWN	NOCHANG
UP	UP	UP	UP	UP	UP	UP	UP	UP
DOWN	NOCHANG	DOWN	NOCHANG	NOCHANG	DOWN	NOCHANG	UP	NOCHANG
NOCHANG	NOCHANG	NOCHANG	NOCHANG	NOCHANG	NOCHANG	NOCHANG	NOCHANG	NOCHANG
NOCHANG	NOCHANG	NOCHANG	NOCHANG	NOCHANG	DOWN	NOCHANG	NOCHANG	NOCHANG
DOWN	DOWN	DOWN	DOWN	NOCHANG	DOWN	DOWN	DOWN	NOCHANG
NOCHANG	NOCHANG	NOCHANG	NOCHANG	NOCHANG	DOWN	NOCHANG	DOWN	NOCHANG
UP	NOCHANG	UP	NOCHANG	NOCHANG	NOCHANG	NOCHANG	UP	NOCHANG
DOWN	UP	NOCHANG	NOCHANG	DOWN	DOWN	NOCHANG	DOWN	NOCHANG
NOCHANG	NOCHANG	NOCHANG	NOCHANG	NOCHANG	UP	NOCHANG	NOCHANG	NOCHANG
DOWN	NOCHANG	NOCHANG	NOCHANG	DOWN	DOWN	NOCHANG	NOCHANG	NOCHANG
NOCHANG	UP	NOCHANG	NOCHANG	NOCHANG	NOCHANG	UP	NOCHANG	NOCHANG
NOCHANG	NOCHANG	NOCHANG	NOCHANG	NOCHANG	UP	NOCHANG	NOCHANG	NOCHANG
NOCHANG	DOWN	NOCHANG	NOCHANG	NOCHANG	DOWN	UP	NOCHANG	NOCHANG
NOCHANG	NOCHANG	NOCHANG	NOCHANG	NOCHANG	DOWN	NOCHANG	DOWN	NOCHANG
DOWN	NOCHANG	DOWN	DOWN	DOWN	DOWN	DOWN	DOWN	DOWN
NOCHANG	NOCHANG	NOCHANG	UP	NOCHANG	NOCHANG	UP	DOWN	NOCHANG
UP	NOCHANG	NOCHANG	UP	UP	DOWN	NOCHANG	UP	UP
NOCHANG	NOCHANG	UP	UP	UP	NOCHANG	NOCHANG	UP	NOCHANG

TCGA-B0-	TCGA-AS-	TCGA-CJ-	TCGA-CJ-	TCGA-AK-	TCGA-B8-	TCGA-BP-	TCGA-CJ-	TCGA-B0-
NOCHAN(	DOWN	NOCHAN(	NOCHAN(	DOWN	NOCHAN(	NOCHAN(	NOCHAN(	DOWN
DOWN	NOCHAN(	NOCHAN(	DOWN	NOCHAN(	DOWN	DOWN	NOCHAN(	NOCHAN(
DOWN	DOWN	DOWN	DOWN	DOWN	DOWN	DOWN	NOCHAN(	DOWN
UP	DOWN	NOCHAN(	UP	NOCHAN(	NOCHAN(	NOCHAN(	DOWN	NOCHAN(
NOCHAN(	DOWN	NOCHAN(	NOCHAN(	DOWN	NOCHAN(	NOCHAN(	NOCHAN(	NOCHAN(
DOWN	NOCHAN(	NOCHAN(	DOWN	NOCHAN(	DOWN	DOWN	NOCHAN(	DOWN
DOWN	NOCHAN(	NOCHAN(	NOCHAN(	NOCHAN(	DOWN	NOCHAN(	UP	DOWN
NOCHAN(	NOCHAN(	NOCHAN(	NOCHAN(	NOCHAN(	NOCHAN(	NOCHAN(	UP	NOCHAN(
UP	NOCHAN(	UP	UP	NOCHAN(	NOCHAN(	NOCHAN(	UP	UP
DOWN	DOWN	NOCHAN(	DOWN	NOCHAN(	DOWN	NOCHAN(	NOCHAN(	DOWN
UP	NOCHAN(	NOCHAN(	NOCHAN(	NOCHAN(	NOCHAN(	UP	NOCHAN(	NOCHAN(
NOCHAN(	UP	NOCHAN(	NOCHAN(	NOCHAN(	DOWN	DOWN	NOCHAN(	DOWN
NOCHAN(	NOCHAN(	NOCHAN(	NOCHAN(	NOCHAN(	NOCHAN(	NOCHAN(	NOCHAN(	DOWN
UP	NOCHAN(	NOCHAN(	NOCHAN(	NOCHAN(	NOCHAN(	NOCHAN(	UP	NOCHAN(
DOWN	NOCHAN(	NOCHAN(	DOWN	NOCHAN(	NOCHAN(	DOWN	UP	UP
NOCHAN(	NOCHAN(	NOCHAN(	NOCHAN(	UP	UP	UP	NOCHAN(	NOCHAN(
UP	DOWN	NOCHAN(	NOCHAN(	NOCHAN(	NOCHAN(	NOCHAN(	UP	NOCHAN(
NOCHAN(	NOCHAN(	NOCHAN(	NOCHAN(	NOCHAN(	NOCHAN(	NOCHAN(	UP	NOCHAN(
DOWN	NOCHAN(	NOCHAN(	DOWN	NOCHAN(	DOWN	DOWN	DOWN	DOWN
DOWN	DOWN	DOWN	DOWN	NOCHAN(	NOCHAN(	DOWN	DOWN	UP
DOWN	NOCHAN(	NOCHAN(	DOWN	NOCHAN(	NOCHAN(	NOCHAN(	UP	NOCHAN(
NOCHAN(	DOWN	NOCHAN(	NOCHAN(	NOCHAN(	NOCHAN(	DOWN	NOCHAN(	DOWN
UP	NOCHAN(	UP	UP	UP	UP	UP	UP	UP
NOCHAN(	NOCHAN(	DOWN	DOWN	NOCHAN(	NOCHAN(	NOCHAN(	NOCHAN(	UP
NOCHAN(	NOCHAN(	NOCHAN(	NOCHAN(	DOWN	NOCHAN(	NOCHAN(	NOCHAN(	NOCHAN(
NOCHAN(	NOCHAN(	NOCHAN(	NOCHAN(	DOWN	NOCHAN(	DOWN	NOCHAN(	NOCHAN(
DOWN	NOCHAN(	DOWN	DOWN	DOWN	DOWN	DOWN	NOCHAN(	NOCHAN(
DOWN	UP	NOCHAN(	NOCHAN(	NOCHAN(	NOCHAN(	NOCHAN(	NOCHAN(	UP
NOCHAN(	NOCHAN(	NOCHAN(	NOCHAN(	NOCHAN(	UP	NOCHAN(	NOCHAN(	UP
DOWN	NOCHAN(	NOCHAN(	NOCHAN(	NOCHAN(	DOWN	DOWN	UP	UP
UP	NOCHAN(	NOCHAN(	UP	NOCHAN(	NOCHAN(	DOWN	NOCHAN(	UP
NOCHAN(	DOWN	NOCHAN(	DOWN	NOCHAN(	NOCHAN(	NOCHAN(	NOCHAN(	DOWN
NOCHAN(	DOWN	NOCHAN(	NOCHAN(	UP	NOCHAN(	UP	NOCHAN(	UP
NOCHAN(	NOCHAN(	NOCHAN(	NOCHAN(	NOCHAN(	NOCHAN(	NOCHAN(	NOCHAN(	NOCHAN(
NOCHAN(	DOWN	UP	UP	UP	NOCHAN(	UP	NOCHAN(	NOCHAN(
NOCHAN(	NOCHAN(	NOCHAN(	DOWN	NOCHAN(	DOWN	NOCHAN(	UP	DOWN
DOWN	DOWN	DOWN	DOWN	NOCHAN(	DOWN	DOWN	NOCHAN(	DOWN
UP	DOWN	UP	UP	UP	NOCHAN(	NOCHAN(	UP	UP
DOWN	NOCHAN(	UP	DOWN	UP	UP	UP	NOCHAN(	DOWN
UP	NOCHAN(	DOWN	NOCHAN(	NOCHAN(	UP	NOCHAN(	UP	UP

TCGA-B0-	TCGA-BP-	TCGA-DV	TCGA-BP-	TCGA-AK-	TCGA-CW	TCGA-B0-	TCGA-B8-	TCGA-B0-
DOWN	DOWN	DOWN	DOWN	NOCHANG	NOCHANG	NOCHANG	DOWN	DOWN
NOCHANG	DOWN	NOCHANG	NOCHANG	NOCHANG	NOCHANG	NOCHANG	NOCHANG	DOWN
DOWN	DOWN	DOWN	DOWN	UP	DOWN	DOWN	DOWN	DOWN
NOCHANG	NOCHANG	NOCHANG	NOCHANG	NOCHANG	NOCHANG	NOCHANG	UP	UP
NOCHANG	NOCHANG	DOWN	NOCHANG	NOCHANG	NOCHANG	NOCHANG	NOCHANG	NOCHANG
NOCHANG	DOWN	NOCHANG	DOWN	NOCHANG	DOWN	DOWN	NOCHANG	DOWN
DOWN	DOWN	DOWN	NOCHANG	UP	DOWN	NOCHANG	NOCHANG	DOWN
NOCHANG	NOCHANG	NOCHANG	NOCHANG	NOCHANG	NOCHANG	UP	NOCHANG	NOCHANG
NOCHANG	UP	NOCHANG	NOCHANG	DOWN	UP	UP	UP	UP
DOWN	DOWN	NOCHANG	NOCHANG	NOCHANG	NOCHANG	DOWN	DOWN	DOWN
NOCHANG	NOCHANG	NOCHANG	NOCHANG	NOCHANG	NOCHANG	NOCHANG	NOCHANG	NOCHANG
NOCHANG	DOWN	NOCHANG	NOCHANG	NOCHANG	NOCHANG	NOCHANG	NOCHANG	NOCHANG
NOCHANG	DOWN	NOCHANG	NOCHANG	DOWN	NOCHANG	DOWN	DOWN	NOCHANG
NOCHANG	NOCHANG	NOCHANG	NOCHANG	NOCHANG	NOCHANG	NOCHANG	DOWN	NOCHANG
NOCHANG	DOWN	NOCHANG	NOCHANG	NOCHANG	NOCHANG	NOCHANG	NOCHANG	NOCHANG
NOCHANG	NOCHANG	NOCHANG	NOCHANG	NOCHANG	UP	UP	NOCHANG	NOCHANG
NOCHANG	DOWN	NOCHANG	NOCHANG	UP	NOCHANG	NOCHANG	NOCHANG	NOCHANG
UP	NOCHANG	NOCHANG	NOCHANG	DOWN	NOCHANG	UP	UP	NOCHANG
DOWN	DOWN	DOWN	DOWN	UP	DOWN	DOWN	DOWN	DOWN
DOWN	DOWN	DOWN	DOWN	DOWN	DOWN	DOWN	DOWN	NOCHANG
DOWN	DOWN	DOWN	DOWN	UP	NOCHANG	DOWN	DOWN	DOWN
DOWN	NOCHANG	NOCHANG	NOCHANG	NOCHANG	NOCHANG	NOCHANG	DOWN	DOWN
UP	UP	UP	UP	DOWN	UP	UP	UP	UP
NOCHANG	DOWN	DOWN	NOCHANG	UP	NOCHANG	NOCHANG	NOCHANG	NOCHANG
NOCHANG	DOWN	DOWN	NOCHANG	NOCHANG	NOCHANG	UP	NOCHANG	NOCHANG
DOWN	DOWN	NOCHANG	NOCHANG	NOCHANG	NOCHANG	UP	NOCHANG	NOCHANG
NOCHANG	DOWN	DOWN	NOCHANG	NOCHANG	DOWN	DOWN	NOCHANG	DOWN
DOWN	DOWN	NOCHANG	NOCHANG	NOCHANG	NOCHANG	NOCHANG	DOWN	NOCHANG
NOCHANG	DOWN	UP	NOCHANG	NOCHANG	NOCHANG	NOCHANG	UP	UP
NOCHANG	DOWN	NOCHANG	NOCHANG	NOCHANG	DOWN	NOCHANG	NOCHANG	NOCHANG
NOCHANG	UP	NOCHANG	NOCHANG	NOCHANG	NOCHANG	UP	NOCHANG	UP
NOCHANG	DOWN	NOCHANG	DOWN	NOCHANG	NOCHANG	DOWN	NOCHANG	NOCHANG
UP	DOWN	NOCHANG	UP	NOCHANG	NOCHANG	UP	NOCHANG	UP
NOCHANG	NOCHANG	NOCHANG	NOCHANG	NOCHANG	NOCHANG	NOCHANG	NOCHANG	NOCHANG
NOCHANG	NOCHANG	UP	DOWN	DOWN	NOCHANG	DOWN	NOCHANG	UP
DOWN	DOWN	NOCHANG	NOCHANG	UP	NOCHANG	NOCHANG	DOWN	NOCHANG
DOWN	DOWN	DOWN	DOWN	NOCHANG	DOWN	DOWN	DOWN	DOWN
NOCHANG	DOWN	NOCHANG	NOCHANG	UP	UP	NOCHANG	NOCHANG	UP
NOCHANG	DOWN	UP	UP	NOCHANG	UP	NOCHANG	NOCHANG	DOWN
NOCHANG	NOCHANG	NOCHANG	UP	NOCHANG	NOCHANG	NOCHANG	UP	UP

TCGA-BP-	TCGA-EU-	TCGA-BP-	TCGA-B0-	TCGA-DV	TCGA-M[illegible]	TCGA-BP-	TCGA-CJ-	TCGA-A3-
NOCHANG	DOWN	DOWN	NOCHANG	DOWN	NOCHANG	DOWN	DOWN	DOWN
DOWN	NOCHANG	NOCHANG	NOCHANG	NOCHANG	DOWN	NOCHANG	NOCHANG	DOWN
DOWN	DOWN	DOWN	DOWN	DOWN	DOWN	DOWN	DOWN	DOWN
NOCHANG	NOCHANG	NOCHANG	NOCHANG	NOCHANG	UP	NOCHANG	NOCHANG	UP
NOCHANG	NOCHANG	NOCHANG	DOWN	NOCHANG	NOCHANG	NOCHANG	NOCHANG	NOCHANG
DOWN	NOCHANG	DOWN	NOCHANG	NOCHANG	DOWN	DOWN	DOWN	DOWN
DOWN	DOWN	DOWN	DOWN	DOWN	DOWN	NOCHANG	NOCHANG	DOWN
NOCHANG	NOCHANG	NOCHANG	NOCHANG	NOCHANG	NOCHANG	NOCHANG	UP	NOCHANG
UP	NOCHANG	UP	NOCHANG	UP	UP	UP	UP	UP
DOWN	NOCHANG	DOWN	NOCHANG	DOWN	DOWN	NOCHANG	DOWN	DOWN
UP	NOCHANG	NOCHANG	NOCHANG	NOCHANG	NOCHANG	NOCHANG	NOCHANG	NOCHANG
NOCHANG	NOCHANG	NOCHANG	NOCHANG	DOWN	NOCHANG	NOCHANG	NOCHANG	NOCHANG
NOCHANG	NOCHANG	NOCHANG	NOCHANG	DOWN	NOCHANG	NOCHANG	DOWN	NOCHANG
NOCHANG	NOCHANG	NOCHANG	NOCHANG	NOCHANG	NOCHANG	NOCHANG	NOCHANG	UP
DOWN	NOCHANG	NOCHANG	DOWN	NOCHANG	NOCHANG	NOCHANG	NOCHANG	NOCHANG
NOCHANG	NOCHANG	NOCHANG	NOCHANG	NOCHANG	NOCHANG	NOCHANG	NOCHANG	NOCHANG
UP	NOCHANG	NOCHANG	DOWN	NOCHANG	NOCHANG	NOCHANG	NOCHANG	NOCHANG
NOCHANG	UP	NOCHANG	NOCHANG	NOCHANG	NOCHANG	NOCHANG	NOCHANG	NOCHANG
DOWN	NOCHANG	DOWN	DOWN	NOCHANG	DOWN	NOCHANG	DOWN	DOWN
DOWN	DOWN	DOWN	DOWN	NOCHANG	NOCHANG	DOWN	DOWN	NOCHANG
DOWN	NOCHANG	DOWN	NOCHANG	NOCHANG	DOWN	NOCHANG	DOWN	DOWN
NOCHANG	NOCHANG	NOCHANG	NOCHANG	DOWN	NOCHANG	NOCHANG	NOCHANG	NOCHANG
UP	UP	UP	UP	NOCHANG	UP	UP	UP	UP
DOWN	NOCHANG	NOCHANG	DOWN	NOCHANG	NOCHANG	NOCHANG	NOCHANG	DOWN
NOCHANG	NOCHANG	NOCHANG	NOCHANG	NOCHANG	NOCHANG	NOCHANG	NOCHANG	NOCHANG
NOCHANG	NOCHANG	DOWN	NOCHANG	NOCHANG	NOCHANG	NOCHANG	NOCHANG	NOCHANG
DOWN	NOCHANG	DOWN	DOWN	NOCHANG	DOWN	DOWN	DOWN	DOWN
NOCHANG	NOCHANG	NOCHANG	NOCHANG	DOWN	NOCHANG	NOCHANG	NOCHANG	NOCHANG
NOCHANG	NOCHANG	NOCHANG	UP	UP	NOCHANG	NOCHANG	NOCHANG	UP
DOWN	DOWN	NOCHANG	DOWN	NOCHANG	NOCHANG	DOWN	NOCHANG	NOCHANG
NOCHANG	NOCHANG	NOCHANG	NOCHANG	DOWN	UP	NOCHANG	NOCHANG	NOCHANG
DOWN	NOCHANG	NOCHANG	NOCHANG	NOCHANG	DOWN	NOCHANG	DOWN	DOWN
NOCHANG	NOCHANG	NOCHANG	NOCHANG	NOCHANG	NOCHANG	NOCHANG	NOCHANG	NOCHANG
NOCHANG	NOCHANG	NOCHANG	NOCHANG	NOCHANG	UP	NOCHANG	NOCHANG	NOCHANG
NOCHANG	NOCHANG	UP	UP	NOCHANG	NOCHANG	UP	UP	NOCHANG
NOCHANG	NOCHANG	NOCHANG	NOCHANG	DOWN	NOCHANG	NOCHANG	DOWN	NOCHANG
DOWN	DOWN	DOWN	DOWN	NOCHANG	DOWN	DOWN	DOWN	DOWN
NOCHANG	NOCHANG	UP	NOCHANG	NOCHANG	UP	NOCHANG	UP	NOCHANG
DOWN	UP	NOCHANG	UP	UP	NOCHANG	UP	NOCHANG	NOCHANG
UP	NOCHANG	NOCHANG	NOCHANG	UP	NOCHANG	UP	NOCHANG	UP

TCGA-B0-	TCGA-DV	TCGA-AK-	TCGA-B0-	TCGA-B0-	TCGA-BP-	TCGA-B8-	TCGA-CZ-	TCGA-DV-
DOWN	DOWN	NOCHAN(	DOWN	DOWN	NOCHAN(	DOWN	DOWN	NOCHANC
DOWN	DOWN	NOCHAN(	DOWN	NOCHAN(	DOWN	NOCHAN(	NOCHAN(	NOCHANC
DOWN	DOWN	NOCHAN(	DOWN	DOWN	DOWN	UP	DOWN	DOWN
UP	NOCHAN(	NOCHAN(	UP	NOCHAN(	UP	DOWN	NOCHAN(	NOCHANC
NOCHAN(	NOCHAN(	NOCHAN(	NOCHAN(	NOCHAN(	DOWN	DOWN	NOCHAN(	NOCHANC
DOWN	DOWN	NOCHAN(	DOWN	DOWN	NOCHAN(	UP	DOWN	NOCHANC
DOWN	DOWN	UP	DOWN	NOCHAN(	UP	NOCHAN(	NOCHAN(	DOWN
NOCHAN(	NOCHAN(	NOCHAN(	NOCHAN(	UP	DOWN	DOWN	NOCHAN(	NOCHANC
UP	UP	NOCHAN(	NOCHAN(	NOCHAN(	UP	DOWN	UP	UP
DOWN	DOWN	NOCHAN(	DOWN	DOWN	DOWN	UP	DOWN	NOCHANC
NOCHAN(	NOCHAN(	NOCHAN(	NOCHAN(	NOCHAN(	NOCHAN(	UP	NOCHAN(	NOCHANC
NOCHAN(	NOCHAN(	NOCHAN(	NOCHAN(	NOCHAN(	UP	DOWN	DOWN	NOCHANC
NOCHAN(	NOCHAN(	NOCHAN(	NOCHAN(	NOCHAN(	NOCHAN(	UP	NOCHAN(	NOCHANC
NOCHAN(	NOCHAN(	NOCHAN(	UP	NOCHAN(	UP	DOWN	NOCHAN(	NOCHANC
DOWN	NOCHAN(	NOCHAN(	NOCHAN(	NOCHAN(	DOWN	DOWN	NOCHAN(	NOCHANC
UP	UP	NOCHAN(	UP	NOCHAN(	NOCHAN(	DOWN	NOCHAN(	NOCHANC
UP	NOCHAN(	NOCHAN(	NOCHAN(	NOCHAN(	UP	NOCHAN(	NOCHAN(	NOCHANC
NOCHAN(	NOCHAN(	UP	NOCHAN(	NOCHAN(	NOCHAN(	NOCHAN(	NOCHAN(	NOCHANC
DOWN	DOWN	NOCHAN(	DOWN	DOWN	NOCHAN(	UP	DOWN	DOWN
NOCHAN(	DOWN	DOWN	DOWN	DOWN	DOWN	NOCHAN(	NOCHAN(	DOWN
DOWN	DOWN	NOCHAN(	DOWN	DOWN	NOCHAN(	UP	DOWN	NOCHANC
DOWN	NOCHAN(	NOCHAN(	DOWN	NOCHAN(	DOWN	NOCHAN(	NOCHAN(	NOCHANC
UP	UP	UP	UP	UP	NOCHAN(	NOCHAN(	UP	UP
NOCHAN(	DOWN	NOCHAN(	DOWN	DOWN	DOWN	NOCHAN(	NOCHAN(	DOWN
NOCHAN(	DOWN	NOCHAN(	DOWN	NOCHAN(	DOWN	UP	NOCHAN(	NOCHANC
NOCHAN(	DOWN	NOCHAN(	DOWN	NOCHAN(	NOCHAN(	DOWN	NOCHAN(	NOCHANC
DOWN	DOWN	NOCHAN(	DOWN	DOWN	DOWN	DOWN	DOWN	DOWN
DOWN	NOCHAN(	NOCHAN(	DOWN	NOCHAN(	NOCHAN(	DOWN	NOCHAN(	NOCHANC
NOCHAN(	NOCHAN(	NOCHAN(	NOCHAN(	NOCHAN(	NOCHAN(	DOWN	NOCHAN(	NOCHANC
NOCHAN(	NOCHAN(	NOCHAN(	DOWN	NOCHAN(	NOCHAN(	UP	NOCHAN(	NOCHANC
NOCHAN(	UP	NOCHAN(	UP	UP	NOCHAN(	DOWN	UP	NOCHANC
DOWN	DOWN	DOWN	DOWN	DOWN	DOWN	UP	NOCHAN(	NOCHANC
UP	NOCHAN(	NOCHAN(	NOCHAN(	NOCHAN(	DOWN	UP	NOCHAN(	NOCHANC
NOCHAN(	NOCHAN(	NOCHAN(	NOCHAN(	UP	UP	DOWN	NOCHAN(	NOCHANC
NOCHAN(	UP	NOCHAN(	UP	UP	DOWN	DOWN	DOWN	UP
NOCHAN(	NOCHAN(	UP	NOCHAN(	NOCHAN(	NOCHAN(	NOCHAN(	NOCHAN(	NOCHANC
DOWN	DOWN	NOCHAN(	DOWN	DOWN	NOCHAN(	DOWN	DOWN	DOWN
NOCHAN(	NOCHAN(	UP	NOCHAN(	UP	NOCHAN(	UP	NOCHAN(	UP
NOCHAN(	NOCHAN(	NOCHAN(	NOCHAN(	NOCHAN(	DOWN	UP	UP	UP
UP	NOCHAN(	NOCHAN(	NOCHAN(	NOCHAN(	NOCHAN(	DOWN	UP	NOCHANC

-5575-01A-01R-1541-07
GE
GE

GE
GE
GE

GE

GE
GE
GE
GE
GE
GE
GE
GE
GE

GE
GE

GE
GE

GE
GE
GE
GE
GE
GE
GE

GE

GE

Table S8: uniCox analysis in KIRC

id	HR	HR.95L	HR.95H	pvalue
age	1.0297	1.01588	1.04372	2.19E-05
grade	2.28263	1.84053	2.83093	5.73E-14
stage	1.92576	1.67699	2.21144	1.60E-20
T	1.97255	1.66095	2.34262	9.65E-15
M	4.49933	3.25415	6.22096	9.22E-20
riskScore	11.512	6.68473	19.8252	1.25E-18

Table S9: multiCox analysis in KIRC

id	HR	HR.95L	HR.95H	pvalue
age	1.03297	1.01805	1.04812	1.25E-05
grade	1.30203	1.01919	1.66336	0.03468
stage	1.63265	1.06679	2.49867	0.02396
T	0.84674	0.57446	1.24809	0.40068
M	1.37948	0.72059	2.64087	0.33157
riskScore	5.61022	3.10308	10.143	1.14E-08
